# The Manchurian Walnut Genome: Insights into Juglone and Lipid Biosynthesis

**DOI:** 10.1093/gigascience/giac057

**Published:** 2022-06-28

**Authors:** Xiang Li, Kewei Cai, Qinhui Zhang, Xiaona Pei, Song Chen, Luping Jiang, Zhiming Han, Minghui Zhao, Yan Li, Xinxin Zhang, Yuxi Li, Shikai Zhang, Su Chen, Guanzheng Qu, Mulualem Tigabu, Vincent L Chiang, Ronald Sederoff, Xiyang Zhao

**Affiliations:** College of Forestry and Grassland, Jilin Agricultural University, Changchun 130117, China; State Key Laboratory of Tree Genetics and Breeding, School of Forestry, Northeast Forestry University, Harbin 150040, China; State Key Laboratory of Tree Genetics and Breeding, School of Forestry, Northeast Forestry University, Harbin 150040, China; State Key Laboratory of Tree Genetics and Breeding, School of Forestry, Northeast Forestry University, Harbin 150040, China; College of Forestry and Grassland, Jilin Agricultural University, Changchun 130117, China; State Key Laboratory of Tree Genetics and Breeding, School of Forestry, Northeast Forestry University, Harbin 150040, China; State Key Laboratory of Tree Genetics and Breeding, School of Forestry, Northeast Forestry University, Harbin 150040, China; State Key Laboratory of Tree Genetics and Breeding, School of Forestry, Northeast Forestry University, Harbin 150040, China; State Key Laboratory of Tree Genetics and Breeding, School of Forestry, Northeast Forestry University, Harbin 150040, China; State Key Laboratory of Tree Genetics and Breeding, School of Forestry, Northeast Forestry University, Harbin 150040, China; State Key Laboratory of Tree Genetics and Breeding, School of Forestry, Northeast Forestry University, Harbin 150040, China; State Key Laboratory of Tree Genetics and Breeding, School of Forestry, Northeast Forestry University, Harbin 150040, China; State Key Laboratory of Tree Genetics and Breeding, School of Forestry, Northeast Forestry University, Harbin 150040, China; State Key Laboratory of Tree Genetics and Breeding, School of Forestry, Northeast Forestry University, Harbin 150040, China; State Key Laboratory of Tree Genetics and Breeding, School of Forestry, Northeast Forestry University, Harbin 150040, China; Southern Swedish Forest Research Center, Faculty of Forest Science, Swedish University of Agricultural Sciences, Lomma SE-234 22, Sweden; State Key Laboratory of Tree Genetics and Breeding, School of Forestry, Northeast Forestry University, Harbin 150040, China; Forest Biotechnology Group, Department of Forestry and Environmental Resources, North Carolina State University, Raleigh, NC 27695, USA; Forest Biotechnology Group, Department of Forestry and Environmental Resources, North Carolina State University, Raleigh, NC 27695, USA; College of Forestry and Grassland, Jilin Agricultural University, Changchun 130117, China; State Key Laboratory of Tree Genetics and Breeding, School of Forestry, Northeast Forestry University, Harbin 150040, China

**Keywords:** Juglans mandshurica, PacBio SMART, Hi-C, HiFi, genome assembly, comparative genomics, juglone, lipid

## Abstract

**Background:**

Manchurian walnut (*Juglans mandshurica* Maxim.) is a tree with multiple industrial uses and medicinal properties in the Juglandaceae family (walnuts and hickories). *J. mandshurica* produces juglone, which is a toxic allelopathic agent and has potential utilization value. Furthermore, the seed of *J. mandshurica* is rich in various unsaturated fatty acids and has high nutritive value.

**Findings:**

Here, we present a high-quality chromosome-scale reference genome assembly and annotation for *J. mandshurica* (*n* = 16) with a contig N50 of 21.4 Mb by combining PacBio high-fidelity reads with high-throughput chromosome conformation capture data. The assembled genome has an estimated sequence size of 548.7 Mb and consists of 657 contigs, 623 scaffolds, and 40,453 protein-coding genes. In total, 60.99% of the assembled genome consists of repetitive sequences. Sixteen super-scaffolds corresponding to the 16 chromosomes were assembled, with a scaffold N50 length of 33.7 Mb and a BUSCO complete gene percentage of 98.3%. *J. mandshurica* displays a close sequence relationship with *Juglans cathayensis*, with a divergence time of 13.8 million years ago. Combining the high-quality genome, transcriptome, and metabolomics data, we constructed a gene-to-metabolite network and identified 566 core and conserved differentially expressed genes, which may be involved in juglone biosynthesis. Five *CYP450* genes were found that may contribute to juglone accumulation. NAC, bZip, NF-YA, and NF-YC are positively correlated with the juglone content. Some candidate regulators (e.g., FUS3, ABI3, LEC2, and WRI1 transcription factors) involved in the regulation of lipid biosynthesis were also identified.

**Conclusions:**

Our genomic data provide new insights into the evolution of the walnut genome and create a new platform for accelerating molecular breeding and improving the comprehensive utilization of these economically important tree species.

## Background

*Juglans mandshurica* Maxim. (NCBI:txid91218; 2n = 2x = 32), well known as “Manchurian walnut”, is a fast-growing and valuable hardwood tree species. The family Juglandaceae contains ∼23 species, all bearing edible and medicinal nuts [1]. *J. mandshurica* was widely cultivated in China, Korea, Siberia, Japan, India, and Russia. It is naturally distributed in the northeast regions of China [2]. Because of its highly desirable wood quality and medically active substances, *J. mandshurica* was widely used in construction, wood processing, oil production, medicine, and pesticide manufacturing. Its immature walnut peel (exocarp) contains bioactive components, including quinones, triterpenoids, flavonoids, phenolics, and alkaloids, which can induce detumescence and analgesia, softening blood vessels and producing anti-inflammatory effects. Juglone (5-hydroxy-1,4-naphthoquinone, C_10_H_6_O_3_) was used for its anticancer activity [3–5]. The walnut embryos of *J. mandshurica* have a high fatty acid content (more than 60%), which is composed of linoleic acid, oleic acid, linolenic acid, palmitic acid, and stearic acid. These fatty acids may aid in the prevention of coronary heart disease by decreasing blood lipids, enhancing immune functions, and modulating nonalcoholic fatty liver disease [6, 7]. In *J. mandshurica*, other tissues, including roots, stems, leaves, branches, and bark, may also have significant medicinal value [8].

Juglone forms orange acicular (long and needle-like) crystals and exhibits various biological activities and multipurpose applications. Juglone is a naphthoquinone heterocyclic compound that was first isolated and purified in the 1950s and artificially synthesized in 1887 [9]. Juglone has antibacterial, antitumor, antiviral, and anti-inflammatory effects and is mainly derived from the root, bark, leaves and immature walnut exocarp (peel) tissues of some Juglandaceae species, including *Carya cathayensis, Carya illinoinensis, Juglans cathayensis, Juglans hindsii, Juglans nigra, Juglans regia, Juglans sigillata*, and *Juglans macrocarpa* [3, 9]. Juglone was considered a potential new drug, and thus its separation, preparation, synthesis, and biological activities have been extensively studied [10–12]. In humans, juglone has antitumor activity and can significantly inhibit liver, colon, lung, and pancreatic cancer [13]. Juglone and related naphthoquinones are enriched in immature walnut exocarp compared with bark and root. Further research is needed for effectively extracting high-purity juglone. Juglone has toxic effects on some plants, showing obvious allelopathy, and therefore has been used as an effective bioherbicide [9]. There remains great potential to utilize juglone for allelopathy, synthetic agrochemicals, and natural colorants in agriculture. *J. mandshurica* might become a crucial plant resource for biomedical research. Targeted studies on its biosynthesis and molecular function are needed to explore its application to human health and economic development.

Understanding the regulation of genes involved in the biosynthesis of juglone in *J. mandshurica* could accelerate the utilization of juglone resources. There are at least 4 different natural metabolic pathways to synthesize 1,4-naphthoquinones (1,4-NQs) [14]. However, the biosynthesis and regulation of juglone remain unknown in plants, and only one primary biosynthetic pathway and a small number of genes are known based on the study of roots and leaves in black walnut (*J. nigra*) [15]. The biosynthesis of juglone is related to the phylloquinone (vitamin K1) pathway, which shares the 1,4-dihydroxynaphthoic acid (DHNA) to synthesize 1,4-NQs by decarboxylases. Ultimately, 1,4-NQs react with 2-oxoglutarate/Fe (II)–dependent dioxygenase (2-ODD) families or by cytochrome P450s to form juglone. Additional transcription factors (TFs) and other elements may also participate in juglone biosynthesis. The biosynthesis of juglone has not been systematically studied by multiomics and genome-based methods. To implement such a strategy, a genome sequence is needed.

In this work, we assembled a high-quality chromosome-level reference genome (548.7 Mb) of *J. mandshurica* by high-fidelity (HiFi) long reads based on the Pacific Biosciences (PacBio) sequencing platform and high-throughput chromosome conformation capture (Hi-C). We detected and annotated 40,453 gene models and 24,415 gene families in *J. mandshurica*. The analysis of gene family evolution and divergence was also performed in this study. We screened potential transcription factors and candidate genes involved in the juglone and lipid biosynthesis pathways. We mapped a gene-to-metabolite network by combining data on the genome, transcriptome, and metabolome. This work provides valuable genetic information on the evolution of *J. mandshurica* and related species and contributes to further elucidation of the juglone and lipid biosynthetic pathways.

## Analysis

### Sequencing and assembly of the *Juglans mandshurica* genome

**Figure 1:**
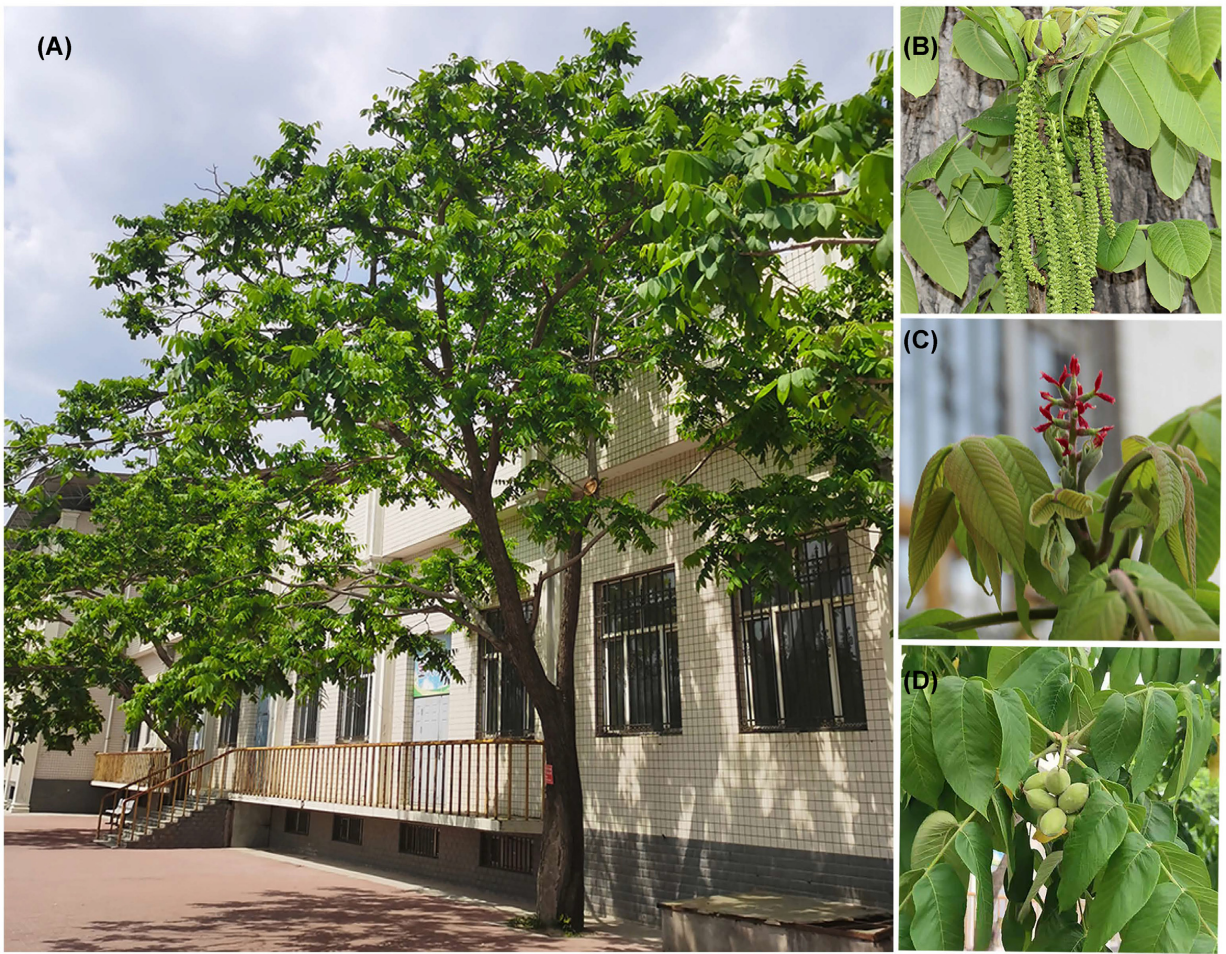
Photographs of *J. mandshurica*. (A) Adult tree. (B) Male flower. (C) Female flower. (D) Mature fruit.

A single adult Manchurian walnut tree (*J. mandshurica*) from northeast China was selected for whole-genome sequencing and assembly (Fig. 1). The genome size, estimated through k-mer analysis, was approximately 547.99 megabases (Mb), with a 0.77% level of heterozygosity and 48.78% of repeat sequences. A total of 14,596,746,422 17-mers were identified based on HiFi sequencing data, and the 17-mer depth was 26 (Supplementary Table S1 and Fig. S1). To assemble this highly heterozygous genome, Illumina, PacBio, and Hi-C technologies were selected for whole-genome sequencing. A total of 14.62 gigabases (Gb), circular consensus sequencing HiFi long reads with a nearly 26× sequence depth (Supplementary Fig. S2), and 56 Gb WGS Illumina short reads were obtained, using Sequel II and Illumina HiSeq 2500 platforms, respectively (Table 1). HiFiasm [16] software was used to assemble the *J. mandshurica* genome. After primary correction and assembly, the initial contig number, total length, and N50 size were 657, 548,677,591 bp (∼548.7 Mb), and 21,388,210 bp (∼21.4 Mb), respectively. Finally, 623 long scaffolds were anchored and oriented on 16 pseudochromosomes with a scaffold N50 of 35,382,463 bp (∼35.38 Mb). The genome size observed in the present study was similar to the results of k-mer analysis based on PacBio HiFi data, which may be attributable to the high-quality sequencing data and assembly. The *J. mandshurica* assembly was further improved using Hi-C paired-end reads using 51 Gb of Hi-C data in Lachesis [17]. A total of 50 contigs and 16 scaffolds were obtained after Hi-C assembly; the contigs N50 and scaffolds N50 were 21.4 Mb and 35.4 Mb, respectively. Consequently, 528 Mb were distributed across 16 chromosome-scale scaffolds and occupied 96.26% of the final genome assembly. Chromosome numbering for *J. mandshurica* was based on the size of chromosomes from maximum (Chr1) to minimum (Chr16). In particular, the assembly quality of *J. mandshurica* in the present study showed high-level comparability with the 2 genome assembly versions of this species reported previously (Table 1).

**Table 1:** The statistics for genome sequencing of *J. mandshurica* (V 3.0) compared with previously reported 2 genome assemblies of this species

Genomic features	*Juglans mandshurica* (V 3.0)	*Juglans mandshurica* (V 2.0)	*Juglans mandshurica* (V 1.0)
Sequence method	PacBio	Nanopore	Illumina
Raw bases (Gb)	14.62	62.87 Gb	*
Raw bases of Hi-C (Gb)	51	101 Gb	*
Raw bases of WGS Illumina (Gb)	56	47.3 Gb	49.05 Gb
Genome size (Mb)	548.7	548.5	558.1
Number of scaffolds after assembly	*	213 (≥2 Kb)	*
N50 of scaffolds (bp) after assembly	*	7,154,770 (≥2 Kb)	*
Number of contigs after assembly	657	215 (≥2 Kb)	*
N50 of contigs (bp) after assembly	21,388,210	7,154,770 (≥2 Kb)	*
Number of scaffolds after Hi-C + assembly	623	189	13,810
N50 of scaffolds (bp) Hi-C + assembly	35,382,463	36,084,664	496,923
Number of contigs Hi-C + assembly	657	397	24,385
N50 of contigs (bp) Hi-C + assembly	21,388,210	6,490,758	114,334
Anchored rate (%)	96.26	99.00	*
Complete BUSCOs (%)	98.3%	92%	*
GC content of the genome (%)	36.72%	38.51%	*
Number of predicted protein-coding genes	40,453	27,901	*
Average gene length (bp)	3,694.76	5,735	*
Average CDS length (bp)	1,104.82	1,226.35	*
Average exon number per gene	6.1	6.06	*
Average exon length (bp)	289.02	244.1	*
Number of tRNA	2,185	581	*
Number of rRNA	4,004	348	*
Number of miRNA	122	132	*
Number of snRNA	272	792	*
Repeat sequences (bp)	334,673,373 (60.99%)	340,401,005 (62.08%)	322,670,024 (50.48%)
Annotated to Interpro	25,953 (64.16%)	25,016 (86.17%)	*
Annotated to GO	18,229 (45.06%)	10,155 (34.98%)	*
Annotated to KEGG_ALL	31,243 (77.23%)	20,806 (71.67%)	*
Annotated to Swiss-Prot	23,059 (57.00%)	20,902 (72.00%)	*
Annotated to NR	32,855 (81.22%)	27,815 (95.81%)	*
DNA TEs	51,543,175 (9.39%)	49,110,954 (8.96%)	*
LINE TEs	45,973,640 (8.38%)	67,022,583 (12.22%)	*
SINE TEs	1,897,331 (0.35%)	58,768 (0.01%)	*
LTR TEs	215,930,087 (39.35%)	226,061,071 (41.23%)	*
Total TEs	326,580,986 (59.52%)	342,253,376 (62.42%)	*

Asterisk (*) represents data were not shown in the original articles. Hi-C, high-throughput chromosome conformation; CDS, coding sequence; GC, guanine and cytosine content; LINE, long interspersed nuclear element; LTR, long terminal repeat; miRNA, microRNA; rRNA, ribosomal RNA; SINE, short interspersed nuclear element; snRNA, small nuclear RNA; TE, transposable element; tRNA, transfer RNA.

The BUSCO software [18] was employed to evaluate the completeness of the *J. mandshurica* assembly. BUSCO assessment showed that 98.3% of the complete BUSCO gene set was captured, indicating increased BUSCO evaluation score (Supplementary Table S2). The mapping rate between the subreads obtained via PacBio sequencing and *J. mandshurica* assembly was 99.44%, and the coverage rate was 99.56%, thereby highlighting the assembly's high integrity (Supplementary Table S3). Additionally, statistical analysis for single-nucleotide polymorphisms (SNPs) and insertions and deletions (indel) showed that the proportion of homozygous SNPs and indels in *the J. mandshurica* assembly was not more than 0.01%, suggesting that the assembly was of high quality (Supplementary Table S4). Furthermore, among the total number of clean bases of mRNA sequencing (Gb), an average of 51,559,765 (93.17%) and 55,542,790 (93.05%) reads were obtained from the transcriptome sequence (RNA sequencing [RNA-seq]) in walnut exocarp and kernel, respectively, which could be mapped back to the genome assembly (Supplementary Table S5). These results for the assembly and assessment verified that we obtained a high-quality reference genome with a high degree of completeness at the chromosome level.

### Gene prediction and annotation of the *Juglans mandshurica* genome

**Figure 2:**
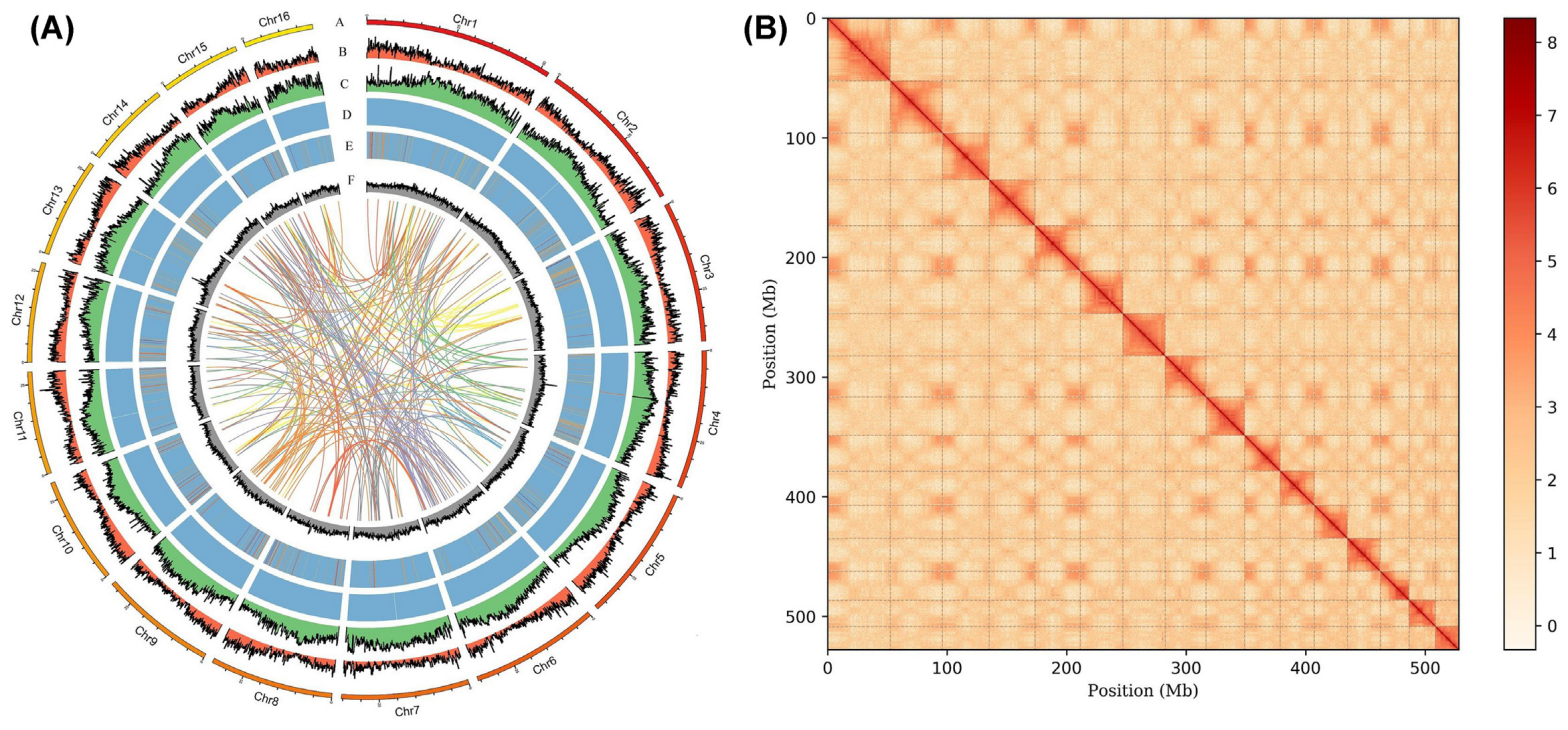
Genome information and Hi-C interaction heatmap of *J. mandshurica*. (A) Distribution of *J. mandshurica* genomic features. A = circular representation of the chromosome; B = gene density; C = repeat sequence density; D = rRNA density; E = tRNA density; F = GC content density. (B) Intensity signal heatmap of the Hi-C chromosome. The color in the figure from light to dark indicates the increase in the intensity of interaction.

In total, 40,515 protein-coding genes were predicted, with an average gene length of 3,694.76 bp, by combining the *de novo*, transcriptome, or Isoform sequencing (Iso-seq) and homology-based methods using MAKER (v3.0) [19] (Supplementary Table S6). For these predicted genes, 40,453 (99.85%) of the genes were anchored to pseudochromosomes, and the average exon number per gene was 6.1 with an average length of 289 bp, and the average coding sequence length was 1,105 bp (Supplementary Fig. S3). The total guanine and cytosine content (GC) content of the genome assembly was 36.7% and was distributed across 16 pseudochromosomes (Table 1 and Fig. 2). Among these genes, 32,901 (81.33%) of the genes were functionally annotated to InterPro (25,953, 64.16%), Gene Ontology (GO) (18,229, 45.06%), KEGG (31,243, 77.23%), Swiss-Prot (23,059, 57%), TrEMBL (31,341, 77.48%), and NR (32,855, 81.22%) public databases, and a total of 16,218 core genes were annotated in the abovementioned databases (Supplementary Table S7 and Supplementary Fig. S4). Gene distribution in 16 pseudochromosomes of the *J. mandshurica* genome was uneven, as found in other plant species, such as *J. regia* × *J. macrocarpa, J. regia, Rhododendron simsii* (azalea), and *Sechium edule* (chayote) [20–23] (Fig. 2). We identified several noncoding RNA genes, containing 122 microRNAs (miRNAs), 2,185 transfer RNAs (tRNAs), 4,004 ribosomal RNAs (rRNAs), and 272 small nuclear RNAs (snRNAs) in the *J. mandshurica* genome (Supplementary Table S8 and Fig. 2).

Furthermore, we identified 334,673,373 bp of repetitive sequences combining the *de novo* and homology-based approaches in *J. mandshurica*, and we accounted for 60.99% of the genome assembly (Supplementary Table S9 and Fig. 2). In total, 326,580,986 bp (59.52%) of transposable elements (TEs) were found, which was comparable to *Acer truncatum* (Shantung maple; ∼61.75%) [24] and *Eucommia ulmoides* (hardy rubber tree; ∼62.5%) [25]; however, it was higher than that in *Tripterygium wilfordii* (thunder duke vine; 52.36%) [26] and *Betula platyphylla* (white birch; 43.0%) [27]. The predominant TEs were long terminal repeat (LTR) retrotransposons, accounting for 39.35% of the assembled genome, followed by the DNA transposons (9.39%), long interspersed nuclear elements (LINEs, 8.38%), and short interspersed nuclear elements (SINEs, 0.35%) (Supplementary Table S9). Most TEs were gypsy and Copias-like LTRs that covered 65,135,736 bp and 93,428,433 bp respectively, and accounted for 11.87% and 17.03% in the assembled genome (Supplementary Table S10 and Supplementary Table S11).

### Gene family identification and evolutionary analysis

**Figure 3:**
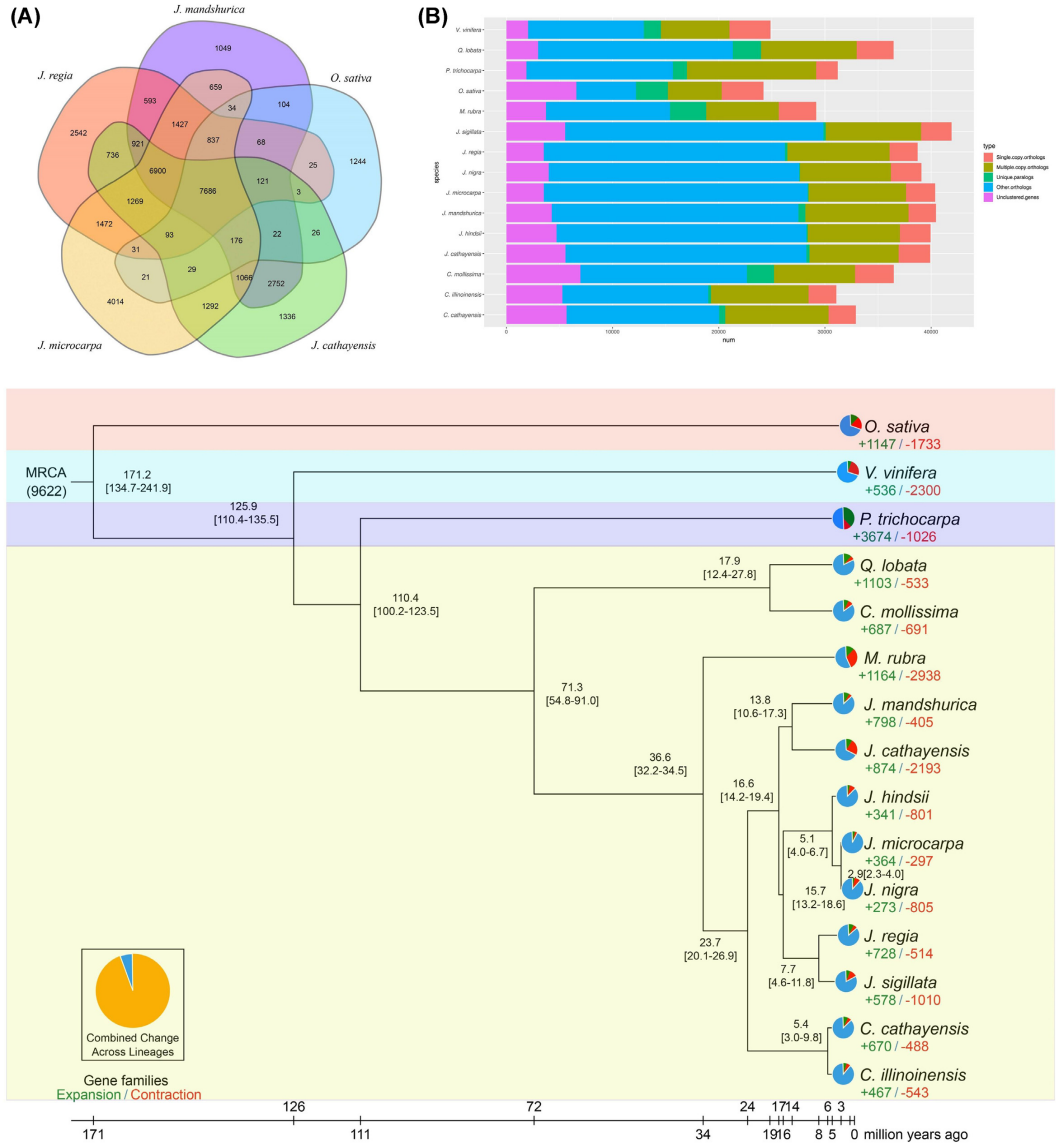
Phylogenetic analyses of the *J. mandshurica* genome. (A) Venn diagram showing the shared and unique gene families among *J. mandshurica* and 4 other species (*J. cathayensis, J. regia, J. macrocarpa*, and *O. sativa*). (B) An overview of orthologous and paralogous genes among *J. mandshurica* and related species. “Single-copy orthologs” include common orthologs with 1 copy in specific species. “Unique paralogs” include genes that do not have any similarity to genes in the other species based on BLAST and OrthoMCL. “Multicopy orthologs” include common orthologs with multiple copy numbers in specific species. “Unclustered” include genes that cannot be clustered into known gene families. “Other orthologs” include genes from families shared in 2 to 15 species. (C) Phylogenetic tree of 15 species including *J. mandshurica, J. cathayensis, J. macrocarpa, J. nigra, J. regia, J. hindsii, J. sigillata, C. cathayensis, C. illinoinensis, Q. lobata, C. mollissima, M. rubra, P. trichocarpa, V. vinifera*, and *O. sativa* based on orthologs of single-gene families. Blue numbers at each node represent the estimated time of each divergent event. Green and orange numbers along each branch indicate the number of expanded and contracted gene families, respectively. Pie charts show the proportions of gene families that underwent expansion or contraction.

*J. mandshurica* shared 7,686 gene families with 4 related plant species and possessed 2,584 single-copy orthologs and 225 unique families (Fig. 3A and Supplementary Table S12). In particular, the number of single- and multiple-copy genes of *J. mandshurica* was similar to that in the other genus *Juglans* species (Fig. 3B and Supplementary Table S13). To examine the genome evolution of *J. mandshurica* and the Juglandaceae family, 558 single-copy orthologous genes from 13 species of rosid families (i.e., *J. cathayensis, J. macrocarpa, J. nigra, J. regia, J. hindsii, J. sigillata, C. cathayensis, C. illinoinensis, Quercus lobata* [valley oak], *Castanea mollissima* [Chinese chestnut], *Morella rubra* [red bayberry], *Populus trichocarpa*, and *Vitis vinifera*) and 1 commelinid species (*Oryza sativa*) were identified via OrthoMCL [28] and employed to construct a phylogenetic tree and evaluate the divergence times using RAxML (version 8.2.11) [29] with default settings (Fig. 3C). A total of 40,453 genes were clustered into 24,415 (60.35%) gene families in *J. mandshurica* with an average of 1.48 genes per family (Supplementary Table S12). *J. mandshurica* was closely related to *J. cathayensis*, with an estimated divergence time of 13.8 (10.6–17.3) million years ago (mya). Species in the genus *Juglans* were clustered in the same group; they shared a common ancestor with species in the genus *Carya*, having diverged approximately 23.7 (20.1–26.9) mya. Within the genus *Carya*, the divergence of its 2 species was estimated at 5.4 (3.0–9.8) mya. Species in the Juglandaceae and Myricaceae families (such as *Morella rubra*) diverged approximately 36.6 (32.2–34.5) mya. Additionally, we analyzed gene family expansion and contraction across the related species using CAFÉ (Computational Analysis of gene Family Evolution) to explore the evolution of *J. mandshurica* (Fig. 3C). Among the 24,415 gene families, 798 and 405 expanded and contracted in *J. mandshurica*, respectively, after divergence from *J. cathayensis*. In addition, regarding the gene families of species in the Juglandaceae family, the number of expansions was higher than that of contractions in *J. macrocarpa* and *J. regia*. In species belonging to the Fagaceae family (i.e., *Quercus lobata* and *Castanea mollissima*), more gene families expanded (1,103 and 687) and fewer gene families contracted (533 and 691), respectively. In total, 521 (65.29%) of the 798 expanded gene families displayed rapid evolution in the *J. mandshurica* genome (family-wide *P* < 0.05) and annotated in functions related to metabolic process, cellular processes, cell, cell part, and binding and catalytic activity based on the GO category (Supplementary Fig. S5). We further clustered these genes from rapidly expanded gene families into 88 KEGG pathways. The expanded gene families were primarily involved in photosynthesis (ko00195), ribosome (ko03010), MAPK signaling pathway (ko04010), plant–pathogen interaction (ko04626), and protein processing in the endoplasmic reticulum (ko04141) (Supplementary Table S14). The contracted families were annotated to 20 KEGG pathways, and they mainly participated in the NF-κB signaling pathway (ko04064), immune deficiency and toll signaling pathway (ko04624), toll-like receptor signaling pathway (ko04620), and MAPK signaling pathway (ko04010) (Supplementary Table S15).

### Analyses of genome synteny and whole-genome duplication

**Figure 4:**
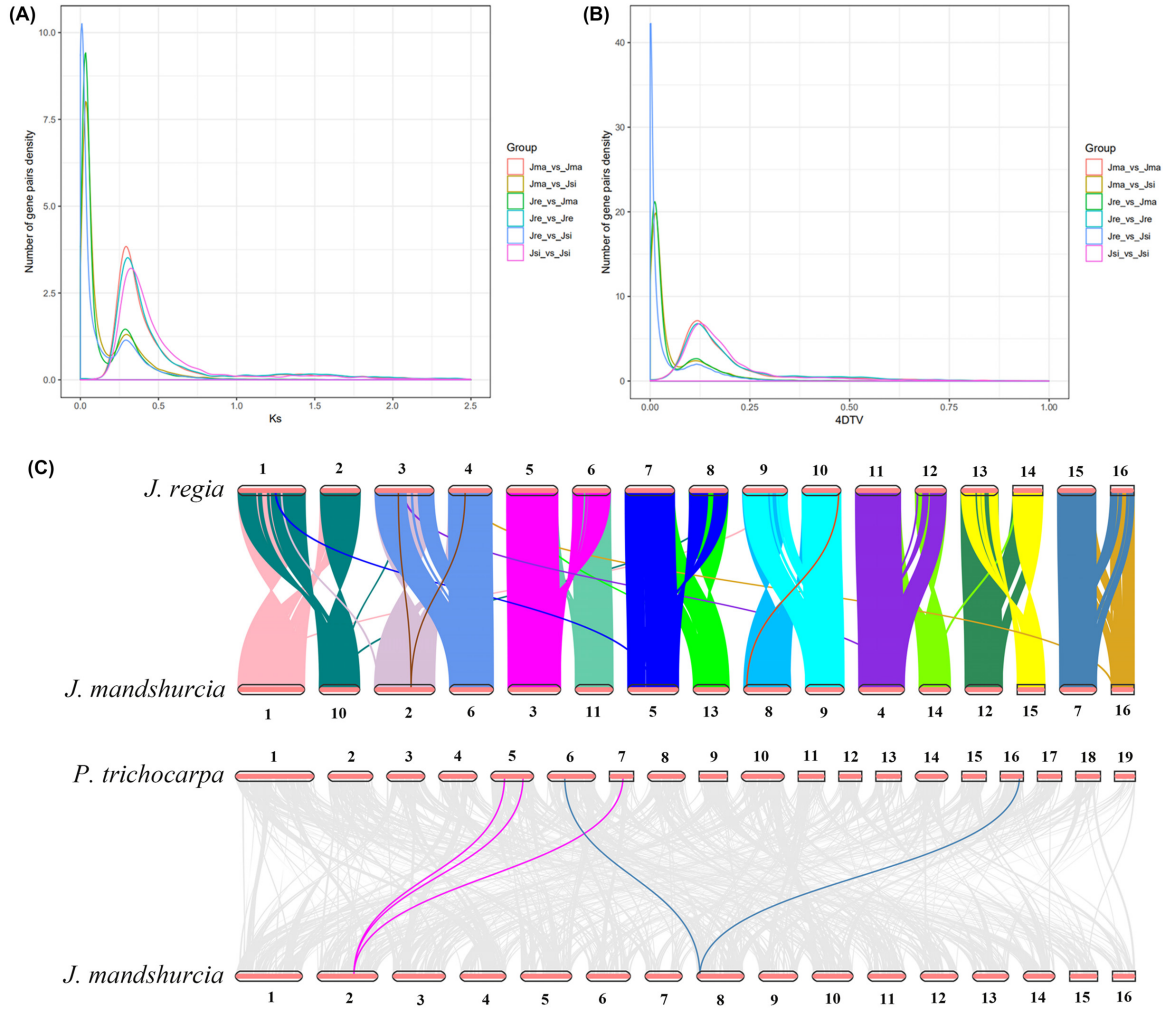
Collinearity and WGD analysis of *Juglans mandshurica* genome. (A) Ks distribution of synthetic orthologs of the selected species (*J. mandshurica, J. regia*, and *J. sigillata*). The x-coordinate is the Ks value, and the y-coordinate represents the number of gene pairs of density. (B) 4dTV analysis. The x-coordinate is the 4DTv value, and the y-coordinate represents the number of gene pairs of density. (C) Schematic representation of syntenic genes among *J. mandshurica, J. regia*, and *P. trichocarpa*. Gray lines in the background indicate collinear blocks of at least 30 genes within the *J. mandshurica* genome and other plants, while the red lines highlight the syntenic gene pairs.

Whole-genome duplication (WGD) occurred in the evolutionary history of most plant species and provided the evolutionary potential for new functions and species diversification [30]. We computed Ks (synonymous substitutions per synonymous site) and 4dTV (4-fold degenerate synonymous sites of the third codons) values among the genes of *J. mandshurica, J. regia*, and *J. sigillata* to analyze gene duplication and divergence. Fossil record showed that the Juglandaceae family appeared in the upper Cretaceous period and radiated in the Paleocene period [31–33]. The Juglandoid WGD must have originated prior to the radiation of Juglandaceae in the Paleocene. Therefore, we selected the Cretaceous–Paleogene boundary (66 mya) as the approximate time of the origin of the Juglandoid WGD. The distribution of these 2 methods was remarkably consistent (Fig. 4A, B). The Ks plot of Jma versus Jma, Jre versus Jre, and Jsi versus Jsi (self-searches within the *J. mandshurica, J. regia*, and *J. sigillata genomes*) reflected the divergence of paralogous genes, originating through the Juglandoid WGD. It showed a significant main peak of approximately 0.3, which was consistent with the results of other similar studies on Juglandoids [22].

Furthermore, we detected synteny between the assembly of the *J. mandshurica* genome and that of *J. regia*. Synteny analysis showed a strong correspondence for all 16 chromosomes in these plants, indicating that the collinearity was maintained at a high level, which suggested the presence of a close evolutionary relationship between 2 species (Fig. 4C and Supplementary Fig. S6). We identified a large number of collinear gene pairs between the chromosomes of *J. mandshurica* (Fig. 2A). We detected linear relationships between *J. mandshurica, J. regia*, and *P. trichocarpa*, and there were significantly distinct syntenic blocks. A total of 49,921 and 38,462 collinear genes were identified between *J. mandshurica* and *J. regia* and between *J. mandshurica* and *P. trichocarpa*, respectively, indicating that 62.5% and 52.3% of the *J. mandshurica* genome was collinear in these plants. To illustrate, Chr2 of *J. mandshurica* shared origins with Chr3 and Chr4 in *J. regia* and with Chr5 and Chr7 in *P. trichocarpa* (Fig. 4C). Therefore, it was evident through the abovementioned results that ancestral collinearity existed between these three species.

### Genomic structural variations between *J. mandshurica* and other 2 walnut species

According to the phylogenetic tree, 7 *Juglans* species were obviously divided into 3 subgroups (Fig. 3). To further understand the genomic differences of walnut species, the structural variation of *J. mandshurica* versus *J. microcarpa* and *J. mandshurica* versus *J. regia* was detected through direct genome comparison. In total, 13,644 translocation events and 347 inversion events were identified between *J. mandshurica* and *J. macrocarpa*, and there were obvious inversion events in chromosome 1 and chromosome 10 in present study (Supplementary Table S16 and Supplementary Fig. S7). Genes affected by the obtained translocation events were enriched in the “fatty acid biosynthesis” (ko00061), “biosynthesis of unsaturated fatty acids” (ko01040), “MAPK signaling pathway” (ko04010), “metabolism of xenobiotics by cytochrome P450” (ko00980), and others, indicating that these translocation events may contribute to the differences in metabolite synthesis, response to the stimulus, and lipid accumulation between *J. mandshurica* and *J. macrocarpa* (Supplementary Table S17). Furthermore, 19,476 translocation events and 309 inversion events were identified between *J. mandshurica* and *J. regia* (Supplementary Table S16 and Supplementary Fig. S7). Genes affected by the identified translocation events were enriched with those related to “stilbenoid, diarylheptanoid and gingerol biosynthesis” (ko00945), “flavonoid biosynthesis” (ko00941), “sesquiterpenoid and triterpenoid biosynthesis” (ko00909), “plant–pathogen interaction” (ko04626), and others, suggesting that these translocation events may be involved in the differences in secondary metabolite synthesis and disease resistance between the *J. mandshurica* and *J. regia* (Supplementary Table S18).

### Gene discovery analysis related to juglone biosynthesis

**Figure 5:**
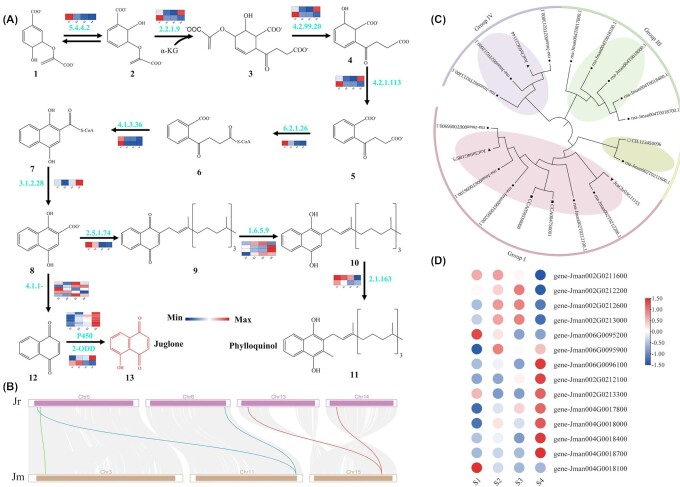
Comparative transcriptomic analysis of genes involved in juglone biosynthesis. (A) Juglone biosynthetic pathway. Numbers under the chemical formulas represent the reaction substrate: 1, chorismite; 2, isochorismate; 3, SEPHCHC; 4, SHCHC; 5, OSB; 6, OSB-CoA; 7, DHNA-CoA; 8, DHNA; 9, demethylphylloquinone; 10, demethylphylloquinol; 11, phylloquinol; 12, 1,4-NQ. Numbers next to arrows represent characterized enzymes or detected enzymatic activities: 5.4.4.2, isochorismate synthase; 2.2.1.9, SEPHCHC synthase; 4.2.99.20, SHCHC synthase; 4.2.1.113, OSB synthase; 6.2.1.26, OSB-CoA ligase; 4.1.3.36, 1,4-dihydroxy-2-naphthoyl-CoA (DHNA-CoA) synthase; 3.1.2.28, DHNA-CoA thioesterase; 2.5.1.74, DHNA phytyl transferase; 1.6.5.9, NDC1; 2.1.1.163, demethylmenaquinone methyltransferase; 4.1.1-, decarboxylase; 2-ODD, 2-oxoglutarate/Fe (II)–dependent dioxygenase; CYP450, cytochrome CYP450. The red chemical formulas represent juglone. (B) Gray lines in the background indicate collinear blocks of at least 30 genes within the *J. mandshurica* genome and *J. regia*. The red lines highlight the syntenic gene pairs related to a decarboxylase (gene-Jman015G0125100), the green lines highlight syntenic gene pairs related to CYP450s (gene-Jman003G0044800), and the blue lines highlight the syntenic gene pairs related to 2-ODD (gene-Jman011G0199700). (C) Lineage-specific expansion of the CYP gene family in *J. mandshurica* and 3 related species. The phylogenetic tree contains 4 subgroups; groups I to IV correspond to the subgroups in the phylogenetic tree. (D) The heatmap represents the expression level of the expanded CYP gene family in *J. mandshurica*. The color scale from blue to red indicates the expression value from low to high.

The high economic value of *J. mandshurica* is attributed to the accumulated juglone in the roots, leaves, bark, and, in particular, the walnut exocarp [9]. As an important quinoid component, juglone's medicinal activity and potential use for sustainable agriculture have been confirmed [9]. However, the biosynthesis, mechanism of action, and regulatory network involved in the juglone pathway require further elucidation; only a few of the relevant genes have been identified via RNA-seq [15]. Juglone biosynthesis in walnut species is partly affiliated with the phylloquinone pathway, wherein the initial substrate is chorismite from the shikimate pathway [15]. These 2 pathways share the DHNA intermediate, and 7 enzymes are used for the DHNA synthesis of phylloquinone. DHNA is subsequently converted to 1,4-NQs by various decarboxylases (Fig. 5A). Juglone is synthesized by 1,4-NQs through hydroxylation facilitated by hydroxylases, CYP450s, and 2-ODD. In the present study, we combined genomic, transcriptomic, and metabolomic technologies to analyze the fruit development process in *J. mandshurica* and identify genes regulating juglone biosynthesis in the exocarp. Among the 146 candidate genes encoding enzymes for juglone biosynthesis identified, 1 encoded isochorismate synthase; 2 encoded 2-succinyl-5-enolpyruvyl-6-hydroxy-3-cyclohexene-2-carboxylate (SEPHCHC), 2-succinyl-6-hydroxy-2,4-cyclohexadiene-2-carboxylate (SHCHC), and o-succinylbenzoate (OSB); 1 encoded OSB–coenzyme A (CoA) ligase; 2 encoded DHNA-CoA; 1 encoded DHNA-CoA thioesterase; 1 encoded DHNA phytyl transferase; 4 encoded NAD(P)H dehydrogenase C1 (NDC1); 2 encoded demethylphylloquinone methyltransferase; 5 encoded decarboxylases; 2 encoded 2-ODDs; and 125 encoded CYP450 (Fig. 5A and Supplementary Table S19). Expression levels of the decarboxylases, *2-ODDs*, and the *CYP450* genes in the S3 and S4 stages were higher than in the S1 and S2 stages; thus, these genes have a potential role in juglone biosynthesis in *J. mandshurica*. Particularly, 16 of 146 key genes were significantly correlated with the juglone (*r* > 0.8 or < −0.8), including 2 genes encoding decarboxylase and 14 genes belonging to the CYP450 family (Supplementary Table S19).

Then we explored the specifically expanded gene families that may be involved in juglone biosynthesis. CYP450s are a class of important oxidative enzymes that are widely distributed in plants and play key roles in the biosynthesis of many natural secondary metabolites. CYP450 enzymes may catalyze various enzymatic steps in the juglone and phytohormone biosynthesis and plant stress responses. CYP450s are typically represented by catalytic reactions involving hydroxylation; furthermore, they catalyze other complex biosynthetic reactions, including epoxidation of aromatic compounds and methyl or amino transfer reactions (transferases). The CYP gene family (14 genes in 4 groups) was identified for the *J. mandshurica* genome, and it appeared to have rapidly expanded in the *J. mandshurica* genome compared with the 3 related species (14 genes in *J. mandshurica* genome, 1 in *C. illinoinensis*, 1 in *C. cathayensis*, 3 in *J. cathayensis*) (Fig. 5B and Supplementary Table S20). The 14 genes in *J. mandshurica* were specifically distributed on chromosomes 2, 4, and 6, and 5 of the 14 genes were in the same group (group Ⅲ) (Fig. 5C). Additionally, the 14 *CYP* genes in *J. mandshurica* were identified in the differentially expressed genes (DEGs) obtained through RNA-seq of the immature exocarp (Fig. 5D). Most *CYP* genes were differentially expressed in the S4 stage. The *CYP* gene family exhibited a particularly rapid expansion, and the inferred increase in transcript abundance may contribute to the juglone accumulation.

### Metabolite profiling and transcriptomics of walnut exocarp development

**Figure 6:**
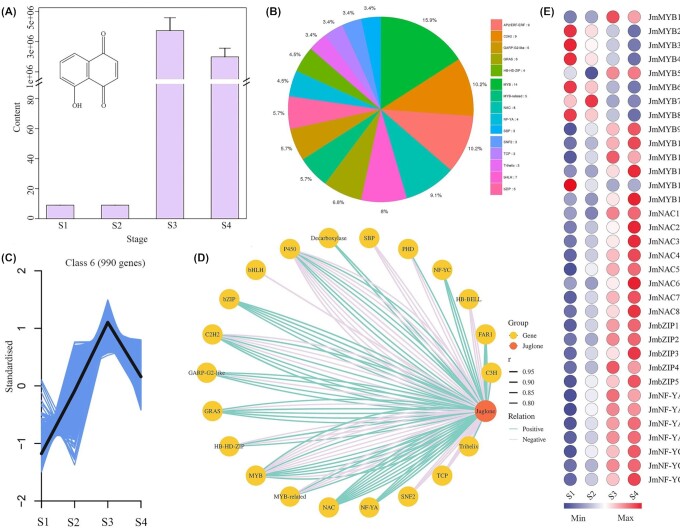
Juglone content and TF regulation during walnut exocarp differentiation. (A) The change of *bona fide* juglone content during walnut exocarp differentiation of *J. mandshurica* (mean ± SD, *n* = 3). The x-axis indicates the different developmental stages (from S1 to S4), and the x-axis represents the juglone content. (B) Frequency distribution of the first top 15 transcriptome factors related to juglone (*r* > 0.8 or < −0.8). (C) Kinetic patterns of coexpressed genes in cluster 6 (990 genes) during walnut exocarp differentiation. (D) Correlation network of juglone genes (2 genes and 19 transcription factors). *r*represents the Pearson correlation coefficient. Line color represents the correlation between genes and juglone (positive and negative correlations) where a positive correlation is a formal representation of an activator and a negative correlation represents a formal representation of an inhibitor. (E) Expression analysis of transcription factors (14 MYBs, 3 FAR1s, 3 NF-YCs, 5 bZIPs, 8 NACs, and 4 NF-YA TFs). The heatmap represents normalized fragments per kilobase of transcript per million fragments. S1 to S4 represent the fruit differentiation stages of *J. mandshurica*.

To further identify the coexpressed genes and regulators in juglone biosynthesis, transcriptomic and metabolomic analyses were performed on the 4 stages of walnut exocarp (Supplementary Fig. S8) and embryo (Supplementary Fig. S9) development. Principal component analysis of the walnut exocarp and embryos revealed that the obvious distinction between the metabolites was from different sample groups, and these metabolites could be used for the next step of metabolomics analysis. A total of 470 secondary metabolic products were detected, mainly containing 7 types of metabolites, including phenolic acids (147, 31.3%), flavonoids (134, 28.5%), tannins (50, 10.6%), alkaloids (47, 10%), lignans and coumarins (28, 6.0%), terpenoids (24, 5.1%), and quinones (21, 4.5%), among others (19, 4.0%) (Supplementary Fig. S10 and Supplementary Table S21). Juglone content increased during the transition from S2 to S3 stages, whereas the juglone content was slightly reduced from S3 to S4 (Fig. 6A). The juglone profiles during the S1 and S2 stages from walnut exocarp were distinct compared with S3 and S4. In total, 195 metabolites were differentially accumulated between S1 and S2 versus S3 and S4. Furthermore, we analyzed the differentially accumulated metabolites between different stages for the juglone component of walnut exocarp. In addition to S1 versus S2 and S3 versus S4, it was evident that 4 of the 6 pairs contain the differentially accumulated juglone (Supplementary Table S22), consistent with the abovementioned results, which show that the juglone content varies during walnut exocarp development in *J. mandshurica*. Additionally, we focused on the DEGs derived from 4 (S1 vs. S3, S1 vs. S4, S2 vs. S3, and S2 vs. S4) paired groups that differentially accumulated juglone, and 897 common DEGs were found in these groups (Supplementary Fig. S11 and Supplementary Table S23). In a combined GO and KEGG enrichment analysis of the 897 DEGs, we observed that the most enriched terms were “extracellular region” and “integral component of membrane” in the GO database and “biosynthesis of secondary metabolites” and “metabolic pathways” in the KEGG pathways (Supplementary Fig. S12 and Supplementary Fig. S13).

TFs are important regulators of plant growth, development, metabolism, and adaptation [34]. We identified some TFs that may be related to juglone biosynthesis of juglone during walnut exocarp development. There were 777, 1,082, 154, and 826 differentially expressed TFs (DEG-TFs) in S1 versus S3, S1 versus S4, S2 versus S3, and S2 versus S4, respectively (Fig. 6B and Supplementary Table S24). In total, 62 MYB, 55 AP2/ERF-ERF, 50 NAC, 48 bHLH, 45 C2H2, 36 WRKY, and 26 bZIP TFs were found in S1 versus S3, and 82 MYB, 73 C2H2, 72 bHLH, 71 AP2/ERF-ERF, 64 NAC, and 60 WRKY TFs were identified in S1 versus S4; these TFs may be involved in juglone biosynthesis. In addition, we observed that the TFs essential for juglone biosynthesis were commonly identified in the 4 groups (i.e., AP2/ERF-ERF, bHLH, bZIP, C2H2, MYB, and NAC). Expression analysis indicated that most TFs showed higher expression in S3 and S4 compared with S1 and S2, which was consistent with the results of metabolites, suggesting the presence of a strong association between these TFs and juglone accumulation during walnut exocarp differentiation.

To identify genes that displayed similar abundance patterns as juglone content, coexpression cluster analysis was performed using K-means methods based on the transcript per million fragment values. All genes were primarily clustered into 10 clusters with distinct expressions, in which cluster 6 (990 genes) showed variation similar to that of the juglone content during walnut exocarp differentiation. This suggested that these genes were crucial to fully elucidate juglone biosynthesis at the transcript level (Fig. 6C). GO enrichment analysis showed that 990 DEGs were enriched in “intracellular,” “intracellular part,” and “RNA metabolic process” (Supplementary Fig. S14). The top enriched KEGG DEGs were “purine metabolism,” “RNA polymerase,” and “spliceosome” (Supplementary Fig. S15).

To more deeply explore the relationship between gene expression and juglone, data obtained from metabolites (juglone) and genes (including CYP450s and MYB, NAC, and bZIP TFs, among others) were employed to construct a gene-to-metabolite correlation network in the walnut exocarp. A total of 1,860 DEGs correlated with juglone content, and the Pearson correlation coefficient was set at *r* > 0.8 or < −0.8 as the cutoff (Supplementary Table S25). The core conserved DEGs (566) in the 4 paired groups (S1 vs. S3, S1 vs. S4, S2 vs. S3, and S2 vs. S4) were mainly enriched in “biosynthesis of secondary metabolites,” “alanine, aspartate, and glutamate metabolism,” and “nitrogen metabolism” in KEGG terms (Supplementary Fig. S16 and Fig. S17). Among the 1,860 DEGs, 155 were identified as TFs, and the top 15 TFs (see the pie chart) consist of MYB, C2H2, AP2/ERF-ERF, bHLH, NAC, and bZIP TFs, which indicates the presence of TF regulation for juglone during walnut exocarp differentiation (Fig. 6B). Among the 14 *CYP450* genes, 5 genes that correlated with juglone (*r* > 0.8 or < −0.8) showed a strong positive correlation with the network, and 2 genes encoding decarboxylases showed the same relationship, thereby suggesting that these genes may be involved in juglone biosynthesis (Fig. 6D and Supplementary Table S26). Among the 155 TFs, all the bZIP (5), NAC (8), NF-YA (4), NF-YC (3), and FAR1 (3) TFs were strongly correlated with juglone, and the majority of these TFs were highly expressed in the S3 and S4 stages, suggesting regulation functions for juglone (Fig. 6E). Therefore, these TFs could considerably influence the gene-to-juglone regulatory network. The 5 core *CYP450* genes and several key TFs could be suitable candidate genes and help provide new insights into natural juglone biosynthesis.

### Gene discovery for lipid biosynthesis and oil body formation

*J. mandshurica* is an important oil plant species, and its oil-rich (>60%) embryos have a variety of different oils such as oleic, linoleic, and unsaturated fatty acids, among others. The oil has important medicinal, nutritional, and industrial value. Although previous studies have found multiple oil components and confirmed their activity as antiviral, antimicrobial, anticancer, or antihelminth agents [35, 36], the molecular regulatory mechanisms for lipid biosynthesis and oil body formation in *J. mandshurica* require further elucidation [37–39]. Oil formation in plant seeds occurs through the lipid biosynthesis pathway, where the fatty acids are synthesized in the plastids, and triacylglycerol (TAG) synthesis occurs in the endoplasmic reticulum. TAGs are stored in an oil body after synthesis and degraded to provide carbon and energy during seed germination and early seedling growth [40].

In the present study, we investigated the regulation of genes involved in lipid biosynthesis and oil body formation. We used a combination of transcriptome sequencing and metabolomics on 4 different stages of differentiation in *J. mandshurica* walnut embryos. Among the 450 metabolites identified in *J. mandshurica* samples, there were 99 lipids (22%), 91 amino acids and their derivatives (20.2%), 77 organic acids (17.1%), 61 phenolic acids (13.6%), 51 nucleotides and their derivatives (11.33%), and 71 other compounds (15.78%) (Supplementary Fig. S18 and Supplementary Table S27). Among these metabolites, lipids were predominant and primarily comprised 41 free fatty acids (41.4%), 28 lysophatidylcholines (28.3%), 14 lysophatidylethanolamines (14.1%), 13 glycerol esters (13.1%), 2 sphingolipids (2.02%), and 1 phosphatidylcholine (1.01%) (Supplementary Fig. S19 and Supplementary Table S28), thereby indicating that the walnut embryos are rich in free fatty acids during differentiation. Among these 41 free fatty acids, 11 had the highest content from S1 to S4 (Supplementary Fig. S20), including linoleic acid (C18:3), stearic acid (C18:0), arachidic acid (20:0), α-linolenic acid, and γ-linolenic acid, suggesting the presence of abundant oils in the *J. mandshurica* walnut embryos. The top enriched KEGG terms of these compared groups mainly were “metabolic pathway,” “biosynthesis of secondary metabolites,” “biosynthesis of amino acids,” and “ATP-binding cassette transporters” (Supplementary Fig. S21). These oils were enriched during walnut differentiation and provided key information on lipid biosynthesis in *J. mandshurica*.

**Figure 7:**
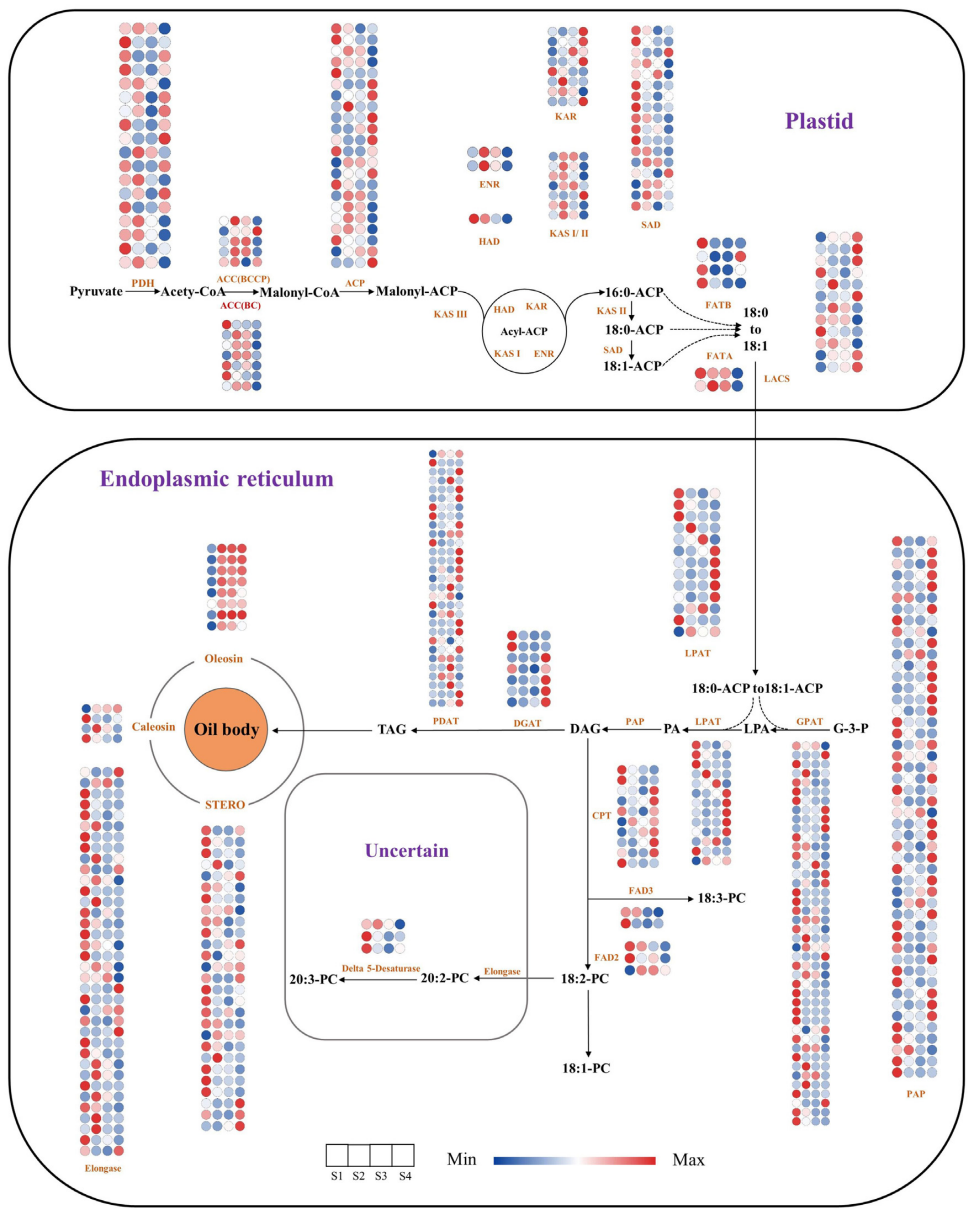
Comparative transcript analysis of genes involved in lipid biosynthesis of developing fruit. ACC (BC), biotin carboxylase subunit of heteromeric acetyl-CoA carboxylase (ACCase); ACC (BCCP), biotin carboxyl carrier protein of heteromeric ACCase; ACP, acyl carrier protein; CPT, diacylglycerol cholinephosphotransferase; DAG, 1,2-diacylglycerol; DGAT, diacylglycerol acyltransferase; FAD2, v-6 desaturase; FAD3, v-3 desaturase; FATA, acyl-ACP thioesterase A; FATB, acyl-ACP thioesterase B; G-3-P, glycerol-3-P; GPAT, glycerol-3-phosphate acyltransferase; KAS, ketoacyl-ACP synthase; LACS, long-chain acyl-CoA synthetase; LPA, 1-acylglycerol-3P; LPAT, 1ysophosphatidic acid acyltransferase; PA, 1,2-diacylglycerol-3P; PAP, phosphatidic acid phosphatase; PDAT, phospholipid: diacylglycerol acyltransferase; PDH, pyruvate dehydrogenase; SAD, stearoyl-ACP desaturase; TAG, triacylglycerol. The color scale from blue to red indicates the expression value from low to high.

A transcriptome survey carried out to better understand the mechanisms underlying lipid biosynthesis and oil body formation facilitated the identification of 346 genes related to lipid synthesis. These genes included 105 for fatty acid biosynthesis, 202 for TAG biosynthesis, and 39 for oil body formation (Fig. 7). Expression levels of most of the structural genes (*PDH* and *ACC*[*BC*]) at the S2 stage were higher than the expression at other stages, whereas the majority of the other genes were significantly expressed in the S4 stage. This could partially explain the persistence of the high level of free fatty acid content in the S2 stage (Supplementary Fig. S22). After their formation, the free fatty acids were activated via long-chain acyl-CoA synthetases (LACS) to generate acyl-CoA derivatives, which were transported out of the plastid (Fig. 7). The transcript level of *LACS* at S4 was higher than in other stages, which contributed to TAG synthesis [41]. Glycerol-3-phosphate acyltransferase catalyzed the glycerol-3-phosphate and acyl-CoA to form lysophosphatidic acid (LPA), and then LPA was then converted to phosphatidic acid (PA) with the help of lysophosphatidic acid acyltransferase (LPAT). Subsequently, phosphatidic acid phosphatase (PAP) converted PA to diacylglycerol (DAG). TAGs were finally synthesized through the reaction of DAG via diacylglycerol acyltransferase (DGAT) or diacylglycerol acyltransferase [42]. Most of the differentially expressed *PAP* genes were expressed at high levels in S4, thereby indicating a dominant role for TAG formation. The expression of *DGAT* and *PAP* genes appeared similar to *PAP* genes. Oil body formation involves the binding of TAGs, which are bound to several proteins, including oleosionoleosin, caleosin, and steroleosin [43]. Oleosin is an oil protein that could increase charge repulsion, allowing oil bodies to be independent of each other. Oleosin maintains high expression in S2, S3, and S4 in the walnut embryos; this suggests that genes encoding these proteins are good candidates for genes regulating oil body formation.

Several TFs regulate plant growth and development. Among the 15,152 DEGs, 1,876 were identified in *J. mandshurica*, representing 12.38% of the DEGs involved in the walnut embryo differentiation (Supplementary Table S29). The top 5 transcription factor families were 110 MYBs, 109 C2H2s, 105 AP2/ERF-ERFs, 102 bHLHs, and 76 NACs, many of which may be related to walnut embryo development and differentiation. Some TFs (ABI3, LEC1, LEC2, FUS3, and WRI1) are considered key regulators of lipid biosynthesis and oil accumulation in many plant species. In the transcriptome analysis, 1 FUS3, 2 ABI3s, 2 LEC2s, and 4 WRI1s were identified, whereas LEC1 TFs were not detected, suggesting that the lipid synthesis was not regulated by these TFs in mature walnut embryos (S4) (Supplementary Table S30). ABI3 is involved in seed development and dormancy, particularly in fatty acid metabolism [44, 45]. Four ABI3 TFs showed high expression in the S4 stage consistent with the oil accumulation pattern, which suggested a role in the regulation of lipid biosynthesis in mature walnut embryos. WRIl and FUS3 were expressed at a high level during the S2 and S3 stages; this suggested a minor role in lipid biosynthesis and oil accumulation. To further investigate the correlation between the identified genes and lipid biosynthesis, an analysis was performed using Pearson's correlation. One ABI3 transcription factor was highly correlated with the eicosadienoic acid (C20:2) (*r* = −0.839). Furthermore, we identified 1 ABI3 gene that was negatively correlated with several glycerides (*r* = −0.828), lysophatidylethanolamine (*r* = −0.844), and lysophatidylcholine (*r* = −0.807), suggesting that an ABI3 interacting protein is an inhibitor or plays a different negative role in lipid biosynthesis.

## Discussion

Third-generation sequencing is a powerful technology, which that will help accelerate genetic improvement for many crop species [46]. Whole-genome sequencing has been performed for many plants, and it has provided useful genomic information for functional gene mining, genetic linkage map construction, quantitative trait analysis, and molecular breeding [47, 48]. Manchurian walnut is widely distributed in northeast China and utilized for its edible kernels, superior wood characteristics, and medicinal value of its secondary products. Research on this species has been limited, and genome-level studies are lacking. This study provides a high-quality and chromosome-level reference genome sequence for molecular breeding and evolutionary studies in the Juglandaceae. We used long (PacBio) HiFi reads, next-generation sequencing, and Hi-C scaffolding to sequence and assemble the genome of *J. mandshurica*, thereby providing new insights and valuable genetic information on juglone and lipid biosynthesis.

In the present study, we report the highest-quality genome assembly for *J. mandshurica* to date, with the longest contig N50 of 21 Mb and the highest genome completeness of 98.3% in terms of BUSCO results. Compared with previous studies on genome assemblies for this species, our contig N50 size (21 Mb) for *J. mandshurica* was improved by 187-fold (N50 was 0.1 Mb) [49] and 3-fold (N50 was 6 Mb) [50]. Our complete BUSCO score was 1,588, which was considerably higher than that of a recent genome assembly (1,375) [50]. After sequencing and assembly, our assembled genome was 548.7 Mb, which was slightly higher than that reported by Yan et al. [50] (548.5 Mb), but lower than that of Bai et al. [49] (580 Mb). We obtained 40,453 protein-coding genes, which was substantially higher than those obtained by Yan et al. (29,032 protein-coding genes). Using the sequence platform of WGS-PacBio Sequel II, we obtained 14.62 Gb of genome sequence; however, the previous genome sequence was 101 Gb in size and was obtained using the Nanopore sequencing platform. The GC content and repeated sequences were 36.72% and 0.33 Gb (60.99%), which were relatively lower than the repeats reported by Yan et al. (38.5% GC content and 0.34 Gb [62.08%] repeat sequences). Our chromosome-scale genome maintained a relatively high assembly quality, thereby providing valuable information for further analysis of evolution and mining of functional genes in *J. mandshurica*.

Currently, 9 species in the Juglandaceae family have been sequenced for whole-genome information, consequently yielding a mass of high-throughput sequence data and contributing to understanding the evolution of Juglandaceae species [51]. Based on 558 single-copy orthologs from 15 plant genomes (9 of which were from the Juglandaceae family), genome-level studies were performed to update the evolutionary relationships between *J. mandshurica* and its related species. The Juglandaceae species maintain close genomic relationships, and as observed in the present study, all of the Juglandaceae species group into the same cluster, consistent with the morphology-based plant taxonomy [51]. For the relationship between *J. mandshurica* and *J. cathayensis*, there is still controversy at present. In the flora of China, *J. mandshurica* has been considered a synonym of *J. cathayensis* according to the phenotypic characteristic, but there is no definitive molecular evidence at the genome level. Here, although the phylogenomic analysis showed that *J. mandshurica* was closest to *J. cathayensis*, they possess a relatively larger divergence time (approximately 13.8 mya), and similar results were found in a previous study [52]. Also, the synteny analysis and genome-wide alignment between *J. mandshurica* and *J. cathayensis* in present study were performed by jcvi [53] and Last [54] software, respectively (Supplementary Fig. S23 and Table 2). The results showed that the number of protein-coding genes in *J. mandshurica* (40,453) was similar to that in *J. cathayensis* (39,905). However, the synteny analysis found that approximately 60% of the *J. mandshurica* genome was collinear with *J. cathayensis*. Additionally, the alignment analysis suggested that the obtained identity genes (identity ≥90%) accounted for ∼66% of genome. Thus, we preliminarily speculated that they were eventually established as 2 separate species. WGDs offer the evolutionary potential to generate new functions in plant species [55]. In particular, in a previous study, the Ks distribution results showed that the main peak of Ks was nearly 0.3, and the Juglandales appeared from the Cretaceous to Paleocene at the periods (56–66 mya) according to the fossil evidence, and there was a Juglandoid WGD for walnut species [56, 57]. In the present study, for comparative genomics, the main peak was also approximately 0.3. Therefore, we indicated that there was a Juglandoid WGD that originated before the radiation of Juglandaceae in the Paleocene, and similar results were also found in a recently reported *J. mandshurica* assembly [50]. In the collinearity comparison with *P. trichocarpa*, many syntenic blocks were identified, which indicated the presence of a close genetic relationship between *J. mandshurica* and *J. regia* and a more distant relationship with *P. trichocarpa*.

**Table 2:** The genome comparison of *J. mandshurica* and *J. cathayensis*

Item	*Juglans mandshurica*	*Juglans cathayensis*
Total genome size (bp)	548,694,591	493,089,748
Scaffold N50 (bp)	35,382,463	31,976,116
Contig N50 (bp)	21,388,210	23,436
Gene number	40,453	39,905
Synteny genes (by jcvi)	25,402	24,016
Gene identity (≥90%) (by last)	26,782	28,067
Gene identity (≥80%) (by last)	31,008	31,655
Gene identity (≥70%) (by last)	33,881	33,927

The Juglandaceae family is an economically important species that has been planted and domesticated since the Han dynasty (206 BC–220 AD). Fruits of many species in this family are rich in medicinal components in the walnut exocarp and the oils in the kernel, particularly for species in the *Carya* and *Juglans* genera [58, 59]. The regulatory mechanism of fruit development/differentiation and the biosynthesis of juglone and lipids in *J. mandshurica* remain largely unknown. Previous studies identified a small number of genes affecting the bioactivity of juglone in walnut roots and leaves, but without knowledge of the fact that these genes were related to juglone accumulation in fruits, particularly in the walnut exocarp [14, 15]. Therefore, it is necessary to further investigate the potential genes involved in controlling juglone biosynthesis during fruit development. Reportedly, genes involved in juglone biosynthesis are partly derived from the phylloquinone pathway. In this pathway, DHNA plays a key role as an intermediate for regulating juglone and phylloquinone biosynthesis. In the present study, for walnut exocarp differentiation, we combined genomic, transcriptomic, and metabolomic data to construct a gene–metabolite network, thereby identifying genes and TFs that may encode decarboxylases, *2-ODDs*, and *CYP450s* involved in juglone biosynthesis. This consequently helped to fully elucidate the blanks of mechanism underlying the juglone accumulation in fruits and indicated a new step to identify the juglone pathway genes in all tissues. In particular, 146 key genes were identified in juglone biosynthesis from the DEGs in our *J. mandshurica* transcriptome, 125 of which were *CYP450* genes. Therefore, CYP450 superfamily members play a crucial role in juglone accumulation and could catalyze the reactions from 1,4-NQ to form juglone; these results were similar to those reported in a previous study [14]. Expression analysis showed that the obtained *CYP450* genes maintain a high expression level during the differentiation of walnut exocarp, which would contribute to juglone accumulation. This result is similar to the secondary metabolite biosynthesis in plants such as *Aconitum vilmorinianum* (aconite) [60], *Salvia miltiorrhiza* (red sage) [61], *Scutellaria baicalensis* (Chinese skullcap) [62], and *Aralia elata* (angelica tree) [63]. Additionally, we identified the expanded CYP450 gene family from the genome assembly of the *J. mandshurica*, suggesting that they may specifically affect juglone biosynthesis. Among the 14 *CYP450* genes, 5 were correlated with juglone (*r* > 0.8 or < −0.8) based on the coexpression network analysis and can be considered candidate genes for further study on juglone biosynthesis. Although some genes in the juglone biosynthetic pathway have been identified, the regulators remain unknown. In a previous study, the expression of TFs, including AP2/ERF, NAC, HSF, WRKY, MYB, C2H2, and GRAS, was changed significantly under juglone treatment [64]. In this study, the common TFs, including AP2/ERF-ERF, bHLH, bZIP, C2H2, MYB, and NAC, were identified in 4 comparative groups during developmental walnut exocarp. In particular, all the potential TFs, including bZip, NAC, NF-YA, and NF-YC, were positively correlated with the abundance of juglone and might have participated in juglone biosynthesis, combining comparative transcriptomics and metabolic profiling. Additionally, we found that 1 NAC (*gene-Jman002G0279700, r* = 0.957) and 1 bZip (*gene-Jman003G0271600, r* = 0.952) TF showed relatively high correlation with juglone, and they could be used as key candidate regulators to identify the target genes in juglone pathways. These results may be valuable for further studies on juglone biosynthesis and its agricultural uses.

In addition to the study of the walnut exocarp, we used the developing/differentiating kernel to identify the genes associated with oil accumulation in *J. mandshurica*. Lipids were the dominant secondary metabolites, and free fatty acids were enriched in *J. mandshurica* embryos. Some free fatty acids (linoleic, stearic, arachidic, α-linolenic, and γ-linolenic acids) were the most abundant during the S1 to S4 stages; these results were similar to those reported in a previous study [37]. LACS is one of the key enzymes in fatty acid metabolism, which can catalyze acyl-CoA synthesis and contribute to TAG assembly. In *Helianthus annuus, HaLACS1* overexpression can effectively increase seed oil content [65]. Expression analysis of *BnLACSs* genes showed that they were involved in fatty acid biosynthesis in *Brassica napus* [66]. Therefore, the *LACS* gene expression level was closely related to seed oil synthesis. In this study, the corresponding genes (*PAP, LACS*, and *DGAT*) involved in lipid biosynthesis also showed high abundance during embryo development, suggesting that these genes and metabolites play key roles in lipid synthesis for oil accumulation. TAG is mainly stored in the cytoplasm in the form of oil bodies after synthesis, and oleosin is the main protein regulating the structure and function of oil bodies. Reportedly, 3 oleosin genes in sesame oil were confirmed to be transcribed in mature seeds, thereby maintaining the structural stability of the oil body [67]. In *Lilium longiflorum*, oil bodies in pollen were mainly protected by special oleosin proteins [68]. Additionally, in the plant kernels with a relatively high oleosin content, the oil volume was relatively smaller [69]. In the present study, expression patterns of the 8 oleosin genes obtained were nearly consistent and showed high expression levels during the embryos' late developmental stage. Therefore, we speculated that these oleosin proteins played an important role in maintaining oil body stability in *J. mandshurica*. TFs are important regulators for plant growth and development. In addition, some TFs (MYB, C2H2, AP2/ERF-ERF, and NAC) were differentially expressed during embryo development. Such regulation of TFs implies that they may play important roles in lipid biosynthesis in *J. mandshurica*, similar to results found in previous studies [70, 71]. For lipid synthesis, several TFs regulate the structural genes and their accumulation, including some positive (ABI3, FUS3, LEC2, AGL15, and WRI1) and negative (MYB76, MYB118, MYB89, GL2, and WRKY6) regulatory factors [72]. Among them, ABI3, FUS3, and WRI1 have been identified in many plants and can regulate the expression of functional genes involved in fatty acid synthesis, thereby regulating seed oil accumulation. Transcriptome analysis of walnut kernel development found that *WRI1* was involved in lipid biosynthesis and polyunsaturated fatty acid metabolism [73]. In *Torreya grandis, TgWRI1* and *TgFUS3* are involved in the regulation of genes related to lipid biosynthesis during seed development [74]. Herein, the authors identified some of the TFs involved in lipid biosynthesis. These TFs (FUS3, ABI3, LEC2, and WRI1) were correlated with lipid metabolites in *J. mandshurica*, similar to a previous study [75–77]. These results established the foundation for identification to help identify genes encoding enzymes that catalyze the formation of fatty acid and oil body formation; thus, these results will be valuable in the future for studies involved in engineering of lipid biosynthesis.

## Methods

### Plant materials and DNA sequencing

Fresh leaves of adult *J. mandshurica* were collected at the campus of Northeast Forestry University (126°37′′57.28′′ E, 45°43′′6.53′′ N), in Harbin, Heilongjiang province, China. High-quality genomic DNA from fresh leaves was extracted by an improved CTAB method [78]. For long-read DNA sequencing, 15 μg sheared DNA was used for circular consensus sequencing (CCS, RRID:SCR_021174). The SMRT Bell HiFi libraries were constructed as follows: (i) DNA was subjected to enzymatic reaction to remove prominent single-stranded ends, and enzymatic reaction was carried out to remove the prominent single-chain end and repair the DNA damage; (ii) after that, A was added at the end of the double chain to repair the DNA terminal; (iii) the T-overhang of the SMRT bell adapter was ligated with the end at 20°C for 15 hours, and the library was purified with 1× AMPure PB after the connection was completed; (iv) determination of concentration and fragment size distribution of samples in library were completed by FEMTO Pulse automatic pulse field capillary electrophoresis and Qubit 3.0 fluorescence detector (Life Technologies, Carlsbad, CA, USA), the BluePippin system was used to select fragment size, the DNA was randomly cut into ∼15-Kb fragments, and the obtained libraries were purified using 1× AMPure PB; (v) the size and quality of the library were evaluated using FEMTO Pulse and Qubit dsDNA HS detection kits; (vi) the sequencing primers and Sequel II DNA polymerase were annealed respectively, and they were used for combing with the final SMRT bell library; and (vii) after library construction, sequencing was performed on the PacBio Sequel II platform  (PacBio Sequel II System, RRID:SCR_017990) (14.62 Gb data, 26-fold coverage of the genome) at Frasergen Bioinformatics (Wuhan, China) at a concentration of 120 pM, and the running time was 30 hours. For short-read DNA sequencing, libraries were constructed from 300- to 500-bp fragments and sequenced using the WGS Illumina HiSeq 2500 platform (Illumina HiSeq 2500 System, San Diego, California, USA, RRID:SCR_016383). In total, 56 Gb of raw data were obtained.

### Genome k-mer analysis

The quality filtered long reads were used for genome size estimation. In present study, we calculated the frequency of each 17-mer from the HiFi sequencing reads (14.62 Gb) and examined the distribution of the 17-mer numbers. Then, we estimated the genome size to be about 547.99 Mb, and the proportion of repeat sequences and heterozygosity rate of the genome were determined to be approximately 48.78% and 0.77%, respectively, using GCE software v1.0.2 (GCE, RRID:SCR_017332) [79].

### RNA extract and Iso-seq sequencing

To obtain high-quality annotation results, Iso-seq sequencing was implemented in this study. The bark fruit and leaves of *J. mandshurica* were collected, frozen in liquid nitrogen immediately, and stored in a refrigerator at −80°C for Iso-seq sequencing. The total RNA was obtained by the TRIzol reagent (Invitrogen, Carlsbad, CA, USA). RNA purity was checked using the kaiaoK5500® Spectrophotometer (Kaiao, Beijing, China). RNA integrity and concentration were assessed using the RNA Nano 6000 Assay Kit of the Bioanalyzer 2100 system (Agilent 2100 Bioanalyzer Instrument, RRID:SCR_019389; Agilent Technologies, Santa Clara, CA, USA). First, full-length cDNA of mRNA was synthesized by the Clonetech (Beijing, China) SMARTer PCR cDNA Synthesis Kit. The obtained full-length cDNA was amplified by polymerase chain reaction (PCR), and the amplified product was purified by PB magnetic beads, and some small fragment cDNAs below 1 kb were further removed. The end-repair of products was implemented, and the adapter with SMRT dumbbell was added to the end of cDNA. The unconnected fragments were digested by exonuclease, and the fragments were purified by PB magnetic beads to obtain the sequencing library. Qubit 3.0 was used for accurate quantification, and the Agilent 2100 was used for library size detection to obtain the high-quality library. After that, the samples were sequenced on the PacBio Sequel II platform, and 53 Gb raw data were obtained.

### Genome assembly

Here, we obtained approximately 14.62 Gb (26× of the genome) sequencing data on the PacBio Sequel II platform. The raw data obtained in the PacBio Sequel II CCS sequencing mode were converted to HiFi data by CCS software using the parameters “-minPasses 3.” Then, these HiFi data (∼15-kb long reads) were further assembled using Hifiasm v.0.2.0  (Hifiasm, RRID:SCR_021069) [16] software with default parameters to get a preliminary assembly genome. Additionally, the gfatools [80] was employed to obtain the sequence graphs with the FASTA format. The Hi-C sequencing was performed on an Illumina HiSeq platform with PE 150 bp and yielded 51 Gb of sequence. Throughout the construction of the Hi-C library and sequencing, we used HTQC v1.92.310 (HTQC, RRID:SCR_006448) [81] software to perform quality control on the raw data and obtained clean data. For clean data, BWA (BWA, RRID:SCR_010910) [82] software (0.7.17) was used for comparison to different contigs and then used for Hi-C associated scaffolding. First, the low-quality reads with self-ligation and nonligation were removed and filtered. In total, 657 contigs were successfully classified into 16 chromosome groups using the agglomerative hierarchical clustering method in Lachesis software (LACHESIS, RRID:SCR_017644) [17], and the clustered contigs were further ordered and oriented. The final reference assembly contained 16 chromosome-scale pseudomolecules, with maximum and minimum lengths of 52 Mb and 19 Mb, respectively. A heatmap of the interaction matrix of all pseudochromosomes was plotted with a resolution of 500 kb. The assembled chromosome number is the same as the haploid chromosome number of *J. mandshurica* (*n* = 16) (Fig. 1A).

### Evaluation of assembly results

After assembly was completed, we evaluated the results using 3 methods. First, the comparison tool minimap2 v2.5 (Minimap2, RRID:SCR_018550) [83] was used to compare the assembled genomes, and the comparison rate of reads, the extent of genome coverage, and the distribution of depth were calculated to evaluate the integrity of the assembly and the uniformity of sequencing coverage. Second, the Burrows–Wheeler Aligner (BWA) [82] was used to compare reads to the reference genome. Finally, based on the single-copy homologous gene set in OrthoDB (OrthoDB, RRID:SCR_011980) [84], BUSCO v5.2.2 (BUSCO, RRID:SCR_015008) [85] was used to predict these genes and calculate their integrity, fragmentation, and possible loss rates. BUSCO assessment indicated that 98.3% of the complete genes were captured.

### Genome annotation

We used homologous and *de novo* annotation to identify repetitive sequences. First, RepeatMasker (Open-4.0.9) (RepeatMasker, RRID:SCR_012954) [86] and RepeatProteinMask (Open-4.09) [86] were used to search for TE sequences from Repbase (release 21.01) (Repbase, RRID:SCR_021169) [87] based on homology. Second, RepeatModeler (Open-1.0.11) (RepeatModeler, RRID:SCR_015027) [88] and LTR-Finder v1.0.7 [89] were used to construct a repeat sequence database, and then RepeatMasker (Open-4.09) [86] was used to identify the repetitive sequences. TRF [90] was used to identify tandem repeat sequences. Finally, the results based on homologous annotation and *de novo* annotation were integrated, and the nonredundant elements after overlapping were removed for the final repeated sequence annotation.

We used homologous, *de novo*, and transcriptome-assisted annotation to predict the structure and function of coding genes. For homologous annotation, 3 to 5 related species were selected, and then TblastN (TBLASTN, RRID:SCR_011822) [91] (E cutoff of 1e^–5^) was used to compare the related species to the reference genome. Then, the aligned sequences and their corresponding proteins were filtered and transmitted to the Exonerate [92] for accurate alignment. Augustus (v3.3.1) [93] and GlimmerHMM v3.0.4 (GlimmerHMM, RRID:SCR_002654) [94] were used for *de novo* annotation. For Iso-seq data, we used Gmap (GMAP, RRID:SCR_008992) [95] to align it to the reference genome and then used TransDecoder (TransDecoder, RRID:SCR_017647) [96] to predict open reading frames in the transcripts to define putative coding sequences. Maker v3.00 (MAKER, RRID:SCR_005309) [19] was used to integrate the predicted gene sets into a nonredundant, more complete, and reliable gene set. Finally, the proteins in the gene collection were annotated by means of curated protein databases, including SwissProt, TrEMBL, KEGG, GO, and NR, using NCBI BLASTP v2.6.0+  (BLASTP, RRID:SCR_001010) (*E*value ≤1e^–5^) [97].

In the annotation process of noncoding RNA, according to the structural characteristics of tRNA, tRNA sequences in the genome were searched using tRNAscan-SE v1.3.1 (tRNAscan-SE, RRID:SCR_010835) [98]. Because rRNA is highly conserved, rRNA sequences from related species can be selected as reference sequences to search for rRNA by BLASTN v2.6.0 (BLASTN, RRID:SCR_001598) [97] alignment. Infernal of Rfam (Rfam, RRID:SCR_007891) [99] was used to predict miRNA and snRNA sequences in the genome.

### Phylogenomic reconstruction and gene family evolution

To identify the gene families in each species, we clustered the proteins of 15 species through the OrthoMCL (V14-137) [28] process based on sequence similarity with the parameter of “-inflation 1.5.,” including 13 rosid species (e.g., *J. cathayensis* [100], *J. macrocarpa* [100], *J. nigra* [100], *J. regia* [100], *J. hindsii* [100], *J. sigillata* [100] *C. cathayensis* [101], *C. illinoinensis* [101], *Quercus lobata* (valley oak) [102], *Castanea mollissima* (Chinese chestnut) [103], *Morella rubra* (red bayberry) [104], *P. trichocarpa* [105], and *V. vinifera* [106]) and 1 commelinid species (*O. sativa*) [107]. Muscle v3.8.31 (MUSCLE, RRID:SCR_011812) [108] was used to conduct multiple sequence alignments of genes within the single-copy homologous gene family of each species, and RAxML v8.2.12 (RAxML, RRID:SCR_006086) [29] was used to construct an evolutionary tree using maximum likelihood.

We utilized the constructed evolutionary tree, along with the TimeTree (TimeTree, RRID:SCR_021162) website and studies in the literature, to obtain time correction points, using the R8S v1.71 (r8s, RRID:SCR_021161) [109] and mcmctree v4.9e in the PAML software (PAML, RRID:SCR_014932) [110], and the bifurcation time was estimated with 5 corrected divergence time points from the TimeTree website as follows: *O. sativa* versus *J. mandshurica* (115–308 mya), *V. vinifera* versus *J. mandshurica* (107–135 mya), *P. trichocarpa* versus *J. mandshurica* (101–131 mya), *Q. lobata* versus *C. mollissima* (6–49 mya), *Q. lobata* versus *J. mandshurica* (51–87 mya), and 2 corrected divergence time points from the*M. rubra* genome article [55], *M. rubra* versus *Juglans* genus (28–34 mya), and the *Carya* genome article [101], genus *Juglans* versus genus *Carya* (∼23 mya). CAFÉ (CAFE, RRID:SCR_005983) [111] was used to simulate the expansion and contraction events of gene families in each lineage of the evolutionary tree.

### Analyses of genome synteny and WGD

*J. regia* and *P. trichocarpa* genomes were selected as comparisons for collinearity analysis with the *J. mandshurica* genome. MCscan (MCScan, RRID:SCR_017650) [53] was used to perform synteny searches, with at least 30 gene pairs required in each syntenic block. TBtools [112] was subsequently used to visualize the schematic diagram. Mummer v4.0.0beta2 (MUMmer, RRID:SCR_018171) [113] was used to estimate the collinearity between the genomes of *J. regia* and *J. mandshurica*.

We used MCscan (MCScan, RRID:SCR_017650) to search for collinear regions in species genomes and calculated the 4dTV of gene pairs contained in the collinear regions to reflect the relative differentiation events and whole-genome duplication in the evolutionary history of *J. mandshurica*. We used the Codeml program of the PAML package to calculate the Ks of *J. mandshurica* syntenic blocks [110]. Synteny analysis on 3 species, including *J. mandshurica, J. regia*, and *J. sigillata*, was performed to confirm the WGD event.

### RNA-seq and data analysis

RNA-seqwas performed by sampling different ripening stages of the *J. mandshurica* fruit at 30 (S1 stage, without hard kernel), 50 (S2 stage, without hard kernel), 70 (S3 stage, with a hard kernel), and 90 (S4 stage, with a hard kernel) days after natural pollination (Supplementary Fig. S24). Samples were frozen in liquid nitrogen immediately, and each stage contained 3 biological replicates. The walnut exocarp and embryos from fruits of each stage were collected and used for RNA-seq (Supplementary Fig. S25). A plant total RNA extraction kit (Takara, Beijing, China) was used to extract total RNA from the walnut exocarp and the embryos. RNA (1 μg) of each sample was used to construct a cDNA library. Sequencing libraries were generated using the TruePrep Flexible DNA Library Prep Kit for MGI (Vazyme, Nanjing, China) following the manufacturer's recommendations, and index codes were added to attribute sequences to each sample. Briefly, mRNA was purified from total RNA using poly-T oligo-attached magnetic beads. Fragmentation was performed using divalent cations under elevated temperature in NEBNext First Strand Synthesis Reaction Buffer (5×) (Ipswich, Massachusetts, USA). Random hexamer primer and RNase H were used for cDNA first-strand synthesis. Then, second-strand cDNA synthesis was performed using the buffer, dNTPs, DNA polymerase I, and RNase H. The library fragments were purified with QiaQuick PCR kits and elution with EB buffer, followed by terminal repair; the A-tailing and adapter added were implemented. Each library was completed after retrieving the target products and performing PCR. Finally, 24 libraries were constructed for RNA-seq, 12 of which were from the developing walnut exocarp, and the remaining were from developing embryos; these high-quality libraries were then sequenced using Illumina HiSeq 2500 platform with paired-end reads.

After sequencing and filtering, we obtained 99.65 Gb of clean data for walnut exocarp and 107.38 Gb of clean data for embryos, respectively (Supplementary Table S20). Filtered high-quality clean reads were aligned with the genome assembly of *J. mandshurica* using HISAT2 v2.1.0 (HISAT2, RRID:SCR_015530) [114] with the default parameters. Analysis of gene transcript abundance was performed using featureCounts (featureCounts, RRID:SCR_012919) [115] using the RNA-seq and Expectation Maximization software [116]. DEseq2 software (DESeq2, RRID:SCR_015687) [117] was used to detect DEGs. DEGs were screened based on the |log_2_Fold Change| ≥ 1 and adjusted *P* < 0.05. The TFs in *J. mandshurica* were detected using iTAK [118] and PlantTFDB (PLANTTFDB, RRID:SCR_003362) [119].

### Metabolomics

We collected fresh and healthy fruits, including the walnut exocarp and embryos, at 4 different stages, and each stage contained 3 biological replicates. The walnut exocarp and embryos were collected to extract metabolites, respectively. First, all collected samples were immediately loaded into a precooled centrifuge tube and frozen with liquid nitrogen. All the samples were then freeze-dried and crushed into a powder before using the mixer mill (MM 400; Retsch, Mannheim, Germany) with zirconia beads for 1.5 minutes at 30 Hz. In this process, 100 mg powder was dissolved in 1.2 mL 70% methanol extract. The extracts were vortexed for 30 seconds each (30 minutes with 6 repeats), and all were stored overnight at 4°C. The solution above was centrifuged at 12,000 rpm for 10 minutes, and the supernatant was collected and filtered with a microporous membrane (0.22 μm) and stored in an injection bottle for ultra-performance liquid chromatography/tandem mass spectrometry (UPLC-MS/MS) analysis. The UPLC-MS/MS analysis was implemented using multiple reaction monitoring (MRM) by the Wuhan MetWare Biotechnology Co., Ltd. (Wuhan, China). In particular, linear ion trap (LIT) and triple quadrupole (QQQ) scans were obtained from the AB 4500 Q TRAP UPLC-MS/MS system that was equipped with an ESI Turbo Ion-Spray interface on Analyst 1.6.3 software (AB Sciex, Framingham, USA) and operated in the positive ion mode. The detailed ESI operation parameters were as follows: ion source, turbine spray; source temperature, 550°C; and ion spray voltage, 5,500 V (positive ion mode) or −4,500 V (negative ion mode). Ion source gas I (GSI), gas II (GSII), and curtain gas were set at 50, 60, and 25.0 psi, respectively, and parameters of collision-induced ionization were set at a high level; tuning and quality calibration of the instrument were performed under QQQ and LIT mode with 10 and 100 µmol/L polypropylene glycol solution, respectively; QQQ scan was performed using MRM experiments, and the collision gas (nitrogen) was set at medium; declustering potential (DP) and collision energy (CE) for each MRM ion pair were obtained according to further DP and CE optimization; and according to the metabolites eluted in each period, a set of specific MRM ion pairs was monitored from this period. The mass spectrum data above were used for qualitative and quantitative analysis based on the MetWare database (MWDB) of MetWare Biotechnology Co., Ltd. (Wuhan, China) to obtain the original metabolite data. For quality control analysis, 1 quality control sample was inserted into each of the 10 test and analysis samples to monitor the repeatability of the analytic process. Partial least squares discriminant analysis was employed to screen variation components. The detailed methods were described in previous studies. The differentially expressed metabolites were screened based on the |log2Fold Change | ≥1 or *P* < 0.05, and variable importance in project ≥1. To study the specific accumulation of metabolites, we performed principal component analysis (PCA) of the metabolites that underwent a significant degree of changes using R [120]. The correlations between the differentially expressed genes and the metabolites were performed based on the Pearson correlation with the correlation coefficient at *r* > 0.8 or < −0.8. Correlation networks were used to visualize the relationships between the genes and metabolites using igraph package at OmicStudio tools [121].

### Functional gene analysis

To identify the *CYP450* genes related to juglone biosynthesis, we downloaded all annotated Arabidopsis CYP450 proteins from the TAIR database. The CYP450 family proteins of *Arabidopsis thaliana* were used as seed sequences, and the whole genome of *J. mandshurica* was searched using BLASTP [97] with the E value ≤1e^–5^. All candidate sequences were screened for the conserved CYP450 domain using Swissport and Batch NCBI CD-Search Tools (Batch Web CD-Search Tool, RRID:SCR_018756). In the expanded gene families of *J. mandshurica*, 14 *CYP450* genes were identified using the Upset process of TBtools and selected for phylogenetic analysis [122]. We constructed a phylogenetic tree to classify the members of the expanded CYP450 gene family in *J. mandshurica* and those related to *C. illinoinensis, C. cathayensis*, and *J. cathayensis*. All candidate sequences were compared using ClustalW (ClustalW, RRID:SCR_017277) in MEGA 7.0 (MEGA Software, RRID:SCR_000667) [119] software using the default parameters. Redundant genes were manually removed, and all nonredundant genes were used for further analysis.

## Data Availability

Raw reads used for genome assembly of *J. mandshurica* have been uploaded to the National Center for Biotechnology Information (NCBI) Sequences Read Archive (SRA) with the following accession numbers: SRR14637189 and SRR14629954. Transcriptomic data have been deposited in SRA with the accession number of PRJNA733587 (including embryos and exocarp). The assembled *J. mandshurica* genome has been deposited in the Genome Warehouse in the National Genomics Data Center (NGDC) under accession number PRJCA006358. Third-generation transcriptomic data have been deposited in the BIG Data Center under accession number PRJCA006794. The metabolomics data in this study were deposited and are available at the Metabolights repository under the accession number MTBLS3657 [123]. All supporting data and materials are available in the *GigaScience* GigaDB database [124].

## Additional Files

**Supplementary Fig. S1**. 17-mer analysis to estimate the *J. mandshurica* genome size.

**Supplementary Fig. S2**. Sequencing depth distribution of the assembled *J. manshurica* genome.

**Supplementary Fig. S3**. Cross-species comparisons of exon number, intron number, gene length, gene GC, CDS GC, exon length, CDS length, and intron length distribution.

**Supplementary Fig. S4**. Upset plot of genes annotated in GO, InterPro, KEGG, NR, Swissprot, and TrEMBL database.

**Supplementary Fig. S5**. The GO category analysis of rapidly expanded gene families in assembly of the *J. mandshurica* genome.

**Supplementary Fig. S6**. Schematic representation of syntenic genes among *J. mandshurica* and *J. regia*.

**Supplementary Fig. S7**. Genomic variation between *J. mandshurica* and the other 2 walnut species.

**Supplementary Fig. S8**. PCA score plot metabolite profiles from different sample groups during development of walnut exocarp.

**Supplementary Fig. S9**. PCA score plot metabolite profiles from different sample groups during development of walnut embryos.

**Supplementary Fig. S10**. Distribution of identified metabolites in green peel in *J. mandshurica*.

**Supplementary Fig. S11**. Venn diagrams of differentially expressed genes (DEGs) in S1, S2, S3, and S4 stages in green peel in *J. mandshurica*.

**Supplementary Fig. S12**. GO enrichment analysis of 897 core DEGs in *J. mandshurica*.

**Supplementary Fig. S13**. KEGG enrichment analysis of 897 core DEGs in *J. mandshurica*.

**Supplementary Fig. S14**. GO enrichment analysis of 990 DEGs identified in cluster 6.

**Supplementary Fig. S15**. KEGG enrichment analysis of 990 DEGs identified in cluster 6.

**Supplementary Fig. S16**. Upset plot of differentially expressed genes associated with juglone (*r*> 0.8 or < −0.8) in S1, S2, S3, and S4 stages in *J. mandshurica*.

**Supplementary Fig. S17**. KEGG enrichment analysis of 566 core DEGs associated with juglone (*r* > 0.8 or < −0.8) in S1, S2, S3, and S4 stages in *J. mandshurica*.

**Supplementary Fig. S18**. Distribution of identified metabolites in walnut kernels in *J. mandshurica*.

**Supplementary Fig. S19**. Distribution of identified lipid components in walnut kernel in *J. mandshurica*.

**Supplementary Fig. S20**. Heatmap of the free fatty acids during walnut kernel development in *J. mandshurica*.

**Supplementary Fig. S21**. KEGG enrichment analysis of different metabolites in 6 comparison groups. (a) S1 versus S2. (b) S1 versus S3. (c) S1 versus S4. (d) S2 versus S3. (e) S2 versus S4. (f) S3 versus S4.

**Supplementary Fig. S22**. Heatmap of the free fatty acids during walnut kernel development in *J. mandshurica*.

**Supplementary Fig. S23**. The schematic representation of syntenic genes among *J. mandshurica* and *J. cathayensis*.

**Supplementary Fig. S24**. Changes of *J. mandshurica* fruits in different development periods. S1 to S4 indicate the fruit collected at 30 days (S1 stage), 50 days (S2 stage), 70 days (S3 stage), and 90 days (S4 stage) after natural pollination.

**Supplementary Fig. S25**. The tissue structure of *J. mandshurica* fruit, including the walnut exocarp and walnut embryos.

**Supplementary Table S1**. The statistics of k-mer analysis.

**Supplementary Table S2**. BUSCO evaluation results for the *J. mandshurica* genome.

**Supplementary Table S3**. Statistics of genome alignment in *J. mandshurica*.

**Supplementary Table S4**. Statistical analysis of SNP and indel types in the *J. mandshurica* genome.

**Supplementary Table S5**. Transcriptome information of walnut exocarp and embryos.

**Supplementary Table S6**. Statistical results of the genetic structure of related species.

**Supplementary Table S7**. Summary of the functional annotation in the *J. mandshurica* genome.

**Supplementary Table S8**. Noncoding genes in the *J. mandshurica* genome.

**Supplementary Table S9**. Statistics of transposable elements and other repeats in the*J. mandshurica* genome.

**Supplementary Table S10**. Repeat sequence classification results statistics in the *J. mandshurica* genome.

**Supplementary Table S11**. Repeat elements in the *J. mandshurica* genome.

**Supplementary Table S12**. Comparison of the estimated number of gene families of *J. mandshurica* with other plants.

**Supplementary Table S13**. Summary of gene ortholog analysis conducted on 16 sequenced genomes.

**Supplementary Table S14**. The KEGG enrichment of the expanded gene family.

**Supplementary Table S15**. The KEGG enrichment of contracted gene family.

**Supplementary Table S16**. Statistical result of genomic variation between *J. mandshurica* and the other 2 walnut species.

**Supplementary Table S17**. The KEGG enrichment of obtained translocation events between *J. mandshurica* and *J. macrocarpa*.

**Supplementary Table S18**. The KEGG enrichment of obtained translocation events between *J. mandshurica* and *J. regia*.

**Supplementary Table S19**. Genes encoding enzymes related to juglone biosynthesis.

**Supplementary Table S20**. The genes of the rapidly expanded CYP450 gene family in *J. mandshurica* and its related species.

**Supplementary Table S21**. The identified metabolites isolated from walnut exocarp of *J. mandshurica*.

**Supplementary Table S22**. The differentially accumulated metabolites (DAMs) during different developmental stages of walnut exocarp.

**Supplementary Table S23**. The differentially expressed genes during different developmental stages of walnut exocarp.

**Supplementary Table S24**. The number of transcription factors in different comparison groups.

**Supplementary Table S25**. The differentially expressed genes correlated with the juglone (*r* > 0.80 or < −0.8).

**Supplementary Table S26**. The gene-to-metabolite correlation coefficient.

**Supplementary Table S27**. The identified metabolites isolated from the walnut embryo of *J. mandshurica*.

**Supplementary Table S28**. The identified metabolites involved in lipid in the embryo of *J. mandshurica*.

**Supplementary Table S29**. The transcription factors identified during the developmental embryo.

**Supplementary Table S30**. The transcription factors involved in lipid synthesis and oil accumulation.

giac057_GIGA-D-21-00355_Original_Submission

giac057_GIGA-D-21-00355_R1

giac057_GIGA-D-21-00355_R2

giac057_Response_to_Reviewer_Comments_Original_Submission

giac057_Response_to_Reviewer_Comments_Revision_1

giac057_Reviewer_1_Report_Original_Submission Peng Zhao -- 1/19/2022 Reviewed

giac057_Reviewer_1_Report_Revision_1 Peng Zhao -- 4/9/2022 Reviewed

giac057_Reviewer_1_Report_Revision_2 Peng Zhao -- 5/8/2022 Reviewed

giac057_Reviewer_2_Report_Original_Submission Nian Wang -- 1/23/2022 Reviewed

giac057_Reviewer_2_Report_Revision_1 Nian Wang -- 3/26/2022 Reviewed

giac057_Reviewer_3_Report_Original_Submission Annarita Marrano, Ph. D. -- 2/2/2022 Reviewed

giac057_Supplemental_Figures_and_Tables

## Abbreviations

BLAST: Basic Local Alignment Search Tool; bp: base pair; BUSCO: Benchmarking Universal Single-Copy Orthologs; DEGs: differentially expressed genes; DAMs: differentially accumulated metabolites; GATK: Genome Analysis Tool Kit; Gb: gigabase pairs; GO: Gene Ontology; HiFi: high fidelity; kb: kilobase pairs; KEGG: Kyoto Encyclopedia of Genes and Genomes; Ks: synonymous substitutions per synonymous; Mb: megabase pairs; mRNA: messenger RNA; mya: million years ago; NCBI: National Center for Biotechnology Information; NGDC: National Genomics Data Center; PacBio: Pacific Biosciences; TFs: transcription factors; TEs: transposable elements; WGD: whole-genome duplication.

## Conflict of Interest

The authors declare no competing interests.

## Funding

This research study was supported by the scientific research startup funds of Jilin Agricultural University (No. 2021002), the Innovation Project of State Key Laboratory of Tree Genetics and Breeding (Northeast Forestry University) (No. 2021A01), and the Fundamental Research Funds for the Central Universities (Northeast Forestry University) (No. 2572020DR01). The awardee of all funding is X. Zhao.

## Authors’ Contributions

X.L., K.W.C., Q.H.Z., and X.N.P. were major contributors in writing the manuscript; Z.M.H., Song.C., L.P.J., M.H.Z., and Y.L. contributed to plant sample collection, DNA/RNA preparation, library construction, and sequencing; S.K.Z., X.X.Z., Y.X.L., and Su.C. worked on genome assembly and annotation; V.C. and R.S. conducted transcriptome analysis and identified functional genes involved in juglone biosynthesis; G.Z.Q. and M.T. analyzed the gene family and constructed the evolutionary tree; and X.Y.Z. conceived of the study, participated in its design and data interpretation, and revised the manuscript critically.
