# Supplementary material for: The Manchurian Walnut Genome: Insights into Juglone and Lipid Biosynthesis
Source: Gigascience. 2022 Jun 28;11:giac057. doi: 10.1093/gigascience/giac057 (PMC9239856; doi:10.1093/gigascience/giac057)
Supplement: giac057_GIGA-D-21-00355_Original_Submission [file giac057_giga-d-21-00355_original_submission.pdf]

GigaScience

The Manchurian Walnut Genome: Insights into Juglone and Lipid Biosynthesis

--Manuscript Draft--

**Manuscript Number:**	GIGA-D-21-00355
**Full Title:**	The Manchurian Walnut Genome: Insights into Juglone and Lipid Biosynthesis
**Article Type:**	Research
**Funding Information:**	Heilongjiang Touyan Innovation Team Program (Tree Genetics and Breeding Innovation Team) — Mr Xiyang Zhao<br>State Key Laboratory Breeding Base of Mountain Bridge and Tunnel Engineering (2021A01) — Mr Xiyang Zhao<br>Fundamental Research Funds for the Central Universities (2572020DR01) — Mr Xiyang Zhao

Abstract:

Background

Manchurian walnut (*Juglans mandshurica* Maxim.) is a tree with multiple industrial uses and medicinal properties in the Juglandaceae family (walnuts and hickories). *J. mandshurica* produces juglone that is a toxic allelopathic agent and has potential utilization value.

Fundings

Here, we present a high-quality chromosome-scale reference genome assembly and annotation for *J. mandshurica* (n=16) with a contig N50 of 21 Mb by combining PacBio HiFi reads with Hi-C data. The assembled genome has an estimated sequence size of 548 Mb, and consists of 623 scaffolds and 40,453 protein-coding genes. 60.99% of the assembled genome consists of repetitive sequences. Sixteen super-scaffolds corresponding to the 16 chromosomes were assembled, with a scaffold N50 length of 33.7 Mb and a BUSCO complete gene percentage of 98.3%. *J. mandshurica* displays a close sequence relationship with *J. cathayensis* , with a divergence time of 13.8 million years ago (mya). Combining the high-quality genome, transcriptome and metabolome data, we constructed a gene-to-metabolite network and identified 566 core and conserved differentially expressed genes, which may be involved in juglone biosynthesis. Five *CYP450* genes were found that may contribute to juglone accumulation. Some candidate regulators (*e.g.* FUS3, ABI3, LEC2 and WRI1 TFs) involved in the regulation of lipid biosynthesis were also identified.

Conclusions

Our genomic data provides new insights into the evolution of the walnut genome and creates a new platform for accelerating molecular breeding and improving the comprehensive utilization of these economically important tree species.

**Corresponding Author:**	Xiyang Zhao<br>Northeast Forestry University<br>Harbin, CHINA
**Corresponding Author Secondary Information:**	
**Corresponding Author's Institution:**	Northeast Forestry University
**Corresponding Author's Secondary Institution:**	
**First Author:**	Xiang Li
**First Author Secondary Information:**	
**Order of Authors:**	Xiang Li

Powered by Editorial Manager® and ProduXion Manager® from Aries Systems Corporation

	Kewei Cai
	Qinhui Zhang
	Xiaona Pei
	Song Chen
	Luping Jiang
	Zhiming Han
	Minghui Zhao
	Yan Li
	Xinxin Zhang
	Yuxi Li
	Shikai Zhang
	Su Chen
	Guanzheng Qu
	Mulualem Tigabu
	Vincent L. Chiang
	Ronald Sederoff
	Xiyang Zhao
**Order of Authors Secondary Information:**	
**Additional Information:**	
**Question**	**Response**
Are you submitting this manuscript to a special series or article collection?	No
**Experimental design and statistics**<br><br>Full details of the experimental design and statistical methods used should be given in the Methods section, as detailed in our Minimum Standards Reporting Checklist. Information essential to interpreting the data presented should be made available in the figure legends.<br><br>Have you included all the information requested in your manuscript?	Yes
**Resources**<br><br>A description of all resources used, including antibodies, cell lines, animals and software tools, with enough information to allow them to be uniquely identified, should be included in the	Yes

Methods section. Authors are strongly encouraged to cite Research Resource Identifiers (RRIDs) for antibodies, model organisms and tools, where possible.<br><br>Have you included the information requested as detailed in our Minimum Standards Reporting Checklist?	
**Availability of data and materials**<br><br>All datasets and code on which the conclusions of the paper rely must be either included in your submission or deposited in publicly available repositories (where available and ethically appropriate), referencing such data using a unique identifier in the references and in the "Availability of Data and Materials" section of your manuscript.<br><br>Have you have met the above requirement as detailed in our Minimum Standards Reporting Checklist?	Yes

Manuscript Click here to access/download;Manuscript;manuscript.docx

The Manchurian Walnut Genome: Insights into Juglone and Lipid Biosynthesis

Xiang Li[1,2,†], Kewei Cai[2,†], Qinhui Zhang[2,†], Xiaona Pei[1,†], Song Chen[2], Luping Jiang[2], Zhiming Han[2], Minghui Zhao[2], Yan Li[2], Xinxin Zhang[2], Yuxi Li[2], Shikai Zhang[2], Su Chen[2], Guanzheng Qu[2], Mulualem Tigabu[3], Vincent L. Chiang[2,4], Ronald Sederoff[4], Xiyang Zhao[1,2,*]

[1] College of Forestry and Grassland, Jilin Agricultural University, Changchun 130117, China.

[2] State Key Laboratory of Tree Genetics and Breeding, School of Forestry, Northeast Forestry University, Harbin 150040, China.

[3] Southern Swedish Forest Research Centre, Swedish University of Agricultural Sciences, Alnarp SE-230 53, Sweden.

[4] Forest Biotechnology Group, Department of Forestry and Environmental Resources, North Carolina State University, Raleigh NC 27695, USA.

[†] These authors contributed equally to this work.

lx2016bjfu@163.com (X.L.);
ckwnefu@163.com (K.C.);
zqh19950201@163.com (Q.Z.);
xiaonapei2020@163.com (X.P.);
chengsongnet@gmail.com (S.C.);
zhuojiuyibei@163.com (L.J.);
h375347046@163.com (Z.H.);
zhaominghui66@163.com (M.Z.);
ly2019nefu@163.com (Y.L.);
zhangxinxin@nefu.edu.cn (X.Z.);
fagongzi@163.com (Y.L.);
zskphd@163.com (S.Z.);
chensunefu@163.com (S.C.);
gzqu@nefu.edu.cn (G.Q.);
mulualem.tigabu@slu.se (M.T.);
vchiang@ncsu.edu (V.L.C.);
ron_sederoff@ncsu.edu (R. S.)

Corresponding author:
Email: zhaoxyphd@163.com (X.Z.)
Tel.: +0086-451-82192225
Fax: +0086-451-82192225

Abstract

Background: Manchurian walnut (*Juglans mandshurica* Maxim.) is a tree with multiple industrial uses and medicinal properties in the Juglandaceae family (walnuts and hickories). *J. mandshurica* produces juglone that is a toxic allelopathic agent and has potential utilization value. **Fundings:** Here, we present a high-quality chromosome-scale reference genome assembly and annotation for *J. mandshurica* (n=16) with a contig N50 of 21 Mb by combining PacBio HiFi reads with Hi-C data. The assembled genome has an estimated sequence size of 548 Mb, and consists of 623 scaffolds and 40,453 protein-coding genes. 60.99% of the assembled genome consists of repetitive sequences. Sixteen super-scaffolds corresponding to the 16 chromosomes were assembled, with a scaffold N50 length of 33.7 Mb and a BUSCO complete gene percentage of 98.3%. *J. mandshurica* displays a close sequence relationship with *J. cathayensis*, with a divergence time of 13.8 million years ago (mya). Combining the high-quality genome, transcriptome and metabolome data, we constructed a gene-to-metabolite network and identified 566 core and conserved differentially expressed genes, which may be involved in juglone biosynthesis. Five *CYP450* genes were found that may contribute to juglone accumulation. Some candidate regulators (*e.g.* FUS3, ABI3, LEC2 and WRI1 TFs) involved in the regulation of lipid biosynthesis were also identified. **Conclusions:** Our genomic data provides new insights into the evolution of the walnut genome and creates a new platform for accelerating molecular breeding and improving the comprehensive utilization of these economically important tree species.

Keywords: *Juglans mandshurica*, PacBio SMART, Hi-C, HiFi, genome assembly, Comparative genomics, Juglone, Lipid

Data Description

Juglans mandshurica Maxim. (2n=2x=32), well known as "Manchurian walnut", is a fast-growing and valuable hardwood tree species. The family Juglandaceae contains ~23 species, all bearing edible and medicinal nuts [1]. *J. mandshurica* was widely cultivated in China, Korea, Siberia, Japan, India, and Russia. It is naturally distributed in the northeast regions of China [2]. Because of its highly desirable wood quality and medically active substances, *J. mandshurica* was widely used in construction, wood processing, oil production, medicine and pesticide manufacturing. Its immature walnut peel (exocarp) contains bioactive components, including quinones, triterpenoids, flavonoids, phenolics and alkaloids, which can induce detumescence and analgesia, softening blood vessels and producing anti-inflammatory effects. Juglone (5-hydroxy-1,4-naphthoquinone, $C_{10}H_6O_3$) was sued for its anticancer activity [3-5]. The walnut embryos of *J. mandshurica* has a high fatty acid content (more than 60%), which is composed of linoleic acid, oleic acid, linolenic acid, palmitic acid and stearic acid. These fatty acids may aid in the prevention of coronary heart disease by decreasing blood lipids, enhancing immune functions, and modulating non-alcoholic fatty liver disease [6, 7]. In *J. mandshurica*, other tissues, including roots, stems, leaves, branches and bark may also have significant medicinal value [8].

Juglone forms orange acicular (long and needle-like) crystals and exhibits various biological activities and multi-purpose applications. Juglone is a naphthoquinone heterocyclic compound, was firstly isolated and purified in the 1950s and artificially synthesized in 1887 [9]. Juglone has antibacterial, antitumor, antiviral, and anti-inflammatory effects and is mainly derived from the root, bark, leaves and immature walnut exocarp (peel) tissues of some Juglandaceae species, including *Carya cathayensis*, *C. illinoinensis*, *Juglans cathayensis*, *J. hindsii*, *J. nigra*, *J. regia*, *J. sigillata*, and *J. macrocarpa* [3, 9]. Juglone was considered as a potential new drug thus its separation, preparation, synthesis and biological activities have been extensively studied [10-12]. In humans, juglone has antitumor activity and can significantly inhibit liver, colon, lung and pancreatic cancer [13]. Juglone and related naphthoquinones are enriched in immature walnut exocarp compared with bark and root. Further research is needed for effectively extracting high purity juglone. Juglone has toxic effects on some plants, showing obvious allelopathy, and therefore has been used as an effective bio-herbicides [9]. There remains great potential to utilize juglone for allelopathy, synthetic agrochemicals and natural colorants in agriculture. *J. mandshurica* might become a crucial plant resource for biomedical research. Targeted studies on its biosynthesis and molecular function are needed to explore its application to human health and economic development.

Understanding the regulation of genes involved in the biosynthesis of juglone in *J. mandshurica* could accelerate the utilization of juglone resources. There are at least four different natural metabolic pathways to synthesize 1,4-naphthoquinone (1,4-NQs) [14]. However, the biosynthesis and regulation of juglone remains unknown in plants, and only one primary biosynthetic pathway and a small number of genes are known based on the study of roots and leaves in black walnut (*J. nigra*) [15]. The biosynthesis

of juglone is related to the phylloquinone (vitamin K1) pathway, which shares the 1,4-dihydroxynaphthoic acid (DHNA) to synthesize 1,4-NQs by decarboxylases. Ultimately, 1,4-NQs react with 2-oxoglutarate/Fe (II)-dependent dioxygenase (2-ODD) families or by cytochrome CYP450s to form juglone. Additional transcription factors (TFs) and other elements may also participate in juglone biosynthesis. The biosynthesis of juglone has not been systematically studied by multi-omics and genome-based methods. To implement such a strategy, a genome sequence is needed.

In this work, we assembled a high-quality chromosome-level reference genome (548 Mb) of *J. mandshurica* by high fidelity (HiFi) long reads based on the Pacific Biosciences (PacBio) sequencing platform and high-throughput chromosome conformation capture (Hi-C). We detected and annotated 40,453 gene models and 24,415 gene families in *J. mandshurica*. The analysis of gene family evolution and divergence was also performed in this study. We screened potential transcription factors and candidate genes involved in the juglone and lipid biosynthesis pathways. We mapped a gene-to-metabolite network by combining data on the genome, transcriptome and the metabolome. This work provides valuable genetic information on the evolution of *J. mandshurica* and related species, and also contributes to further elucidation of the juglone and lipid biosynthetic pathways.

Methods and analyses

Sequencing and assembly of the *Juglans mandshurica* genome

Fresh leaves, bark and fruit of adult *J. mandshurica* were collected at Northeast Forestry University (126°37'57.28" E, 45°43'6.53" N), in Harbin, Heilongjiang province, China. High quality genomic DNA from fresh leaves was extracted by an improved CTAB method [16]. For long-read DNA sequencing, 15 µg of sheared DNA was used for circular consensus sequencing (CCS). SMRT Bell libraries were constructed from DNA randomly cut into ~15 Kb fragments and then sequenced on the PacBio Sequel II platform (14.62 Gb data, 25-fold coverage of the genome). For short-read DNA sequencing, libraries were constructed from 300-500 bp fragments and sequenced using the WGS Illumina HiSeq platform. Totally, 43.18 Gb of raw data were obtained. For RNA sequencing and metabolite quantification, the different ripening stages of *J. mandshurica* fruit were sampled at 30 days (S1 stage), 50 days (S2 stage), 70 days (S3 stage) and 90 days (S4 stage) after natural pollination (Figure S1). Samples were frozen in liquid nitrogen immediately and each stage contained three biological replicates. The walnut exocarp and embryos (Figure S2) from fruits of each stage were collected and used for RNA sequencing. A plant total RNA extraction kit (Takara, Beijing, China) was used to extract total RNA from the walnut exocarp and the embryos. 20 µg of RNA of each sample was used to construct a cDNA library, and then sequenced on the Illumina HiSeq 2500 platform with paired end reads.

We calculated the frequency of each 17-mer from the Illumina WGS sequencing reads (43.2 Gb) and examined the distribution of the 17-mer numbers. The raw data obtained in the PacBio Sequel II CCS sequencing mode was converted to HiFi data by CCS software using default parameters. The assembly software Hifiasm (version 0.2.0)

[17] was used on the HiFi data to and get a preliminary assembly.

The Hi-C sequencing was performed on an Illumina HiSeq platform with PE 150 bp and yielded 54Gb of sequence. Throughout the construction of the Hi-C library and sequencing, we used HTQC (v1.92.310) [18] software to perform quality control on the raw data and obtained clean data. For clean data, BWA [19] software (0.7.17) was used for comparison, and then used for chromosome construction. Based on the principle of chromosome interaction, we used 3D-DNA [20] software to cluster, sort and orient contigs or scaffolds to obtain the chromosome level genome.

The final reference assembly contained 16 chromosome-scale pseudomolecules, with maximum and minimum lengths of 52 Mb and 19 Mb, respectively. A heat map of the interaction matrix of all pseudochromosomes was plotted with a resolution of 500 kb. The assembled chromosome number is the same as the haploid chromosome number of *J. mandshurica* (n = 16) (Figure 1a). A single adult Manchurian walnut tree from northeast China was selected for whole-genome sequencing and assembly (Figure S3). The genome size of *J. mandshurica* estimated by *K*-mer analysis is ~484 Mb, with a level of heterozygosity of 0.96 %. 41,412,724,319 17-mers were identified based on Illumina sequencing data, and the 17-mer depth was 82 (Table S1, Figure S4). To assembly this highly heterozygous genome, Illumina, PacBio and Hi-C (high-throughput chromosome conformation capture) technologies were selected for whole-genome sequence. 14.62 gigabases (Gb), CCS HiFi long reads with ~25x sequence depth (Figure S5), and 43.18 Gb WGS Illumina short reads were obtained, using Sequel II and Illumina Hiseq2000 platforms, respectively (Table 1). The HiFiasm [17] software was used to assemble the *J. mandshurica* genome, and a 549 Mb assembly was obtained. The genome size was higher than the results of *K*-mer analysis, which may be due to heterozygosity. The *J. mandshurica* assembly was further improved using Hi-C paired-end reads using 53 Gb of Hi-C data. As a result, 0.53 Gb were distributed across 16 chromosome-scale scaffolds using 3D-DNA [20], occupying 96.26% of the final genome assembly (Figure 1b). After primary correction and assembly, the initial contig number, total length and N50 size were 657, 548,677,591 bp (~548 Mb) and 21,388,210 bp (~21Mb), respectively. A total of 623 long scaffolds were finally anchored and oriented on 16 pseudochromosomes with a scaffold N50 of 35,382,463 bp (~35.38 Mb).

After assembly was completed, we evaluated the results using three methods. First, the comparison tool minimap2 (v2.5) [21] was used to compare the assembled genomes, and the comparison rate of reads, the extent of genome coverage and the distribution of depth were calculated to evaluate the integrity of the assembly and the uniformity of sequencing coverage. Secondly, the Burrows-Wheler Aligner (BWA) [19] was used to compare reads to the reference genome. Finally, based on the single-copy homologous gene set in OrthoDB [22, 23], BUSCO (v5.2.2) [23] was used to predict these genes and calculate their integrity, fragmentation, and possible loss rates. In addition, the Benchmarking Universal Single-Copy Orthologs (BUSCO) software [23] was employed to evaluate the completeness of the *J. mandshurica* assembly. The BUSCO assessment shows that 98.3% of the complete BUSCO gene set was captured, indicating a higher BUSCO evaluation score (Table S2). We obtained a total of clean bases of mRNA-seq (Gb), and average of 51,559,765 (93.17%) and 55,542,790 (93.05%) reads

obtained from transcriptome sequence (RNA-seq) in walnut exocarp and kernel could be mapped back to the genome assembly, respectively (Table S3). These results for the assembly and assessment verified that we obtained a high-quality reference genome with high completeness at the chromosome level.

Table 1: The statistics for genome sequencing of *Juglans mandshurica*

Genomic features	*Juglans mandshurica*
Raw bases of WGS-PacBio Sequel II (Gb)	14.62
Raw bases of HiC (Gb)	53
Raw bases of WGS Illumina (Gb)	43.18
Clean bases of mRNAseq (Gb)	207.03
Genome size (Mb)	548.7
GC content of the genome (%)	36.72
Number of scaffolds	623
Chromosome-scale scaffolds (bp)	528,151,479
N50 of scaffolds (bp)	35,382,463
Number of contigs	657
Size of contigs (bp)	548,677,591
N50 of contigs (bp)	21,388,210

Gene prediction and annotation of the *Juglans mandshurica* genome

We used homologous and *de novo* annotation to identify repetitive sequences. Firstly, RepeatMasker (Open-4.0.9) [24] and RepeatProteinMask (Open-4.09) [24] were used to search for TE sequences from Repbase (release 21.01) [25] based on homology. Secondly, RepeatModeler (Open-1.0.11) [26] and LTR-Finder [27] (v1.0.7) were used to construct a repeat sequence database, and then Repeatmasker (Open-4.09) [24] was used to identify the repetitive sequences. TRF [28] was used to identify tandem repeat sequences. Finally, the results based on homologous annotation and *de novo* annotation were integrated and the non-redundant elements after overlapping, were removed for the final repeated sequence annotation.

We used homologous, *de novo* and transcriptome assisted annotation to predict the structure and function of coding genes. For homologous annotation, 3-5 related species were selected, and then TblastN [29] (E-value cutoff of 1e-5) was used to compare the related species to the reference genome. Then, the aligned sequences and their corresponding proteins were filtered and transmitted to the Exonerate [30] for accurate alignment. Augustus (v3.3.1) [31] and GlimmerHMM (v3.0.4) [32] were used for *de novo* annotation. For isoform-sequencing (Iso-Seq) data, we used Gmap [33] to align it to the reference genome, and then used TransDecoder [34] to predict open reading frames (ORFs) in the transcripts to define putative coding sequences (CDS). Maker (v3.00) [35] was used to integrate the predicted gene sets into a non-redundant, more complete and reliable gene set. Finally, the proteins in the gene collection were annotated by means of curated protein databases including SwissProt, TrEMBL, KEGG, GO and NR using NCBI BLASTP (ncbi blast v2.6.0+) [34] (E-value $\leq$1e−5).

Totally, 40,515 protein-coding genes were predicted, with an average gene length

of 3574.61 bp by combining the *de novo*, transcriptome or IsoSeq (Isoform sequencing) and homology-based methods using MAKER (v3.00 [35] (Table S4). For these predicted genes, 40,453 (99.85%) of the genes were anchored to the pseudochromosomes, and the average exon number per gene was 6.1 with an average length of 289.0. and the average CDS length was 1105. bp (Figure S6). The total GC content of the genome assembly was 36.7 % and was distributed across 16 pseudochromosomes (Table 1 and Figure 1a). Among these genes, 32,901 (81.33%) of the genes were functionally annotated to InterPro (25,953, 64.16%), Gene Ontology (GO) (18,229, 45.06%), Kyoto Encyclopedia of Genes and Genomes (KEGG) (31,243, 77.23%), Swissprot (23,059, 57%), TrEMBL (31,341, 77.48%) and NR (32,855, 81.22%) public databases, and the number of core genes annotated in all databases above were 16,218 (Table S5 and Figure S7). The gene distribution in 16 pseudochromosomes of the *J. mandshurica* genome was uneven, as found in other plant species (e.g., *Rhododendron simsii* (azalea) and *Sechium edule;* (chayote) [36, 37] (Figure 1a). In the annotation process of non-coding RNA, according to the structural characteristics of tRNA, tRNA sequences in the genome were searched using tRNAscan-SE (v1.3.1) [38]. Because rRNA is highly conserved, rRNA sequences from related species can be selected as reference sequences to search for rRNA by BLASTN (v2.6.0) [34] alignment. Infernal of Rfam [39] was used to predict miRNA and snRNA sequences in the genome. We identified several noncoding RNA genes, containing 122 microRNAs (miRNAs), 2,185 transfer RNAs (tRNAs), 4,004 ribosomal RNAs (rRNAs) and 272 small nuclear RNAs (snRNAs) in the *J. mandshurica* genome (Table S6 and Figure 1a).

We also identified 334,673,373 bp of repetitive sequences combining the *de novo* and homology-based approaches in *J. mandshurica*, accounting for 60.99% of the genome assembly (Table S7 and Figure 1a). 326,580,986 bp (59.52%) of transposable elements (TEs) were found, which is comparable to *Acer truncatum* (Shantung maple) [40] (~61.75%) and *Eucommia ulmoides* (hardy rubber tree) [41] (~62.5%) but higher than that in *Tripterygium wilfordii* (thunder duke vine) [42] (52.36%) and *Betula platyphylla* (white birch) [43] (43.0%). The predominant TEs were long terminal repeat (LTR) retrotransposons, accounting for 39.35% of the assembled genome, followed by the DNA transposons (9.39%), long interspersed nuclear elements (LINEs, 8.38%) and short interspersed nuclear elements (SINEs, 0.35%) (Table S7). The majority of the TEs were gypsy and copia-like LTRs that covered 65,135,736 bp and 93,428,433 bp respectively, accounting for 11.87 % and 17.03% of the assembled genome (Table S8 and Table S9).

Gene family identification and evolutionary analysis

To identify the gene families in each species, we clustered the proteins of 15 species through the OrthoMCL (V14-137) [44] process based on sequence similarity with the parameter of "-inflation 1.5.", including 13 rosid species (i.e., *Juglans cathayensis* [45], *Juglans macrocarpa* [45], *Juglans nigra* [45], *Juglans regia* [45], *Juglans hindsii* [45], *Juglans sigillata* [45] *Carya cathayensis* [46], *Carya illinoinensis*

[46], *Quercus lobata* (valley oak) [47], *Castanea mollissima* (Chinese chestnut) [48], *Morella rubra* (red bayberry) [49], *Populus trichocarpa* [50] and *Vitis vinifera* [51] and one commelinid species (*Oryza sativa*) [52] . Muscle (V3.8.31) [53] was used to conduct multiple sequence alignments of genes within the single-copy homologous gene family of each species, and RAxML (V8.2.12) [54] was used to construct an evolutionary tree using Maximum Likelihood.

Utilizing the constructed evolutionary tree, combining with the Timetree (http://www.timetree.org/) website and literature to obtain time correction points, using the software R8S [55] (v1.71) and mcmctree (v4.9e) in the PAML software [56], the bifurcation time was estimated with five corrected divergence time point from the TimeTree website (http://www.timetree.org/): *O. sativa* vs. *J. mandshurica* (115-308 Mya), *V. vinifera* vs. *J. mandshurica* (107-135 Mya), *P. trichocarpa* vs. *J. mandshurica* (101-131Mya), *Q. lobata* vs. *C. mollissima* (6-49 Mya), *Q. lobata* vs. *J. mandshurica* (51-87 Mya), and two corrected divergence time point from *M. rubra* genome article [57]: *M. rubra* vs *Juglans* genus (28-34 Mya) and *Carya* genome article [46]: genus *Juglans* vs. genus *Carya* (~23 Mya). CAFÉ [58] was used to simulate the expansion and contraction events of gene families in each lineage of the evolutionary tree.

The results showed that *J. mandshurica* shared 7,686 gene families with four related plant species, and possessed 2,584 single-copy orthologs and 225 unique families (Figure 2a and Table S10). Particularly, the number of single- and multiple-copy genes of *J. mandshurica* was similar to that in the other genus *Juglans* species (Figure 2b and Table S11). To study the genome evolution of *J. mandshurica* and the Juglandaceae, 558 single-copy orthologous genes from 13 species of rosid families (i.e., *Juglans cathayensis*, *J. macrocarpa*, *J. nigra*, *J. regia*, J. *hindsii*, *J. sigillata*, *Carya cathayensis*, *C. illinoinensis*, *Quercus lobata* (valley oak), *Castanea mollissima* (Chinese chestnut), *Morella rubra* (red bayberry), *Populus trichocarpa* and *Vitis vinifera* and one commelinid species (*Oryza sativa*) were identified by OrthoMCL [44] and employed to construct a phylogenetic tree and to evaluate the divergence times using RAxML (version 8.2.11) [54] employing default settings (Figure 2c). 40,453 genes were clustered into 24,415 (60.35%) gene families in *J. mandshurica* with an average of 1.48 genes per family (Table S10). *J. mandshurica* showed a close relationship with *J. cathayensis*, with an estimated divergence time of 13.8 (10.6-17.3) million years ago (mya). All of the species in the genus Juglans were clustered in the same group, and they shared a common ancestor with species in the genus *Carya* having diverged approximately 23.7 (20.1-26.9) mya. Within the genus Carya, the divergence of its two species was estimated at 5.4 (3.0-9.8) mya. Juglandaceae and Myricaceae (such as *Morella rubra*) species diverged approximately 36.6 (32.2-34.5) mya. We also carried out gene family expansion and contraction analysis across the related species using CAFÉ to explore the evolution of *J. mandshurica* (Figure 2c). Of the 24,415 gene families, 798 and 405 expanded and contracted in *J. mandshurica*, respectively, after divergence from *J. cathayensis*. In addition, for the gene families of Juglandaceae species, the gene family number of expansions was high compared to contractions in *J. macrocarpa* and *J. regia*. More gene families expanded (1,103 and 687) and fewer gene families contracted (533 and 691) in Fagaceae species, including *Quercus lobata* and

Castanea mollissima. Totally, 521 (65.29%) of 798 expanded gene families displayed rapid evolution in the *J. mandshurica* genome (family-wide *p*-value < 0.05) and annotated in functions related to metabolic process, cellular processes, cell, cell part, binding and catalytic activity based on the GO category (Figure S8). These genes from rapidly expanded gene families were further clustered into 88 KEGG pathways. The expanded gene families mainly involved in photosynthesis (ko00195), ribosome (ko03010), MAPK signaling pathway (ko04010), plant-pathogen interaction (ko04626) and protein processing in endoplasmic reticulum (ko04141) (Table S12). The contracted families were annotated to 20 KEGG pathways, and they mainly participated in NF-kappa B signaling pathway (ko04064), toll and Imd signaling pathway (ko04624), toll-like receptor signaling pathway (ko04620) and MAPK signaling pathway (ko04010) (Table S13).

Analyses of genome synteny and whole-genome duplication (WGD)

The genomes of *J. regia* and *P. trichocarpa* were selected as comparisons for collinearity analysis with the *J. mandshurica* genome. MCscan [58] was used to perform synteny searches, with at least thirty gene pairs required per syntenic block. Then TBtools [59] was used to visualize the schematic diagram. Mummer (v4.0.0beta2) [60] was used to estimate the collinearity between the genomes of *J. regia* and *J. mandshurica*.

Mcscan was used to search for collinear regions of species genomes, and the 4dTV of gene pairs contained in the collinear regions were calculated to reflect the relative differentiation events and whole-genome duplication in the evolutionary history of *J. mandshurica*. We used the Codeml program of the PAML [56] package to calculate the ks of *J. mandshurica* syntenic blocks. Synteny analysis on three species including *J. mandshurica, J. regia* and *J. sigillata* was performed to confirm the WGD event.

Whole-genome duplication (WGD) occurred in the evolutionary history of most plant species and provided evolutionary potential for new functions and species diversification [61]. We computed Ks (synonymous substitutions per synonymous) and 4dTV (fourfold degenerate synonymous sites of the third codons) value among the *J. mandshurica*, *J. regia* and *J. sigillata* genes to analyze gene duplication and divergence. The fossil record showed that Juglandaceae appeared in the upper Cretaceous and the family radiated in the Paleocene [62-64]. The Juglandoid WGD must have therefore originated prior to the radiation of Juglandaceae in the Paleocene. We therefore chose the Cretaceous-Paleogene (C-Pg) boundary (66 mya) as the approximate time of the origin of the Juglandoid WGD. The distribution of these two methods is remarkably consistent (Figure 3a, b). The Ks plot of Jma_vs_Jma, Jre_vs_Jre, Jsi_vs_Jsi (self-searches within the *J. mandshurica*, *J. regia* and *J. sigillata genomes*) reflected the divergence of paralogous genes, which originated by the Juglandoid WGD, it showed a significant main peak around 0.3, which is consistent with the results of other Juglandoid research [65].

We also detected synteny between the assembly of the *J. mandshurica* genome and that of *J. regia*. Synteny analysis showed strong correspondence for all 16 chromosomes in these plants, indicating that the collinearity has been maintained at a

high level, which indicates a close evolutionary relationship of these two species (Figure 3c and Figure S9). A large number of collinear gene pairs were found between *J. mandshurica* chromosomes (Figure 1a). We detected linear relationships between *J. mandshurica*, *J. regia* and *P. trichocarpa*, and there are significant distinct syntenic blocks. 49,921 and 38,462 collinear genes were identified between *J. mandshurica* and *J. regia* and between *J. mandshurica* and *P. trichocarpa*, respectively, indicating that 62.5% and 52.3% of the *J. mandshurica* genome was colinear in these plants. For example, Chr2 of *J. mandshurica* shares origins with Chr3 and Chr4 in *J. regia* and with Chr5 and Chr7 in *P. trichocarpa* (Figure 3c). The results suggested that ancestral collinearity existed between these three species.

Gene discovery analysis related to Juglone biosynthesis

To identified the *CYP450* genes related to juglone biosynthesis, we downloaded all annotated Arabidopsis CYP450 proteins of from the TAIR database. The CYP450 family proteins of *Arabidopsis thaliana* was used as seed sequences, and the whole genome of *J. mandshurica* was searched using BLASTP [34] with the E value $\leq 1e^{-5}$. All the candidate sequences were screened for the the CYP450 domain using Swissport. 14 expanded *CYP450* genes were identified using the Upset process of TBtools [59] and selected for phylogenetic analysis. To classify the members of the expanded CYP450 gene family in *J. mandshurica* and related *C. illinoinensis*, *C. cathayensis* and *J. cathayensis*, we constructed a phylogenetic tree. All candidate sequences were compared using ClustalW in MEGA 7.0 [66] software using the default parameters. Redundant genes were manually removed, and all non-redundant genes were used for further analysis.

As a forest tree species of high economic value, *J. mandshurica* specifically accumulates juglone in its roots, leaves, bark, and especially the walnut exocarp [9]. Juglone is an important quinoid component. Its medicinal activity and potential use for sustainable agriculture has been confirmed [9]. However, the biosynthesis, mechanism of action and regulatory network involved in the juglone pathway is still unclear, and only a few of the relevant genes have been identified using RNA-sequencing [15]. The biosynthesis of juglone in walnut species is known to be partly derived from the phylloquinone pathway, where the initial substrate was chorismite from the shikimate pathway [15]. These two pathways shared the 1,4-dihydroxynaphthoic acid (DHNA) intermediate, and seven enzymes are used for the DHNA synthesis of phylloquinone. Then, DHNA is converted to 1,4-naphthoquinone (1,4-NQs), by various decarboxylases. Through hydroxylases, CYP450s and 2-ODD, the 1,4-NQs synthesize juglone by hydroxylation. In this study, combining the genomic, transcriptomic and metabolomic technologies, we were able to analyze the process of fruit development in *J. mandshurica*, and to identify genes regulating juglone biosynthesis in the jugland exocarp. 146 candidate genes encoding the enzymes for the juglone biosynthesis were found including one gene encoding isochorismate synthase, two genes encoding SEPHCHC, SHCHC and OSB, one genes coding OSB-CoA ligase, two genes coding DHNA-CoA, one genes coding DHNA-CoA thioesterase, one genes coding DHNA phytyl transferase, four genes coding NDC1, two genes coding demethylphylloquinone methyltransferase, five genes coding decarboxylases, two genes coding 2-ODDs and

125 genes coding *CYP450* (Figure 4a and Table S14). The expression level of the decarboxylases, *2-ODDs* and the *CYP450* genes in the S3 and S4 stages was higher than the S1 and S2 stages, indicating a potential role for these genes in juglone biosynthesis in *J. mandshurica*.

We next explored the specifically expanded gene families that may be involved in juglone biosynthesis. CYP450s are a class of important oxidative enzymes, which are widely distributed in plants, and play key roles in the biosynthesis of many natural secondary metabolites. CYP450 enzymes may catalyze various enzymatic steps in the juglone biosynthesis, phytohormones biosynthesis and plant stress responses. For the CYP450s, the most representative catalytic reaction is hydroxylation, and they also catalyze other complex biosynthetic reactions such as epoxidation of aromatic compounds and methyl or amino transfer reactions (transferases). The CYP gene family (14 genes in four groups) were identified for the *J. mandshurica* genome, and appear to have rapidly expanded in the *J. mandshurica* genome compared to the three related species (14 genes in *J. mandshurica* genome, one in *C. illinoinensis*, two in *C. cathayensis*, three in *J. cathayensis*) (Figure 4b and Table S15). The 14 genes in *J. mandshurica* are specifically distributed on chromosome 2, chromosome 4 and chromosome 6, and five of the fourteen genes were in the same group (Group Ⅲ) (Figure 4c). The 14 CYP genes in *J. mandshurica* were also found in the differentially expressed genes (DEGs) obtained by RNA-seq of the immature exocarp (Figure 4d). The majority of CYP genes were differentially expressed in the S4 stage. The CYP gene family exhibited particularly rapid expansion and the inferred increase in transcript abundance may contribute to the accumulation of juglone.

Metabolite profiling and transcriptomics of walnut exocarp development

Twenty-four libraries were constructed for RNA-seq, in which 12 were from developing walnut exocarp, and the remaining were from developing embryos. After sequencing and filtering, we obtained 99.65 Gb of clean data for walnut exocarp and 107.38 Gb of clean data for embryos, respectively (Table S20). Filtered high-quality clean reads were aligned with the genome assembly of *J. mandshurica* using HISAT2 (v2.1.0) [67] with the default parameters. Analysis of gene transcript abundance was carried out by featureCounts [68] using RNAseq and Expectation Maximization (RSEM) software [69]. DEseq2 software [70] was used to detect differentially expressed genes (DEGs). Differentially expressed genes (DEGs) were screened based on the $|\log_2\text{Fold Change}| \geq 1$, and adjusted P-value <0.05. The transcription factors in *J. mandshurica* were detected using iTAK [71] and PlantTFDB [72].

The fresh, healthy fruit (including walnut exocarp and embryos) of four different stages were collected, each stage contained three biological replicates. The walnut exocarp and embryos were collected to extract metabolites, respectively. Firstly, all collected samples were immediately loaded into a precooled centrifuge tube and frozen with liquid nitrogen. Then, all the samples were freeze-dried and crushed into a powder prior using mixer mill (MM 400, Retsch) with zirconia beads for 1.5 min at 30 Hz. In which, 100 mg powder were dissolved in 1.2 mL 70 % methanol extract. The extracts were vortexed for 30 second each 30 min with six repeats, and then all samples placed

at 4 ℃ refrigerator overnight. The solution above was centrifuged at 12000rpm for 10min, the supernatant was collected and filtered with a microporous membrane (0.22 μm) and stored in an injection bottle for ultra-performance liquid chromatography/tandem mass spectrometry (UPLC-MS/MS) analysis. The UPLC-MS/MS [78] analysis was implemented using multiple reaction monitoring (MRM) by the Wuhan MetWare Biotechnology Co., Ltd., (Wuhan, China). Particularly, linear ion trap (LIT) and triple quadrupole (QQQ) scans were obtained from AB 4500 Q TRAP UPLC/MS/MS system that was equipped an ESI Turbo Ion-Spray interface on Analyst 1.6.3 software (AB Sciex) and operated in the positive ion mode. The detail ESI operation parameter were as follows: Ion source, turbine spray; source temperature, 550 ℃; ion spray voltage (IS), 5500 V (positive ion mode) or -4500 V (negative ion mode); ion source gas I (GSI), gas II (GSII) and curtain gas (CUR) were set at 50, 60 and 25.0 psi, respectively, and the parameters of collision induced ionization are set at high level; tuning and quality calibration of instrument were performed under QQQ and LIT mode with 10 and 100 μmol/L polypropylene glycol solution, respectively; QQQ scan was performed using MRM experiments, and the collision gas (nitrogen) was set at medium level; DP and CE for each MRM ion pair were obtained according to further DP and CE optimization; according to the metabolites eluted in each period, a set of specific MRM ion pairs were monitored from this period. The mass spectrum data above was used for qualitative and quantitative analysis on the basis of the MetWare database (MWDB) of MetWare Biotechnology Co., Ltd. (Wuhan, China) to obtain the original metabolite data. For quality control (QC) analysis, one quality control sample was inserted into each of the ten test and analysis samples to monitor the repeatability of the analytic process. Partial least-squares discriminant analysis (OPLS-DA) was employed to screen variation components. The detailed methods also were described in previous studies [73-75]. The differentially expressed metabolites (DEMs) were screened based on the $|\log_2$Fold Change$| \geq 1$ or P-value <0.05, and variable importance in project (VIP) ≥ 1. To study the specific accumulation of metabolites, the principal component analysis (PCA) of significantly changed metabolites was performed by R (www.r-project.org/). The correlations between the differentially expressed genes and the metabolites were performed based on the Pearson correlation with the correlation coefficient (r) value > 0.8 or < -0.8. Correlation networks were used to visualize the relationships between the genes and metabolites using OmicStudio tools (https://www.omicsmart.com/).

To further identify the co-expressed genes and regulators in juglone biosynthesis, transcriptomics and metabolomics were carried out on four stages of walnut exocarp development. From the principal component analysis of walnut exocarp (Figure S10) and embryos (Figure S11), the obvious distinction of metabolites was found from different sample groups, and these metabolites can be used for next step of metabolomics analysis. A total of 470 secondary metabolic products were detected, mainly containing seven types of metabolites, including phenolic acids (147, 31.3%), flavonoids (134, 28.5%), tannins (50, 10.6%), alkaloids (47, 10%), lignans and coumarins (28, 6.0%), terpenoids (24. 5.1%), quinones (21, 4.5%) and others (19, 4.0%) (Figure S12 and Table S16). Juglone content increased during the transition from S2 to

S3 stage, whereas the juglone content was slightly reduced from S3 to S4 (Figure 5a). The juglone profiles during the S1 and S2 stages from walnut exocarp was distinct compared to S3 and S4. Totally, 195 metabolites were differentially accumulated between S1 and S2 vs S3 and S4. We also analyzed the differentially accumulated metabolites among different stages for the Juglone component of walnut exocarp. In addition to S1 vs S2 and S3 vs S4, four of the six pairs contain the differentially accumulated juglone (Table S17), consistent with the results above, which show that the juglone content varies during the development of walnut exocarp in *J. mandshurica*. Furthermore, we focused on the DEGs derived from four (S1 vs S3, S1 vs S4, S2 vs S3 and S2 vs S4) paired groups that differentially accumulated juglone, and 897 common DEGs were found in these groups (Figure S13 and Table S18). In a combined GO and KEGG enrichment analysis of 897 DEGs, we observed that the most enriched terms were "extracellular region", and "integral component of membrane" in the GO database, and "biosynthesis of secondary metabolites", and "metabolic pathways" in the KEGG pathways (Figure S14 and Figure S15).

Transcription factors (TFs) are important regulators of plant growth, development, metabolism and adaptation. We identified some transcription factors (TFs) that may be related to the biosynthesis of juglone during walnut exocarp development. There are 777, 1,082, 154 and 826 differentially expressed TFs (DEG-TFs) in S1 vs S3, S1 vs S4, S2 vs S3 and S2 vs S4, respectively (Figure 5b and Table S19). In which, 62 MYB, 55 AP2/ERF-ERF, 50 NAC, 48 bHLH, 45 C2H2, 36 WRKY and 26 bZip TFs were found in S1 vs S3, and 82 MYB, 73 C2H2, 72 bHLH, 71 AP2/ERF-ERF, 64 NAC and 60 WRKY TFs were identified in S1 vs S4, and these TFs may be involved in juglone biosynthesis. In addition, we observed that the common TFs identified in four groups mainly contains AP2/ERF-ERF, bHLH, bZIP, C2H2, MYB and NAC, which are essential for juglone biosynthesis. Expression analysis also indicated that the majority of TFs showed higher expression in S3 and S4 compared with the S1 and S2, which is consistent with the results of metabolites, suggesting a strong association of these TFs with juglone accumulation during walnut exocarp differentiation.

To obtain genes that displayed similar abundance patterns as juglone content, co-expression cluster analysis was performed using K-means methods based on the FPKM values. All genes were mainly clustered into ten clusters with distinct expression in which cluster 6 (990 genes) showed variation similar to that of the juglone content during walnut exocarp differentiation, suggesting that these genes were important to the understanding of juglone biosynthesis at the transcript level (Figure 5c). GO enrichment analysis showed that 990 DEGs were enriched in "intracellular", "intracellular part" and "RNA metabolic process" (Figure S16). The top enriched KEGG DEGs are "Purine metabolism", "RNA polymerase" and "Spliceosome" (Figure S17).

To more deeply explore the relationship between gene expression and Juglone, data obtained from metabolites (juglone) and genes (including *CYP450s* and MYB, NAC, bZip TFs *etc.*) were employed to construct a gene-to-metabolite correlation network in walnut exocarp. 1,860 DEGs correlated with juglone content, and the Pearson correlation coefficient was set at $r > 0.8$ or < -0.8 as the cutoff (Table S20). The

core conserved DEGs (566) in the four paired groups (S1 vs S3, S1 vs S4, S2 vs S3 and S2 vs S4) were mainly enriched in "biosynthesis of secondary metabolites", "alanine, aspartate and glutamate metabolism" and "nitrogen metabolism" in KEGG terms (Figure S18 and Figure S19). 155 of 1,860 DEGs were identified as transcription factors, and the top fifteen TFs (see the pie chart) consist of MYB, C2H2, AP2/ERF-ERF, bHLH, NAC, and bZip TFs, which indicates obvious TF regulation for juglone during walnut exocarp differentiation (Figure 5b). 5 of the 14 *CYP450* genes that correlated with juglone (r>0.8 or < -0.8) showed a strong positive correlation with the network, and two genes encoding decarboxylases showed the same relationship, suggesting that these genes may be involved in juglone biosynthesis (Figure 5d and Table S21). Among the 155 TFs, all of the bZIP (5), NAC (8), NF-YA (4), NF-YC (3) and FAR1 (3) TFs were strongly correlated with juglone, and the majority of these TFs were highly expressed in the S3 and S4 stages, suggesting regulation functions for juglone (Figure 5e). Thus, these TFs could play crucial roles in the gene-to-juglone regulatory network. The five core *CYP450* genes and several key TFs could be good candidate genes, providing new insights into natural juglone biosynthesis.

Gene discovery for lipid biosynthesis and oil body formation

J. mandshurica is an important oil plant species, and its oil-rich (more than 50%) embryos have a variety of different oils such as oleic acid, linoleic acid, unsaturated fattyacids etc. The oil has important medicinal, nutritional and industrial value. Although, previous studies have found multiple oil components and confirmed their activity as antiviral, antimicrobial, anticancer or antihelminth agents [76, 77], the molecular regulatory mechanisms for lipid biosynthesis and oil body formation in *J. mandshurica* are still poorly understood [78-80]. The formation of oils in plant seeds occurs through the lipid biosynthesis pathway, where the fatty acids are synthesized in the plastids, and the synthesis of triacylglycerides (TAGs) takes place in the endoplasmic reticulum. TAGs are stored in an oil body after synthesis and degraded to provide carbon and energy during seed germination and early seedling growth [81].

In this work, we investigated the regulation of genes involved in the lipid biosynthesis and oil body formation, using a combination of transcriptome sequencing and metabolomics, on four different stages of differentiation of walnut embryos in *J. mandshurica*. We identified 450 metabolites in *J. mandshurica* samples including 99 lipids (22%), 91 amino acids and their derivatives (20.2%), 77 organic acids (17.1%), 61 phenolic acids (13.6%), 51 nucleotides and their derivatives (11.33%) and 71 other compounds (15.78%) (Figure S20 and Table S22). Among these metabolites, lipids were predominant, mainly consisting of 41 free fatty acids (41.4%), 28 lysophatidylcholine (LPC) (28.3%), 14 lysophatidylethanolamines (LPE) (14.1%), 13 glycerol esters (13.1%), 2 sphingolipids (2.02%) and 1 phosphatidylcholine (PC) (1.01%) (Figure S21 and Table S23), indicating that the walnut embryos are rich in free fatty acids during differentiation. 11 of the 41 free fatty acids had the highest content from S1 to S4 (Figure S22) such as linoleic acid (C18:3), stearic acid (C18:0), arachidic acid (20:0), α-linolenic acid and γ-linolenic acid, indicating abundant oils in walnut embryoss of *J. mandshurica*. The top enriched KEGG terms of these compared groups

mainly were “metabolic pathway”, “biosynthesis of secondary metabolites”, “biosynthesis of amino acids” and “ABC transporters” (Figure S23). These oils were enriched during walnut differentiation, providing key information on lipid biosynthesis in *J. manshurica.*

A transcriptome survey was carried out to gain greater insight into lipid biosynthesis and oil body formation. We identified 346 genes related to lipid synthesis, including 105 for fatty acid (FA) biosynthesis, 202 for triacylglycerol (TAG) biosynthesis and 39 for oil body formation (Figure 6). The expression levels of the majority of structural genes (e.g. PDH and ACC(BC)) at the S2 stage were higher than the expression at other stages, whereas the majority of the other genes were significantly expressed in the S4 stage. This may partially explain why the content of the free fatty acids is maintained at a high level in the S2 stage (Figure S24). After their formation, the free fatty acids are activated by long-chain acyl-CoA synthetases (LACS) to generate acyl coenzyme A (Acyl-CoA) derivatives, which are transported out of the plastid. The transcript level of LACS at S4 was higher than in other stages, which contributes to the synthesis of triacylglycerol (TAG). The TAGs are synthesized and oil bodies are formed in the endoplasmic reticulum [82]. Glycerol- 3- phosphate acyltransferase (GPAT) catalyzes the glycerol-3-phosphate and acyl coenzyme A to form lysophosphatidic acid (LPA), and then LPA was converted to phosphatidic acid (PA) by lysophosphatidic acid acyltransferase (LPAT). Then phosphatidic acid phosphatase (PAP) converts PA to diacylglycerol (DAG). TAGs are finally synthesized with the reaction of DAG by diacylglycerol acyltransferase (DGAT) or diacylglycerol acyltransferase (PDAT) [83]. The majority of differentially expressed PAP genes were expressed at high levels in S4, indicating a dominant role for TAG formation. The expression of DGAT genes was similar to PAP genes. To form oil bodies, TAGs are bound to several proteins, including oleosion, caleosin and steroleosin (STERO) [84]. Oleosin is an oil protein that could increase charge repulsion, allowing oil bodies to be independent of each other. Oleosin maintains high expression in S2, S3 and S4 in the walnut embryos, which suggests that the genes encoding these proteins are good candidates for genes regulating oil body formation.

Several transcription factors (TFs) regulate plant growth and development. 1,876 of 15,152 DEGs were identified in *J. mandshurica*, representing the 12.38% of the DEGs during the differentiation of the walnut embryos (Table S24). The top five transcription factor families are 110 MYBs, 109 C2H2s, 105 AP2/ERF-ERFs, 102 bHLHs and 76 NACs, many of which may be related to walnut embryos development and differentiation. Some TFs (i.e., ABI3, LEC1, LEC2, FUS3 and WRI1) are considered to be key regulators of lipid biosynthesis and oil accumulation in many plant species. For the transcriptome analysis, 1FUS3, 2 ABI3s, 2 LEC2s and 4 WRI1s were identified, whereas LEC1 TFs were not detected, suggesting that the lipid synthesis was not regulated by these transcription factors in mature walnut embryos (S4) (Table S25). ABI3 is involved in seed development and dormancy, and particularly in fatty acid metabolism [85, 86]. Four ABI3 TFs showed high expression in the S4 stage consistent with the oil accumulation pattern, suggesting a role in regulation of lipid biosynthesis in mature walnut embryos. WRIl and FUS3 are expressed at a high level during the S2

and S3 stages, suggesting a minor role in lipid biosynthesis and oil accumulation. To further investigate the correlation between the identified genes and lipid biosynthesis, an analysis was performed using Pearson's correlation. One ABI3 transcription factor was highly correlated with the eicosadienoic acid (C20:2) (r=-0.839). We also found one ABI3 gene negatively correlated with several glycerides (r=-0.828), lysophatidylethanolamine (r=-0.844) and lysophatidylcholine (r=-0.807), which suggests that an ABI3 interacting protein is an inhibitor or plays a different negative role in lipid biosynthesis.

Discussion

Third-generation sequencing is a powerful technology, which will accelerate genetic improvement for many crop species [87]. Whole-genome sequencing has been carried out for many plants, providing useful genomic information for functional gene mining, genetic linkage map construction, quantitative trait analysis and molecular breeding [88, 89]. Manchurian walnut, is widely distributed in northeast China and utilized for its edible kernals, elite wood characteristics and the medicinal value of its secondary products. Research on this species has been limited, and genome-level studies are lacking. This study provides a high-quality and chromosome-level reference genome sequence for molecular breeding and for evolutionary studies in the Juglandaceae. Using long (PacBio) HiFi reads, Next Generation Sequencing (NGS) and Hi-C scaffolding, we sequenced and assembled the genome of *J. mandshurica*, providing new insights and valuable genetic information on juglone and lipid biosynthesis.

Here, we report the highest quality genome assembly for *J. mandshurica* to date, with the longest contig N50 of 21 Mb and the highest genome completeness of 98.3% in terms of BUSCO results. Compared with previous studies on genome assemblies for this species, our contig N50 size (21Mb) for *J. mandshurica* was improved by 187-fold (N50 was 0.1) [90] and 3-fold (N50 was 6 Mb) [91]. Our complete BUSCO score was 1,592, higher than a recent genome assembly of 1,375) [91]. After sequencing and assembly, our assembled genome was 548.7 Mb, slightly higher than that of Yan et al., (548.5 Mb) [91], but smaller than that of Stevens et al., (580 Mb) [90]. We obtained a total of 40,453 protein-coding genes, which is substantially higher than that of Yan et al., with (29,032 protein-coding genes) [91]. Through the sequence platform of WGS-PacBio Sequel II, we obtained 14.62 Gb of genome sequence, whereas the previous genome sequence was 10.1 Gb based on the Nanopore sequencing platform. We found that the GC content and repeated sequences were 36.72% and 0.33 Gb (60.99%), a relatively lower level of repeats compared with that of Yan et al., where the recent genome assembly was 38.5% GC content and the repeat sequences were 0.34 Gb (62.08%). Our chromosome-scale genome should serve as the best available reference genome for *J. mandshurica*, providing valuable information for further analysis of evolution and mining of functional genes.

To date, nine Juglandaceae species have been sequenced for whole-genome information, yielding a mass of high throughput sequence data and contributing to understanding the evolution of Juglandaceae species [92]. Based on 558 single-copy

orthologs from 15 plant genomes (9 of 15 was Juglandaceae family species), genome level studies were carried out to update the evolutionary relationships of *J. mandshurica* and its related species. The Juglandaceae species maintain close genomic relationships [92]. In our study, all of the Juglandaceae species group into the same cluster, consistent with the morphology-based plant taxonomy. The phylogenomic analysis showed that the *J. mandshurica* was closest to *J. cathayensis* (divergence at ~13.8 mya). Genome-wide duplications (WGD) offer the evolutionary potential to generate new functions in plant species [57]. For comparative genomics, there was a Juglandoid WGD that originated prior to the radiation of Juglandaceae in the Paleocene, and similar results were also found in previous study [93]. In the collinearity comparison with *P. trichocarpa*, many syntenic blocks were found, indicating a close genetic relationship between *J. mandshurica* and *J. regia*, and a more distant relationship with *P. trichocarpa*.

The Juglandaceae family is an economically important species which has been planted and domesticated since the Han dynasty (206 BC to 220 AD). The fruit of many species in this family is rich in medicinal components in the walnut exocarp and and the oils in the kernel, especially for species in the *Carya* and *Juglans* genera [94, 95]. The regulatory mechanism of fruit development/differentiation and the biosynthesis of juglone and lipids in *J. manshurica* remain largely unknown. Here, for walnut exocarp differentiation, we combined genomic, transcriptomic, and metabolomic data to construct a gene-metabolite network, identifying genes and TFs that may encode decarboxylases, 2-ODDs and CYP450s involved in juglone biosynthesis. In previous work, a small number of genes were identified affecting the bioactivity of juglone in walnut roots and leaves [14, 15]. Here,125 CYP family genes were found that showed high expression during the differentiation of walnut exocarp, indicating a key role of the CYP family. This result is similar to the secondary metabolite biosynthesis in plants such as *Aconitum vilmorinianum* (aconite) [96], *Salvia miltiorrhiza* (red sage) [97], *Scutellaria baicalensis* (Chinese skullcap) [98] and *Aralia elata* (angelica tree) [99]. We identified the expanded *CYP450* gene family from the genome assembly of the *J. mandshurica*, suggesting that they may specifically affect the biosynthesis of juglone. 5 of 14 *CYP450* genes were correlated with juglone ($r > 0.8$ or < -0.8), and are candidate genes for further study on juglone biosynthesis. Although some genes in the juglone biosynthetic pathway have been identified, the regulators remain unknown. We have also identified potential TFs (e.g. bZip, NAC, NF-YA and NF-YC TFs) that are positively correlated with the abundance of juglone and may participate in juglone biosynthesis. These results may be valuable for further studies on juglone biosynthesis and its agricultural uses.

In addition to the study of the walnut exocarp, we used the developing/differentiating kernel to identify genes involved in oil accumulation in *J. mandshurica*. Lipids were the dominant secondary metabolites. Free fatty acids were enriched in embryos of *J. mandshurica.* Some free fatty acids (e.g. linoleic acid, stearic acid, arachidic acid, α-linolenic acid and γ-linolenic acid) were the most abundant during the S1 to S4 stages. The corresponding genes (e.g. PAP, LACS and DGAT) involved in lipid biosynthesis also show high abundance during embryo development,

suggesting that these genes and metabolites play key roles in lipid synthesis for oil accumulation. We also found some transcription factors (e.g. MYB, C2H2, AP2/ERF-ERF, and NAC) that are differentially expressed during embryo development. Such regulation of TFs implies that they may play important roles in the biosynthesis of lipids in *J. mandshurica*, similar to results found in previous studies [100, 101]. Some TFs known to take part in lipid biosynthesis, were identified. These TFs were correlated with lipid metabolites, similar to a previous study [102-104]. These results lay the foundation for identification of genes encoding enzymes that catalyze the formation of fatty acids and oil bodies, and will be valuable in the future for studies or engineering of lipid biosynthesis.

Data Availability

Raw reads used for genome assembly of *J. mandshurica* has been uploaded to the National Center for Biotechnology Information (NCBI) Sequences Read Archive (SRA) with the accession numbers: SRR14637189 and SRR14629954. Transcriptomic data have been deposited in SRA with the accession number of PRJNA733587 (including embryos and exocarp). The assembled *J. mandshurica* genome has been deposited in the Genome Warehouse in National Genomics Data Center (NGDC) (https://ngdc.cncb.ac.cn/) under accession number PRJCA006358. Third generation transcriptomic data have been deposited in BIG Data Center under accession number PRJCA006794. All supporting data and materials are available in the GigaScience GigaDB database. The metabolomics data in this study were deposited and available at Metabolights repository (www.ebi.ac.uk/metabolights/MTBLS3657) under the accession numbers MTBLS3657.

Additional files

Supplementary Figure 1. Changes of *J. mandshurica* fruits in different development periods. S1-S4 indicate the fruit collected at 30 days (S1 stage), 50 days (S2 stage), 70 days (S3 stage) and 90 days (S4 stage) after natural pollination.

Supplementary Figure 2. The tissue structure of *J. mandshurica* fruit including the walnut exocarp and walnut embryos.

Supplementary Figure 3. Photographs of *J. mandshurica*. (a) male flower (b) female flower (c) mature fruit (d) adult tree

Supplementary Figure 4. 17-mer analysis to estimate the *J. mandshurica* genome size. The x-axis and y-axis indicate the 17-mer number and frequency of kmer, respectively

Supplementary Figure 5. Sequencing depth distribution of the assembled *J. manshurica* genome

Supplementary Figure 6. Cross-species comparisons of exon number, intron number, gene length, gene GC, CDS GC, exon length, CDS length and intron length distribution

Supplementary Figure 7. Upset plot of genes annotated in GO, InterPro, KEGG, NR, Swissprot and TrEMBL database.

Supplementary Figure 8. The GO category analysis of rapidly expanded gene families in assembly *J. mandshurica* genome

Supplementary Figure 9. Schematic representation of syntenic genes among *J.*

mandshurica and *J. regia*

Supplementary Figure 10. PCA score plot metabolite profiles from different sample groups during developmental walnut exocarp

Supplementary Figure 11. PCA score plot metabolite profiles from different sample groups during developmental walnut embryos

Supplementary Figure 12. Distribution of identified metabolites in green peel in *J. mandshurica*

Supplementary Figure 12. Venn diagrams of differentially expression genes (DEGs) in S1, S2, S3 and S4 stages in green peel in *J. mandshurica*

Supplementary Figure 14. GO enrichment analysis of 897 core DEGs in *J. mandshurica*

Supplementary Figure 15. KEGG enrichment analysis of 897 core DEGs in *J. mandshurica*

Supplementary Figure 16. GO enrichment analysis of 990 DEGs identified in cluster 6

Supplementary Figure 17. KEGG enrichment analysis of 990 DEGs identified in cluster 6

Supplementary Figure 18. Upset plot of differentially expression genes associated with Juglone ($r > 0.8$ or < -0.8) in S1, S2, S3 and S4 stage in *J. mandshurica*

Supplementary Figure 19. KEGG enrichment analysis of 566 core DEGs associated with Juglone ($r > 0.8$ or < -0.8) in S1, S2, S3 and S4 stage in *J. mandshurica*

Supplementary Figure 20. Distribution of identified metabolites in walnut kernels in *J. mandshurica*

Supplementary Figure 21. Distribution of identified lipid components in walnut kernel in *J. mandshurica*

Supplementary Figure 22. Heatmap of the free fatty acids during walnut kernel development in *J. mandshurica*

Supplementary Figure 23. KEGG enrichment analysis of different metabolites in six comparison groups. (a) S1 vs S2 (b) S1 vs S3 (c) S1 vs S4 (d) S2 vs S3 (e) S2 vs S4 (f) S3 vs S4

Supplementary Figure 24. Heatmap of the free fatty acids during walnut kernel development in *J. mandshurica*

Supplementary Table 1. The statistics of K-mer analysis

Supplementary Table 2. BUSCO evaluation results for *J. mandshurica* genome

Supplementary Table 3. The information of transcriptome of walnut exocarp and embryos

Supplementary Table 4. Statistical results of genetic structure of related species

Supplementary Table 5. Summary of the functional annotation in *J. mandshurica* genome

Supplementary Table 6. Non-coding genes in the *J. mandshurica* genome

Supplementary Table 7. Statistics of transposable elements and other repeats in *J. mandshurica* genome

Supplementary Table 8. Repeat sequence classification results statistics in *J. mandshurica* genome

Supplementary Table 9. Repeat elements in *J. mandshurica* genome

Supplementary Table 10. Comparison of the estimated number of gene families of *J. mandshurica* with other plants

Supplementary Table 11. Summary of gene ortholog analysis conducted on 16 sequenced genomes

Supplementary Table 12. The KEGG enrichment of expanded gene family

Supplementary Table 13. The KEGG enrichment of contracted gene family

Supplementary Table 14. Genes encoding enzymes related to juglone biosynthesis

Supplementary Table 15. The genes of rapidly expanded CYP450 gene family in *J. mandshurica* and its related species

Supplementary Table 16. The identified metabolites isolated from walnut exocarp of *J. mandshurica*

Supplementary Table 17. The differentially accumulated metabolites (DAMs) during different developmental stage of walnut exocarp

Supplementary Table 18. The differentially expressed genes during different developmental stage of walnut exocarp

Supplementary Table 19. The number of transcription factors in different compare groups

Supplementary Table 20. The differentially expressed genes correlated with the Juglone ($r > 0.80$ or < -0.8)

Supplementary Table 21. The gene-to-metabolite correlation coefficient

Supplementary Table 22. The identified metabolites isolated from walnut embryo of *J. mandshurica*

Supplementary Table 23. The identified metabolites involved in lipid in embryo of *J. mandshurica*

Supplementary Table 24. The transcription factors identified during the developmental embryo

Supplementary Table 25. The transcription factors involve in the lipid synthesis and oil accumulation

Abbreviations

BLAST: Basic Local Alignment Search Tool; bp: base pair; BUSCO: Benchmarking Universal Single-Copy Orthologs; DEGs: differentially expressed genes; DAMs: differentially accumulated metabolites; GATK: Genome Analysis Tool Kit; Gb: gigabase pairs; GO: Gene Ontology; HiFi: high fidelity; kb: kilobase pairs;KEGG: Kyoto Encyclopedia of Genes and Genomes; Ks: synonymous substitutions per synonymous; Mb: megabase pairs; mRNA: messenger RNA; mya: million years ago; NCBI: National Center for Biotechnology Information; NGDC: National Genomics Data Center; PacBio: Pacific Biosciences; TFs: transcription factors; TEs: transposable elements; VIP: variable importance in project; WGD: whole-genome duplication.

Competing interests

The authors declare no competing interests.

Funding

This research study was supported by the Innovation Project of State Key Laboratory of Tree Genetics and Breeding (Northeast Forestry University) (No. 2021A01), the Fundamental Research Funds for the Central Universities (Northeast Forestry University) (No. 2572020DR01) and Heilongjiang Touyan Innovation Team Program (Tree Genetics and Breeding Innovation Team).

Authors’ contributions

X.L, K.W.C, Q.H.Z and X.N.P was a major contributor in writing the manuscript; Z.M.H, S.C.1, L.P.J, M.H.Z and Y.L contributed to plant sample collection, DNA/RNA preparation, library construction and sequencing; S.K.Z, X.X.Z, Y.X.L and S.C. worked on genome assembly and annotation, V.C and R.S conducted transcriptome analysis and identified functional genes involved in juglone biosynthesis. G.Z.Q and M.T analyzed the gene family and constructed the evolutionary tree. XYZ conceived of the study, participated in its design and data interpretation, and revised the manuscript critically.

References

1. Bai WN and Zhang L. Nuclear and chloroplast DNA phylogeography reveal two refuge areas with asymmetrical gene flow in a temperate walnut tree from East Asia. New Phytologist 2010;**188**(3):892-901.
2. Zhang L, Guo C, Lu X, Sun X and Deng J. Flower Development of Heterodichogamous *Juglans mandshurica* (Juglandaceae). Frontiers in Plant Science 2021;**12**:541163.
3. Luan F, Wang Z, Yang Y, Ji Y and Zeng N. Juglans mandshurica Maxim.: A Review of Its Traditional Usages, Phytochemical Constituents, and Pharmacological Properties. Frontiers in Pharmacology 2021;**11**:569800.
4. Zhang Y-Y, Zhang F, Zhang Y-S, Thakur K, Zhang J-G, Liu Y, et al. Mechanism of Juglone-Induced Cell Cycle Arrest and Apoptosis in Ishikawa Human Endometrial Cancer Cells. Journal of agricultural and food chemistry 2019;**67**(26):7378-89.
5. Prasad CV, Shrivastava S, Gm VMN and Mallavadhani UV. Synthesis and anticancer activity of some novel 5,6-fused hybrids of juglone based 1,4-naphthoquinones. European Journal of Medicinal Chemistry: Chimie Therapeutique 2014;**83**:84-91.
6. Umarov AU, Burnasheva SN and Makhmudova KS. Oil from the seeds of Juglans mandshurica and Anabasis Aphylla. Chemistry of Natural Compounds 1970;**6**(2):258-9.
7. Yu N, Lin L and Wang F. Extraction of manchurian walnut oil assisted by ultrasonic and fatty acid composition. Food Science and Technology 2014;**39**(10):184-8.
8. Yang H, Gan C, Guo Y, Qu L and Wang J. Two novel compounds from green walnut husks (Juglans mandshurica Maxim.). Natural Product Research 2020;**2**:1-9.
9. Islam A and Widhalm JR. Agricultural Uses of Juglone: Opportunities and Challenges. Agronomy 2020;**10**:1500.
10. Girzu M, Carnat A, Privat AM, Fialip J and Lamaison AP. Sedative Effect of Walnut Leaf Extract and Juglone, an Isolated Constituent. Pharmaceutical Biology 1998;**36**(4):280-6.
11. Silva-Belmares SY, Saenz-Galindo A, Garcia JJV and Lopez-Lopez LI. Ultrasonic and

Microwave Assisted Synthesis of Nitrogen-Containing Derivatives of Juglone as Potential Antibacterial Agents. Letters in Organic Chemistry 2014;**11**(8):573-82.

12. Shen CC, Afraj SN, Hung CC, Barve BD and Kuo YH. Synthesis, biological evaluation, and correlation of cytotoxicity versus redox potential of 1,4-naphthoquinone derivatives. Bioorganic & Medicinal Chemistry Letters 2021;**17**:127976.
13. Shi L, Ma X, A L, Chen G and Wei H. Research Progress of Qinglongyi(cortex juglandis mandshuricae). Guiding Journal of Traditional Chinese Medicine and Pharmacy 2020;**26**(05):122-5.
14. Widhalm JR and Rhodes D. Biosynthesis and molecular actions of specialized 1,4-naphthoquinone natural products produced by horticultural plants. Horticulture Research 2016;**3**:16046.
15. Mccoy RM, Utturkar SM, Crook JW, Thimmapuram J and Widhalm J. The origin and biosynthesis of the naphthalenoid moiety of juglone in black walnut. HORTICULTURE RESEARCH 2018;**5**(1):67.
16. Porebski S, Bailey LG and Baum BR. Modification of a CTAB DNA extraction protocol for plants containing high polysaccharide and polyphenol components. Plant Molecular Biology Reporter 1997;**15**(1):8-15.
17. Cheng H, Concepcion GT, Feng X, Zhang H and Li H. Haplotype-resolved de novo assembly using phased assembly graphs with hifiasm. Nature Methods 2021;**18**(2):1-6.
18. Xi Y, Di L, Fei L, Wu J and Zhu B. HTQC: A fast quality control toolkit for Illumina sequencing data. BMC Bioinformatics 2013;**14**(1):33.
19. Li H. Aligning sequence reads, clone sequences and assembly contigs with BWA-MEM. Genomics 2013;**1303**:3097.
20. Dudchenko O, Batra SS, Omer AD, Nyquist SK, Hoeger M, Durand NC, et al. De novo assembly of the Aedes aegypti genome using Hi-C yields chromosome-length scaffolds. Science 2017;**356**(6333):92.
21. Li H. Minimap2: fast pairwise alignment for long nucleotide sequences. Bioinformatics 2018;**34**(18):3094-100.
22. Waterhouse RM, Fredrik T, Li J, Zdobnov EM and Kriventseva EV. OrthoDB: a hierarchical catalog of animal, fungal and bacterial orthologs. Nucleic Acids Research 2013;**41**:D358-D65.
23. Mosè M, Matthew RB, Mathieu S, Felipe AS, Evgeny MZ. BUSCO Update: Novel and Streamlined Workflows along with Broader and Deeper Phylogenetic Coverage for Scoring of Eukaryotic, Prokaryotic, and Viral Genomes. of Eukaryotic, Prokaryotic, and Viral Genomes, Molecular Biology and Evolution 2021; **38**(10): 4647–4654.
24. Chen N. Using RepeatMasker to identify repetitive elements in genomic sequences. Current protocols in bioinformatics 2004;**25**:4-10.
25. Jurka J, Kapitonov VV, Pavlicek A, Klonowski P, Kohany O and Walichiewicz J. Repbase Update, a database of eukaryotic repetitive elements. Cytogenetic & Genome Research 2005;**110**(1-4):462-7.
26. György A, Norbert G, Luc DM and Wojciech M. TEclass--a tool for automated classification of unknown eukaryotic transposable elements. Bioinformatics 2009;**25**(10):1329-30.
27. Xu Z and Wang H. LTR_FINDER: an efficient tool for the prediction of full-length LTR retrotransposons. Nucleic acids research 2007;**35**:W265-W8.
28. Benson G. Tandem repeats finder: a program to analyze DNA sequences. Nucleic acids research

1999;**27**(2):573-80.

29. Schffer AA, Richa A, Yu YK, Michael GE and Altschul SF. Composition-based statistics and translated nucleotide searches: Improving the TBLASTN module of BLAST. BMC Biology 2006;**4**:41.
30. Slater G and Birney E. Automated generation of heuristics for biological sequence comparison. Bmc Bioinformatics 2005;**6**:31.
31. Mario S, Oliver K, Irfan G, Alec H, Stephan W and Burkhard M. AUGUSTUS: ab initio prediction of alternative transcripts. Nucleic Acids Research 2006;**34**:W435-W9.
32. Majoros W, Pertea M and Salzberg S. TigrScan and GlimmerHMM: two open source ab initio eukaryotic gene-finders. Bioinformatics 2004;**20**(16):2878-9.
33. Thomas D and Watanabe C, K. GMAP: a genomic mapping and alignment program for mRNA and EST sequences. Bioinformatics 2005;**21**(9):1859-75.
34. Camacho C, Coulouris G, Avagyan V, Ning M, Papadopoulos J, Bealer K, et al. BLAST+: architecture and applications. Bmc Bioinformatics 2009;**10**(1):421.
35. Campbell MS, Holt C, Moore B and Yandell M. Genome annotation and curation using MAKER and MAKER-P. Current protocols in bioinformatics 2014;**48**:4.11.1-4..39.
36. Yang FS, Nie S, Liu H, Shi TL and Mao JF. Chromosome-level genome assembly of a parent species of widely cultivated azaleas. Nature Communications 2020;**11**:5269.
37. Fu A, Wang Q, Mu J, Ma L and Zuo J. Combined genomic, transcriptomic, and metabolomic analyses provide insights into chayote (Sechium edule) evolution and fruit development. Horticulture research 2021;**8**:35.
38. Lowe TM and Eddy SR. tRNAscan-SE: a program for improved detection of transfer RNA genes in genomic sequence. Nucleic Acids Research 1997;**25**(5):955-64.
39. Nawrocki EP, Kolbe DL and Eddy SR. Infernal 1.0: inference of RNA alignments. Bioinformatics 2009;**25**(10):1335-7.
40. Ma Q, Sun T, Li S, Wen J, Zhu L, Yin T, et al. The Acer truncatum genome provides insights into the nervonic acid biosynthesis. The Plant Journal 2020;**104**(3):662-78.
41. Li Y, Wei H, Yang J, Kang D and Kang X. High-quality de novo assembly of the Eucommia ulmoides haploid genome provides new insights into evolution and rubber biosynthesis. Horticulture Research 2020;**7**(1):183.
42. Tu L, Su P, Zhang Z, Gao L and Gao W. Genome of Tripterygium wilfordii and identification of cytochrome P450 involved in triptolide biosynthesis. Nature Communications 2020;**11**(1):971.
43. Chen S, Wang Y, Yu L, Zheng T, Wang S, Yue Z, et al. Genome sequence and evolution of Betula platyphylla. Horticulture Research 2021;**8**(1):37.
44. L. L, Stoeckert CJ and Roos DS. OrthoMCL: Identification of Ortholog Groups for Eukaryotic Genomes. Genome Research 2003;**13**(9):2178-89.
45. Stevens KA, Woeste K, Chakraborty S, Crepeau MW and Langley CH. Genomic Variation Among and Within Six Juglans Species. G3-Genes Genomes Genetics 2018;**8**(7):g3.200030.2018.
46. Huang Y, Xiao L, Zhang Z, Zhang R, Wang Z, Huang C, et al. The genomes of pecan and Chinese hickory provide insights into Carya evolution and nut nutrition. GigaScience 2019;**8**(5):giz036.
47. Sork VL, Fitz-Gibbon ST, Puiu D, Crepeau M and Salzberg SL. First Draft Assembly and

Annotation of the Genome of a California Endemic Oak Quercus lobata Née (Fagaceae). G3 (Bethesda, Md) 2016;**6**(11):3485-95.

48. Daniell H. Complete Plastid Genome Sequences of Three Rosids (Castanea, Prunus, Theobroma): Evidence for At Least Two Independent Transfersn of rpl22 to the Nucleus. Molecular Biology & Evolution 2011;**28**(1):835-847.
49. Jia HM, Jia HJ, Cai QL, Wang Y, Zhao HB, Yang WF, et al. The red bayberry genome and genetic basis of sex determination. Plant Biotechnology Journal 2019;**17**(2):397-409.
50. Tuskan G, Difazio S, Jansson S, Bohlmann J, Grigoriev I, Hellsten U, et al. The Genome of Black Cottonwood, Populus trichocarpa (Torr. & Gray). Science 2006;**313**(5793):1596-604.
51. Jaillon O, Aury JM, Noel B, Policriti A, Clepet C, Casagrande A, et al. The grapevine genome sequence suggests ancestral hexaploidization in major angiosperm phyla. Nature 2007;**449**(7161):463.
52. Kawahara Y, Bastide M, Hamilton JP, Kanamori H, McCombie WR, Ouyang S, et al. Improvement of the Oryza sativa Nipponbare reference genome using next generation sequence and optical map data. Rice (New York, NY). 2013;**6**(1):4.
53. Edgar RC. MUSCLE: multiple sequence alignment with high accuracy and high throughput. Nucleic acids research 2004;**32**:1792-1797.
54. Alexandros S. RAxML version 8: a tool for phylogenetic analysis and post-analysis of large phylogenies. Bioinformatics 2014;**30**(9):1312-3.
55. Sanderson MJ. r8s: Inferring absolute rates of molecular evolution and divergence times in the absence of a molecular clock. Bioinformatics 2003;**19**(2):301-2.
56. Yang Z. PAML 4: Phylogenetic Analysis by Maximum Likelihood. Molecular Biology and Evolution 2007;**24**(8):1586-91.
57. Zhang J, Zhang W, Ji F, Qiu J, Song X, Bu D, et al. A high-quality walnut genome assembly reveals extensive gene expression divergences after whole-genome duplication. Plant biotechnology journal 2020;**18**:1848-1850.
58. Tang H, Bowers JE, Wang X, Ming R, Alam M and Paterson AH. Synteny and Collinearity in Plant Genomes. Science 2008;**320**(5875):486-8.
59. Chen C, Chen H, Zhang Y, Thomas HR and Xia R. TBtools: An Integrative Toolkit Developed for Interactive Analyses of Big Biological Data. Molecular Plant 2020;**13**(8):1194-1202.
60. Delcher AL, Salzberg SL and Phillippy AM. Using MUMmer to Identify Similar Regions in Large Sequence Sets. Current Protocols in Bioinformatics 2003;**1**:10.3. 1-.3. 8.
61. Wang P, Luo Y, Huang J, Gao S, Zhu G, Dang Z, et al. The genome evolution and domestication of tropical fruit mango. Genome Biology 2020;**21**:60.
62. Manchester SR and Garden MB. Fossil History of the Juglandaceae. Annals of the Missouri Botanical Garden 1987;**21**:1-137.
63. Manchester SR. Early history of theJuglandaceae. Plant Systematics & Evolution 1989;**162**(1):231-50.
64. Zhang JB, Li RQ, Xiang XG, Manchester SR, Li L, Wang W, et al. Integrated Fossil and Molecular Data Reveal the Biogeographic Diversification of the Eastern Asian-Eastern North American Disjunct Hickory Genus (Carya Nutt.). Plos One 2013;**8**(7):e70449.
65. Zhu TT, Wang L, You FM, Rodriguez JC, Deal KR, Chen LM, et al. Sequencing a Juglans regia × J. microcarpa hybrid yields high-quality genome assemblies of parental species. Horticulture

Research 2019;**6**:55.

66. Kumar S, Stecher G and Tamura K. MEGA7: Molecular evolutionary genetics analysis version 7.0 for bigger datasets. Molecular Biology and Evolution 2015;**33**:1870-4.

67. Kim D, Paggi JM, Park C, Bennett C and Salzberg SL. Graph-based genome alignment and genotyping with HISAT2 and HISAT-genotype. Nature Biotechnology 2019;**37**(8):1.

68. Liao Y, Smyth GK and Shi W. featureCounts: An efficient general-purpose program for assigning sequence reads to genomic features. Bioinformatics 2013;**30**:923–30.

69. Li B and Dewey CN. RSEM: accurate transcript quantification from RNA-Seq data with or without a reference genome. BMC Bioinform 12:323. BMC Bioinformatics 2011;**12**(1):93-9.

70. Love MI, Huber W and Anders S. Moderated estimation of fold change and dispersion for RNA-seq data with DESeq2. Genome Biology 2014;**15**(12):550.

71. Zheng Y, Jiao C, Sun H, G.Rosli H, A.Pombo M, Zhang P, et al. iTAK: A Program for Genome-wide Prediction and Classification of Plant Transcription Factors, Transcriptional Regulators, and Protein Kinases. Molecular Plant 2016;**9**:1667-1670.

72. Tian F, Yang DC, Meng YQ, Jin J and Gao G. PlantRegMap: charting functional regulatory maps in plants. Nucleic Acids Research 2020;**48**:D1104-D13.

73. Dong T, Han R, Yu J, Zhu M, Zhang Y, Gong Y, et al. Anthocyanins accumulation and molecular analysis of correlated genes by metabolome and transcriptome in green and purple asparaguses (Asparagus officinalis , L.). Food Chemistry 2018;**271**(15):18-28.

74. Yuan H, Zeng X, Shi J, Xu Q, Wang Y, Jabu D, et al. Time-Course Comparative Metabolite Profiling under Osmotic Stress in Tolerant and Sensitive Tibetan Hulless Barley. Biomed Research International 2018;**2018**:1-12.

75. Sheng Z and Huang ZX. Fractal Analysis of the Relation between the Observation Scale and the Prediction Cycle in Short-Term Traffic Flow Prediction. International Journal of Intelligent Transportation Systems Research 2018;**17**(1):1-8.

76. Li Jn and Gao Rx. Research Progress on the Genetic Breeding of Juglans mandshurica in China. J Anhui Agric Sci 2020;**48**(17):4-7.

77. Zhao Z. Analysis and Evaluation on Fatty Acid Composition of Hickory Nut Oil. J Anhui Agric Sci 2009;**37**(06):2473-4.

78. Ding M, Lou H, Chen W, Zhou Y, Zhang Z, Xiao M, et al. Comparative transcriptome analysis of the genes involved in lipid biosynthesis pathway and regulation of oil body formation in Torreya grandis kernels Industrial Crops and Products 2020;**145**:112051.

79. Zhang L, Liu M, Long H, Dong W and Tan X. Tung Tree (Vernicia fordii) Genome Provides A Resource for Understanding Genome Evolution and Improved Oil Production. Genomics Proteomics & Bioinformatics 2020;**17**(6):558-75.

80. Rao G, Zhang J, Liu X, Lin C and Wang C. De novo assembly of a new Olea europaea genome accession using nanopore sequencing. Horticulture Research 2021;**8**:64.

81. Huang R, Zhou Y, Zhang J, Ji F and Pei D. Transcriptome Analysis of Walnut (Juglans regia L.) Embryos Reveals Key Developmental Stages and Genes Involved in Lipid Biosynthesis and Polyunsaturated Fatty Acid Metabolism. Journal of Agricultural and Food Chemistry 2020;**69**(1): 377–96.

82. Wang X, Liang H, Guo D, Guo L, Duan X, Jia Q, et al. Integrated analysis of transcriptomic and proteomic data from tree peony (P. ostii) seeds reveals key developmental stages and

candidate genes related to oil biosynthesis and fatty acid metabolism. Horticulture research 2019;**6**:111.

83. Li N, Meng H, Li S, Zhang Z and Luo K. Two novel plastid fatty acid exporters contribute to seed oil accumulation in Arabidopsis. Plant Physiology 2020;**182**(4):1910-9.

84. Gong W, Song Q, Ji K, Gong S, Wang L, Chen L, et al. Full-Length Transcriptome from Camellia oleifera Seed Provides Insight into the Transcript Variants Involved in Oil Biosynthesis. Journal of agricultural and food chemistry 2020;**68**(49):14670–14683.

85. Elhai N, Duncan RW and Stasolla C. Molecular regulation of seed oil accumulation. Journal of Advanced Nutrition and Human Metabolism 2016;**2**:e1296.

86. Wang J, K SS, Du C, Li C, Fan J, Sitakanta P, et al. Comparative Transcriptomic Analysis of Two Brassica napus Near-Isogenic Lines Reveals a Network of Genes That Influences Seed Oil Accumulation. Frontiers in plant science 2016;**7**:1498.

87. Neale DB, Martínez-García P, De L, Montanari S and Wei XX. Novel Insights into Tree Biology and Genome Evolution as Revealed Through Genomics. Annual Review of Plant Biology 2017;**68**(1):13.1-.27.

88. Chen H, Zeng Y, Yang Y, Huang L and Qiu Q. Allele-aware chromosome-level genome assembly and efficient transgene-free genome editing for the autotetraploid cultivated alfalfa. Nature Communications 2020;**11**(1):2494.

89. Edger PP, Poorten TJ, Vanburen R, Hardigan MA, Colle M, Mckain MR, et al. Origin and evolution of the octoploid strawberry genome. NPG Open Access 2019;**51**(3):541-7.

90. Bai WN, Yan PC, ZHang BW, Woeste KE, Lin K and Zhang DY. Demographically idiosyncratic responses to climate change and rapid Pleistocene diversification of the walnut genus Juglans (Juglandaceae) revealed by whole-genome sequences. New Phytologist 2018;**217**(4):1726-36.

91. Yan F, Xi RM, She RX, Chen PP, Yan YJ, Yang G, et al. Improved de novo chromosome- level genome assembly of the vulnerable walnut tree Juglans mandshurica reveals gene family evolution and possible genome basis of resistance to lesion nematode. Mol Ecol Resource 2021;**00**:1-14.

92. Guo W, Chen J, Li J, Huang J and Lim KJ. Portal of Juglandaceae: A comprehensive platform for Juglandaceae study. Horticulture Research 2020;**7**:35.

93. Martínez- García PJ, Crepeau MW, Puiu D, Gonzalez- Ibeas D, Whalen J, Stevens KA, et al. The walnut (Juglans regia) genome sequence reveals diversity in genes coding for the biosynthesis of non- structural polyphenols. The Plant Journal 2016;**87**(5):507-32.

94. Farag and Mohamed A. Headspace Analysis of Volatile Compounds in Leaves from the Juglandaceae (Walnut) Family. Journal of Essential Oil Research 2008;**20**(4):323-327.

95. Okan K, Aydin S, Apaydin E and Sevindik E. Antimicrobial Activity of Essential Oils from Juglans regia L. (Juglandaceae) Leaves Grown in the West Anatolian Area. ProEnvironment 2018;**11**:32-6.

96. Li YG, Mou FJ and Li KZ. De novo RNA sequencing and analysis reveal the putative genes involved in diterpenoid biosynthesis in Aconitum vilmorinianum roots. 3 Biotech 2021;**11**(2):1-12.

97. Ma Y, Cui G, Chen T, Ma X and Huang L. Expansion within the CYP71D subfamily drives the heterocyclization of tanshinones synthesis in Salvia miltiorrhiza. Nature Communications 2021;**12**(1):685.

98. Zhao Q, Weng JK, Chen XY, Martin C, Cui MY, Levsh O, et al. Two CYP82D Enzymes

Function as Flavone Hydroxylases in the Biosynthesis of Root-Specific 4'-Deoxyflavones in Scutellaria baicalensis. Molecular Plant 2018;**11**(1):135-48.

99. Cheng Y, Liu H, Tong X, Liu Z, Zhang X, Li D, et al. Identification and analysis of CYP450 and UGT supergene family members from the transcriptome of Aralia elata (Miq.) seem reveal candidate genes for triterpenoid saponin biosynthesis. BMC Plant Biology 2020;**20**(1):214.

100. Xing GL, Li JY, Li WL, Lam SM, Yuan HL, Shui GH, et al. AP2/ERF and R2R3-MYB family transcription factors: potential associations between temperature stress and lipid metabolism in Auxenochlorella protothecoides. Biotechnology for Biofuels 2021;**14**(1):1-16.

101. Maeo K, Tokuda T, Ayame A, Mitsui N and Nakamura K. An AP2-type transcription factor, WRINKLED1, of Arabidopsis thaliana binds to the AW-box sequence conserved among proximal upstream regions of genes involved in fatty acid synthesis. Plant Journal 2010;**60**(3):476-87.

102. Scbbastien B, Sylvie MW, Alexandra T, Christine R and Locc L. Role of WRINKLED1 in the transcriptional regulation of glycolytic and fatty acid biosynthetic genes in Arabidopsis. The Plant Journal 2009;**60**(6):933-47.

103. Meng Z, Xia C, Jia Q and Ohlrogge J. FUSCA3 activates triacylglycerol accumulation in Arabidopsis seedlings and tobacco BY2 cells. Plant Journal 2016;**88**(1):95-107.

104. Bo S, Allen WB, Zheng P, Li C and Glassman K. Expression of ZmLEC1 and ZmWRI1 Increases Seed Oil Production in Maize. Plant physiology 2010;**153**:980-7.

Figure legends

Figure 1 Genome information and Hi-C interaction heatmap of *J. mandshurica*. **a** distribution of *J. mandshurica* genomic features. (A) Circular representation of the Chromosome. (B) gene density. (C) repeat sequence density. (D) rRNA density. (E) tRNA density. (F) GC content density. **b** Intensity signal heatmap of the Hi-C chromosome.

Figure 2 Phylogenetic analyses of the *J. mandshurica* genome. (**a**) Venn diagram showing the shared and unique gene families among *J. mandshurica* and four other species (*J. cathayensis*, *J. regia*, *J. macrocarpa* and *O. sativa*). (**b)** an overview of orthologous and paralogous genes among *J. mandshurica* and related species. 'Single-copy orthologs' include common orthologs with one copy in specific species. 'Unique paralogs' include genes that do not have any similarity to genes in the other species based on BLAST and OrthoMCL. 'Multi-copy orthologs' include common orthologs with multiple copy numbers in specific species. 'Unclustered' include genes that cannot be clustered into known gene families. 'Other orthologs' include genes from families shared in 2–15 species. (**c)** phylogenetic tree of 15 species based on orthologs of single-gene families. Blue numbers at each node represent the estimated time of each divergent event. Green and orange numbers along each branch indicate the number of expanded and contracted gene families, respectively. Pie charts show the proportions of gene families that underwent expansion or contraction.

Figure 3 Collinearity and WGD analysis of *Juglans mandshurica* genome. **(a)** Ks distribution of synthetic orthologs of the selected species (*J. mandshurica*, *J. regia* and *J. sigillata*). (**b)** 4dTV analysis. The x-coordinate is the 4DTv value, and the y-coordinate represents the number of gene pairs of density. (**c)** schematic representation of syntenic genes among *J. mandshurica*, *J. regia* and *P. trichocarpa*. Gray lines in the background indicate collinear blocks of at least thirty genes within the *J. mandshurica* genome and other plants, while the red lines highlight the syntenic gene pairs.

Figure 4 Comparative transcriptomic analysis of genes involved in juglone biosynthesis. **(a)** Juglone biosynthetic pathway. Numbers under the chemical formulae represent the reaction substrate. 1, Chorismite; 2, Isochorismate; 3, SEPHCHC; 4, SHCHC; 5, OSB; 6, OSB-CoA; 7, DHNA-CoA; 8, DHNA; 9, Demethylphylloquinone; 10, Demethylphylloquinol; 11, Phylloquinol; 12, 1,4-NQ. Numbers next to arrows represent characterized enzymes or detected enzymatic

activities. 5.4.4.2, isochorismate synthase; 2.2.1.9, 2-succinyl-5-enolpyruvyl-6-hydroxy-3-cyclohexene-2-carboxylate (SEPHCHC) synthase; 4.2.99.20, 2-succinyl-6-hydroxy-2,4-cyclohexadiene-2-carboxylate (SHCHC) synthase; 4.2.1.113, 4.2.1.113 o-succinylbenzoate (OSB) synthase; 6.2.1.26, OSB-CoA ligase; 4.1.3.36, 1,4-dihydroxy-2-naphthoyl-CoA (DHNA-CoA) synthase; 3.1.2.28, DHNA-CoA thioesterase; 2.5.1.74, DHNA phytyl transferase; 1.6.5.9, NAD(P)H dehydrogenase C1 (NDC1); 2.1.1.163, demethylmenaquinone methyltransferase; 4.1.1-, decarboxylase; 2-ODD, 2-ODD: 2-oxoglutarate/Fe(II)-dependent dioxygenase; CYP450, cytochrome CYP450. The red chemical formulae represent Juglone. (**b)** Gray lines in the background indicate collinear blocks of at least thirty genes within the *J. mandshurica* genome and *J. regia*. The red lines highlight the syntenic gene pairs related to a decarboxylase (gene-Jman015G0125100), the green lines highlight syntenic gene pairs related to CYP450s (gene-Jman003G0044800), the blue lines highlight the syntenic gene pairs related to 2-ODD (gene-Jman011G0199700). (**c)** Lineage-specific expansion of the CYP gene family in *J. mandshurica* and three related species. (**d)** the heatmap represents the expression level of the expanded CYP gene family in *J. mandshurica*.

Figure 5 Juglone content and TF regulation during walnut exocarp differentiation. **(a)** The change of *bona fide* juglone content during walnut exocarp differentiation of *J. mandshurica* (mean ± SD, n=3); **b**, Frequency distribution of the first top 15 transcriptome factors related to juglone (r>0.8). (**c)** kinetic patterns of co-expressed genes in cluster 6 (990 genes) during walnut exocarp differentiation. (**d)** Correlation network of juglone-genes (2 genes and 19 transcription factors). r represents the Pearson correlation coefficient. Line color represents the correlation between genes and Juglone (positive and negative correlations) where a positive correlation is a formal representation of an activator and a negative correlation represents a formal representation of an inhibitor. (**e**) Expression analysis of transcription factors (14 MYBs, 3 FAR1s, 3 NF-YCs, 5 bZIP,s 8 NACs and 4 NF-YA TFs). The heatmap represents normalized fragments per kilobase of transcript per million fragments (FPKM). S1-S4 represents the fruit differentiation stages of *J. mandshurica*.

Figure 6 Comparative transcript analysis of genes involved in lipid biosynthesis of developing fruit. PDH, pyruvate dehydrogenase; ACC (BC), Biotin carboxylase subunit of heteromeric acetyl-CoA carboxylase (ACCase); ACC(BCCP), biotin carboxyl carrier protein of heteromeric ACCase; ACP, acyl carrier protein; KAS, ketoacyl-ACP synthase; SAD, stearoyl-ACP desaturase; FATA, acyl-ACP

thioesterase A; FATB, acyl-ACP thioesterase B; LACS, long-chain acyl-CoA sythetase; GPAT, glycerol-3-phosphate acyltransferase; LPAT, 1ysophosphatidic acid acyltransferase; PAP, phosphatidic acid phosphatase; DGAT, diacylglycerol acyltransferase; PDAT, phospholipid: diacylglycerol acyltransferase; TAG, triacylglycerol; CPT, diacylglycerol cholinephosphotransferase; FAD2, v-6 desaturase; FAD3, v-3 desaturase. G-3-P, glycerol-3-P; LPA, 1-Acylglycerol-3P; PA, 1,2-diacylglycerol-3P; DAG, 1,2- diacylglycerol.

Figure 1

Click here to access/download;Figur

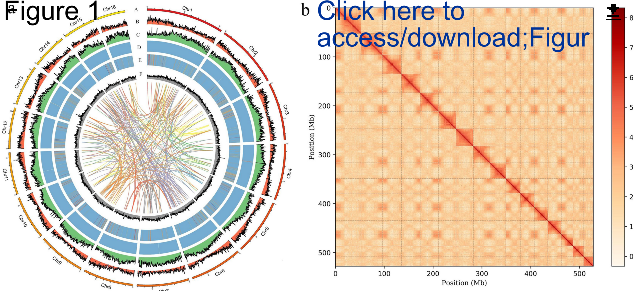

Click here to access/download;Figure;Figure 2.pdf

Figure 2

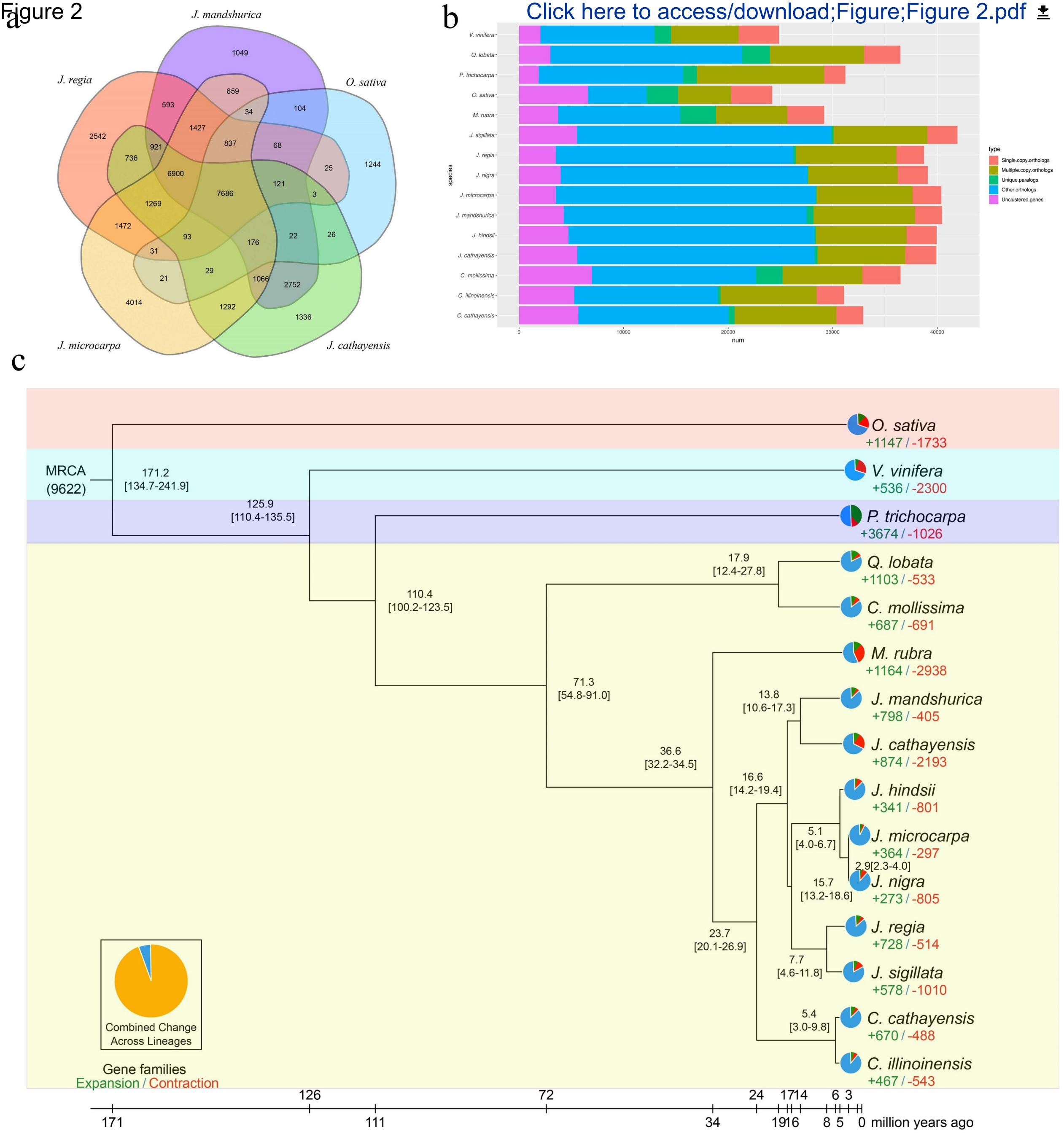

Click here to access/download;Figure;Figure 3.pdf

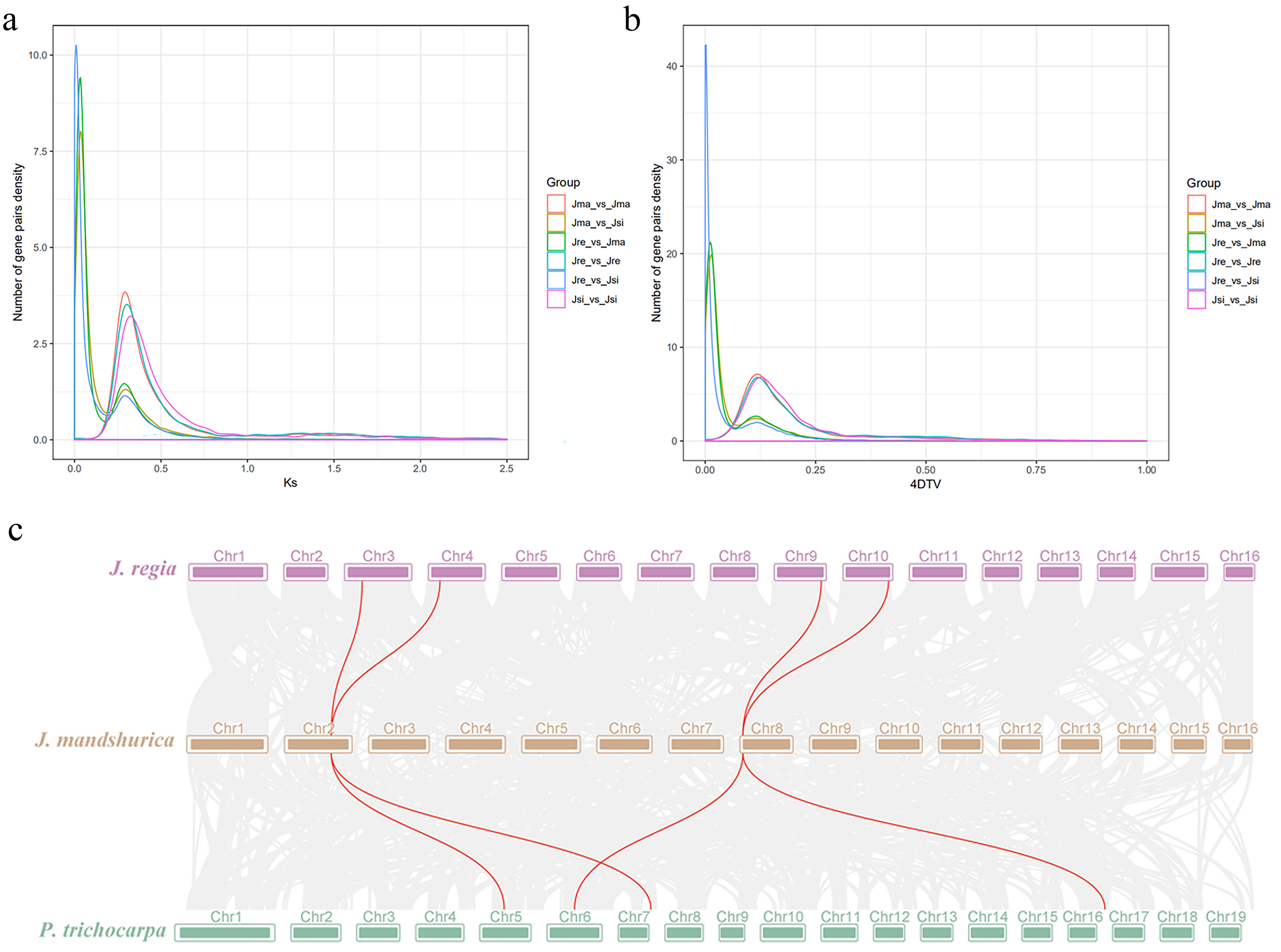

Figure 4

Click here to access/download;Figure;Figure 4.pdf

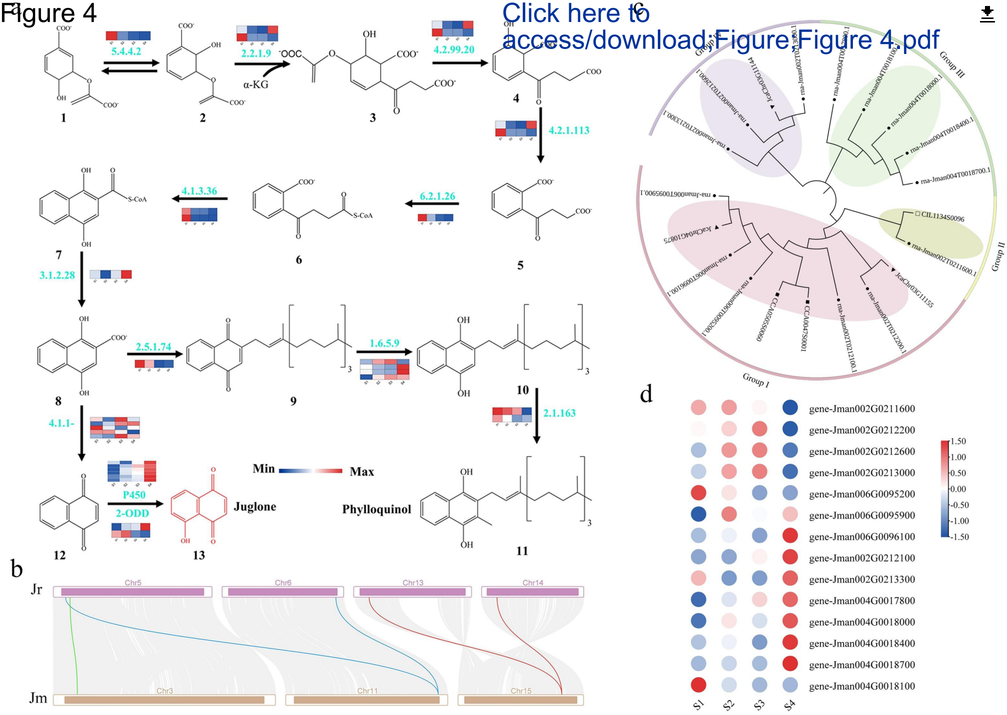

Figure 5

Click here to access/download;Figure;Figure

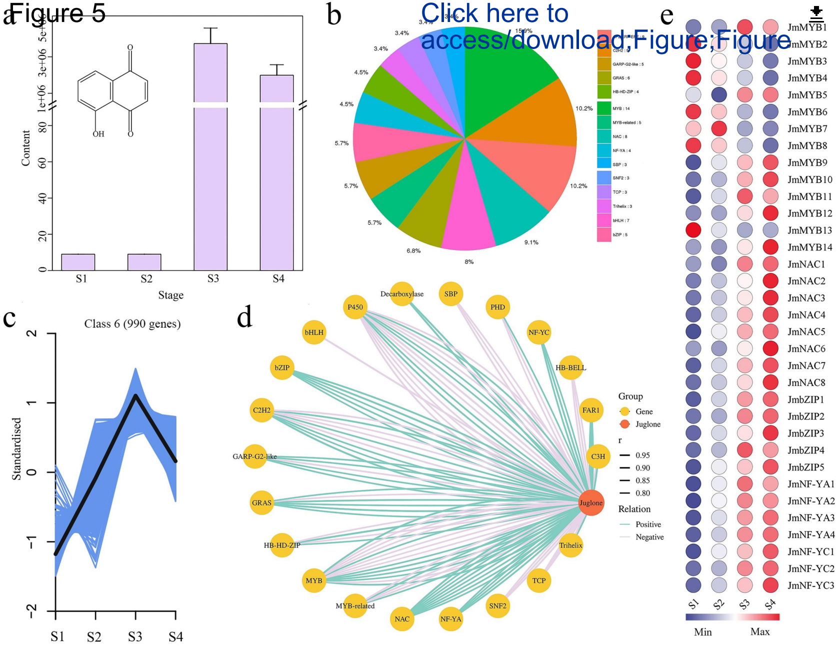

Figure 6

Click here to access/download;Figure;Figure 6.pdf

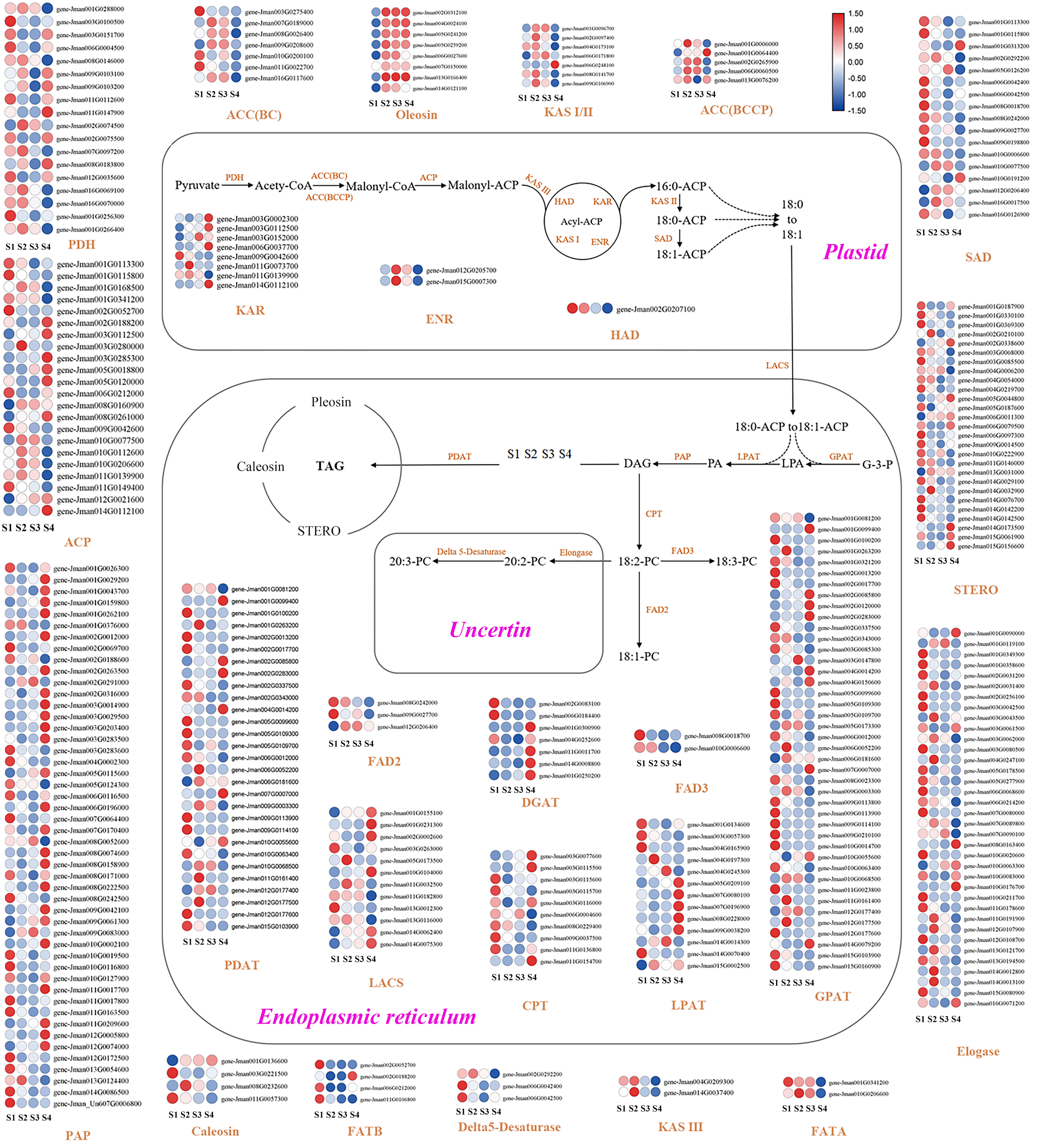

Supplementary Figures

Click here to access/download
Supplementary Material
Supplementary Figures.docx

Supplementary Tables

Click here to access/download
Supplementary Material
Supplementary Tables.xlsx

Click here to access/download;Personal Cover;Cover Letter.doc

Dear Editor,

On behalf of my co-authors, I am submitting the enclosed material "The Manchurian Walnut Genome: Insights into Juglone and Lipid Biosynthesis" for possible publication in **GigaScience**.

According to the comments, we have carefully checked and modified the present manuscript from the following aspects: (1) the detailed methods on metabolomics have been added from line 391-423; (2) the PCA analysis for the metabolights has been added in Figure S10 (walnut exocarp) and Figure S11 (walnut embryos). In the manuscript, we descripted the PCA methods and results from line 426-428 and 435-438. From the principal component analysis of walnut exocarp (Figure S10) and embryos (Figure S11), the obvious distinction of metabolites was found from different sample groups, and these metabolites can be used for subsequent metabolomics analysis; (3) the metabolomics data in this study were deposited and available at Metabolights repository (www.ebi.ac.uk/metabolights/MTBLS3657) under the accession numbers MTBLS3657, see line 681-683; (4) based on odb10, the BUSCO software with latest version (v5.2.2) was employed to evaluate the completeness of the *J. mandshurica* assembly, see line 147. The final BUSCO assessment shows that 98.3% of the complete BUSCO gene set was captured, and the results were added in Table S2; (5) in addition, we also added the photographs of *J. mandshurica* fruits in different development periods and the tissue structure of *J. mandshurica* fruit including the walnut exocarp and walnut embryos, see Figure S1 and Figure S2. All contents modified above has been marked by red color in present manuscript.

All authors certify that they have participated sufficiently in the work to take public responsibility for the appropriateness of the experimental design and method, and the collection, analysis, and interpretation of the data.

The authors have reviewed the final version of the manuscript and approve it for publication. To the best of our knowledge and belief, this manuscript has not been published in whole or in part nor is it being considered for publication elsewhere.

Now the manuscript is submitted online, and I look forward to hearing from you.

Yours Sincerely,

Xiyang Zhao

State Key Laboratory of Tree Genetics and Breeding, School of Forestry, Northeast Forestry University, Harbin, 150040, China.

Tel.: +0086-451-82192225

Fax: +0086-451-82192225

Email: zhaoxyphd@163.com
