# Supplementary material for: The Manchurian Walnut Genome: Insights into Juglone and Lipid Biosynthesis
Source: Gigascience. 2022 Jun 28;11:giac057. doi: 10.1093/gigascience/giac057 (PMC9239856; doi:10.1093/gigascience/giac057)
Supplement: giac057_GIGA-D-21-00355_R2 [file giac057_giga-d-21-00355_r2.pdf]

GigaScience

The Manchurian Walnut Genome: Insights into Juglone and Lipid Biosynthesis

--Manuscript Draft--

**Manuscript Number:**	GIGA-D-21-00355R2
**Full Title:**	The Manchurian Walnut Genome: Insights into Juglone and Lipid Biosynthesis
**Article Type:**	Research
**Funding Information:**	State Key Laboratory Breeding Base of Mountain Bridge and Tunnel Engineering (2021A01) — Mr Xiyang Zhao<br>Fundamental Research Funds for the Central Universities (2572020DR01) — Mr Xiyang Zhao<br>Heilongjiang Touyan Innovation Team Program (Tree Genetics and Breeding Innovation Team) — Mr Xiyang Zhao

Abstract:

Background

Manchurian walnut (*Juglans mandshurica* Maxim.) is a tree with multiple industrial uses and medicinal properties in the Juglandaceae family (walnuts and hickories). *J. mandshurica* produces juglone that is a toxic allelopathic agent and has potential utilization value. Furthermore, the seed of *J. mandshurica* is rich in various unsaturated fatty acids and has high nutritive value.

Fundings

Here, we present a high-quality chromosome-scale reference genome assembly and annotation for *J. mandshurica* (n=16) with a contig N50 of 21.4 Mb by combining PacBio HiFi reads with Hi-C data. The assembled genome has an estimated sequence size of 548.7 Mb, and consists of 657 contigs, 623 scaffolds and 40,453 protein-coding genes. Totally, 60.99% of the assembled genome consists of repetitive sequences. Sixteen super-scaffolds corresponding to the 16 chromosomes were assembled, with a scaffold N50 length of 33.7 Mb and a BUSCO complete gene percentage of 98.3% . *J. mandshurica* displays a close sequence relationship with *J. cathayensis* , with a divergence time of 13.8 million years ago (mya). Combining the high-quality genome, transcriptome, and metabolomics data, we constructed a gene-to-metabolite network and identified 566 core and conserved differentially expressed genes, which may be involved in juglone biosynthesis. Five *CYP450* genes were found that may contribute to juglone accumulation. NAC, bZip, NF-YA and NF-YC are positively correlated with the juglone content. Some candidate regulators (e.g. FUS3, ABI3, LEC2 and WRI1 TFs) involved in the regulation of lipid biosynthesis were also identified.

Conclusions

Our genomic data provides new insights into the evolution of the walnut genome and creates a new platform for accelerating molecular breeding and improving the comprehensive utilization of these economically important tree species.

**Corresponding Author:**	Xiyang Zhao<br>Northeast Forestry University<br>Harbin, CHINA
**Corresponding Author Secondary Information:**	
**Corresponding Author's Institution:**	Northeast Forestry University
**Corresponding Author's Secondary Institution:**	
**First Author:**	Xiang Li
**First Author Secondary Information:**	

Powered by Editorial Manager® and ProduXion Manager® from Aries Systems Corporation

**Order of Authors:**	Xiang Li<br>Kewei Cai<br>Qinhui Zhang<br>Xiaona Pei<br>Song Chen<br>Luping Jiang<br>Zhiming Han<br>Minghui Zhao<br>Yan Li<br>Xinxin Zhang<br>Yuxi Li<br>Shikai Zhang<br>Su Chen<br>Guanzheng Qu<br>Mulualem Tigabu<br>Vincent L. Chiang<br>Ronald Sederoff<br>Xiyang Zhao
**Order of Authors Secondary Information:**	
**Response to Reviewers:**	Response to Reviwer1:<br>(1) 1. The author should compare the genomes of Juglans species (Marrano et al., 2021; Zhu et al., 2019; Zhang et al., 2020). As some genomes from other walnuts have been available (Marrano et al., 2021; Zhu et al., 2019; Zhang et al., 2020), it is necessary to perform a comparison using multiple genomes to detect some structural variation (SV). The comparison may give some interesting discoveries. In my opinion, the author should at least compare the genome of this species with other published species, such as Juglans regia (https://academic.oup.com/gigascience/article/9/5/giaa050/5841058?login=true), J. microcarpa (https://www.nature.com/articles/s41438-019-0139-1) to explain the differences in traits between the two species, but there were no data in the revised version.<br>Response: Thanks for your comments. The relative content has been added. See line 284-305.<br><br>(2) This manuscript was related to Lipid Biosynthesis. I question the value of the J. mandshurica oil compared to the relative species J. regia, J. mandshurica is more value for their resistance, medicinal crop, rootstock traits. J. mandshurica oil value is less than common walnut (J. regia).<br>Response: Thanks for your comments. The walnut embryos of J. mandshurica has a high fatty acid content, which is composed of linoleic acid, oleic acid, linolenic acid, palmitic acid and stearic acid. Recently, the oil content of J. mandshurica was defined as 68% in the “Catalogue of main tree species for forestry biomass energy” from National Forestry and Grassland Administration (http://www.gov.cn/xinwen/2017-11/17/content_5240492.htm). In previous study, it revealed that a mature J. regia embryo accumulated 69% oil in Pei’s paper [1]. Therefore, the oil content of J. mandshurica and J. regia was similar, and they are important woody oilseed tree species. In addition, it is reported that in J. mandshurica, other tissues, including roots, stems, leaves, branches, and bark may also have significant medicinal value [2].<br>[1] R Huang, Zhou Y, J Zhang, et al. Transcriptome Analysis of Walnut (Juglans regia L.) Embryos Reveals Key Developmental Stages and Genes Involved in Lipid Biosynthesis and Polyunsaturated Fatty Acid Metabolism[J]. Journal of Agricultural and Food Chemistry, 2020, 69(1).

	[2] Zhang, S., Zhan, W., Zhang, H., Song, S., Qu, X. et al. (2022). Genetic Improvement in Juglans mandshurica and Its Uses in China: Current Status and Future Prospects. Phyton-International Journal of Experimental Botany, 91(3), 489–505.<br><br>(3) What is the role of the authors in listing the chemical equations instead of English name in Figure 5?<br>Response: Thanks for your comments. The chemical formula can clearly show the structures of various compounds, especially the juglone.<br><br>(4) Line 447: Please add the Figure cite of these sentences.<br>Response: Thanks for your comments. The Figure cite has been added. See line 471.<br><br>(5) Figure7: Which tissues were used in comparative transcript analysis of genes involved in lipid biosynthesis of developing fruit.<br>Response: Thanks for your comments. The walnut embryo was used to perform the comparative transcript analysis of genes involved in lipid biosynthesis of developing fruit.<br><br><br>Response to Reviwer3:<br>(1) I quickly looked at the authors' responses. It seems they worked to a better version of the manuscript, even if I haven't had the time for reading this new version carefully. It might need some English revision. I have one only question: why the contig and scaffold N50 size does not change from the PacBio assembly to the Hi-C assembly? See lines 104-114.<br>Response: Thanks for your comments. The language has been revised. For the contig N50 and scaffold N50, we need to explain that the HiFi reads has high accuracy (> 99%); the contig-level PacBio assembly was obtained based on high-quality reads, and contig N50 was calculated based on reads; the chromosome-scale scaffolds were obtained based on contigs using Hi-C technology, and scaffold N50 was calculated based on contigs, rather than the reads. Therefore, the change of contig and scaffold N50 is very small (nearly unchanged) by Hi-C assembly. Similar results were also found in previous study.
**Additional Information:**	
**Question**	**Response**
Are you submitting this manuscript to a special series or article collection?	No
**Experimental design and statistics**<br><br><br>Full details of the experimental design and statistical methods used should be given in the Methods section, as detailed in our Minimum Standards Reporting Checklist. Information essential to interpreting the data presented should be made available in the figure legends.<br><br><br>Have you included all the information requested in your manuscript?	Yes
**Resources**<br><br><br>A description of all resources used,	Yes

including antibodies, cell lines, animals and software tools, with enough information to allow them to be uniquely identified, should be included in the Methods section. Authors are strongly encouraged to cite Research Resource Identifiers (RRIDs) for antibodies, model organisms and tools, where possible.<br><br>Have you included the information requested as detailed in our Minimum Standards Reporting Checklist?	
**Availability of data and materials**<br><br>All datasets and code on which the conclusions of the paper rely must be either included in your submission or deposited in publicly available repositories (where available and ethically appropriate), referencing such data using a unique identifier in the references and in the “Availability of Data and Materials” section of your manuscript.<br><br>Have you have met the above requirement as detailed in our Minimum Standards Reporting Checklist?	Yes

Manuscript Click here to access/download;Manuscript;Revised manuscript 20220523-final.docx

The Manchurian Walnut Genome: Insights into Juglone and Lipid Biosynthesis

Xiang Li[1,2,†], Kewei Cai[2,†], Qinhui Zhang[2,†], Xiaona Pei[1,†], Song Chen[2], Luping Jiang[2], Zhiming Han[2], Minghui Zhao[2], Yan Li[2], Xinxin Zhang[2], Yuxi Li[2], Shikai Zhang[2], Su Chen[2], Guanzheng Qu[2], Mulualem Tigabu[3], Vincent L. Chiang[2,4], Ronald Sederoff[4], Xiyang Zhao[1,2,*]

[1] College of Forestry and Grassland, Jilin Agricultural University, Changchun 130117, China.

[2] State Key Laboratory of Tree Genetics and Breeding, School of Forestry, Northeast Forestry University, Harbin 150040, China.

[3] Southern Swedish Forest Research Center, Faculty of Forest Science, Swedish University of Agricultural Sciences, Lomma SE-234 22, Sweden.

[4] Forest Biotechnology Group, Department of Forestry and Environmental Resources, North Carolina State University, Raleigh NC 27695, USA.

[†] These authors contributed equally to this work.

lx2016bjfu@163.com (X.L.);
ckwnefu@163.com (K.C.);
zqh19950201@163.com (Q.Z.);
xiaonapei2020@163.com (X.P.);
chengsongnet@gmail.com (S.C.);
zhuojiuyibei@163.com (L.J.);
h375347046@163.com (Z.H.);
zhaominghui66@163.com (M.Z.);
ly2019nefu@163.com (Y.L.);
zhangxinxin@nefu.edu.cn (X.Z.);
fagongzi@163.com (Y.L.);
zskphd@163.com (S.Z.);
chensunefu@163.com (S.C.);
gzqu@nefu.edu.cn (G.Q.);
mulualem.tigabu@slu.se (M.T.);
vchiang@ncsu.edu (V.L.C.);
ron_sederoff@ncsu.edu (R. S.)

Corresponding author:
Email: zhaoxyphd@163.com (X.Z.)
Tel.: +0086-451-82192225
Fax: +0086-451-82192225
Xiang Li [0000-0001-5330-3396];
Kewei Cai [0000-0002-8765-7085];
Mulualem Tigabu [0000-0003-2471-1168];
Xiyang Zhao [0000-0002-1744-5431]

Abstract

Background: Manchurian walnut (*Juglans mandshurica* Maxim.) is a tree with multiple industrial uses and medicinal properties in the Juglandaceae family (walnuts and hickories). *J. mandshurica* produces juglone that is a toxic allelopathic agent and has potential utilization value. Furthermore, the seed of *J. mandshurica* is rich in various unsaturated fatty acids and has high nutritive value.

Fundings: Here, we present a high-quality chromosome-scale reference genome assembly and annotation for *J. mandshurica* (n=16) with a contig N50 of 21.4 Mb by combining PacBio HiFi reads with Hi-C data. The assembled genome has an estimated sequence size of 548.7 Mb, and consists of 657 contigs, 623 scaffolds and 40,453 protein-coding genes. Totally, 60.99% of the assembled genome consists of repetitive sequences. Sixteen super-scaffolds corresponding to the 16 chromosomes were assembled, with a scaffold N50 length of 33.7 Mb and a BUSCO complete gene percentage of 98.3%. *J. mandshurica* displays a close sequence relationship with *J. cathayensis*, with a divergence time of 13.8 million years ago (mya). Combining the high-quality genome, transcriptome, and metabolomics data, we constructed a gene-to-metabolite network and identified 566 core and conserved differentially expressed genes, which may be involved in juglone biosynthesis. Five *CYP450* genes were found that may contribute to juglone accumulation. NAC, bZip, NF-YA and NF-YC are positively correlated with the juglone content. Some candidate regulators (*e.g.* FUS3, ABI3, LEC2 and WRI1 TFs) involved in the regulation of lipid biosynthesis were also identified.

Conclusions: Our genomic data provides new insights into the evolution of the walnut genome and creates a new platform for accelerating molecular breeding and improving the comprehensive utilization of these economically important tree species.

Keywords: *Juglans mandshurica*, PacBio SMART, Hi-C, HiFi, genome assembly, comparative genomics, juglone, lipid

Background

Juglans mandshurica Maxim. (NCBI:txid91218; 2n = 2x = 32), well known as "Manchurian walnut", is a fast-growing and valuable hardwood tree species. The family Juglandaceae contains ~23 species, all bearing edible and medicinal nuts [1]. *J. mandshurica* was widely cultivated in China, Korea, Siberia, Japan, India, and Russia. It is naturally distributed in the northeast regions of China [2]. Because of its highly desirable wood quality and medically active substances, *J. mandshurica* was widely used in construction, wood processing, oil production, medicine, and pesticide manufacturing. Its immature walnut peel (exocarp) contains bioactive components, including quinones, triterpenoids, flavonoids, phenolics and alkaloids, which can induce detumescence and analgesia, softening blood vessels and producing anti-inflammatory effects. Juglone (5-hydroxy-1,4-naphthoquinone, $C_{10}H_6O_3$) was sued for its anticancer activity [3-5]. The walnut embryos of *J. mandshurica* has a high fatty acid content (more than 60%), which is composed of linoleic acid, oleic acid, linolenic acid, palmitic acid and stearic acid. These fatty acids may aid in the prevention of coronary heart disease by decreasing blood lipids, enhancing immune functions, and modulating non-alcoholic fatty liver disease [6, 7]. In *J. mandshurica*, other tissues, including roots, stems, leaves, branches, and bark may also have significant medicinal value [8].

Juglone forms orange acicular (long and needle-like) crystals and exhibits various biological activities and multi-purpose applications. Juglone is a naphthoquinone heterocyclic compound, was firstly isolated and purified in the 1950s and artificially synthesized in 1887 [9]. Juglone has antibacterial, antitumor, antiviral, and anti-inflammatory effects and is mainly derived from the root, bark, leaves and immature walnut exocarp (peel) tissues of some Juglandaceae species, including *Carya cathayensis*, *C. illinoinensis*, *Juglans cathayensis*, *J. hindsii*, *J. nigra*, *J. regia*, *J. sigillata*, and *J. macrocarpa* [3, 9]. Juglone was considered as a potential new drug thus its separation, preparation, synthesis, and biological activities have been extensively studied [10-12]. In humans, juglone has antitumor activity and can significantly inhibit liver, colon, lung and pancreatic cancer [13]. Juglone and related naphthoquinones are enriched in immature walnut exocarp compared with bark and root. Further research is needed for effectively extracting high purity juglone. Juglone has toxic effects on some plants, showing obvious allelopathy, and therefore has been used as an effective bio-herbicides [9]. There remains great potential to utilize juglone for allelopathy, synthetic agrochemicals and natural colorants in agriculture. *J. mandshurica* might become a crucial plant resource for biomedical research. Targeted studies on its biosynthesis and molecular function are needed to explore its application to human health and economic development.

Understanding the regulation of genes involved in the biosynthesis of juglone in *J. mandshurica* could accelerate the utilization of juglone resources. There are at least four different natural metabolic pathways to synthesize 1,4-naphthoquinone (1,4-NQs) [14]. However, the biosynthesis and regulation of juglone remains unknown in plants, and only one primary biosynthetic pathway and a small number of genes are known

based on the study of roots and leaves in black walnut (*J. nigra*) [15]. The biosynthesis of juglone is related to the phylloquinone (vitamin K1) pathway, which shares the 1,4-dihydroxynaphthoic acid (DHNA) to synthesize 1,4-NQs by decarboxylases. Ultimately, 1,4-NQs react with 2-oxoglutarate/Fe (II)-dependent dioxygenase (2-ODD) families or by cytochrome P450s to form juglone. Additional transcription factors (TFs) and other elements may also participate in juglone biosynthesis. The biosynthesis of juglone has not been systematically studied by multi-omics and genome-based methods. To implement such a strategy, a genome sequence is needed.

In this work, we assembled a high-quality chromosome-level reference genome (548.7 Mb) of *J. mandshurica* by high fidelity (HiFi) long reads based on the Pacific Biosciences (PacBio) sequencing platform and high-throughput chromosome conformation capture (Hi-C). We detected and annotated 40,453 gene models and 24,415 gene families in *J. mandshurica*. The analysis of gene family evolution and divergence was also performed in this study. We screened potential transcription factors and candidate genes involved in the juglone and lipid biosynthesis pathways. We mapped a gene-to-metabolite network by combining data on the genome, transcriptome and the metabolome. This work provides valuable genetic information on the evolution of *J. mandshurica* and related species, and contributes to further elucidation of the juglone and lipid biosynthetic pathways.

Analysis

Sequencing and assembly of the *Juglans mandshurica* genome

A single adult Manchurian walnut tree (*J. mandshurica*) from northeast China was selected for whole-genome sequencing and assembly (Fig. 1). The genome size estimated through k-mer analysis, was approximately 547.99 megabases (Mb), with a 0.77% level of heterozygosity and 48.78% of repeat sequences. A total of 14,596,746,422 17-mers were identified based on HiFi sequencing data, and the 17-mer depth was 26. (Supplementary Table S1 and Fig. S1). To assemble this highly heterozygous genome, Illumina, PacBio, and Hi-C (high-throughput chromosome conformation capture) technologies were selected for whole-genome sequencing. A total of 14.62 gigabases (Gb), circular consensus sequencing HiFi long reads with a nearly 26× sequence depth (Supplementary Fig. S2), and 56 Gb WGS Illumina short reads were obtained, using Sequel II and Illumina HiSeq 2500 platforms, respectively (Table 1). HiFiasm [16] software was used to assemble the *J. mandshurica* genome. After primary correction and assembly, the initial contig number, total length, and N50 size were 657, 548,677,591 bp (~548.7 Mb), and 21,388,210 bp (~21.4 Mb), respectively. Finally, 623 long scaffolds were anchored and oriented on 16 pseudochromosomes with a scaffold N50 of 35,382,463 bp (~35.38 Mb). The genome size observed in the present study was similar to the results of k-mer analysis based on PacBio HiFi data, this may be attribute to the high-quality sequencing data and assembly. The *J. mandshurica* assembly was further improved using Hi-C paired-end reads using 51 Gb of Hi-C data in Lachesis [17]. A total of 50 contigs and 16 scaffolds were obtained after Hi-C assembly; the contigs N50 and scaffolds N50 were 21.4 Mb

and 35.4 Mb, respectively. Consequently, 528 Mb were distributed across 16 chromosome-scale scaffolds and occupied 96.26% of the final genome assembly. Chromosome numbering for *J. mandshurica* was based on the size of chromosomes from maximum (Chr1) to minimum (Chr16). Particularly, the assembly quality of *J. mandshurica* in the present study showed high-level comparability with the two genome assembly versions of this species reported previously (Table 1).

The Benchmarking Universal Single-Copy Orthologs (BUSCO) software [18] was employed to evaluate the completeness of the *J. mandshurica* assembly. BUSCO assessment showed that 98.3% of the complete BUSCO gene set was captured, indicating increased BUSCO evaluation score (Supplementary Table S2). The mapping rate between the subreads obtained via PacBio sequencing and *J. mandshurica* assembly was 99.44 %, and the coverage rate was 99.56 %, thereby highlighting the assembly's high integrity (Supplementary Table S3). Additionally, statistical analysis for single nucleotide polymorphisms (SNP) and insertions and deletions (indel) showed that the proportion of homozygous SNPs and indels in *J. mandshurica* assembly was not more than 0.01 %, suggesting that the assembly was of high-quality (Supplementary Table S4). Furthermore, among the total number of clean bases of mRNA-seq (Gb), obtained, an average of 51,559,765 (93.17%) and 55,542,790 (93.05%) reads that were obtained from transcriptome sequence (RNA-seq) in walnut exocarp and kernel, respectively, could be mapped back to the genome assembly (Supplementary Table S5). These results for the assembly and assessment verified that we obtained a high-quality reference genome with a high degree of completeness at the chromosome level.

Table 1 The statistics for genome sequencing of *J. mandshurica* compared with previously reported two genome assemblies of this species

Genomic features	*Juglans mandshurica* (V 3.0)	*Juglans mandshurica* (V 2.0)	*Juglans mandshurica* (V 1.0)
Sequence method	PacBio	Nanopore	Illumina
Raw bases (Gb)	14.62	62.87 Gb	*
Raw bases of Hi-C (Gb)	51	101 Gb	*
Raw bases of WGS Illumina (Gb)	56	47.3 Gb	49.05Gb
Genome size (Mb)	548.7	548.5	558.1
Number of scaffolds after assembly	*	213 (>=2 Kb)	*
N50 of scaffolds (bp) after assembly	*	7,154,770 (>=2 Kb)	*
Number of contigs after assembly	657	215 (>=2 Kb)	*
N50 of contigs (bp) after assembly	21,388,210	7,154,770 (>=2 Kb)	*
Number of scaffolds after Hi-C+assembly	623	189	13,810
N50 of scaffolds (bp) Hi-C+assembly	35,382,463	36,084,664	496,923
Number of contigs Hi-C+assembly	657	397	24385
N50 of contigs (bp) Hi-C+assembly	21,388,210	6,490,758	114,334
Anchored rate (%)	96.26	99.00	*
Complete BUSCOs (%)	98.3%	92%	*
GC content of the genome (%)	36.72%	38.51%	*
Number of predicted protein-coding genes	40,453	27,901	*

Average gene length (bp)	3,694.76	5,735	*
Average CDS length (bp)	1,104.82	1226.35	*
Average exon number per gene	6.1	6.06	*
Average exon length (bp)	289.02	244.1	*
Number of tRNA	2,185	581	*
Number of rRNA	4,004	348	*
Number of miRNA	122	132	*
Number of snRNA	272	792	*
Repeat sequences (bp)	334,673,373 (60.99%)	340,401,005 (62.08%)	293,181,849,6 (50.10%)
Annotated to Interpro	25,953 (64.16%)	25,016 (86.17%)	*
Annotated to GO	18,229 (45.06%)	10,155 (34.98%)	*
Annotated to KEGG_ALL	31,243 (77.23%)	20,806 (71.67%)	*
Annotated to Swiss-Prot	23,059 (57.00%)	20,902 (72%)	*
Annotated to NR	32,855 (81.22%)	27,815 (95.81%)	*
DNA TEs	51,543,175 (9.39%)	49,110,954 (8.96%)	*
LINE TEs	45,973,640 (8.38%)	67,022,583 (12.22%)	*
SINE TEs	1,897,331 (0.35)	58,768 (0.01%)	*
LTR TEs	215,930,087 (39.35)	226,061,071 (41.23%)	*
Total transposable element (TEs)	326580986 (59.52)	342,253,376 (62.42%)	*

Asterisk (*) represents data were not shown in the original articles.

Gene prediction and annotation of the *Juglans mandshurica* genome

In total, 40,515 protein-coding genes were predicted, with an average gene length of 3,694.76 bp, by combining the *de novo*, transcriptome or Iso-Seq (Isoform sequencing), and homology-based methods using MAKER (v3.0) [19] (Supplementary Table S6). For these predicted genes, 40,453 (99.85%) of the genes were anchored to pseudochromosomes, and the average exon number per gene was 6.1 with an average length of 289 bp and the average coding sequence length was 1105 bp (Supplementary Fig. S3). The total GC content of the genome assembly was 36.7 % and was distributed across 16 pseudochromosomes (Table 1 and Fig. 2). Among these genes, 32,901 (81.33%) of the genes were functionally annotated to InterPro (25,953, 64.16%), Gene Ontology (GO) (18,229, 45.06%), Kyoto Encyclopedia of Genes and Genomes (KEGG) (31,243, 77.23%), Swiss-Prot (23,059, 57%), TrEMBL (31,341, 77.48%), and NR (32,855, 81.22%) public databases, and a total of 16,218 core genes were annotated in the abovementioned databases (Supplementary Table S7 and Supplementary Fig. S4). Gene distribution in 16 pseudochromosomes of the *J. mandshurica* genome was uneven, as found in other plant species, such as *J. regia* × *J. macrocarpa*, *J. regia*, *Rhododendron simsii* (azalea), and *Sechium edule* (chayote) [20-23] (Fig. 2). We identified several noncoding RNA genes, containing 122 microRNAs (miRNAs), 2,185 transfer RNAs (tRNAs), 4,004 ribosomal RNAs (rRNAs), and 272 small nuclear RNAs (snRNAs) in the *J. mandshurica* genome (Supplementary Table S8 and Fig. 2).

Furthermore, we identified 334,673,373 bp of repetitive sequences combining the *de novo* and homology-based approaches in *J. mandshurica*, and we accounted for 60.99% of the genome assembly (Supplementary Table S9 and Fig. 2). In total, 326,580,986 bp (59.52%) of transposable elements (TEs) were found, which was

comparable to *Acer truncatum* (Shantung maple; ~61.75%) [24] and *Eucommia ulmoides* (hardy rubber tree; ~62.5%) [25]; however, it was higher than that in *Tripterygium wilfordii* (thunder duke vine; 52.36%) [26] and *Betula platyphylla* (white birch; 43.0%) [27]. The predominant TEs were long terminal repeat (LTR) retrotransposons, accounting for 39.35% of the assembled genome, followed by the DNA transposons (9.39%), long interspersed nuclear elements (LINEs, 8.38%), and short interspersed nuclear elements (SINEs, 0.35%) (Supplementary Table S9). Most TEs were gypsy and Copias-like LTRs that covered 65,135,736 bp and 93,428,433 bp respectively, and accounted for 11.87 % and 17.03% in the assembled genome (Supplementary Table S10 and Table S11).

Gene family identification and evolutionary analysis

J. mandshurica shared 7,686 gene families with four related plant species and possessed 2,584 single-copy orthologs and 225 unique families (Fig. 3a and Supplementary Table S12). Particularly, the number of single- and multiple-copy genes of *J. mandshurica* was similar to that in the other genus *Juglans* species (Fig. 3b and Supplementary Table S13). To examine the genome evolution of *J. mandshurica* and the Juglandaceae family, 558 single-copy orthologous genes from 13 species of rosid families (i.e., *J. cathayensis*, *J. macrocarpa*, *J. nigra*, *J. regia*, *J. hindsii*, *J. sigillata*, *C. cathayensis*, *C. illinoinensis*, *Quercus lobata* [valley oak], *Castanea mollissima* [Chinese chestnut], *Morella rubra* [red bayberry], *Populus trichocarpa*, and *Vitis vinifera*) and one commelinid species (*Oryza sativa*) were identified via OrthoMCL [28] and employed to construct a phylogenetic tree and evaluate the divergence times using RAxML (version 8.2.11) [29] employing default settings (Fig. 3c). A total of 40,453 genes were clustered into 24,415 (60.35%) gene families in *J. mandshurica* with an average of 1.48 genes per family (Supplementary Table S12). *J. mandshurica* was closely related to *J. cathayensis*, with an estimated divergence time of 13.8 (10.6–17.3) million years ago (mya). Species in the genus Juglans were clustered in the same group; they shared a common ancestor with species in the genus *Carya* having diverged approximately 23.7 (20.1–26.9) mya. Within the genus Carya, the divergence of its two species was estimated at 5.4 (3.0–9.8) mya. Species in the Juglandaceae and Myricaceae families (such as *Morella rubra*) diverged approximately 36.6 (32.2–34.5) mya. Additionally, we analyzed gene family expansion and contraction across the related species using CAFÉ (Computational Analysis of gene Family Evolution) to explore the evolution of *J. mandshurica* (Fig. 3c). Among the 24,415 gene families, 798 and 405 expanded and contracted in *J. mandshurica*, respectively, after divergence from *J. cathayensis*. In addition, regarding the gene families of species in Juglandaceae family, the number of expansions was higher than that of contractions in *J. macrocarpa* and *J. regia*. In species belonging to the Fagaceae family, i.e., *Quercus lobata* and *Castanea mollissima*, more gene families expanded (1,103 and 687) and fewer gene families contracted (533 and 691), respectively. In total, 521 (65.29%) of the 798 expanded gene families displayed rapid evolution in the *J. mandshurica* genome (family-wide *P*-value < 0.05) and annotated in functions related to metabolic process, cellular processes, cell, cell part, and binding and catalytic activity based on the GO

category (Supplementary Fig. S5). We further clustered these genes from rapidly expanded gene families were further clustered into 88 KEGG pathways. The expanded gene families were primarily involved in photosynthesis (ko00195), ribosome (ko03010), MAPK signaling pathway (ko04010), plant–pathogen interaction (ko04626), and protein processing in the endoplasmic reticulum (ko04141) (Supplementary Table S14). The contracted families were annotated to 20 KEGG pathways, and they mainly participated in the NF-κB signaling pathway (ko04064), immune deficiency and toll signaling pathway (ko04624), toll-like receptor signaling pathway (ko04620), and MAPK signaling pathway (ko04010) (Supplementary Table S15).

Analyses of genome synteny and whole-genome duplication (WGD)

WGD occurred in the evolutionary history of most plant species and provided the evolutionary potential for new functions and species diversification [30]. We computed Ks (synonymous substitutions per synonymous site) and 4dTV (4-fold degenerate synonymous sites of the third codons) value among the genes of *J. mandshurica*, *J. regia*, and *J. sigillata* to analyze gene duplication and divergence. Fossil record showed that the Juglandaceae family appeared in the upper Cretaceous period and radiated in the Paleocene period [31-33]. The Juglandoid WGD must have originated prior to the radiation of Juglandaceae in the Paleocene. Therefore, we selected the Cretaceous–Paleogene (C-Pg) boundary (66 mya) as the approximate time of the origin of the Juglandoid WGD. The distribution of these two methods was remarkably consistent (Fig. 4a, b). The Ks plot of Jma_vs._Jma, Jre_vs._Jre, Jsi_vs._Jsi (self-searches within the *J. mandshurica*, *J. regia* and *J. sigillata genomes*) reflected the divergence of paralogous genes, originated though the Juglandoid WGD. It showed a significant main peak of approximately 0.3, which was consistent with the results of other similar studies on Juglandoids [22].

Furthermore, we detected synteny between the assembly of the *J. mandshurica* genome and that of *J. regia.* Synteny analysis showed a strong correspondence for all 16 chromosomes in these plants, indicating that the collinearity was maintained at a high level, which suggested the presence of a close evolutionary relationship between two species (Fig. 4c and Supplementary Fig. S6). We identified a large number of collinear gene pairs between the chromosomes of *J. mandshurica* (Fig. 2a). We detected linear relationships between *J. mandshurica*, *J. regia* and *P. trichocarpa*, and there were significantly distinct syntenic blocks. A total of 49,921 and 38,462 collinear genes were identified between *J. mandshurica* and *J. regia* and between *J. mandshurica* and *P. trichocarpa*, respectively, indicating that 62.5% and 52.3% of the *J. mandshurica* genome was colinear in these plants. To illustrate, Chr2 of *J. mandshurica* shared origins with Chr3 and Chr4 in *J. regia* and with Chr5 and Chr7 in *P. trichocarpa* (Fig. 4c). Therefore, it was evident through the abovementioned results that ancestral collinearity existed between these three species.

Genomic SVs between *J. mandshurica* and other two walnut species

According to the phylogenetic tree, seven *Juglans* species were obviously divided into three subgroups (Fig. 3). To further understand the genomic differences of walnut

species, the structural variation (SV) of *J. mandshurica* vs *J. microcarpa* and *J. mandshurica* vs *J. regia* was detected through direct genome comparison. Totally, 13,644 translocation events, and 347 inversion events were identified between *J. mandshurica* and *J. macrocarpa*, and there are obvious inversion events in chromosome 1 and chromosome 10 in present study (Supplementary Table S16 and Supplementary Fig. S7). Genes affected by the obtained translocation events were enriched in the ‘fatty acid biosynthesis’ (ko00061), ‘biosynthesis of unsaturated fatty acids’ (ko01040), ‘MAPK signaling pathway’ (ko04010) and ‘metabolism of xenobiotics by cytochrome P450’ (ko00980) et al., indicating that these translocation events may contribute to the differences in metabolites synthesis, response to the stimulus, and lipid accumulation between *J. mandshurica* and *J. macrocarpa* (Supplementary Table S17). Furthermore, 19,476 translocation events, and 309 inversion events were identified between *J. mandshurica* and *J. regia* (Supplementary Table S16 and Supplementary Fig. S7). Genes affected by the identified translocation events were enriched with those related to ‘stilbenoid, diarylheptanoid and gingerol biosynthesis’ (ko00945), ‘flavonoid biosynthesis’ (ko00941), ‘sesquiterpenoid and triterpenoid biosynthesis’ (ko00909), ‘Plant-pathogen interaction’ (ko04626) et al., suggesting that these translocation events may be involved in the differences in secondary metabolites synthesis and disease resistance between the *J. mandshurica* and *J. regia* (Supplementary Table S18).

Gene discovery analysis related to Juglone biosynthesis

The high economic value of *J. mandshurica* is attribute to the accumulated juglone in the roots, leaves, bark, and in particular, the walnut exocarp [9]. As an important quinoid component, juglone’s medicinal activity and potential use for sustainable agriculture have been confirmed [9]. However, the biosynthesis, mechanism of action, and regulatory network involved in the juglone pathway require further elucidation; only a few small numbers of the relevant genes have been identified via RNA-sequencing [15]. Juglone biosynthesis in walnut species is partly affiliated with the phylloquinone pathway, wherein the initial substrate was chorismite from the shikimate pathway [15]. These two pathways shared the 1,4-dihydroxynaphthoic acid (DHNA) intermediate, and seven enzymes are used for the DHNA synthesis of phylloquinone. DHNA is subsequently converted to 1,4-naphthoquinone (1,4-NQs), by various decarboxylases (Fig. 5a). Juglone is synthesized by 1,4-NQs through hydroxylation facilitated by hydroxylases, CYP450s, and 2-ODD. In the present study, we combined genomic, transcriptomic, and metabolomic technologies to analyze the fruit development process in *J. mandshurica*, and identify genes regulating juglone biosynthesis in the exocarp. Among the 146 candidate genes encoding enzymes for juglone biosynthesis identified, 1 encoded isochorismate synthase; 2 encoded SEPHCHC, SHCHC, and OSB; 1 encoded OSB-CoA ligase; 2 encoded DHNA-CoA, 1 encoded DHNA-CoA thioesterase, 1 encoded DHNA phytyl transferase, 4 encoded NDC1, 2 encoded demethylphylloquinone methyltransferase, 5 encoded decarboxylases, 2 encoded 2-ODDs, and 125 encoded CYP450 (Fig. 5a and Supplementary Table S19). Expression levels of the decarboxylases, *2-ODDs*, and the *CYP450* genes in the S3 and S4 stages were higher than in the S1 and S2 stages; thus, these genes have a potential role in juglone biosynthesis in *J. mandshurica*. Particularly,

16 of 146 key genes were significantly correlated with the juglone (r > 0.8 or < −0.8), including 2 genes encoding decarboxylase and 14 genes belonging to CYP450 family (Supplementary Table S19).

Then we explored the specifically expanded gene families that may be involved in juglone biosynthesis. CYP450s are a class of important oxidative enzymes that are widely distributed in plants and play key roles in the biosynthesis of many natural secondary metabolites. CYP450 enzymes may catalyze various enzymatic steps in the juglone and phytohormones biosynthesis and plant stress responses. CYP450s are typically represented by catalytic reactions involving hydroxylation; furthermore, they catalyze other complex biosynthetic reactions including epoxidation of aromatic compounds and methyl or amino transfer reactions (transferases). The CYP gene family (14 genes in 4 groups) were identified for the *J. mandshurica* genome, and they appeared to have rapidly expanded in the *J. mandshurica* genome compared with the three related species (14 genes in *J. mandshurica* genome, 1 in *C. illinoinensis*, 1 in *C. cathayensis*, 3 in *J. cathayensis*) (Fig. 5b and Supplementary Table S20). The 14 genes in *J. mandshurica* are specifically distributed on chromosomes 2, 4, and 6, and 5 of the 14 genes were in the same group (Group Ⅲ) (Fig. 5c). Additionally, the 14 *CYP* genes in *J. mandshurica* were identified in the differentially expressed genes (DEGs) obtained through RNA-seq of the immature exocarp (Fig. 5d). Most *CYP* genes were differentially expressed in the S4 stage. The *CYP* gene family exhibited a particularly rapid expansion and the inferred increase in transcript abundance may contribute to the juglone accumulation.

Metabolite profiling and transcriptomics of walnut exocarp development

To further identify the co-expressed genes and regulators in juglone biosynthesis, transcriptomic and metabolomic analyses were performed on the four stages of walnut exocarp (Supplementary Fig. S8) and embryos (Supplementary Fig. S9) development. Principal component analysis of the walnut exocarp and embryos revealed that the obvious distinction between the metabolites was from different sample groups, and these metabolites can be used for the next step of metabolomics analysis. A total of 470 secondary metabolic products were detected, mainly containing seven types of metabolites, including phenolic acids (147, 31.3%), flavonoids (134, 28.5%), tannins (50, 10.6%), alkaloids (47, 10%), lignans and coumarins (28, 6.0%), terpenoids (24. 5.1%), and quinones (21, 4.5%), among others (19, 4.0%) (Supplementary Fig. S10 and Supplementary Table S21). Juglone content increased during the transition from S2 to S3 stages, whereas the juglone content was slightly reduced from S3 to S4 (Fig. 6a). The juglone profiles during the S1 and S2 stages from walnut exocarp were distinct compared with S3 and S4. In total, 195 metabolites were differentially accumulated between S1 and S2 vs. S3 and S4. Furthermore, we analyzed the differentially accumulated metabolites between different stages for the juglone component of walnut exocarp. In addition to S1 vs. S2 and S3 vs. S4, it was evident that four of the six pairs contain the differentially accumulated juglone (Supplementary Table S22), consistent with the abovementioned results, which show that the juglone content varies during walnut exocarp development in *J. mandshurica*. Additionally, we focused on the DEGs

derived from four (S1 vs. S3, S1 vs. S4, S2 vs. S3, and S2 vs. S4) paired groups that differentially accumulated juglone, and 897 common DEGs were found in these groups (Supplementary Fig. S11 and Supplementary Table S23). In a combined GO and KEGG enrichment analysis of the 897 DEGs, we observed that the most enriched terms were "extracellular region" and "integral component of membrane" in the GO database, and "biosynthesis of secondary metabolites" and "metabolic pathways" in the KEGG pathways (Supplementary Fig. S12 and Fig. S13).

Transcription factors (TFs) are important regulators of plant growth, development, metabolism, and adaptation [34]. We identified some transcription factors (TFs) that may be related to juglone biosynthesis of juglone during walnut exocarp development. There were 777, 1082, 154, and 826 differentially expressed TFs (DEG-TFs) in S1 vs. S3, S1 vs. S4, S2 vs. S3, and S2 vs. S4, respectively (Fig. 6b and Supplementary Table S24). In which, 62 MYB, 55 AP2/ERF-ERF, 50 NAC, 48 bHLH, 45 C2H2, 36 WRKY, and 26 bZIP TFs were found in S1 vs. S3, and 82 MYB, 73 C2H2, 72 bHLH, 71 AP2/ERF-ERF, 64 NAC, and 60 WRKY TFs were identified in S1 vs. S4; these TFs may be involved in juglone biosynthesis. In addition, we observed that the TFs essential for juglone biosynthesis were commonly identified in the four groups, i.e., AP2/ERF-ERF, bHLH, bZIP, C2H2, MYB, and NAC. Expression analysis indicated that most TFs showed higher expression in S3 and S4 compared with the S1 and S2, which was consistent with the results of metabolites, suggesting the presence of a strong association between these TFs and juglone accumulation during walnut exocarp differentiation.

To identify genes that displayed similar abundance patterns as juglone content, co-expression cluster analysis was performed using K-means methods based on the FPKM values. All genes were primarily clustered into ten clusters with distinct expressions, in which cluster 6 (990 genes) showed variation similar to that of the juglone content during walnut exocarp differentiation. This suggested that these genes were crucial to fully elucidate juglone biosynthesis at the transcript level (Fig. 6c). GO enrichment analysis showed that 990 DEGs were enriched in "intracellular," "intracellular part" and "RNA metabolic process" (Supplementary Fig. S14). The top enriched KEGG DEGs were "purine metabolism," "RNA polymerase" and "spliceosome" (Supplementary Fig. S15).

To more deeply explore the relationship between gene expression and juglone, data obtained from metabolites (juglone) and genes (including CYP450s and MYB, NAC, and bZIP TFs, among others) were employed to construct a gene-to-metabolite correlation network in the walnut exocarp. A total of 1,860 DEGs correlated with juglone content and the Pearson correlation coefficient was set at $r > 0.8$ or < -0.8 as the cutoff (Supplementary Table S25). The core conserved DEGs (566) in the four paired groups (S1 vs. S3, S1 vs. S4, S2 vs. S3, and S2 vs. S4) were mainly enriched in "biosynthesis of secondary metabolites," "alanine, aspartate, and glutamate metabolism" and "nitrogen metabolism" in KEGG terms (Supplementary Fig. S16 and Fig. S17). Among the 1,860 DEGs, 155 were identified as TFs, and the top fifteen15 TFs (see the pie chart) consist of MYB, C2H2, AP2/ERF-ERF, bHLH, NAC, and bZIP TFs, this indicates the presence of TF regulation for juglone during walnut exocarp

differentiation (Fig. 6b). Among the 14 *CYP450* genes, 5 genes that correlated with juglone (r > 0.8 or < −0.8) showed a strong positive correlation with the network, and 2 genes encoding decarboxylases showed the same relationship, thereby suggesting that these genes may be involved in juglone biosynthesis (Fig. 6d and Supplementary Table S26). Among the 155 TFs, all the bZIP (5), NAC (8), NF-YA (4), NF-YC (3)), and FAR1 (3) TFs were strongly correlated with juglone, and the majority of these TFs were highly expressed in the S3 and S4 stages, suggesting regulation functions for juglone (Fig. 6e). Therefore, these TFs could considerably influence the gene-to-juglone regulatory network. The five core *CYP450* genes and several key TFs could be suitable candidate genes, and help provide new insights into natural juglone biosynthesis.

Gene discovery for lipid biosynthesis and oil body formation

J. mandshurica is an important oil plant species, and its oil-rich (>50%) embryos have a variety of different oils such as oleic, linoleic, and unsaturated fatty acids, among others. The oil has important medicinal, nutritional, and industrial value. Although previous studies have found multiple oil components and confirmed their activity as antiviral, antimicrobial, anticancer, or antihelminth agents [35, 36], the molecular regulatory mechanisms for lipid biosynthesis and oil body formation in *J. mandshurica* require further elucidation [37-39]. Oil formation in plant seeds occurs through the lipid biosynthesis pathway, where the fatty acids are synthesized in the plastids, and triacylglycerol (TAG) synthesis occurs in the endoplasmic reticulum. TAGs are stored in an oil body after synthesis and degraded to provide carbon and energy during seed germination and early seedling growth [40].

In the present study, we investigated the regulation of genes involved in lipid biosynthesis and oil body formation. We used a combination of transcriptome sequencing and metabolomics on four different stages of differentiation *J. mandshurica* walnut embryos. Among the 450 metabolites identified in *J. mandshurica* samples, there were 99 lipids (22%), 91 amino acids and their derivatives (20.2%), 77 organic acids (17.1%), 61 phenolic acids (13.6%), 51 nucleotides and their derivatives (11.33%), and 71 other compounds (15.78%) (Supplementary Fig. S18 and Supplementary Table S27). Among these metabolites, lipids were predominant and primarily comprised 41 free fatty acids (41.4%), 28 lysophatidylcholine (LPC) (28.3%), 14 lysophatidylethanolamines (LPE) (14.1%), 13 glycerol esters (13.1%), 2 sphingolipids (2.02%), and 1 phosphatidylcholine (PC) (1.01%) (Supplementary Fig. S19 and Supplementary Table S28), thereby indicating that the walnut embryos are rich in free fatty acids during differentiation. Among these 41 free fatty acids, 11 had the highest content from S1 to S4 (Supplementary Fig. S20), including linoleic acid (C18:3), stearic acid (C18:0), arachidic acid (20:0), α-linolenic acid, and γ-linolenic acid, suggesting the presence of abundant oils in the *J. mandshurica* walnut embryos. The top enriched KEGG terms of these compared groups mainly were “metabolic pathway,” “biosynthesis of secondary metabolites,” “biosynthesis of amino acids” and “ATP-binding cassette transporters” (Supplementary Fig. S21). These oils were enriched during walnut differentiation and provided key information on lipid biosynthesis in *J. mandshurica*.

A transcriptome survey carried out to better understand the mechanisms underlying lipid biosynthesis and oil body formation facilitated the identification of 346 genes related to lipid synthesis. These genes included 105 for fatty acid (FA) biosynthesis, 202 for TAG biosynthesis, and 39 for oil body formation (Fig. 7). Expression levels of most of the structural genes (*PDH* and *ACC*[*BC]*) at the S2 stage were higher than the expression at other stages, whereas the majority of the other genes were significantly expressed in the S4 stage. This could partially explain the persistence of the high level of free fatty acid content in the S2 stage (Supplementary Fig. S22). After their formation, the free fatty acids are activated via long-chain acyl-CoA synthetases (LACS) to generate acyl-coenzyme A (Acyl-CoA) derivatives, which are transported out of the plastid (Fig. 7). The transcript level of *LACS* at S4 was higher than in other stages, which contributed to TAG synthesis [41]. Glycerol- 3- phosphate acyltransferase (GPAT) catalyzes the glycerol-3-phosphate and acyl -coenzyme A to form lysophosphatidic acid (LPA), and then LPA was then converted to phosphatidic acid (PA) with the help of lysophosphatidic acid acyltransferase (LPAT). Subsequently, phosphatidic acid phosphatase (PAP) converts PA to diacylglycerol (DAG). TAGs are finally synthesized with through the reaction of DAG via diacylglycerol acyltransferase (DGAT) or diacylglycerol acyltransferase (PDAT) [42]. Most of the differentially expressed *PAP* genes were expressed at high levels in S4, thereby indicating a dominant role for TAG formation. The expression of *DGAT* and *PAP* genes appeared to have similar to *PAP* genes. To form oil bodies, expression levels. Oil body formation involves the binding of TAGs are bound to several proteins, including oleosionoleosin, caleosin, and steroleosin (STERO) [43]. Oleosin is an oil protein that could increase charge repulsion, allowing oil bodies to be independent of each other. Oleosin maintains high expression in S2, S3, and S4 in the walnut embryos; this suggested that genes encoding these proteins were good candidates for genes regulating oil body formation.

Several TFs regulate plant growth and development. Among the 15,152 DEGs, 1,876 were identified in *J. mandshurica*, representing 12.38% of the DEGs involved in the walnut embryo differentiation (Supplementary Table S29). The top five transcription factor families were 110 MYBs, 109 C2H2s, 105 AP2/ERF-ERFs, 102 bHLHs, and 76 NACs, many of which may be related to walnut embryos development and differentiation. Some TFs (ABI3, LEC1, LEC2, FUS3, and WRI1) are considered key regulators of lipid biosynthesis and oil accumulation in many plant species. In the transcriptome analysis, 1FUS3, 2 ABI3s, 2 LEC2s, and 4 WRI1s were identified, whereas LEC1 TFs were not detected, suggesting that the lipid synthesis was not regulated by these TFs in mature walnut embryos (S4) (Supplementary Table S30). ABI3 is involved in seed development and dormancy, in particular, in fatty acid metabolism [44, 45]. Four ABI3 TFs showed high expression in the S4 stage consistent with the oil accumulation pattern, which suggested a role in the regulation of lipid biosynthesis in mature walnut embryos. WRIl and FUS3 are expressed at a high level during the S2 and S3 stages; this suggested a minor role in lipid biosynthesis and oil accumulation. To further investigate the correlation between the identified genes and lipid biosynthesis, an analysis was performed using Pearson’s correlation. One ABI3 transcription factor was highly correlated with the eicosadienoic acid (C20:2) (r =

−0.839). Furthermore, we identified one ABI3 gene that was negatively correlated with several glycerides (r = −0.828), lysophatidylethanolamine (r = −0.844) and lysophatidylcholine (r = −0.807), suggesting that an ABI3 interacting protein is an inhibitor or plays a different negative role in lipid biosynthesis.

Discussion

Third-generation sequencing is a powerful technology, which that will help accelerate genetic improvement for many crop species [46]. Whole-genome sequencing has been performed for many plants, and it has provided useful genomic information for functional gene mining, genetic linkage map construction, quantitative trait analysis, and molecular breeding [47, 48]. Manchurian walnut is widely distributed in northeast China and utilized for its edible kernels, superior wood characteristics, and medicinal value of its secondary products. Research on this species has been limited, and genome-level studies are lacking. This study provides a high-quality and chromosome-level reference genome sequence for molecular breeding and evolutionary studies in the Juglandaceae. We used long (PacBio) HiFi reads, next-generation sequencing, and Hi-C scaffolding to sequence and assemble the genome of *J. mandshurica*, thereby providing new insights and valuable genetic information on juglone and lipid biosynthesis.

In the present study, we report the highest-quality genome assembly for *J. mandshurica* to date, with the longest contig N50 of 21 Mb and the highest genome completeness of 98.3% in terms of BUSCO results. Compared with previous studies on genome assemblies for this species, our contig N50 size (21 Mb) for *J. mandshurica* was improved by 187-fold (N50 was 0.1 Mb) [49] and 3-fold (N50 was 6 Mb) [50]. Our complete BUSCO score was 1,588, which was considerably higher than that of a recent genome assembly (1,375) [50]. After sequencing and assembly, our assembled genome was 548.7 Mb, which was slightly higher than that reported by Yan et al. (548.5 Mb), but lower than that of Stevens et al. (580 Mb) [49]. We obtained 40,453 protein-coding genes, which was substantially higher than those obtained by Yan et al. (29,032 protein-coding genes) [50]. Using the sequence platform of WGS-PacBio Sequel II, we obtained 14.62 Gb of genome sequence; however, the previous genome sequence was 101 Gb in size and was obtained using the Nanopore sequencing platform. The GC content and repeated sequences were 36.72% and 0.33 Gb (60.99%), which were relatively lower than the repeats reported by Yan et al. (38.5% GC content and 0.34 Gb [62.08%] repeat sequences). Our chromosome-scale genome maintained a relatively high assembly quality, thereby providing valuable information for further analysis of evolution and mining of functional genes in *J. mandshurica*.

Currently, nine species in the Juglandaceae family have been sequenced for whole-genome information, consequently yielding a mass of high throughput sequence data and contributing to understanding the evolution of Juglandaceae species [51]. Based on 558 single-copy orthologs from 15 plant genomes (9 of which were from the Juglandaceae family), genome-level studies were performed to update the evolutionary relationships between *J. mandshurica* and its related species. The Juglandaceae species maintain close genomic relationships, and as observed in the present study, all of the

Juglandaceae species group into the same cluster, consistent with the morphology-based plant taxonomy [51]. In which, for the relationship of *J. mandshurica* and *J. cathayensis*, it is still controversy at present. In Flora of China, *J. mandshurica* was consider as a synonym of *J. cathayensis* according to the phenotypic characteristic, but there is no definitive molecular evidence at the genome level. Here, although the phylogenomic analysis showed that the *J. mandshurica* was closest to *J. cathayensis*, they possess a relatively larger divergence time (approximately 13.8 mya), similar results were found in previous study [52]. Also, the synteny analysis and genome-wide alignment between *J. mandshurica* and *J. cathayensis* in present study was performed by jcvi [53] and Last [54] software, respectively (Supplementary Fig. S23 and Table 2). The results showed that the number of protein coding genes in *J. mandshurica* (40,453) was similar to that in *J. cathayensis* (39,905). However, the synteny analysis found that approximately 60% of the *J. mandshurica* genome was colinear with *J. cathayensis*. Additionally, the alignment analysis suggested that the obtained identity genes (identity >=90%) approximately was accounted for ~66% of genome. Thus, we preliminarily speculated that they were eventually established as two separate species. WGDs offer the evolutionary potential to generate new functions in plant species [55]. Particularly, in a previous study, the Ks distribution results showed that the main peak of Ks was nearly 0.3, and the Juglandales appeared from the Cretaceous to Paleocene at the periods (56~66 mya) according to the fossil evidence, and there was a Juglandoid WGD for walnut species [56, 57]. In the present study, for comparative genomics, the main peak was also approximately 0.3. Therefore, we indicated that there was a Juglandoid WGD that originated before the radiation of Juglandaceae in the Paleocene, and similar results were also found in a recently reported *J. mandshurica* assembly [50]. In the collinearity comparison with *P. trichocarpa*, many syntenic blocks were identified, which indicated the presence of a close genetic relationship between *J. mandshurica* and *J. regia*, and a more distant relationship with *P. trichocarpa*.

Table 2 The genome comparison of *J. mandshurica* and *J. cathayensis*

Item	*Juglans mandshurica*	*Juglans cathayensis*
Total genome size(bp)	548,694,591	493,089,748
Scaffold N50(bp)	35,382,463	31,976,116
Contig N50(bp)	21,388,210	23,436
Gene number	40,453	39,905
Synteny genes (by jcvi)	25,402	24,016
Genes identity (>=90%) (by last)	26,782	28,067
Genes identity (>=80%) (by last)	31,008	31,655
Genes identity (>=70%) (by last)	33,881	33,927

The Juglandaceae family is an economically important species that has been planted and domesticated since the Han dynasty (206 BC–220 AD). Fruits of many species in this family are rich in medicinal components in the walnut exocarp and the oils in the kernel, particularly for species in the *Carya* and *Juglans* genera [58, 59]. The regulatory mechanism of fruit development/differentiation and the biosynthesis of juglone and lipids in *J. mandshurica* remain largely unknown. Previous studies identified a small number of genes affecting the bioactivity of juglone in walnut roots

and leaves, but without knowledge of the fact that these genes were related to juglone accumulation in fruits, particularly in the walnut exocarp [14, 15]. Therefore, it is necessary to further investigate the potential genes involved in controlling juglone biosynthesis during developmental fruit. Reportedly, genes involved in juglone biosynthesis are partly derived from the phylloquinone pathway. In this pathway, DHNA plays a key role of as an intermediate for regulating the juglone and phylloquinone biosynthesis. In the present study, for walnut exocarp differentiation, we combined genomic, transcriptomic, and metabolomic data to construct a gene-–metabolite network, thereby identifying genes and TFs that may encode decarboxylases, *2-ODDs*, and *CYP450s* involved in juglone biosynthesis, which fills up. This consequently helped to fully elucidate the blanks of mechanism underlying the juglone accumulation in fruits and indicated a new step to identify the juglone pathway genes in all tissues. Particularly, 146 key genes were identified in juglone biosynthesis from the DEGs in our *J. mandshurica* transcriptome, 125 of which were *CYP450* genes. Therefore, CYP450 superfamily members play a crucial role in juglone accumulation and could catalyze the reactions from 1,4-NQ to form juglone; these results were similar to those reported in a previous study [14]. Expression analysis showed that the obtained *CYP450* genes maintain a high expression level during the differentiation of walnut exocarp, which would contribute to juglone accumulation. This result is similar to the secondary metabolite biosynthesis in plants such as *Aconitum vilmorinianum* (aconite) [60], *Salvia miltiorrhiza* (red sage) [61], *Scutellaria baicalensis* (Chinese skullcap) [62], and *Aralia elata* (angelica tree) [63]. Additionally, we identified the expanded CYP450 gene family from the genome assembly of the *J. mandshurica*, suggesting that they may specifically affect juglone biosynthesis. Among the 14 *CYP450* genes, 5 were correlated with juglone ($r > 0.8$ or < -0.8) based on the co-expression network analysis, and can be considered candidate genes for further study on juglone biosynthesis. Although some genes in the juglone biosynthetic pathway have been identified, the regulators remain unknown. In a previous study, the expression of TFs including, such as AP2/ERF, NAC, HSF, WRKY, MYB, C2H2, and GRAS was, changed significantly changed under juglone treatment [64]. In this study, the common TFs including AP2/ERF-ERF, bHLH, bZIP, C2H2, MYB, and NAC were identified in four comparative groups during developmental walnut exocarp. Particularly, all the potential TFs including bZip, NAC, NF-YA, and NF-YC are positively correlated with the abundance of juglone and may participate in juglone biosynthesis, combining comparative transcriptomics and metabolic profiling. Additionally, we found that one NAC (*gene-Jman002G0279700*, $r = 0.957$) and one bZip TFs (*gene-Jman003G0271600*, $r = 0.952$) showed relatively high correlation with juglone, and they could be used as key candidate regulators to identify the target genes in juglone pathways. These results may be valuable for further studies on juglone biosynthesis and its agricultural uses.

In addition to the study of the walnut exocarp, we used the developing/differentiating kernel to identify the genes associated with oil accumulation in *J. mandshurica*. Lipids were the dominant secondary metabolites, and free fatty acids were enriched in *J. mandshurica* embryos. Some free fatty acids (linoleic, stearic,

arachidic, α-linolenic, and γ-linolenic acids) were the most abundant during the S1 to S4 stages; these results were similar to those reported in a previous study [37]. Long-chain acyl-coenzyme A synthetase (LACS) is one of the key enzymes in fatty acid metabolism, which can catalyze acyl-coenzyme A synthesis and contribute to TAG assembly. In *Helianthus annuus, HaLACS1* overexpression can effectively increase seed oil content [65]. Expression analysis of *BnLACSs* genes showed that they were involved in fatty acid biosynthesis in *Brassica napus* [66]. Therefore, the *LACS* gene expression level was closely related to seed oil synthesis. In this study, the corresponding genes (*PAP*, *LACS*, and *DGAT*) involved in lipid biosynthesis also show high abundance during embryo development, suggesting that these genes and metabolites play key roles in lipid synthesis for oil accumulation. TAG is mainly stored in the cytoplasm in the form of oil bodies after synthesis, and Oleosin is the main protein regulating the structure and function of oil bodies. Reportedly, three Oleosin genes in sesame oil were confirmed to be transcribed in mature seeds, thereby maintaining the structural stability of the oil body [67]. In *Lilium longiflorum*, oil bodies in pollen are mainly protected by special oleosin proteins [68]. Additionally, in the plant kernels with a relatively high oleosin content, the oil volume was relatively smaller [69]. In the present study, expression patterns of the eight oleosin genes obtained were nearly consistent and showed high efxpression levels during the embryo's late developmental stage. Therefore, we speculated that these oleosin proteins played an important role in maintaining oil body stability in *J. mandshurica*. TFs are important regulators for plant growth and development. In addition, some TFs (MYB, C2H2, AP2/ERF-ERF, and NAC) were differentially expressed during embryo development. Such regulation of TFs implies that they may play important roles in lipid biosynthesis in *J. mandshurica*, similar to results found in previous studies [70, 71]. For lipid synthesis, several TFs regulate the structural genes and their accumulation, including some positive (ABI3, FUS3, LEC2, AGL15, and WRI1) and negative (MYB76, MYB118, MYB89, GL2, and WRKY6) regulatory factors [72]. Among them, ABI3, FUS3, and WRI1 have been identified in many plants and can regulate the expression of functional genes involved in fatty acid synthesis, thereby regulating seed oil accumulation. Transcriptome analysis of walnut kernel development found that *WRI1* was involved in lipid biosynthesis and polyunsaturated fatty acid metabolism [73]. In *Torreya grandis*, *TgWRI1*, and *TgFUS3* are involved in the regulation of genes related to lipid biosynthesis during developmental seed [74]. Herein, the authors identified some of the TFs involved in lipid biosynthesis. These TFs (FUS3, ABI3, LEC2, and WRI1) were correlated with lipid metabolites in *J. mandshurica*, similar to a previous study [75-77]. These results established the foundation for identification to help identify genes encoding enzymes that catalyze the formation of fatty acid and oil body formation; thus, these results will be valuable in the useful for future for studies involved in engineering of lipid biosynthesis.

Methods

Plant Materials and DNA sequencing

Fresh leaves of adult *J. mandshurica* were collected at the campus of Northeast Forestry University (126°37'57.28" E, 45°43'6.53" N), in Harbin, Heilongjiang province, China. High quality genomic DNA from fresh leaves was extracted by an improved CTAB method [78]. For long-read DNA sequencing, 15 μg of sheared DNA was used for circular consensus sequencing (CCS, RRID:SCR_021174). The SMRT Bell HiFi libraries were constructed as followers: (1) DNA was subjected to enzymatic reaction to remove prominent single-stranded ends; enzymatic reaction was carried out to remove the prominent single-chain end and repair the DNA damage; (2) after that, A was added at the end of the double chain to repair DNA terminal; (3) The T-overhang of the SMRT bell adapter was ligated with the end at 20°C for 15 h, and the library was purified with 1x AMPure PB after the connection was completed; (4) determination of concentration and fragment size distribution of samples in library were completed by FEMTO Pulse automatic pulse field capillary electrophoresis and Qubit 3.0 fluorescence detector (Life Technologies， Carlsbad，CA，USA); BluePippin system was used to select fragment size, the DNA randomly cut into ~15 Kb fragments; the obtained libraries were purified using 1x AMPure PB; (5) the size and quality of the library were evaluated using FEMTO Pulse and Qubit dsDNA HS detection kits; (6) The sequencing primers and Sequel II DNA polymerase were annealed respectively, and they were used for combing with the final SMRTbell library; (6) after library construction, sequencing was performed on PacBio Sequel II platform (PacBio Sequel II System, RRID:SCR_017990) (14.62 Gb data, 26-fold coverage of the genome) at Frasergen Bioinformatics (Wuhan, China) at a concentration of 120 pM, and the running time was 30 h. For short-read DNA sequencing, libraries were constructed from 300–500 bp fragments and sequenced using the WGS Illumina HiSeq 2500 platform (Illumina HiSeq 2500 System, RRID:SCR_016383). Totally, 56 Gb of raw data were obtained.

Genome k-mer analysis

The quality filtered long reads were used for genome size estimation. In present study, we calculated the frequency of each 17-mer from the HiFi sequencing reads (14.62 Gb) and examined the distribution of the 17-mer numbers. Then, we estimated the genome size to be about 547.99 Mb, and the proportion of repeat sequences and heterozygosity rate of the genome were determined to be approximately 48.78% and 0.77%, respectively, using GCE software v1.0.2 (GCE, RRID:SCR_017332) [79].

RNA extract and Iso-Seq Sequencing

To obtain high-quality annotation results, Iso-seq sequencing was implemented in this study. The bark fruit, and leaves of *J. mandshurica* were collected, frozen in liquid nitrogen immediately, and stored in a refrigerator at −80 °C for Iso-seq sequencing. The total RNA was obtained by the TRIzol reagent (Invitrogen, Carlsbad, CA, United States). RNA purity was checked using the kaiaoK5500®Spectrophotometer (Kaiao, Beijing, China). RNA integrity and concentration was assessed using the RNA Nano 6000 Assay Kit of the Bioanalyzer 2100 system (Agilent 2100 Bioanalyzer Instrument,

RRID:SCR_019389; Agilent Technologies, CA, USA). Firstly, full-length cDNA of mRNA was synthesized by Clonetech SMARTer PCR cDNA Synthesis Kit. The obtained full-length cDNA was amplified by PCR, and the amplified product was purified by PB magnetic beads, and some small fragment cDNAs below 1 kb were further removed. The end-repair of products was implemented, and the adapter with SMRT dumbbell was added to the end of cDNA. The unconnected fragments were digested by exonuclease, and the fragments were purified by PB magnetic beads to obtain sequencing library. Qubit 3.0 was used for accurate quantification and Agilent 2100 was used for library size detection to obtain the high-quality library. After that, the samples were sequenced on the PacBio Sequel II platform, and 53 Gb raw data were obtained.

Genome assembly

Here, we obtained approximately 14.62 Gb (26 x of the genome) sequencing data on the PacBio Sequel II platform. The raw data obtained in the PacBio Sequel II CCS sequencing mode was converted to HiFi data by CCS software using the parameters '-minPasses 3'. Then, these HiFi data (~15 Kb long reads) were further assembled using Hifiasm v.0.2.0 (Hifiasm, RRID:SCR_021069) [16] software with default parameters to get a preliminary assembly genome. Additionally, the gfatools [80] was employed to obtain the sequence graphs with FASTA format. The Hi-C sequencing was performed on an Illumina HiSeq platform with PE 150 bp and yielded 51 Gb of sequence. Throughout the construction of the Hi-C library and sequencing, we used HTQC v1.92.310 (HTQC, RRID:SCR_006448) [81] software to perform quality control on the raw data and obtained clean data. For clean data, BWA (BWA, RRID:SCR_010910) [82] software (0.7.17) was used for comparison to different contig, and then used for Hi-C associated scaffolding. Firstly, the low-quality reads with self-ligation and non-ligation were removed and filtered. Totally, 657 contigs were successfully classified into 16 chromosome groups using the agglomerative hierarchical clustering method in Lachesis software (LACHESIS, RRID:SCR_017644) [17], and the clustered contigs were further ordered and oriented. The final reference assembly contained 16 chromosome-scale pseudomolecules, with maximum and minimum lengths of 52 Mb and 19 Mb, respectively. A heat map of the interaction matrix of all pseudochromosomes was plotted with a resolution of 500 kb. The assembled chromosome number is the same as the haploid chromosome number of *J. mandshurica* (n = 16) (Fig. 1a).

Evaluation of assembly results

After assembly was completed, we evaluated the results using three methods. First, the comparison tool minimap2 v2.5 (Minimap2, RRID:SCR_018550)[83] was used to compare the assembled genomes, and the comparison rate of reads, the extent of genome coverage and the distribution of depth were calculated to evaluate the integrity of the assembly and the uniformity of sequencing coverage. Secondly, the Burrows-Wheler Aligner (BWA) [82] was used to compare reads to the reference genome. Finally, based on the single-copy homologous gene set in OrthoDB (OrthoDB,

RRID:SCR_011980) [84], BUSCO v5.2.2 (BUSCO, RRID:SCR_015008) [85] was used to predict these genes and calculate their integrity, fragmentation, and possible loss rates. BUSCO assessment indicated that 98.3% of the complete genes were captured.

Genome annotation

We used homologous and *de novo* annotation to identify repetitive sequences. Firstly, RepeatMasker (Open-4.0.9) (RepeatMasker, RRID:SCR_012954) [86] and RepeatProteinMask (Open-4.09) [86] were used to search for TE sequences from Repbase (release 21.01) (Repbase, RRID:SCR_021169) [87] based on homology. Secondly, RepeatModeler (Open-1.0.11) (RepeatModeler, RRID:SCR_015027) [88] and LTR-Finder v1.0.7 [89] were used to construct a repeat sequence database, and then Repeatmasker (Open-4.09) [86] was used to identify the repetitive sequences. TRF [90] was used to identify tandem repeat sequences. Finally, the results based on homologous annotation and *de novo* annotation were integrated and the non-redundant elements after overlapping, were removed for the final repeated sequence annotation.

We used homologous, *de novo* and transcriptome assisted annotation to predict the structure and function of coding genes. For homologous annotation, 3-5 related species were selected, and then TblastN (TBLASTN, RRID:SCR_011822) [91] (E-value cutoff of 1e-5) was used to compare the related species to the reference genome. Then, the aligned sequences and their corresponding proteins were filtered and transmitted to the Exonerate [92] for accurate alignment. Augustus (v3.3.1) [93] and GlimmerHMM v3.0.4 (GlimmerHMM, RRID:SCR_002654) [94] were used for *de novo* annotation. For isoform-sequencing (Iso-Seq) data, we used Gmap (GMAP, RRID:SCR_008992) [95] to align it to the reference genome, and then used TransDecoder (TransDecoder, RRID:SCR_017647) [96] to predict open reading frames in the transcripts to define putative coding sequences. Maker v3.00 (MAKER, RRID:SCR_005309) [19] was used to integrate the predicted gene sets into a non-redundant, more complete and reliable gene set. Finally, the proteins in the gene collection were annotated by means of curated protein databases including SwissProt, TrEMBL, KEGG, GO and NR using NCBI BLASTP v2.6.0+ (BLASTP, RRID:SCR_001010) (*E*-value ≤1e−5) [97].

In the annotation process of non-coding RNA, according to the structural characteristics of tRNA, tRNA sequences in the genome were searched using tRNAscan-SE v1.3.1 (tRNAscan-SE, RRID:SCR_010835) [98]. Because rRNA is highly conserved, rRNA sequences from related species can be selected as reference sequences to search for rRNA by BLASTN v2.6.0 (BLASTN, RRID:SCR_001598) [97] alignment. Infernal of Rfam (Rfam, RRID:SCR_007891) [99] was used to predict miRNA and snRNA sequences in the genome.

Phylogenomic reconstruction and gene family evolution

To identify the gene families in each species, we clustered the proteins of 15 species through the OrthoMCL (V14-137) [28] process based on sequence similarity with the parameter of "-inflation 1.5.", including 13 rosid species (i.e., *J. cathayensis* [100], *J. macrocarpa* [100], *J. nigra* [100], *J. regia* [100], *J. hindsii* [100], *J. sigillata* [100] *C.*

cathayensis [101], *C. illinoinensis* [101], *Quercus lobata* (valley oak) [102], *Castanea mollissima* (Chinese chestnut) [103], *Morella rubra* (red bayberry) [104], *P. trichocarpa* [105] and *V. vinifera* [106] and one commelinid species (*O. sativa*) [107] . Muscle v3.8.31 (MUSCLE, RRID:SCR_011812) [108] was used to conduct multiple sequence alignments of genes within the single-copy homologous gene family of each species, and RAxML v8.2.12 (RAxML, RRID:SCR_006086) [29] was used to construct an evolutionary tree using Maximum Likelihood.

We utilized the constructed evolutionary tree, along with the TimeTree (TimeTree, RRID:SCR_021162) website and studies in the literature to obtain time correction points, using the R8S v1.71 (r8s, RRID:SCR_021161) [109]and mcmctree v4.9e in the PAML software (PAML, RRID:SCR_014932) [110], the bifurcation time was estimated with five corrected divergence time point from the TimeTree website as follows: *O. sativa* vs. *J. mandshurica* (115–308 Mya), *V. vinifera* vs. *J. mandshurica* (107–135 Mya), *P. trichocarpa* vs. *J. mandshurica* (101–131Mya), *Q. lobata* vs. *C. mollissima* (6–49 Mya), *Q. lobata* vs. *J. mandshurica* (51–87 Mya), and two corrected divergence time point from *M. rubra* genome article [55]: *M. rubra* vs *Juglans* genus (28–34 Mya) and *Carya* genome article [101]: genus *Juglans* vs. genus *Carya* (~23 Mya). CAFÉ (CAFE, RRID:SCR_005983) [111] was used to simulate the expansion and contraction events of gene families in each lineage of the evolutionary tree.

Analyses of genome synteny and whole-genome duplication (WGD)

J. regia and *P. trichocarpa* genomes were selected as comparisons for collinearity analysis with the *J. mandshurica* genome. MCscan (MCScan, RRID:SCR_017650) [53] was used to perform synteny searches, with at least thirty gene pairs required in each syntenic block. TBtools [112] was subsequently used to visualize the schematic diagram. Mummer v4.0.0beta2 (MUMmer, RRID:SCR_018171) [113] was used to estimate the collinearity between the genomes of *J. regia* and *J. mandshurica*.

We used MCscan (MCScan, RRID:SCR_017650) to search for collinear regions in species genomes, and calculated the 4dTV of gene pairs contained in the collinear regions to reflect the relative differentiation events and whole-genome duplication in the evolutionary history of *J. mandshurica*. We used the Codeml program of the PAML package to calculate the Ks of *J. mandshurica* syntenic blocks [110]. Synteny analysis on three species including *J. mandshurica, J. regia*, and *J. sigillata* was performed to confirm the WGD event.

RNA-seq sequencing and data analysis

RNA sequencing was performed by sampling different ripening stages of the *J. mandshurica* fruit at 30 (S1 stage, without hard kernel), 50 (S2 stage, without hard kernel), 70 (S3 stage, with a hard kernel), and 90 days (S4 stage, with a hard kernel) after natural pollination (Supplementary Fig. S24). Samples were frozen in liquid nitrogen immediately and each stage contained three biological replicates. he walnut exocarp and embryos from fruits of each stage were collected and used for RNA sequencing (Supplementary Fig. S25). A plant total RNA extraction kit (Takara, Beijing, China) was used to extract total RNA from the walnut exocarp and the embryos. RNA

(1 μg) of each sample was used to construct a cDNA library. Sequencing libraries were generated using TruePrep Flexible DNA Library Prep Kit for MGI (Vazyme, Nanjing, China) following the manufacturer's recommendations and index codes were added to attribute sequences to each sample. Briefly, mRNA was purified from total RNA using poly-T oligo-attached magnetic beads. Fragmentation was performed using divalent cations under elevated temperature in NEBNext First Strand Synthesis Reaction Buffer (5X). Random hexamer primer and RNase H were used for cDNA first-strand synthesis. Then, second-strand cDNA synthesis was performed using the buffer, dNTPs, DNA polymerase I, and RNase H. The library fragments were purified with QiaQuick PCR kits and elution with EB buffer, followed by terminal repair; the A-tailing and adapter added were implemented. Each library was completed after retrieving the target products and performing PCR. Finally, 24 libraries were constructed for RNA-seq, 12 of which were from the developing walnut exocarp, and the remaining were from developing embryos; these high-quality libraries were then sequenced using Illumina HiSeq 2500 platform with paired-end reads.

After sequencing and filtering, we obtained 99.65 Gb of clean data for walnut exocarp and 107.38 Gb of clean data for embryos, respectively (Table S20). Filtered high-quality clean reads were aligned with the genome assembly of *J. mandshurica* using HISAT2 v2.1.0 (HISAT2, RRID:SCR_015530) [114] with the default parameters. Analysis of gene transcript abundance was performed using featureCounts (featureCounts, RRID:SCR_012919) [115] using the RNA-seq and Expectation Maximization software [116]. DEseq2 software (DESeq2, RRID:SCR_015687) [117] was used to detect DEGs. DEGs were screened based on the $|\log_2\text{Fold Change}| \geq 1$, and adjusted P-value < 0.05. The TFs in *J. mandshurica* were detected using iTAK [118] and PlantTFDB (PLANTTFDB, RRID:SCR_003362) [119].

Metabolomics

We collected fresh and healthy fruits including the walnut exocarp and embryos at four different stages, each stage contained three biological replicates. The walnut exocarp and embryos were collected to extract metabolites, respectively. First, all collected samples were immediately loaded into a precooled centrifuge tube and frozen with liquid nitrogen. All the samples were then freeze-dried and crushed into a powder before using the mixer mill (MM 400, Retsch) with zirconia beads for 1.5 min at 30 Hz. In this process, 100 mg of the powder was dissolved in 1.2 mL of 70 % methanol extract. The extracts were vortexed for 30 s each (30 min with six repeats), and all were stored overnight at 4℃. The solution above was centrifuged at 12,000 rpm for 10 min, the supernatant was collected and filtered with a microporous membrane (0.22 μm) and stored in an injection bottle for ultra-performance liquid chromatography/tandem mass spectrometry (UPLC–MS/MS) analysis. The UPLC–MS/MS analysis was implemented using multiple reaction monitoring (MRM) by the Wuhan MetWare Biotechnology Co., Ltd., (Wuhan, China). Particularly, linear ion trap (LIT) and triple quadrupole (QQQ) scans were obtained from AB 4500 Q TRAP UPLC–MS/MS system that was equipped with an ESI Turbo Ion-Spray interface on Analyst 1.6.3 software (AB Sciex) and operated in the positive ion mode. The detailed ESI operation

parameters were as follows: ion source, turbine spray; source temperature, 550℃; ion spray voltage (IS), 5500 V (positive ion mode) or −4500 V (negative ion mode); ion source gas I (GSI), gas II (GSII) and curtain gas (CUR) were set at 50, 60, and 25.0 psi, respectively, and parameters of collision-induced ionization are set at a high level; tuning and quality calibration of the instrument were performed under QQQ and LIT mode with 10 and 100 μmol/L polypropylene glycol solution, respectively; QQQ scan was performed using MRM experiments, and the collision gas (nitrogen) was set at medium; DP and CE for each MRM ion pair were obtained according to further DP and CE optimization; and according to the metabolites eluted in each period, a set of specific MRM ion pairs were monitored from this period. The mass spectrum data above was used for qualitative and quantitative analysis based on the MetWare database (MWDB) of MetWare Biotechnology Co., Ltd. (Wuhan, China) to obtain the original metabolite data. For quality control (QC) analysis, one quality control sample was inserted into each of the ten test and analysis samples to monitor the repeatability of the analytic process. Partial least-squares discriminant analysis (OPLS-DA) was employed to screen variation components. The detailed methods were described in previous studies. The differentially expressed metabolites (DEMs) were screened based on the |log2Fold Change | ≥1 or *P*-value <0.05, and variable importance in project (VIP) ≥ 1. To study the specific accumulation of metabolites, we performed principal component analysis (PCA) of the metabolites that underwent a significant degree of changes using R [120]. The correlations between the differentially expressed genes and the metabolites were performed based on the Pearson correlation with the correlation coefficient at r > 0.8 or < −0.8. Correlation networks were used to visualize the relationships between the genes and metabolites using igraph package at OmicStudio tools [121].

Functional gene analysis

To identify the *CYP450* genes related to juglone biosynthesis, we downloaded all annotated Arabidopsis CYP450 proteins from the TAIR database. The CYP450 family proteins of *Arabidopsis thaliana* were used as seed sequences, and the whole genome of *J. mandshurica* was searched using BLASTP [97] with the E value $\leq 1e^{-5}$. All candidate sequences were screened for the conserved CYP450 domain using Swissport and Batch NCBI CD-Search Tools (Batch Web CD-Search Tool, RRID:SCR_018756) . In the expanded gene families of *J. mandshurica*, 14 *CYP450* genes were identified using the Upset process of TBtools and selected for phylogenetic analysis [122]. We constructed a phylogenetic tree to classify the members of the expanded CYP450 gene family in *J. mandshurica* and those related to *C. illinoinensis*, *C. cathayensis*, and *J. cathayensis*. All candidate sequences were compared using ClustalW (ClustalW, RRID:SCR_017277) in MEGA 7.0 (MEGA Software, RRID:SCR_000667) [119] software using the default parameters. Redundant genes were manually removed, and all non-redundant genes were used for further analysis.

Data Availability

Raw reads used for genome assembly of *J. mandshurica* has been uploaded to the National Center for Biotechnology Information (NCBI) Sequences Read Archive (SRA)

with the accession numbers: SRR14637189 and SRR14629954. Transcriptomic data have been deposited in SRA with the accession number of PRJNA733587 (including embryos and exocarp). The assembled *J. mandshurica* genome has been deposited in the Genome Warehouse in National Genomics Data Center (NGDC) under accession number PRJCA006358. Third generation transcriptomic data have been deposited in BIG Data Center under accession number PRJCA006794. The metabolomics data in this study were deposited and available at Metabolights repository under the accession numbers MTBLS3657 [123]. All supporting data and materials are available in the *GigaScience* GigaDB database [124].

Additional files

Supplementary Figure 1. 17-mer analysis to estimate the *J. mandshurica* genome size.
Supplementary Figure 2. Sequencing depth distribution of the assembled *J. manshurica* genome
Supplementary Figure 3. Cross-species comparisons of exon number, intron number, gene length, gene GC, CDS GC, exon length, CDS length and intron length distribution
Supplementary Figure 4. Upset plot of genes annotated in GO, InterPro, KEGG, NR, Swissprot and TrEMBL database.
Supplementary Figure 5. The GO category analysis of rapidly expanded gene families in assembly *J. mandshurica* genome
Supplementary Figure 6. Schematic representation of syntenic genes among *J. mandshurica* and *J. regia*
Supplementary Figure 7 Genomic variation between *J. mandshurica* and other two walnut species.
Supplementary Figure 8. PCA score plot metabolite profiles from different sample groups during developmental walnut exocarp
Supplementary Figure 9. PCA score plot metabolite profiles from different sample groups during developmental walnut embryos
Supplementary Figure 10. Distribution of identified metabolites in green peel in *J. mandshurica*
Supplementary Figure 11. Venn diagrams of differentially expression genes (DEGs) in S1, S2, S3 and S4 stages in green peel in *J. mandshurica*
Supplementary Figure 12. GO enrichment analysis of 897 core DEGs in *J. mandshurica*
Supplementary Figure 13. KEGG enrichment analysis of 897 core DEGs in *J. mandshurica*
Supplementary Figure 14. GO enrichment analysis of 990 DEGs identified in cluster 6
Supplementary Figure 15. KEGG enrichment analysis of 990 DEGs identified in cluster 6
Supplementary Figure 16. Upset plot of differentially expression genes associated with Juglone ($r > 0.8$ or < -0.8) in S1, S2, S3 and S4 stage in *J. mandshurica*
Supplementary Figure 17. KEGG enrichment analysis of 566 core DEGs associated with Juglone ($r > 0.8$ or < -0.8) in S1, S2, S3 and S4 stage in *J. mandshurica*

Supplementary Figure 18. Distribution of identified metabolites in walnut kernels in *J. mandshurica*

Supplementary Figure 19. Distribution of identified lipid components in walnut kernel in *J. mandshurica*

Supplementary Figure 20. Heatmap of the free fatty acids during walnut kernel development in *J. mandshurica*

Supplementary Figure 21. KEGG enrichment analysis of different metabolites in six comparison groups. (a) S1 vs S2 (b) S1 vs S3 (c) S1 vs S4 (d) S2 vs S3 (e) S2 vs S4 (f) S3 vs S4

Supplementary Figure 22. Heatmap of the free fatty acids during walnut kernel development in *J. mandshurica*

Supplementary Figure 23. The schematic representation of syntenic genes among *J. mandshurica* and *J. cathayensis*.

Supplementary Figure 24. Changes of *J. mandshurica* fruits in different development periods. S1-S4 indicate the fruit collected at 30 days (S1 stage), 50 days (S2 stage), 70 days (S3 stage) and 90 days (S4 stage) after natural pollination.

Supplementary Figure 25. The tissue structure of *J. mandshurica* fruit including the walnut exocarp and walnut embryos.

Supplementary Table 1. The statistics of K-mer analysis

Supplementary Table 2. BUSCO evaluation results for *J. mandshurica* genome

Supplementary Table 3. Statistics of genome alignment in *J. mandshurica*

Supplementary Table 4. Statistical analysis of SNP types in t *J. mandshurica* genome

Supplementary Table 5. The information of transcriptome of walnut exocarp and embryos

Supplementary Table 6. Statistical results of genetic structure of related species

Supplementary Table 7. Summary of the functional annotation in *J. mandshurica* genome

Supplementary Table 8. Non-coding genes in the *J. mandshurica* genome

Supplementary Table 9. Statistics of transposable elements and other repeats in *J. mandshurica* genome

Supplementary Table 10. Repeat sequence classification results statistics in *J. mandshurica* genome

Supplementary Table 11. Repeat elements in *J. mandshurica* genome

Supplementary Table 12. Comparison of the estimated number of gene families of *J. mandshurica* with other plants

Supplementary Table 13. Summary of gene ortholog analysis conducted on 16 sequenced genomes

Supplementary Table 14. The KEGG enrichment of expanded gene family

Supplementary Table 15. The KEGG enrichment of contracted gene family

Supplementary Table 16. Table S16 Statistical result of Genomic variation between *J. mandshurica* and other two walnut species.

Supplementary Table 17. Table S17 The KEGG enrichment of obtained translocation events between *J. mandshurica* and *J. macrocarpa*

Supplementary Table 18. Table S18 The KEGG enrichment of obtained translocation

events between *J. mandshurica* and *J. regia*

Supplementary Table 19. Genes encoding enzymes related to juglone biosynthesis

Supplementary Table 20. The genes of rapidly expanded CYP450 gene family in *J. mandshurica* and its related species

Supplementary Table 21. The identified metabolites isolated from walnut exocarp of *J. mandshurica*

Supplementary Table 22. The differentially accumulated metabolites (DAMs) during different developmental stage of walnut exocarp

Supplementary Table 23. The differentially expressed genes during different developmental stage of walnut exocarp

Supplementary Table 24. The number of transcription factors in different compare groups

Supplementary Table 25. The differentially expressed genes correlated with the Juglone ($r > 0.80$ or < -0.8)

Supplementary Table 26. The gene-to-metabolite correlation coefficient

Supplementary Table 27. The identified metabolites isolated from walnut embryo of *J. mandshurica*

Supplementary Table 28. The identified metabolites involved in lipid in embryo of *J. mandshurica*

Supplementary Table 29. The transcription factors identified during the developmental embryo

Supplementary Table 30. The transcription factors involve in the lipid synthesis and oil accumulation

Abbreviations

BLAST: Basic Local Alignment Search Tool; bp: base pair; BUSCO: Benchmarking Universal Single-Copy Orthologs; DEGs: differentially expressed genes; DAMs: differentially accumulated metabolites; GATK: Genome Analysis Tool Kit; Gb: gigabase pairs; GO: Gene Ontology; HiFi: high fidelity; kb: kilobase pairs;KEGG: Kyoto Encyclopedia of Genes and Genomes; Ks: synonymous substitutions per synonymous; Mb: megabase pairs; mRNA: messenger RNA; mya: million years ago; NCBI: National Center for Biotechnology Information; NGDC: National Genomics Data Center; PacBio: Pacific Biosciences; TFs: transcription factors; TEs: transposable elements; VIP: variable importance in project; WGD: whole-genome duplication.

Competing interests

The authors declare no competing interests.

Funding

This research study was supported by the Scientific research start-up funds of Jilin Agricultural University (No. 2021002), the Innovation Project of State Key Laboratory of Tree Genetics and Breeding (Northeast Forestry University) (No. 2021A01), and the Fundamental Research Funds for the Central Universities (Northeast Forestry University) (No. 2572020DR01)." The Awardee of all Funding are from X Zhao.

Authors' contributions

X.L, K.W.C, Q.H.Z and X.N.P was a major contributor in writing the manuscript; Z.M.H, S.C.1, L.P.J, M.H.Z and Y.L contributed to plant sample collection, DNA/RNA preparation, library construction and sequencing; S.K.Z, X.X.Z, Y.X.L and S.C. worked on genome assembly and annotation, V.C and R.S conducted transcriptome analysis and identified functional genes involved in juglone biosynthesis. G.Z.Q and M.T analyzed the gene family and constructed the evolutionary tree. XYZ conceived of the study, participated in its design and data interpretation, and revised the manuscript critically.

Acknowledge

Thanks to the members of the College of Forestry and Grassland of Jilin Agricultural University and State Key Laboratory of Tree Genetics and Breeding for their assistance during laboratory works and for fruitful discussions. Thanks to the assistance of Wuhan MetWare Biotechnology Co., Ltd. and Frasergen Bioinformatics Co., Ltd. (Wuhan, China). We also thank Bullet Edits Limited for the linguistic editing and proofreading of the manuscript.

References

1. Bai WN and Zhang L. Nuclear and chloroplast DNA phylogeography reveal two refuge areas with asymmetrical gene flow in a temperate walnut tree from East Asia. *New Phytol* 2010;**188**(3):892-901. doi: 10.1111/j.1469-8137.2010.03407.x.
2. Zhang LJ, Guo C, Lu XJ, Sun XM, Liu CP and Zhou Q, et al. Flower development of heterodichogamous *Juglans mandshurica* (Juglandaceae). *Front Plant Sci* 2021;**12**:541163. doi: 10.3389/fpls.2021.541163.
3. Luan F, Wang Z, Yang Y, Ji Y and Zeng N. *Juglans mandshurica* Maxim.: A review of its traditional usages, phytochemical constituents, and pharmacological properties. *Front. Pharmacol* 2021;**11**:569800. doi: 10.3389/fphar.2020.569800.
4. Zhang YY, Zhang F, Zhang YS, Thakur K, Zhang JG, Liu Y, et al. Mechanism of juglone-induced cell cycle arrest and apoptosis in Ishikawa Human endometrial cancer cells. *J Agric Food Chem* 2019;**67**(26):7378-89. doi: 10.1021/acs.jafc.9b02759.
5. Mallavadhani UV, Prasad CV, Shrivastava S and Naidu V. Synthesis and anticancer activity of some novel 5,6-fused hybrids of juglone based 1,4-naphthoquinones. *Eur J Med Chem* 2014;**83**:84-91. doi: 10.1016/j.ejmech.2014.06.012.
6. Umarov AU, Burnasheva SN and Makhmudova KS. Oil from the seeds of *Juglans mandshurica* and Anabasis Aphylla. *Chem Nat Compd* 1970;**6**(2):258-9. doi: 10.1007/BF00941697.
7. Yu N, Lin L and Wang F. Extraction of manchurian walnut oil assisted by ultrasonic and fatty acid composition. *Food Sci Technol* 2014;**39**(10):184-8. doi: 10.13684/j.cnki.spkj.2014.10.039.
8. Yang H, Gan C, Guo Y, Qu L and Wang J. Two novel compounds from green walnut husks (*Juglans mandshurica* Maxim.). *Nat Prod Res* 2020;**2**:1-9. doi: 10.1080/14786419.2020.1860976.
9. Islam A and Widhalm JR. Agricultural uses of juglone: opportunities and challenges. *Agronomy* 2020;**10**:1500. doi: 10.3390/agronomy10101500.
10. Girzu M, Carnat A, Privat AM, Fialip J and Lamaison AP. Sedative effect of Walnut leaf extract and

juglone, an isolated constituent. *Pharm Biol* 2008;**36**(4):280-286. doi: 10.1076/phbi.36.4.280.4580.

11. Silva-Belmares SY, Saenz-Galindo A, Garcia JJV and Lopez-Lopez LI. Ultrasonic and microwave assisted synthesis of nitrogen-containing derivatives of juglone as potential antibacterial agents. *Lett Org Chem* 2014;**11**(8):573-82. doi: 10.2174/1570178611666140421225621.
12. Shen CC, Afraj SN, Hung CC, Barve BD and Kuo YH. Synthesis, biological evaluation, and correlation of cytotoxicity versus redox potential of 1,4-naphthoquinone derivatives. *Bioorg Med Chem Lett* 2021;**17**:127976. doi: 10.1016/j.bmcl.2021.127976.
13. Shi LL, Ma XL, Ala TTY, Chen G and Wei HY. Research progress of Qinglongyi (cortex *Juglandis mandshuricae*). *Guiding Journal of Traditional Chinese Medicine and Pharmacy* 2020;**26**(05):122-5. doi: 10.13862/j.cnki.cn43-1446/r.2020.05.031.
14. Widhalm JR and Rhodes D. Biosynthesis and molecular actions of specialized 1,4-naphthoquinone natural products produced by horticultural plants. *Hortic Res* 2016;**3**:16046. doi: 10.1038/hortres.2016.46.
15. Mccoy RM, Utturkar SM, Crook JW, Thimmapuram J and Widhalm J. The origin and biosynthesis of the naphthalenoid moiety of juglone in black walnut. *Hortic Res* 2018;**5**:67. doi: 10.1038/s41438-018-0067-5.
16. Cheng HY, Concepcion G, Feng XW, Zhang HW and Li H. Haplotype-resolved de novo assembly using phased assembly graphs with hifiasm. *Nat Methods* 2021;**18**(2):1-6. doi: 10.1038/s41592-020-01056-5.
17. Burton JN, Adey A, Patwardhan RP, Qiu R, Kitzman JO and Shendure J. Chromosome-scale scaffolding of de novo genome assemblies based on chromatin interactions. *Nat Biotechnol* 2013;**31**(12):1119-25. doi: 10.1038/nbt.2727.
18. Mosè M, Berkeley MR, Mathieu S, Simo FA and Zdobnov EM. BUSCO update: novel and streamlined workflows along with broader and deeper phylogenetic coverage for scoring of Eukaryotic, Prokaryotic, and Viral genomes. *Mol Biol Evol* 2021;**38**(10):4647-4654. doi: 10.1093/molbev/msab199.
19. Campbell MS, Holt C, Moore B and Yandell M. Genome annotation and curation using MAKER and MAKER-P. *Curr Protoc Bioinf* 2014;**48**:4.11.1-4..39. doi: 10.1002/0471250953.bi0411s48.
20. Yang FS, Nie S, Liu H, Shi TL and Mao JF. Chromosome-level genome assembly of a parent species of widely cultivated azaleas. *Nat Commun* 2020;**11**:5269. doi: 10.1038/s41467-020-18771-4.
21. Fu AZ, Wang Q, Mu JL, Ma LL and Wen CL, et al. Combined genomic, transcriptomic, and metabolomic analyses provide insights into chayote (Sechium edule) evolution and fruit development. *Hortic Res* 2021;**8**:35. doi: 10.1038/s41438-021-00487-1.
22. Zhu TT, Wang L, You FM, Rodriguez JC, Deal KR, Chen L, et al. Sequencing a *Juglans regia*×*J. microcarpa* hybrid yields high-quality genome assemblies of parental species. *Hortic Res* 2019;**6**:55. doi: 10.1038/s41438-019-0139-1.
23. Marrano A, Britton M, Zaini PA, Zimin AV and Neale DB. High-quality chromosome-scale assembly of the walnut (*Juglans regia* L.) reference genome. *Gigascience* 2019;**9**(5):giaa050. doi: 10.1093/gigascience/giaa050.
24. Ma Q, Sun T, Li S, Wen J, Zhu L, Yin T, et al. The *Acer truncatum* genome provides insights into the nervonic acid biosynthesis. *The Plant Journal* 2020;**104**(3):662-78. doi: 10.1111/tpj.14954.
25. Li Y, Wei H, Yang J, Kang D and Kang X. High-quality *de novo* assembly of the *Eucommia ulmoides* haploid genome provides new insights into evolution and rubber biosynthesis. *Hortic Res* 2020;**7**(1):183. doi: 10.1038/s41438-020-00406-w.

26. Tu L, Su P, Zhang Z, Gao L and Gao W. Genome of *Tripterygium wilfordii* and identification of cytochrome P450 involved in triptolide biosynthesis. *Nat Commun* 2020;**11**(1):971. doi: 10.1038/s41467-020-14776-1.

27. Chen S, Wang Y, Yu L, Zheng T, Wang S, Yue Z, et al. Genome sequence and evolution of *Betula platyphylla*. *Hortic Res* 2021;**8**(1):37. doi: 10.1038/s41438-021-00481-7.

28. Li L, Stoeckert CJ and Roos DS. OrthoMCL: Identification of Ortholog Groups for Eukaryotic Genomes. *Genome Res* 2003;**13**(9):2178-89. doi: 10.1101/gr.1224503.

29. Alexandros S. RAxML version 8: a tool for phylogenetic analysis and post-analysis of large phylogenies. *Bioinformatics* 2014;**30**(9):1312-1313. doi: 10.1093/bioinformatics/btu033.

30. Wang P, Luo Y, Huang J, Gao S, Zhu G, Dang Z, et al. The genome evolution and domestication of tropical fruit mango. *Genome Biol* 2020;**21**:60. doi: 10.1186/s13059-020-01959-8.

31. Manchester SR and Garden MB. Fossil History of the Juglandaceae. *Ann Mo Bot Gard* 1987;**21**:1-137.

32. Manchester SR. Early history of theJuglandaceae. *Plant Syst Evol* 1989;**162**(1):231-50. doi: 10.1007/BF00936919.

33. Zhang JB, Li RQ, Xiang XG, Manchester SR, Li L, Wang W, et al. Integrated fossil and molecular data reveal the biogeographic diversification of the Eastern Asian-Eastern North American disjunct Hickory genus (Carya Nutt.). *Plos One* 2013;**8**(7):e70449. doi: 10.1371/journal.pone.0070449.

34. Yoon Y, Seo DH, Shin H, Kim HJ, Kim CM and Jang G. The Role of Stress-Responsive Transcription Factors in Modulating Abiotic Stress Tolerance in Plants. agronomy 2020;**10**(6):788.

35. Li JN and Gao RX. Research progress on the genetic breeding of *Juglans mandshurica* in China. *J Anhui Agric Sci* 2020;**48**(17):4-7. doi: 10.3969/j.issn.0517-6611.2020.17.002.

36. Zhao Z. Analysis and evaluation on fatty acid composition of Hickory nut oil. *J Anhui Agric Sci* 2009;**37**(06):2473-4. doi: 10.13989/j.cnki.0517-6611.2009.06.113.

37. Ding M, Lou H, Chen W, Zhou Y, Zhang Z, Xiao M, et al. Comparative transcriptome analysis of the genes involved in lipid biosynthesis pathway and regulation of oil body formation in *Torreya grandis* kernels. *Ind Crops Prod* 2020;**145**:112051. doi: 10.1016/j.indcrop.2019.112051.

38. Zhang L, Liu M, Long H, Dong W and Tan X. Tung tree (*Vernicia fordii*) genome provides a resource for understanding genome evolution and improved oil production. *Genomics Proteomics Bioinf* 2020;**17**(6):558-75. doi: 10.1016/j.gpb.2019.03.006.

39. Rao G, Zhang J, Liu X, Lin C and Wang C. *De novo* assembly of a new *Olea europaea* genome accession using nanopore sequencing. *Hortic Res* 2021;**8**:64. doi: 10.1038/s41438-021-00498-y.

40. Huang R, Zhou Y, Zhang J, Ji F and Pei D. Transcriptome analysis of Walnut (*Juglans regia* L.) embryos reveals key developmental stages and genes involved in lipid biosynthesis and polyunsaturated fatty acid metabolism. *J Agric Food Chem* 2020;**69**(1): 377–396. doi: 10.1021/acs.jafc.0c05598.

41. Wang X, Liang H, Guo D, Guo L, Duan X, Jia Q, et al. Integrated analysis of transcriptomic and proteomic data from tree peony (P. ostii) seeds reveals key developmental stages and candidate genes related to oil biosynthesis and fatty acid metabolism. *Hortic Res* 2019;**6**:111. doi: 10.1038/s41438-019-0194-7.

42. Li N, Meng H, Li S, Zhang Z and Luo K. Two novel plastid fatty acid exporters contribute to seed oil accumulation in Arabidopsis. *Plant Physiol* 2020;**182**(4):1910-9. doi: 10.1104/pp.19.01344.

43. Gong W, Song Q, Ji K, Gong S, Wang L, Chen L, et al. Full-length transcriptome from *Camellia oleifera* seed provides insight into the transcript variants involved in oil biosynthesis. *J Agric Food*

Chem 2020;**68**(49):14670–83. doi: 10.1021/acs.jafc.0c05381.

44. Elhai N, Duncan RW and Stasolla C. Molecular regulation of seed oil accumulation. *Journal of Advanced Nutrition and Human Metabolism* 2016;**2**:e1296. doi: 10.14800/janhm.1296.
45. Wang J, K SS, Du C, Li C, Fan J, Sitakanta P, et al. Comparative transcriptomic analysis of two *Brassica napus* near-isogenic lines reveals a network of genes that influences seed oil accumulation. Front Plant Sci 2016;**7**:1498. doi: 10.3389/fpls.2016.01498.
46. Neale DB, Martínez-García P, De L, Montanari S and Wei XX. Novel insights into tree biology and genome evolution as revealed through genomics. *Annu Rev Plant Biol* 2017;**68**(1):13.1-.27. doi: 10.1146/annurev-arplant-042916-041049.
47. Chen H, Zeng Y, Yang Y, Huang L and Qiu Q. Allele-aware chromosome-level genome assembly and efficient transgene-free genome editing for the autotetraploid cultivated alfalfa. *Nat Commun* 2020;**11**(1):2494. doi: 10.1038/s41467-020-16338-x.
48. Edger PP, Poorten TJ, Vanburen R, Hardigan MA, Colle M, Mckain MR, et al. Origin and evolution of the octoploid strawberry genome. *Nat Genet* 2019;**51**(3):541-547. doi: 10.1038/s41588-019-0356-4.
49. Bai WN, Yan PC, ZHang BW, Woeste KE, Lin K and Zhang DY. Demographically idiosyncratic responses to climate change and rapid Pleistocene diversification of the walnut genus Juglans (Juglandaceae) revealed by whole-genome sequences. *New Phytol* 2018;**217**(4):1726-36. doi: 10.1111/nph.14917.
50. Yan F, Xi RM, She RX, Chen PP, Yan YJ, Yang G, et al. Improved *de novo* chromosome- level genome assembly of the vulnerable walnut tree *Juglans mandshurica* reveals gene family evolution and possible genome basis of resistance to lesion nematode. *Mol Ecol Resour* 2021;**21**:2063-2076. doi: 10.1111/1755-0998.13394.
51. Guo W, Chen J, Li J, Huang J and Lim KJ. Portal of Juglandaceae: A comprehensive platform for Juglandaceae study. *Hortic Res* 2020;**7**:35. doi: 10.1038/s41438-020-0256-x.
52. Bai WN, Wang WT and Zhang DY. Phylogeographic breaks within Asian butternuts indicate the existence of a phytogeographic divide in East Asia. *New Phytologist* 2015;**209**(4):1757-1772. doi: 10.1111/nph.13711.
53. Tang H, Bowers JE, Wang X, Ming R, Alam M and Paterson AH. Synteny and collinearity in plant genomes. *Science* 2008;**320**(5875):486-488. doi: 10.1126/science.1153917.
54. Frith MC and Kawaguchi R. Split-alignment of genomes finds orthologies more accurately. *Genome biology* 2015;**16**(1):106. doi: 10.1186/s13059-015-0670-9.
55. Zhang J, Zhang W, Ji F, Qiu J, Song X, Bu D, et al. A high-quality walnut genome assembly reveals extensive gene expression divergences after whole-genome duplication. *Plant Biotechnol J* 2020;**18**:1848-1850. doi: 10.1111/pbi.13350.
56. Martínez- García PJ, Crepeau MW, Puiu D, Gonzalez- Ibeas D, Whalen J, Stevens KA, et al. The walnut (*Juglans regia*) genome sequence reveals diversity in genes coding for the biosynthesis of non- structural polyphenols. *The Plant Journal* 2016;**87**(5):507-532. doi: 10.1111/tpj.13207.
57. Luo MC, You FM, Li P, Wang JR, Zhu T, Dandekar AM, et al. Synteny analysis in Rosids with a walnut physical map reveals slow genome evolution in long-lived woody perennials. *BMC Genomics* 2015;**16**(1):627-44. doi: 10.1186/s12864-015-1906-5.
58. Farag MA. Headspace analysis of volatile Compounds in leaves from the Juglandaceae (Walnut) family. *J Essent Oil Res* 2008;**20**(4):323-327. doi: 10.1080/10412905.2008.9700023.
59. Delaviz H, Mohammadi J, Ghalamfarsa G, Mohammadi B and Farhadi N. A review study on

phytochemistry and pharmacology applications of Juglans regia plant[J]. Pharmacognosy reviews, 2017, **11**(22):145. doi: 10.4103/phrev.phrev_10_17.

60. Li YG, Mou FJ and Li KZ. *De novo* RNA sequencing and analysis reveal the putative genes involved in diterpenoid biosynthesis in *Aconitum vilmorinianum* roots. *3 Biotech* 2021;**11**(2):1-12. doi: 10.1007/s13205-021-02646-6.

61. Ma Y, Cui G, Chen T, Ma X and Huang L. Expansion within the CYP71D subfamily drives the heterocyclization of tanshinones synthesis in *Salvia miltiorrhiza*. *Nat Commun* 2021;**12**(1):685. doi: 10.1038/s41467-021-20959-1.

62. Zhao Q, Weng JK, Chen XY, Martin C, Cui MY, Levsh O, et al. Two CYP82D enzymes function as flavone hydroxylases in the biosynthesis of root-specific 4'-Deoxyflavones in *Scutellaria baicalensis*. *Mol Plant* 2018;**11**(1):135-148. doi: 10.1016/j.molp.2017.08.009.

63. Cheng Y, Liu H, Tong X, Liu Z, Zhang X, Li D, et al. Identification and analysis of CYP450 and UGT supergene family members from the transcriptome of *Aralia elata* (Miq.) seem reveal candidate genes for triterpenoid saponin biosynthesis. *BMC Plant Biol* 2020;**20**(1):214. doi: 10.1186/s12870-020-02411-6.

64. Chi WC, Fu SF, Huang TL, Chen YA, Chen CC and Huang HJ. Identification of transcriptome profiles and signaling pathways for the allelochemical juglone in rice roots. *Plant Mol Biol* 2011;**77**:591-607. doi: 10.1007/s11103-011-9841-6.

65. Zhao LF, Katavic V, Li FL, Haughn GW and Ljerka K. Insertional mutant analysis reveals that long-chain acyl-CoA synthetase 1 (LACS1), but not LACS8, functionally overlaps with LACS9 in Arabidopsis seed oil biosynthesis. *The Plant Journal* 2010;**64**(6):1048-58. doi: 10.1111/j.1365-313X.2010.04396.x.

66. Aznar-Moreno JA, Venegas Calerón M, Martínez-Force E, Garcés R, Mullen R, Gidda SK, et al. Sunflower (*Helianthus annuus*) long-chain acyl-coenzyme A synthetases expressed at high levels in developing seeds. *Physiol Plant* 2014;150(3):363-373. doi: 10.1111/ppl.12107.

67. Tai S, Chen M, Peng CC and Tzen J. Gene family of Oleosin isoforms and their structural stabilization in Sesame seed oil bodies. *Journal of the Agricultural Chemical Society of Japan* 2002;**66**(10):2146-53. doi: 10.1271/bbb.66.2146.

68. Jiang PL, Wang CS, Hsu CM, Jauh GY, Tzen and C. JT. Stable oil bodies sheltered by a unique Oleosin in Lily pollen. *Plant Cell Physiol* 2007;**48**(6):812-21. doi: 10.1093/pcp/pcm051.

69. Tzen J, Cao Y, Laurent P, Ratnayake C and Huang A. Lipids, proteins, and structure of seed oil bodies from diverse species. *Plant Physiol* 1993;**101**(1):267-76. doi: 10.1104/pp.101.1.267.

70. Xing GL, Li JY, Li WL, Lam SM, Yuan HL, Shui GH, et al. AP2/ERF and R2R3-MYB family transcription factors: potential associations between temperature stress and lipid metabolism in Auxenochlorella protothecoides. *Biotechnol Biofuels* 2021;**14**(1):22. doi: 10.1186/s13068-021-01881-6.

71. Maeo K, Tokuda T, Ayame A, Mitsui N and Nakamura K. An AP2-type transcription factor, WRINKLED1, of Arabidopsis thaliana binds to the AW-box sequence conserved among proximal upstream regions of genes involved in fatty acid synthesis. *The Plant Journal* 2010;**60**(3):476-487. doi: 10.1111/j.1365-313X.2009.03967.x.

72. Kumar N, Chaudhary A, Singh D and Teotia S. Transcriptional regulation of seed oil accumulation in Arabidopsis thaliana : role of transcription factors and chromatin remodelers. *J Plant Biochem Biotechnol* 2020;**29**:754-768. doi: 10.1007/s13562-020-00616-2.

73. Chen DJ, Luo XG, Yan LH, Si CL and Zhang TC. Transcriptome analysis of unsaturated fatty acids

biosynthesis shows essential genes in sprouting of Acer truncatum Bunge seeds. *Food Biosci* 2020;**41**:100739. doi: 10.1016/j.fbio.2020.100739.

74. Ding MZ, Lou HQ, Chen WC, Zhou Y, Zhang ZH, Xiao MH, et al. Comparative transcriptome analysis of the genes involved in lipid biosynthesis pathway and regulation of oil body formation in Torreya grandis kernels. *Ind Crops Prod* 2020;**145**:112051. doi: 10.1016/j.indcrop.2019.112051.
75. Scbbastien B, Sylvie MW, Alexandra T, Christine R and Locc L. Role of WRINKLED1 in the transcriptional regulation of glycolytic and fatty acid biosynthetic genes in Arabidopsis. *The Plant Journal* 2009;**60**(6):933-47. doi: 10.1111/j.1365-313X.2009.04011.x.
76. Meng Z, Xia C, Jia Q and Ohlrogge J. FUSCA3 activates triacylglycerol accumulation in Arabidopsis seedlings and tobacco BY2 cells. *The Plant Journal* 2016;**88**(1):95-107. doi: 10.1111/tpj.13233.
77. Bo S, Allen WB, Zheng P, Li C and Glassman K. Expression of ZmLEC1 and ZmWRI1 Increases Seed Oil Production in Maize. *Plant Physiol* 2010;**153**:980-987. doi: 10.1104/pp.110.157537.
78. Porebski S, Bailey LG and Baum BR. Modification of a CTAB DNA extraction protocol for plants containing high polysaccharide and polyphenol components. *Plant Mol Biol Rep* 1997;**15**(1):8-15. doi: 10.1007/BF02772108.
79. Liu B, Shi Y, Yuan J, Hu X, Zhang H, Li N, et al. Estimation of genomic characteristics by analyzing k-mer frequency in de novo genome projects. *Quant Biol* 2013;**35**:62-67. doi: 10.48550/arXiv.1308.2012.
80. Li H, Feng XW and Chu C. The design and construction of reference pangenome graphs with minigraph. *Genome Biology* 2020;**21**:265. doi: 10.1186/s13059-020-02168-z.
81. Yang X, Liu D, Liu F, Wu J, Zou J and Xiao X. HTQC: A fast quality control toolkit for Illumina sequencing data. *BMC Bioinf* 2013;**14**(1):33. doi: 10.1186/1471-2105-14-33.
82. Li H. Aligning sequence reads, clone sequences and assembly contigs with BWA-MEM. *Genomics* 2013;**1303**:3097. doi:10.48550/arXiv.1303.3997 .
83. Li H. Minimap2: fast pairwise alignment for long nucleotide sequences. *Bioinformatics* 2018;**34**(18):3094-100. doi: 10.1093/bioinformatics/bty191.
84. Waterhouse RM, Fredrik T, Li J, Zdobnov EM and Kriventseva EV. OrthoDB: a hierarchical catalog of animal, fungal and bacterial orthologs. *Nucleic Acids Res* 2013;**41**:D358-D365. doi: 10.1093/nar/gks1116.
85. Simão F, Waterhouse RM, Panagiotis I, Kriventseva EV and Zdobnov EM. BUSCO: assessing genome assembly and annotation completeness with single-copy orthologs. *Bioinformatics* 2015;**31**(19):3210-3212. doi: 10.1093/nar/gks1116.
86. Chen N. Using RepeatMasker to identify repetitive elements in genomic sequences. *Curr Protoc Bioinf* 2004;**25**:4-10. doi: 10.1002/0471250953.bi0410s05.
87. Jurka J, Kapitonov VV, Pavlicek A, Klonowski P, Kohany O and Walichiewicz J. Repbase Update, a database of eukaryotic repetitive elements. *Cytogenet Genome Res* 2005;**110**(1-4):462-467. doi: 10.1159/000084979.
88. Abrusán G, Grundmann N, DeMester L and Makalowski W. TEclass--a tool for automated classification of unknown eukaryotic transposable elements. *Bioinf* 2009;**25**(10):1329-30. doi: 10.1093/bioinformatics/btp084.
89. Xu Z and Wang H. LTR_FINDER: an efficient tool for the prediction of full-length LTR retrotransposons. *Nucleic Acids Res* 2007;**35**:W265-W268. doi: 10.1093/nar/gkm286.
90. Benson G. Tandem repeats finder: a program to analyze DNA sequences. *Nucleic Acids Res*

1999;**27**(2):573-80. doi: 10.1093/nar/27.2.573.

91. Gertz, EM, Yu YK, Agarwala R, Schäffer AA and Altschul SF. Composition-based statistics and translated nucleotide searches: Improving the TBLASTN module of BLAST. BMC Biol 2006;**4**:41. doi: 10.1186/1741-7007-4-41.
92. Slater G and Birney E. Automated generation of heuristics for biological sequence comparison. BMC Bioinf 2005;**6**:31. doi: 10.1186/1471-2105-6-31.
93. Stanke M, Keller O, Gunduz I, Hayes A, Waack S and Morgenstern B. AUGUSTUS: ab initio prediction of alternative transcripts. *Nucleic Acids Res* 2006;34:W435-W9. doi: 10.1093/nar/gkl200.
94. Majoros W, Pertea M and Salzberg S. TigrScan and GlimmerHMM: two open source ab initio eukaryotic gene-finders. *Bioinformatics* 2004;**20**(16):2878-2879. doi: 10.1093/bioinformatics/bth315.
95. Wu T and Watanabe C K. GMAP: a genomic mapping and alignment program for mRNA and EST sequences. *Bioinformatics* 2005;**21**(9):1859-1875. doi: 10.1093/bioinformatics/bti310.
96 Haas BJ, Papanicolaou A, Yassour M, Grabherr M, Blood PD, Bowden J, et al. *De novo* transcript sequence reconstruction from RNA-seq using the Trinity platform for reference generation and analysis. *Nat Protoc.* 2013;**8**:1494-1512. doi: 10.1038/nprot.2013.084.
97. Camacho C, Coulouris G, Avagyan V, Ning M, Papadopoulos J, Bealer K, et al. BLAST+: architecture and applications. *BMC Bioinf* 2009;**10**(1):421. doi: 10.1186/1471-2105-10-421.
98. Lowe TM and Eddy SR. tRNAscan-SE: a program for improved detection of transfer RNA genes in genomic sequence. *Nucleic Acids Res* 1997;**25**(5):955-964. doi: 10.1093/nar/25.5.0955.
99. Nawrocki E, Kolbe DL and Eddy SR. Infernal 1.0: inference of RNA alignments. *Bioinformatics* 2009;**25**(10):1335-7. doi: 10.1093/bioinformatics/btp157.
100. Stevens KA, Woeste K, Chakraborty S, Crepeau MW and Langley CH. Genomic Variation Among and Within Six *Juglans* Species. *G3: Genes, Genomes, Genet* 2018;**8**(7):2153-5165. doi: 10.1534/g3.118.200030.
101. Huang Y, Xiao L, Zhang Z, Zhang R, Wang Z, Huang C, et al. The genomes of pecan and Chinese hickory provide insights into *Carya* evolution and nut nutrition. *GigaScience* 2019;**8**(5):giz036. doi: 10.1093/gigascience/giz036.
102. Sork VL, Fitz-Gibbon ST, Puiu D, Crepeau M and Salzberg SL. First draft assembly and annotation of the genome of a california endemic Oak *Quercus lobata* Née (Fagaceae). *G3: Genes, Genomes, Genet* 2016;**6**(11):3485-3495. doi: 10.1534/g3.116.030411.
103. Jansen RK, Saski C, Lee SB, Hansen A and Daniell H. Complete plastid genome sequences of three Rosids (Castanea, Prunus, Theobroma): evidence for at least two independent transfersn of rpl22 to the nucleus. *Mol Biol Evol* 2011;**28**(1):835-847. doi: 10.1093/molbev/msq261.
104. Jia HM, Jia HJ, Cai QL, Wang Y, Zhao HB, Yang WF, et al. The red bayberry genome and genetic basis of sex determination. *Plant Biotechnol J* 2019;**17**(2):397-409. doi: 10.1111/pbi.12985.
105. Tuskan G, Difazio S, Jansson S, Bohlmann J, Grigoriev I, Hellsten U, et al. The genome of Black Cottonwood, *Populus trichocarpa* (Torr. & Gray). *Science* 2006;**313**(5793):1596-1604. doi: 10.1126/science.1128691.
106. Jaillon O, Aury JM, Noel B, Policriti A, Clepet C, Casagrande A, et al. The grapevine genome sequence suggests ancestral hexaploidization in major angiosperm phyla. *Nature* 2007;**449**(7161):463-467. doi: 10.1038/nature06148.
107. Kawahara Y, Bastide M, Hamilton JP, Kanamori H, McCombie WR, Ouyang S, et al. Improvement of the *Oryza sativa* Nipponbare reference genome using next generation sequence and optical map

data. *Rice* 2013;**6**:4. doi: 10.1186/1939-8433-6-4.

108. Edgar RC. MUSCLE: multiple sequence alignment with high accuracy and high throughput. *Nucleic Acids Res* 2004;**32**:1792-1797. doi: 10.1093/nar/gkh340.
109. Sanderson MJ. r8s: Inferring absolute rates of molecular evolution and divergence times in the absence of a molecular clock. *Bioinformatics* 2003;**19**(2):301-302. doi: 10.1093/bioinformatics/19.2.301.
110. Yang Z. PAML 4: Phylogenetic Analysis by Maximum Likelihood. *Mol Biol Evol* 2007;**24**(8):1586-1591. doi: 10.1093/molbev/msm088.

111 De Bie T, Cristianini N, Demuth JP, et al. CAFE: A computational tool for the study of gene family evolution. *Bioinformatics* 2006;**22**(10):1269-1271. doi: 10.1093/bioinformatics/btl097.

112. Chen C, Chen H, Zhang Y, Thomas HR and Xia R. TBtools: An integrative toolkit developed for interactive analyses of big biological data. *Molecular Plant* 2020;**13**(8):1194-1202. doi: 10.1101/289660.
113. Delcher AL, Salzberg SL and Phillippy AM. Using MUMmer to identify similar regions in large sequence sets. *Curr Protoc Bioinf* 2003;**Chapter10**(1):Unit10.3. doi: 10.1002/0471250953.bi1003s00.
114. Kim D, Paggi JM, Park C, Bennett C and Salzberg SL. Graph-based genome alignment and genotyping with HISAT2 and HISAT-genotype. *Nat Biotechnol* 2019;**37**:907-915. doi: 10.1038/s41587-019-0201-4.
115. Liao Y, Smyth GK and Shi W. featureCounts: An efficient general-purpose program for assigning sequence reads to genomic features. *Bioinformatics* 2013;**30**:923-930. doi: 10.1093/bioinformatics/btt656.
116. Li B and Dewey CN. RSEM: accurate transcript quantification from RNA-Seq data with or without a reference genome. *BMC Bioinform* 2011;**12**:323. doi: 10.1186/1471-2105-12-323.
117. Love MI, Huber W and Anders S. Moderated estimation of fold change and dispersion for RNA-seq data with DESeq2. *Genome Biol* 2014;**15**(12):550. doi: 10.1186/PREACCEPT-8897612761307401.
118. Zheng Y, Jiao C, Sun H, G.Rosli H, A.Pombo M, Zhang P, et al. iTAK: a program for genome-wide prediction and classification of plant transcription factors, transcriptional regulators, and protein kinases. *Molecular Plant* 2016;**9**:1667-1670. doi: 10.1016/j.molp.2016.09.014.
119. Tian F, Yang DC, Meng YQ, Jin J and Gao G. PlantRegMap: charting functional regulatory maps in plants. *Nucleic Acids Res* 2020;**48**:D1104-D1113. doi: 10.1093/nar/gkz1020.
120. Ihaka R and Gentleman R. R: a language for data analysis and graphics. *Journal of computational and graphical statistics*, 1996;**5**(3): 299-314. doi: 10.2307/1390807.
121. Csardi G and Nepusz T. The igraph software package for complex network research. *Interjournal Complex Systems*, 2006; **1695**(5):1-9.
122. Kumar S, Stecher G and Tamura K. MEGA7: Molecular evolutionary genetics analysis version 7.0 for bigger datasets. *Mol Biol Evol* 2015;**33**:1870-1874. doi: 10.1093/molbev/msw054.
123. Metabolomics data at Metabolights repository. www.ebi.ac.uk/metabolights/MTBLS3657.
124. Li X; Cai K; Zhang Q; Pei X; Chen S; Jiang L; et al. Supporting data for "The Manchurian Walnut Genome: Insights into Juglone and Lipid Biosynthesis". *GigaScience Database*. 2022. http://dx.doi.org/10.5524/102223.

Figure legends

Figure 1. Photographs of *J. mandshurica*. (a) male flower (b) female flower (c) mature fruit (d) adult tree.

Figure 2 Genome information and Hi-C interaction heatmap of *J. mandshurica*. **a** distribution of *J. mandshurica* genomic features. (A) Circular representation of the Chromosome. (B) gene density. (C) repeat sequence density. (D) rRNA density. (E) tRNA density. (F) GC content density. **b** Intensity signal heatmap of the Hi-C chromosome. The color in the figure from light to dark indicates the increase in the intensity of interaction.

Figure 3 Phylogenetic analyses of the *J. mandshurica* genome. (**a**) Venn diagram showing the shared and unique gene families among *J. mandshurica* and four other species (*J. cathayensis, J. regia, J. macrocarpa* and *O. sativa*). (**b)** an overview of orthologous and paralogous genes among *J. mandshurica* and related species. 'Single-copy orthologs' include common orthologs with one copy in specific species. 'Unique paralogs' include genes that do not have any similarity to genes in the other species based on BLAST and OrthoMCL. 'Multi-copy orthologs' include common orthologs with multiple copy numbers in specific species. 'Unclustered' include genes that cannot be clustered into known gene families. 'Other orthologs' include genes from families shared in 2–15 species. (**c)** phylogenetic tree of 15 species including *J. mandshurica, J. cathayensis, J. macrocarpa, J. nigra, J. regia, J. hindsii, J. sigillata, C. cathayensis, C. illinoinensis, Q. lobata, C. mollissima, M. rubra, P. trichocarpa, V. vinifera* and *O. sativa* based on orthologs of single-gene families. Blue numbers at each node represent the estimated time of each divergent event. Green and orange numbers along each branch indicate the number of expanded and contracted gene families, respectively. Pie charts show the proportions of gene families that underwent expansion or contraction.

Figure 4 Collinearity and WGD analysis of *Juglans mandshurica* genome. **(a)** Ks distribution of synthetic orthologs of the selected species (*J. mandshurica, J. regia* and *J. sigillata*). The x-coordinate is the Ks value, and the y-coordinate represents the number of gene pairs of density. (**b)** 4dTV analysis. The x-coordinate is the 4DTv value, and the y-coordinate represents the number of gene pairs of density. (**c**) schematic representation of syntenic genes among *J. mandshurica, J. regia* and *P. trichocarpa*. Gray lines in the background indicate collinear blocks of at least thirty genes

within the *J. mandshurica* genome and other plants, while the red lines highlight the syntenic gene pairs.

Figure 5 Comparative transcriptomic analysis of genes involved in juglone biosynthesis. **(a)** Juglone biosynthetic pathway. Numbers under the chemical formulae represent the reaction substrate. 1, Chorismite; 2, Isochorismate; 3, SEPHCHC; 4, SHCHC; 5, OSB; 6, OSB-CoA; 7, DHNA-CoA; 8, DHNA; 9, Demethylphylloquinone; 10, Demethylphylloquinol; 11, Phylloquinol; 12, 1,4-NQ. Numbers next to arrows represent characterized enzymes or detected enzymatic activities. 5.4.4.2, isochorismate synthase; 2.2.1.9, 2-succinyl-5-enolpyruvyl-6-hydroxy-3-cyclohexene-2-carboxylate (SEPHCHC) synthase; 4.2.99.20, 2-succinyl-6-hydroxy-2,4-cyclohexadiene-2-carboxylate (SHCHC) synthase; 4.2.1.113, 4.2.1.113 o-succinylbenzoate (OSB) synthase; 6.2.1.26, OSB-CoA ligase; 4.1.3.36, 1,4-dihydroxy-2-naphthoyl-CoA (DHNA-CoA) synthase; 3.1.2.28, DHNA-CoA thioesterase; 2.5.1.74, DHNA phytyl transferase; 1.6.5.9, NAD(P)H dehydrogenase C1 (NDC1); 2.1.1.163, demethylmenaquinone methyltransferase; 4.1.1-, decarboxylase; 2-ODD, 2-ODD: 2-oxoglutarate/Fe(II)-dependent dioxygenase; CYP450, cytochrome CYP450. The red chemical formulae represent Juglone. (**b)** Gray lines in the background indicate collinear blocks of at least thirty genes within the *J. mandshurica* genome and *J. regia*. The red lines highlight the syntenic gene pairs related to a decarboxylase (gene-Jman015G0125100), the green lines highlight syntenic gene pairs related to CYP450s (gene-Jman003G0044800), the blue lines highlight the syntenic gene pairs related to 2-ODD (gene-Jman011G0199700). (**c)** Lineage-specific expansion of the CYP gene family in *J. mandshurica* and three related species. The phylogenetic tree contains four subgroups including Group I- Ⅳ correspond to the subgroups in the phylogenetic tree. (**d)** the heatmap represents the expression level of the expanded CYP gene family in *J. mandshurica*. The color scale from blue to red indicates the expression value from low to high.

Figure 6 Juglone content and TF regulation during walnut exocarp differentiation. (a) The change of *bona fide* juglone content during walnut exocarp differentiation of *J. mandshurica* (mean ± SD, n=3). The x-axis indicates the different developmental stages (from S1 to S4), the x-axis represents the juglone content. **b**, Frequency distribution of the first top 15 transcriptome factors related to juglone (r > 0.8 or < -0.8). (**c)** kinetic patterns of co-expressed genes in cluster 6 (990 genes) during

walnut exocarp differentiation. (**d)** Correlation network of juglone-genes (2 genes and 19 transcription factors). r represents the Pearson correlation coefficient. Line color represents the correlation between genes and Juglone (positive and negative correlations) where a positive correlation is a formal representation of an activator and a negative correlation represents a formal representation of an inhibitor. (**e**) Expression analysis of transcription factors (14 MYBs, 3 FAR1s, 3 NF-YCs, 5 bZIP,s 8 NACs and 4 NF-YA TFs). The heatmap represents normalized fragments per kilobase of transcript per million fragments (FPKM). S1-S4 represents the fruit differentiation stages of *J. mandshurica*.

Figure 7 Comparative transcript analysis of genes involved in lipid biosynthesis of developing fruit. PDH, pyruvate dehydrogenase; ACC (BC), Biotin carboxylase subunit of heteromeric acetyl-CoA carboxylase (ACCase); ACC(BCCP), biotin carboxyl carrier protein of heteromeric ACCase; ACP, acyl carrier protein; KAS, ketoacyl-ACP synthase; SAD, stearoyl-ACP desaturase; FATA, acyl-ACP thioesterase A; FATB, acyl-ACP thioesterase B; LACS, long-chain acyl-CoA sythetase; GPAT, glycerol-3-phosphate acyltransferase; LPAT, 1ysophosphatidic acid acyltransferase; PAP, phosphatidic acid phosphatase; DGAT, diacylglycerol acyltransferase; PDAT, phospholipid: diacylglycerol acyltransferase; TAG, triacylglycerol; CPT, diacylglycerol cholinephosphotransferase; FAD2, v-6 desaturase; FAD3, v-3 desaturase. G-3-P, glycerol-3-P; LPA, 1-Acylglycerol-3P; PA, 1,2-diacylglycerol-3P; DAG, 1,2- diacylglycerol. The color scale from blue to red indicates the expression value from low to high.

Figure 1

Click here to access/download;Figure;Figure 1.pdf

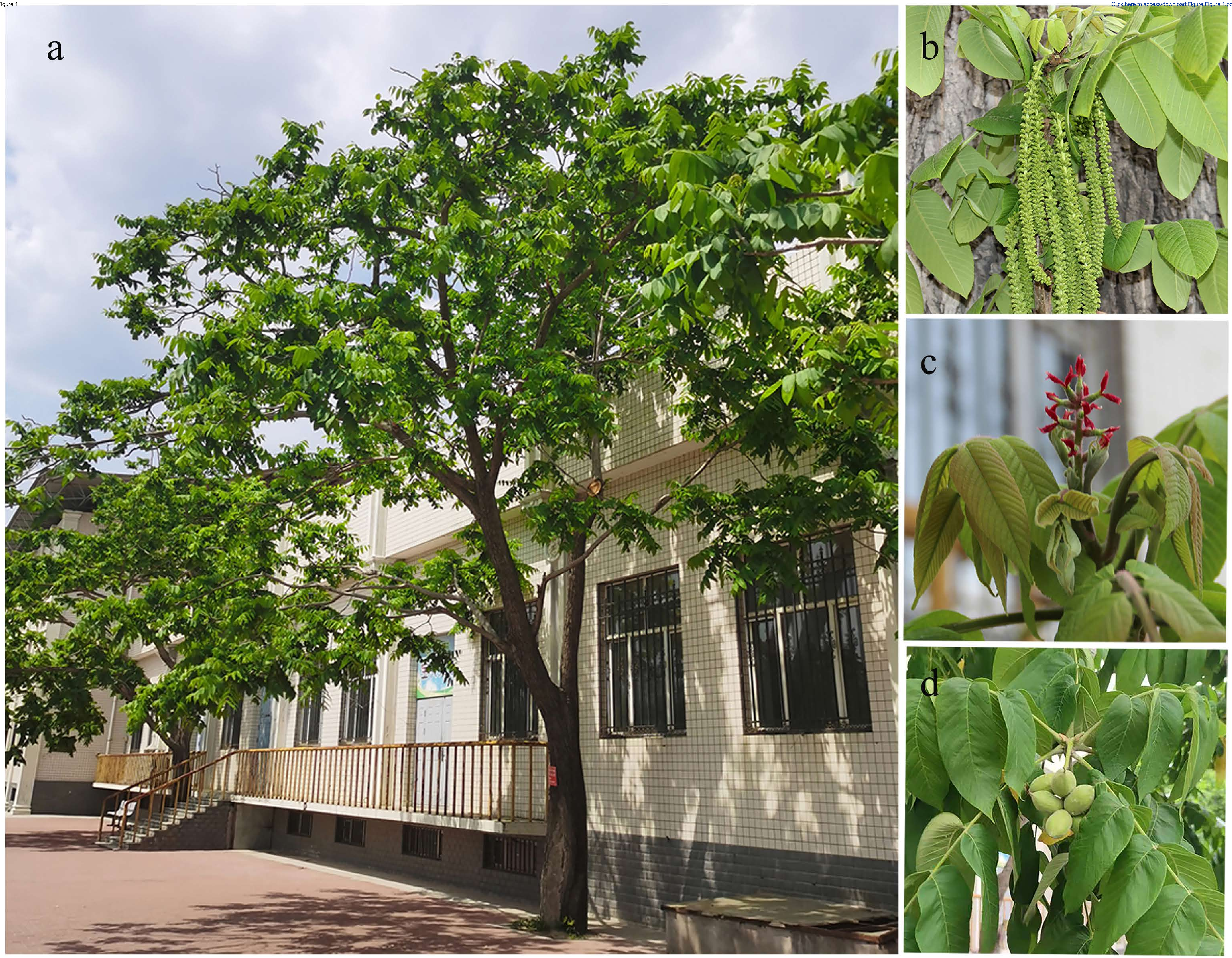

Figure 2

Click here to access/download;Figur

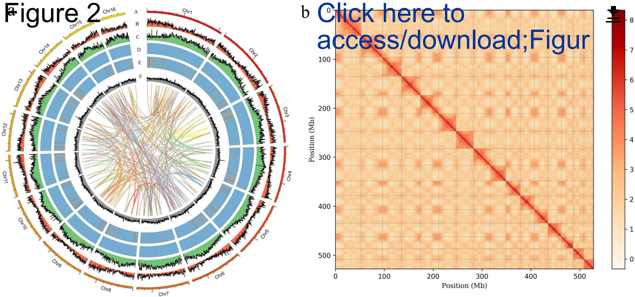

Figure 3

Click here to access/download;Figure;Figure 3.pdf

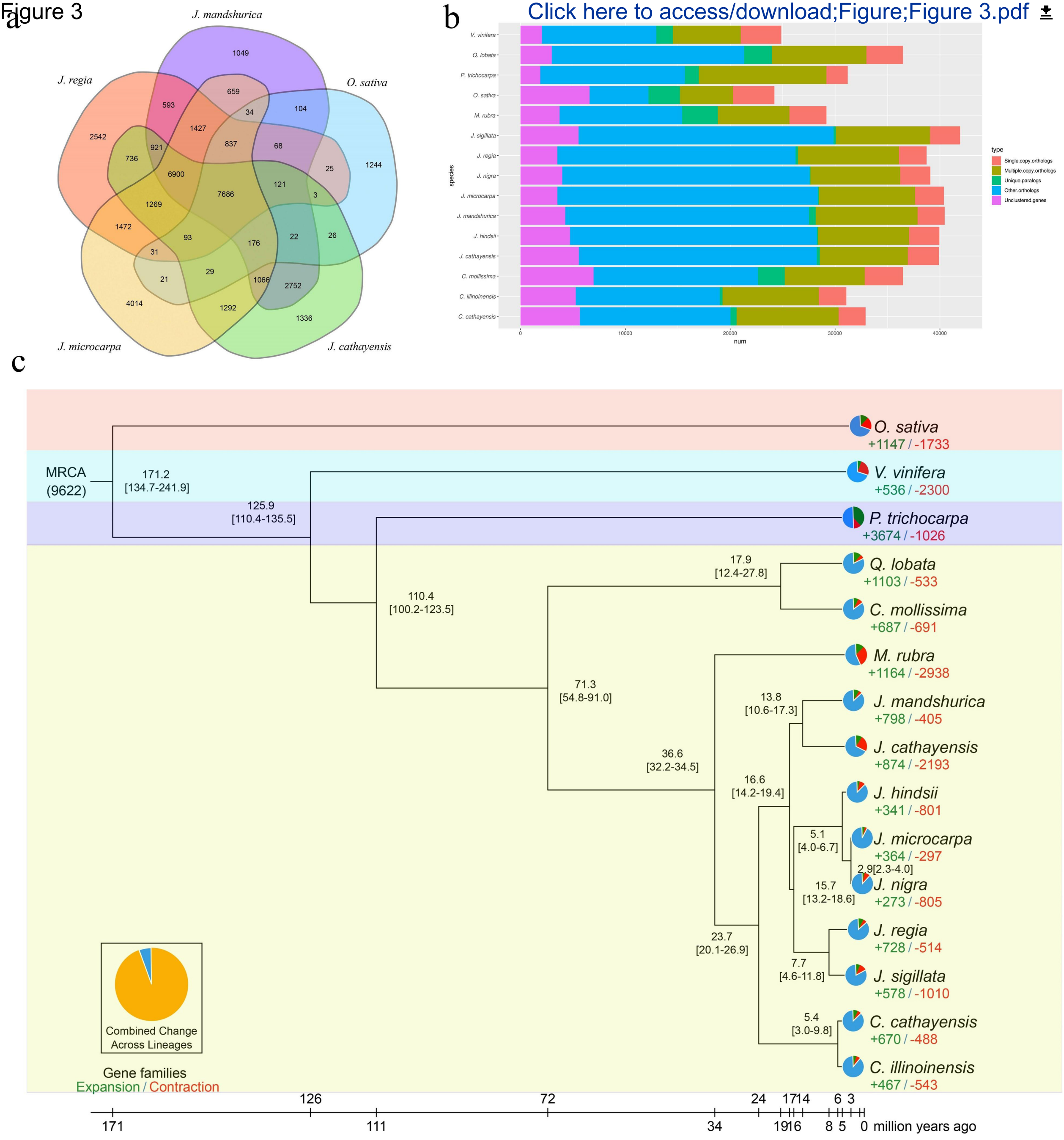

Click here to access/download;Figure;Figure 4.pdf

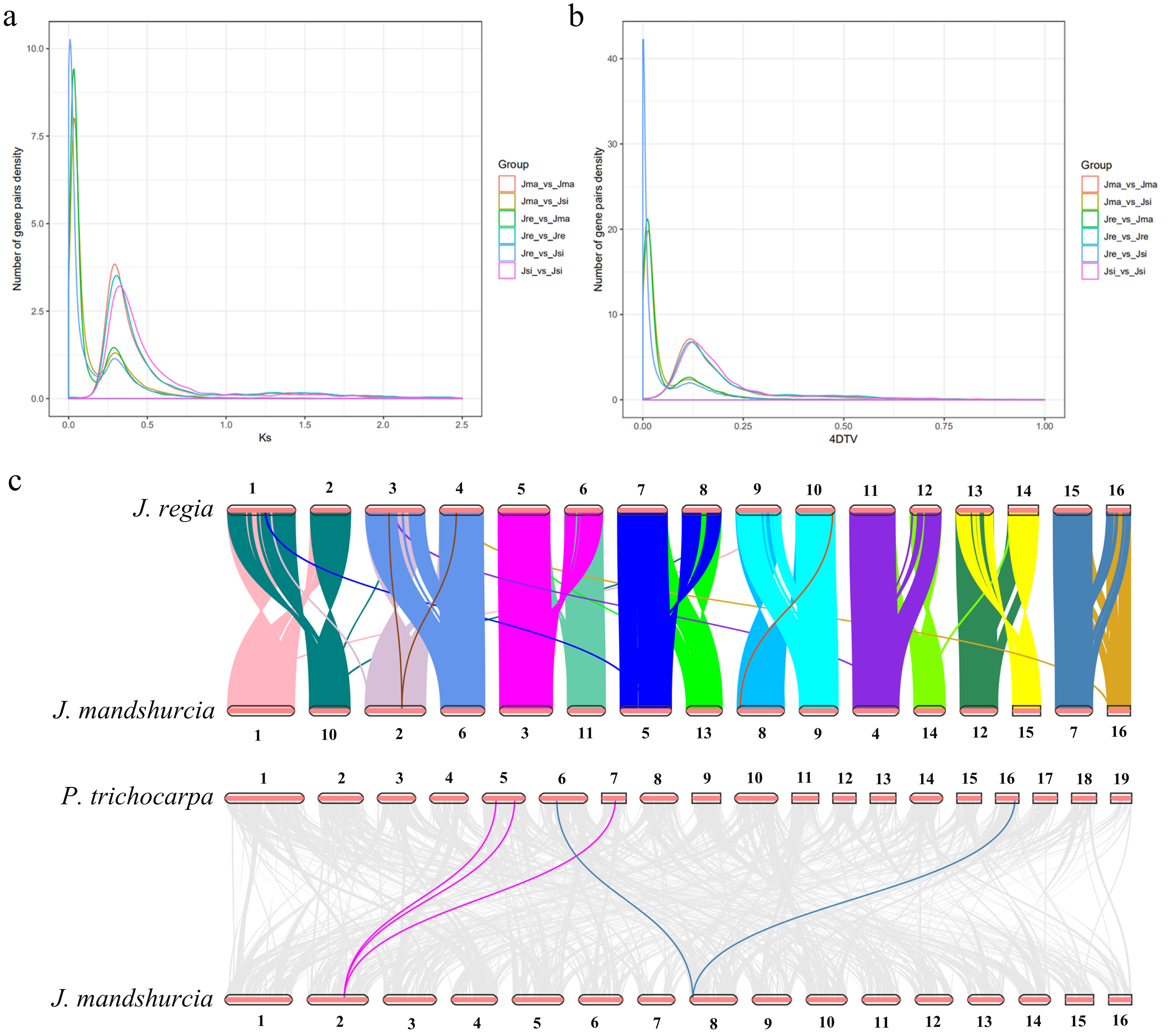

Figure 5

Click here to access/download;Figure;Figure 5.pdf

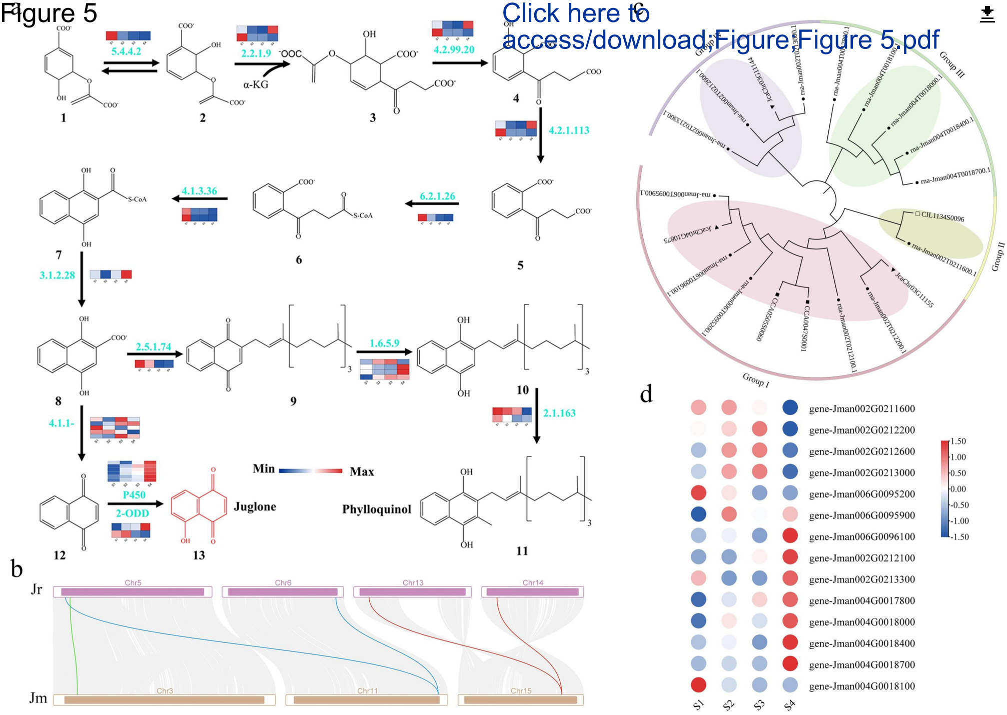

Figure 6

Click here to access/download;Figure;Figure

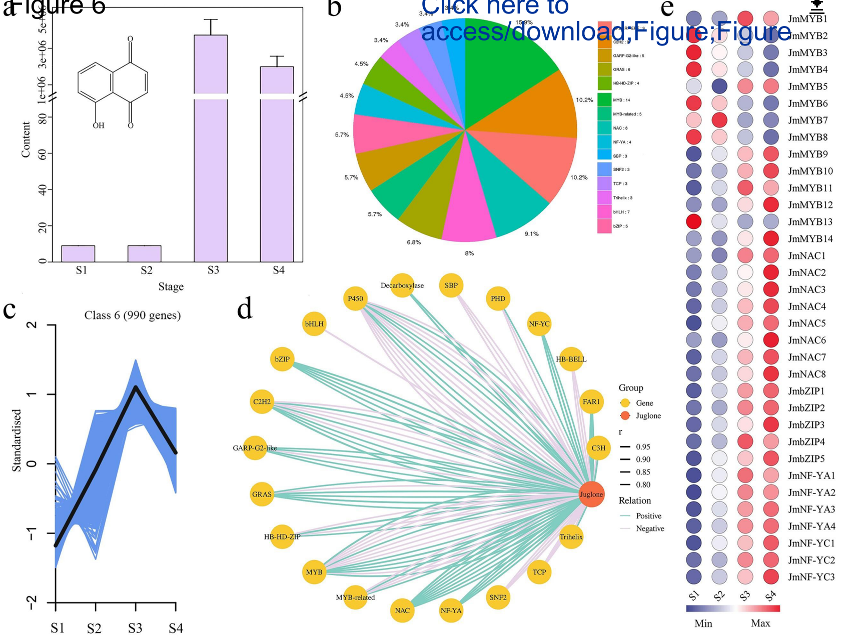

Figure 7

Click here to access/download

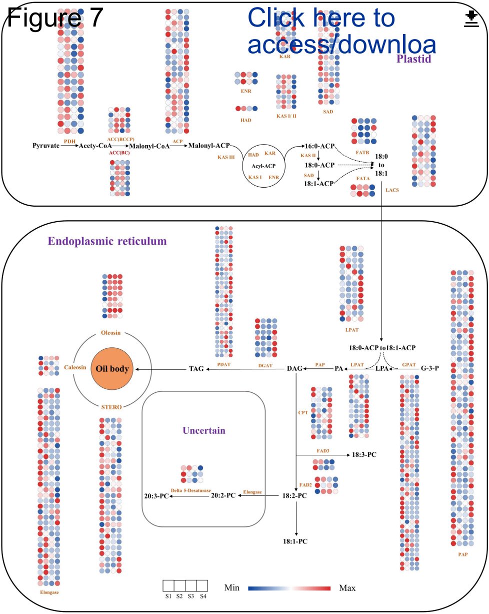

Click here to access/download
Supplementary Material
Supplementary Figures.docx

Supplementary Tables

Click here to access/download
Supplementary Material
Supplementary Tables.xlsx

Cover letter

Click here to access/download;Personal Cover;Cover Letter.doc

Dear Editor,

On behalf of my co-authors, I am submitting the enclosed material "The Manchurian Walnut Genome: Insights into Juglone and Lipid Biosynthesis" for possible publication in **GigaScience**.

According to the comments, we have carefully checked and modified the present manuscript from the following aspects: (1) the detailed methods on metabolomics have been added from line 391-423; (2) the PCA analysis for the metabolights has been added in Figure S10 (walnut exocarp) and Figure S11 (walnut embryos). In the manuscript, we descripted the PCA methods and results from line 426-428 and 435-438. From the principal component analysis of walnut exocarp (Figure S10) and embryos (Figure S11), the obvious distinction of metabolites was found from different sample groups, and these metabolites can be used for subsequent metabolomics analysis; (3) the metabolomics data in this study were deposited and available at Metabolights repository (www.ebi.ac.uk/metabolights/MTBLS3657) under the accession numbers MTBLS3657, see line 681-683; (4) based on odb10, the BUSCO software with latest version (v5.2.2) was employed to evaluate the completeness of the *J. mandshurica* assembly, see line 147. The final BUSCO assessment shows that 98.3% of the complete BUSCO gene set was captured, and the results were added in Table S2; (5) in addition, we also added the photographs of *J. mandshurica* fruits in different development periods and the tissue structure of *J. mandshurica* fruit including the walnut exocarp and walnut embryos, see Figure S1 and Figure S2. All contents modified above has been marked by red color in present manuscript.

All authors certify that they have participated sufficiently in the work to take public responsibility for the appropriateness of the experimental design and method, and the collection, analysis, and interpretation of the data.

The authors have reviewed the final version of the manuscript and approve it for publication. To the best of our knowledge and belief, this manuscript has not been published in whole or in part nor is it being considered for publication elsewhere.

Now the manuscript is submitted online, and I look forward to hearing from you.

Yours Sincerely,

Xiyang Zhao

State Key Laboratory of Tree Genetics and Breeding, School of Forestry, Northeast Forestry University, Harbin, 150040, China.

Tel.: +0086-451-82192225

Fax: +0086-451-82192225

Email: zhaoxyphd@163.com
