# Supplementary material for: The Manchurian Walnut Genome: Insights into Juglone and Lipid Biosynthesis
Source: Gigascience. 2022 Jun 28;11:giac057. doi: 10.1093/gigascience/giac057 (PMC9239856; doi:10.1093/gigascience/giac057)
Supplement: giac057_Supplemental_Figures_and_Tables [file giac057_supplemental_figures_and_tables.zip › Supplementary Figures.docx]

**The Manchurian Walnut Genome: Insights into Juglone and Lipid Biosynthesis**


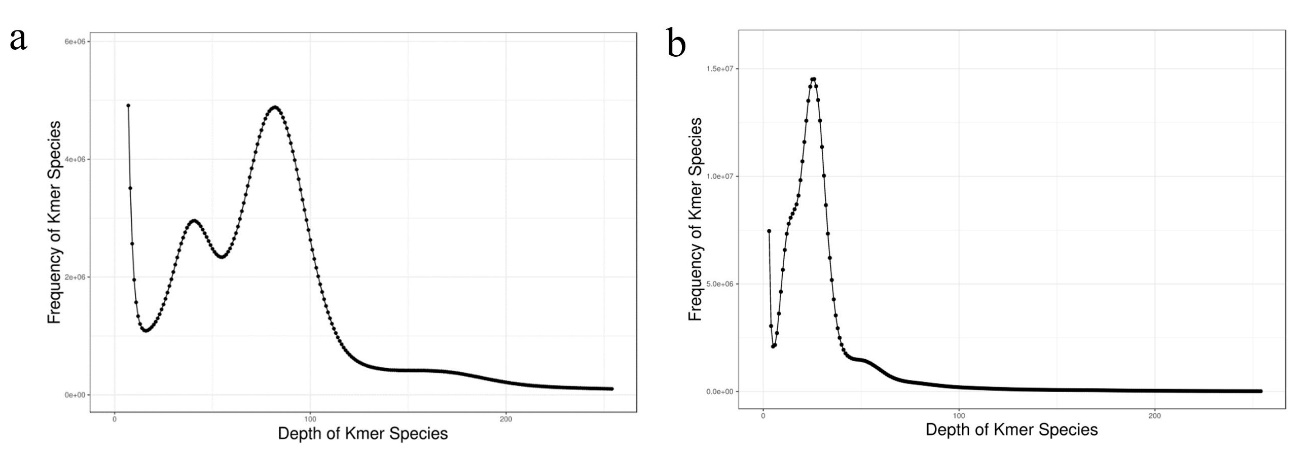


**Figure S1** 17-mer analysis to estimate the *J. mandshurica* genome size based on the PacBio HiFi data. The x-axis and y-axis indicate the 17-mer number and frequency of kmer, respectively.


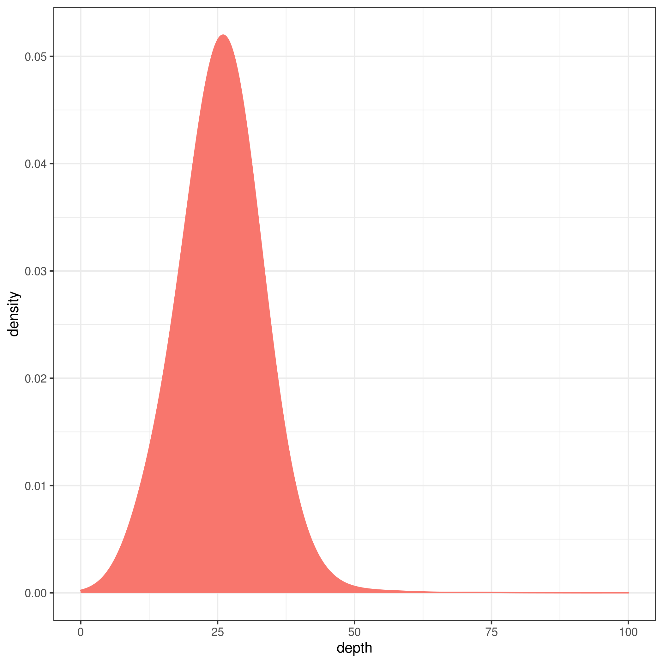


**Figure S2.** Sequencing depth distribution of the assembled *J. manshurica* genome.


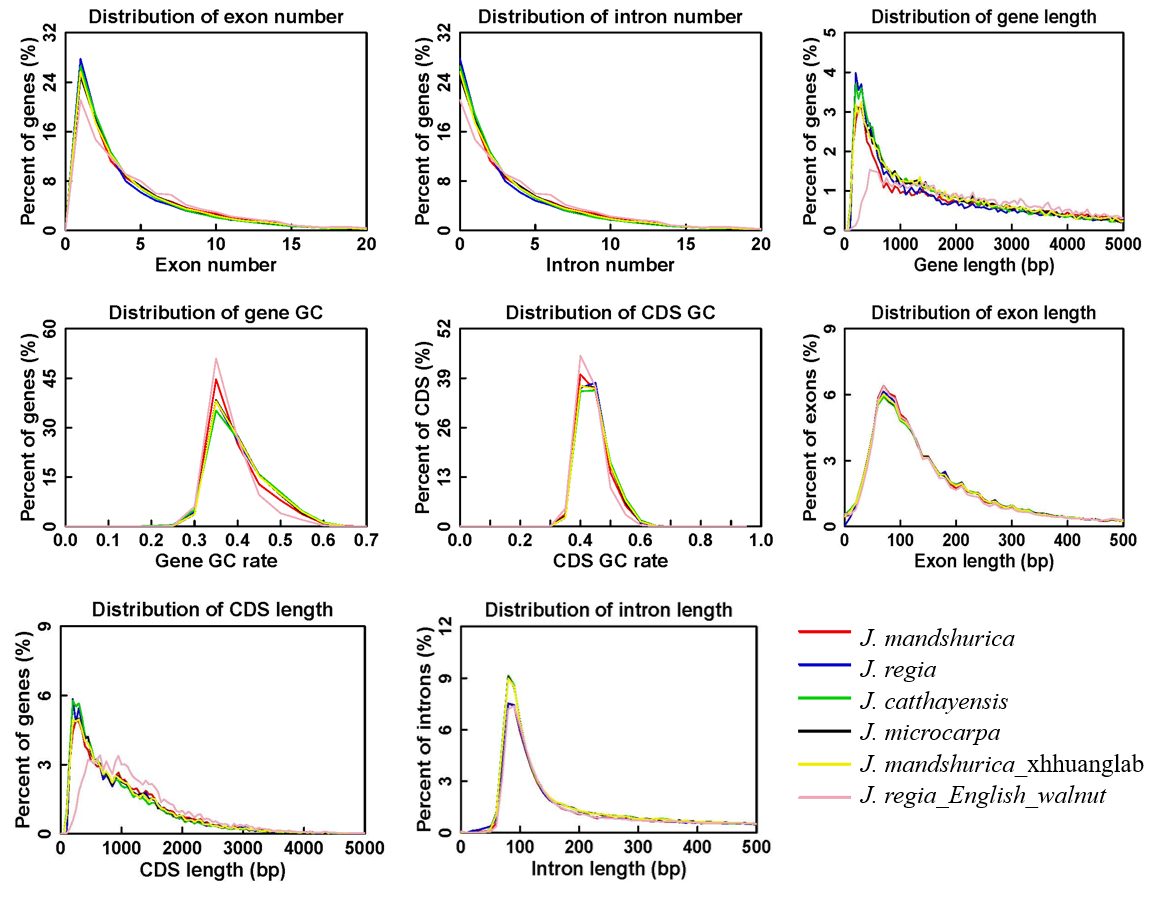


**Figure S3.** Cross-species comparisons of exon number, intron number, gene length, gene GC, CDS GC, exon length, CDS length and intron length distribution.


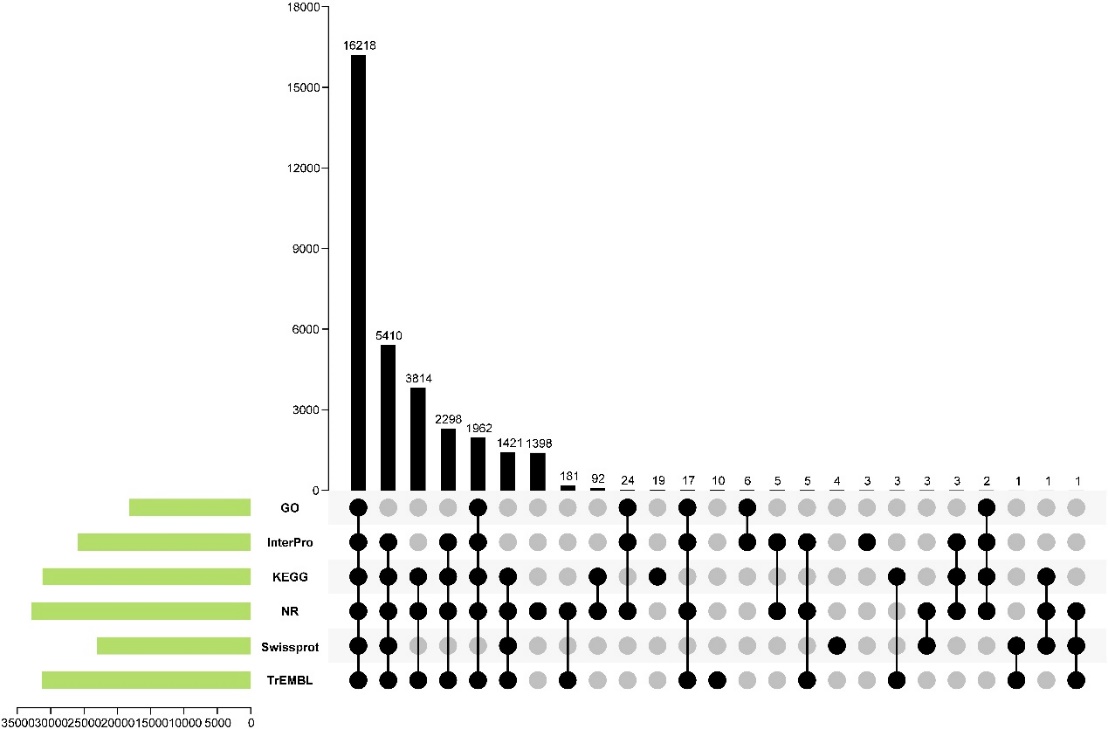


**Figure S4.** Upset plot of genes annotated in GO, InterPro, KEGG, NR, Swissprot and TrEMBL database.


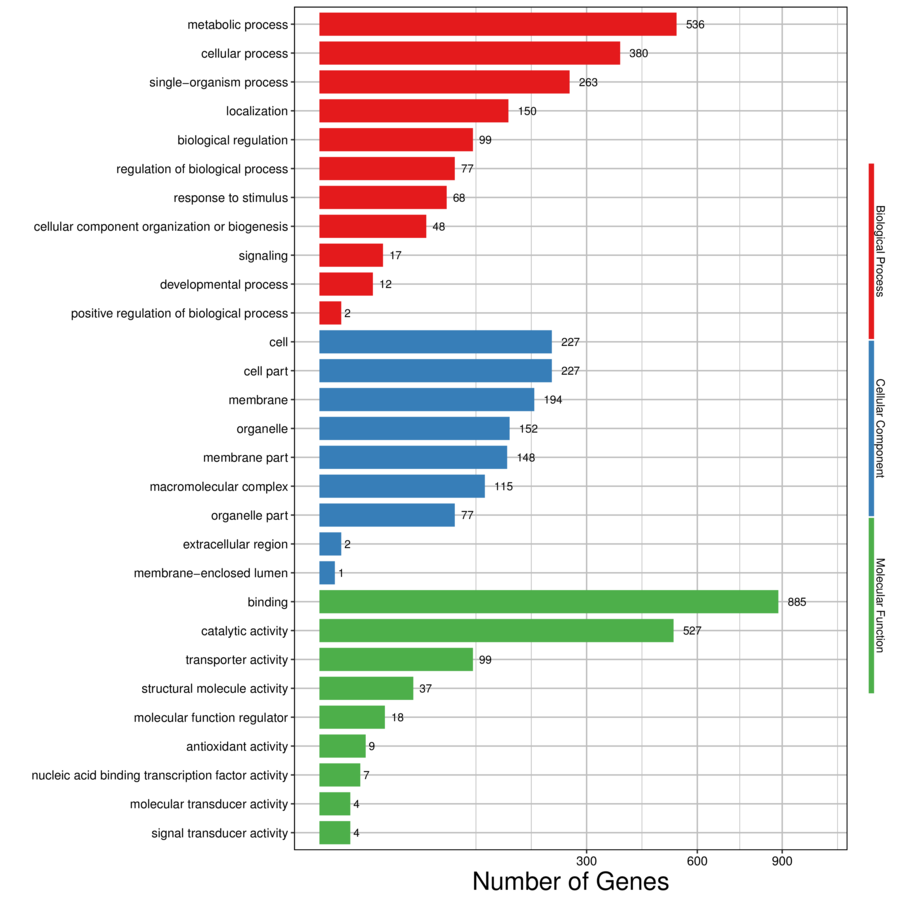


**Figure S5.** The GO category analysis of rapidly expanded gene families in assembly *J. mandshurica* genome. The x-axis indicates the number of genes, and the y-axis represents the GO terms including biological process, cellular component and molecular function.


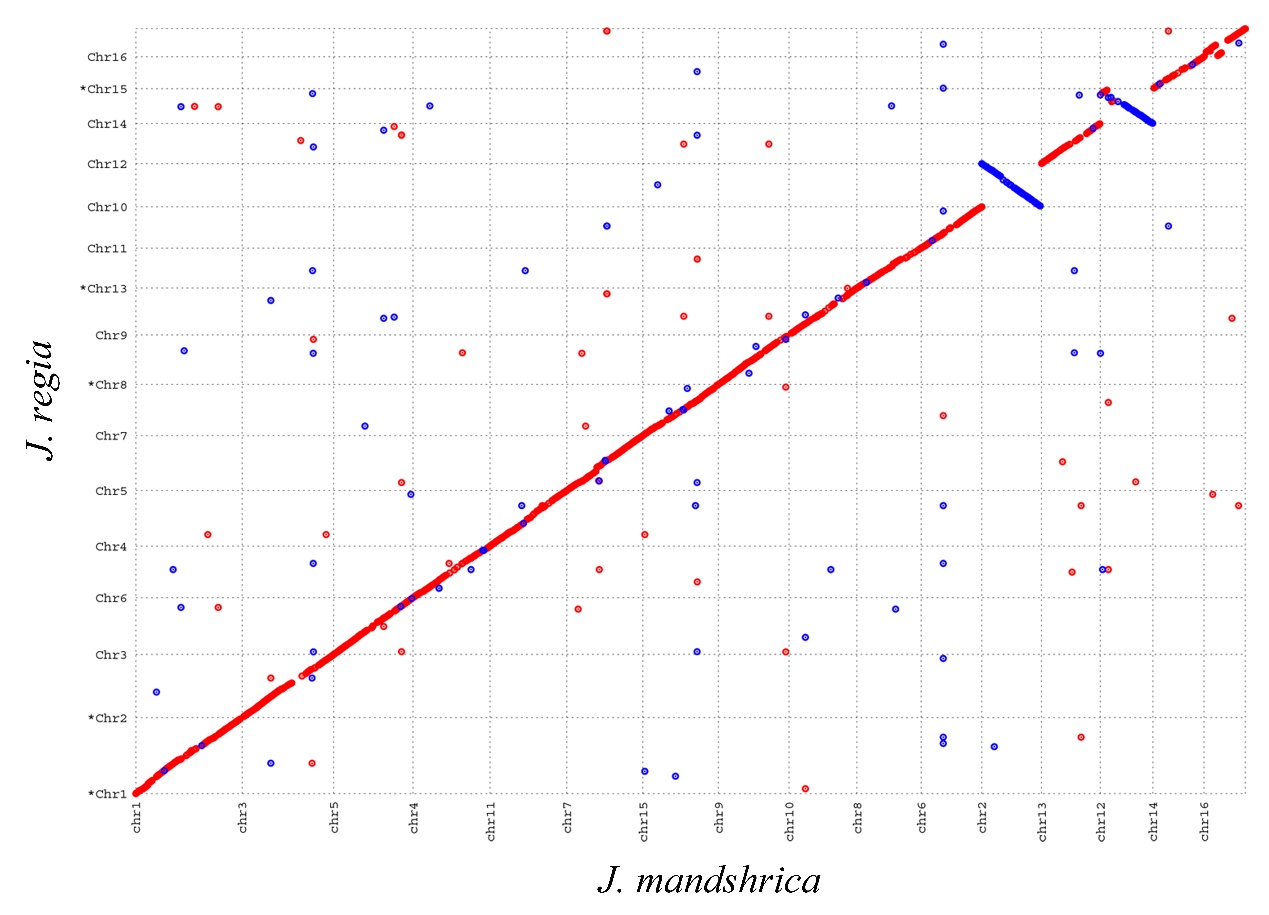


**Figure S6.** Schematic representation of syntenic genes among *J. mandshurica* and *J. regia*. The x-axis indicates the chromosome number from *J. mandshurica*, and the y-axis represents the chromosome number from *J. regia.*


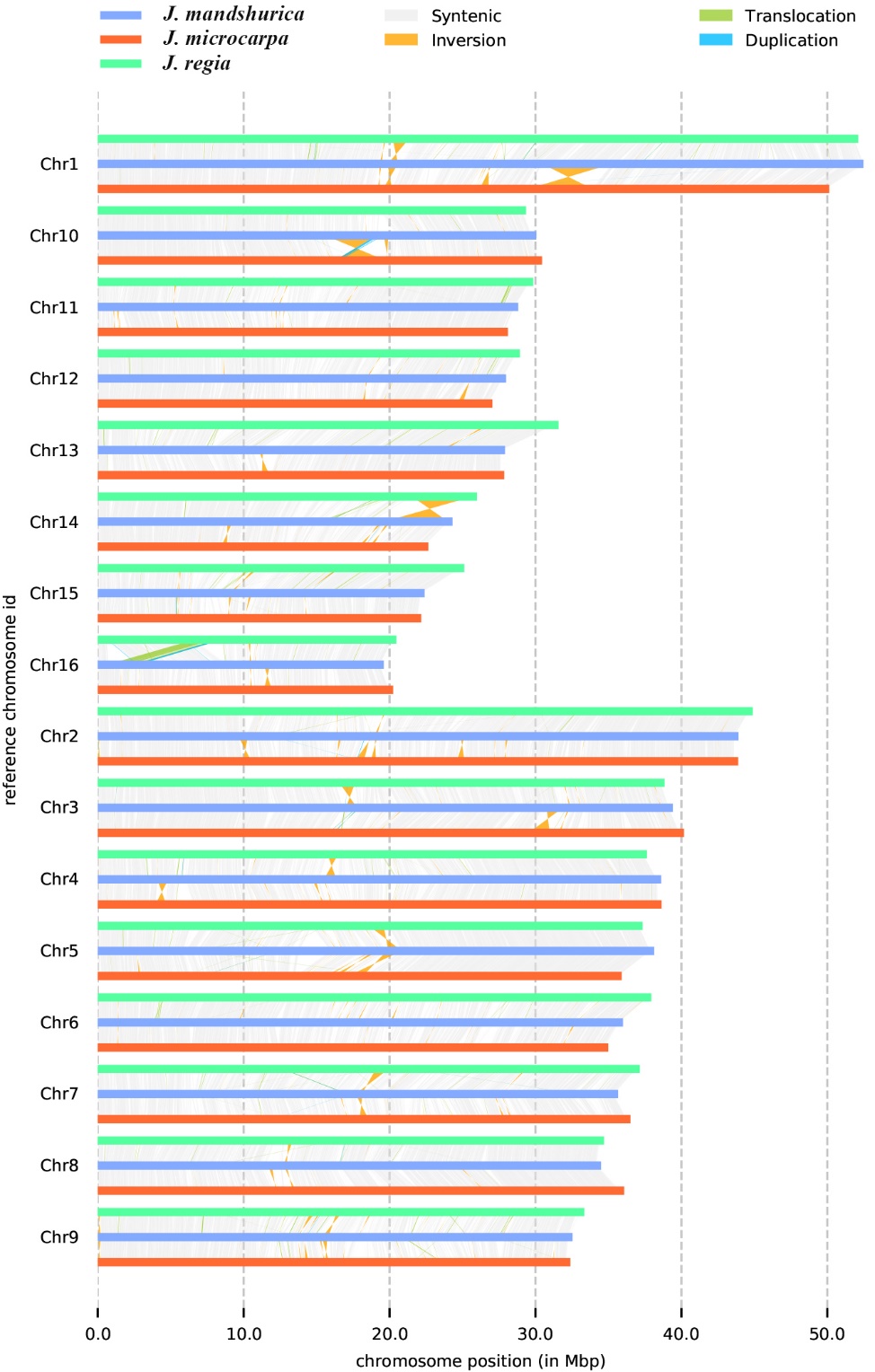


**Figure S7** Genomic variation between *J. mandshurica* and other two walnut species. The red, light green and blue line indicate the *J. macrocarpa*, *J. regia* and *J. mandshurica* genome. The grey line in the background indicates collinear blocks within *J. mandshurica* and other plant genomes.


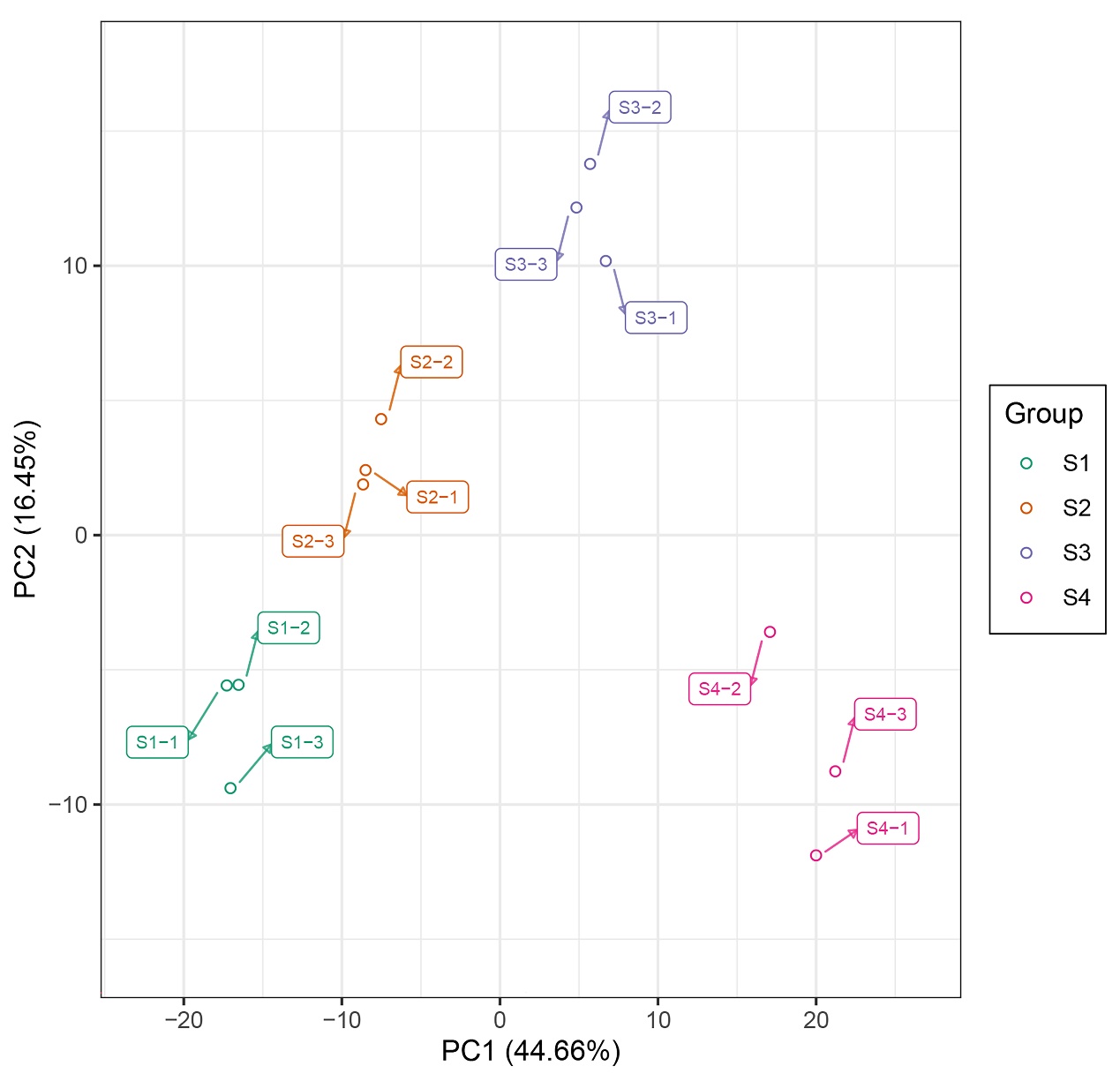


**Figure S8** PCA score plot metabolite profiles from different sample groups during developmental walnut exocarp. The x-axis indicates the PC1 with 44.66%, and the y-axis indicates the PC2 with 16.45%.


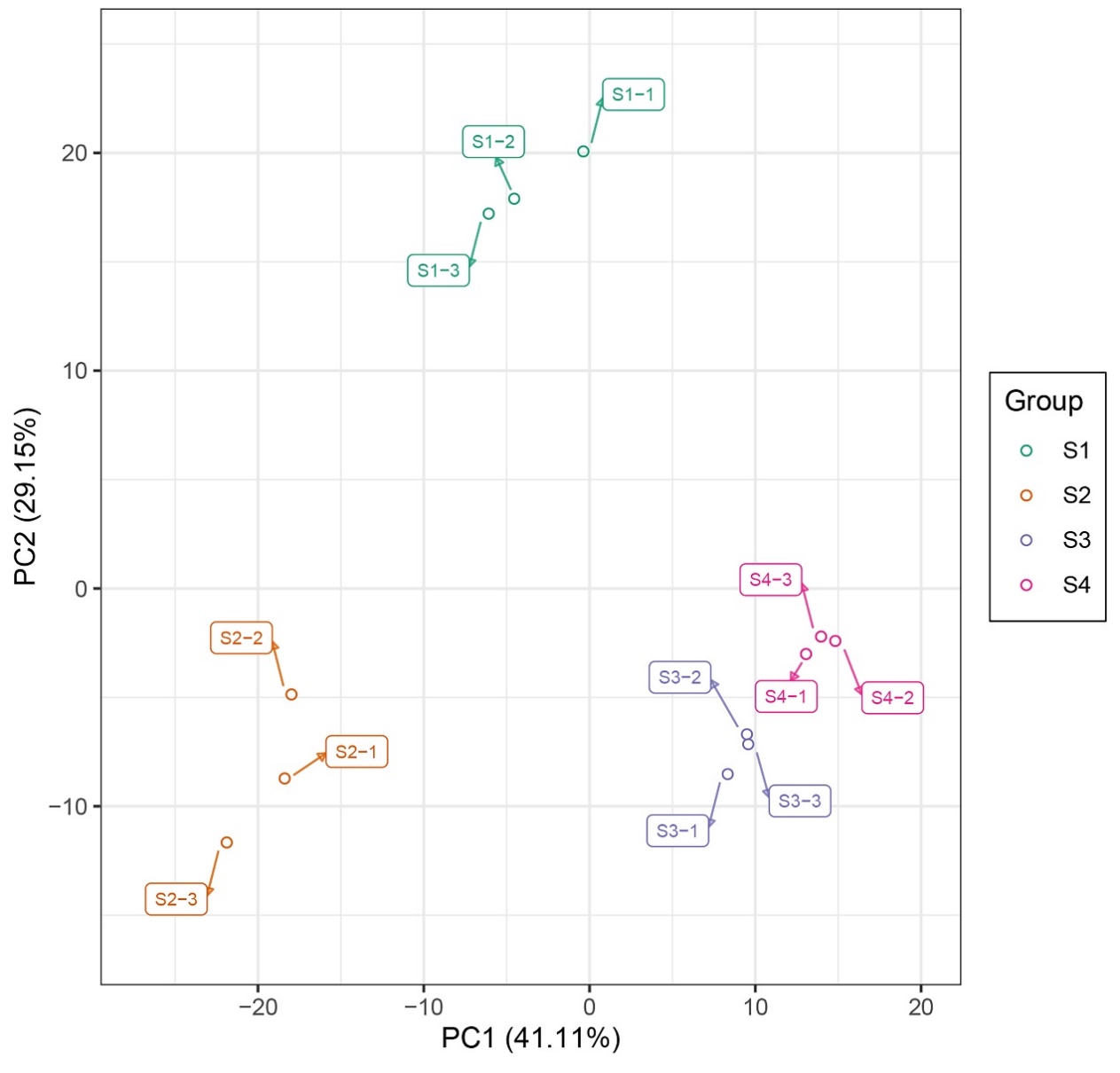


**Figure S9** PCA score plot metabolite profiles from different sample groups during developmental walnut embryos. The x-axis indicates the PC1 with 41.11%, and the y-axis indicates the PC2 with 29.15%.


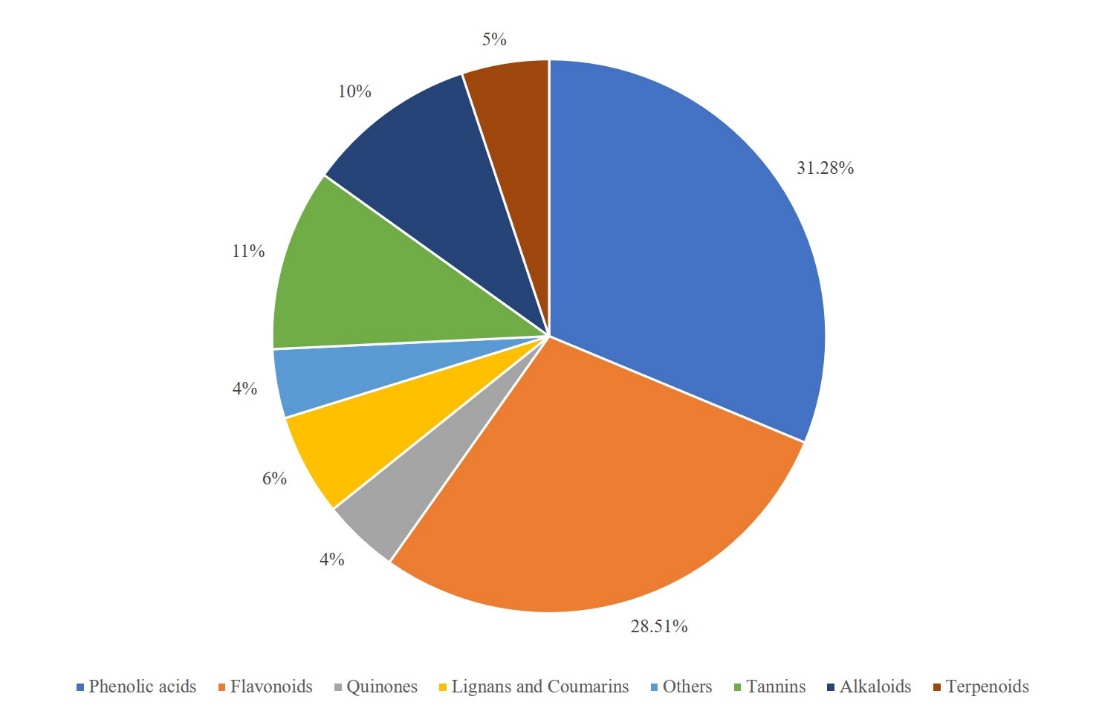


**Figure S10**. Distribution of identified metabolites during developmental green peel in *J. mandshurica*.


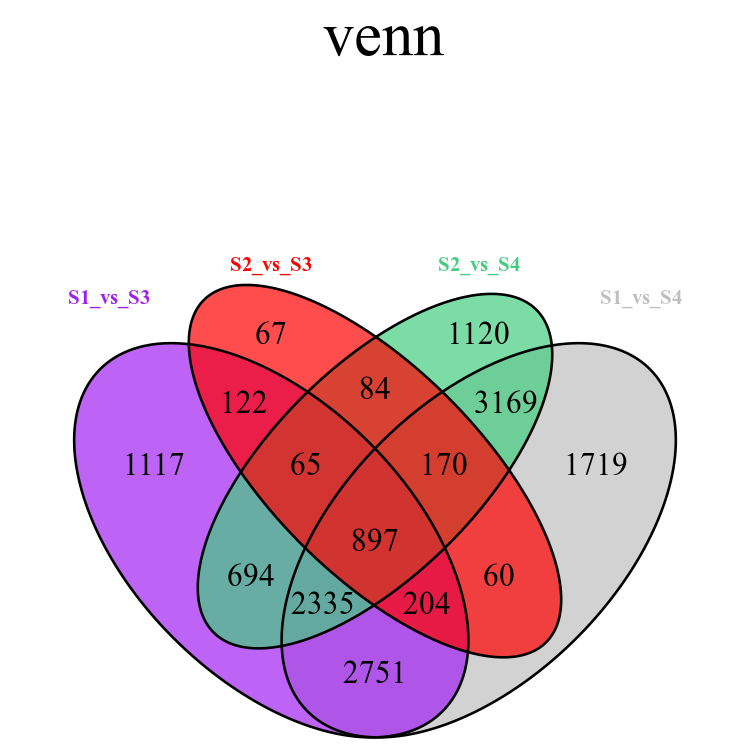


**Figure S11.** Venn diagrams of differentially expression genes (DEGs) in S1_vs_S3, S2_vs_S3, S2_vs_S4 and S1_vs_S4 stages in green peel in *J. mandshurica*.


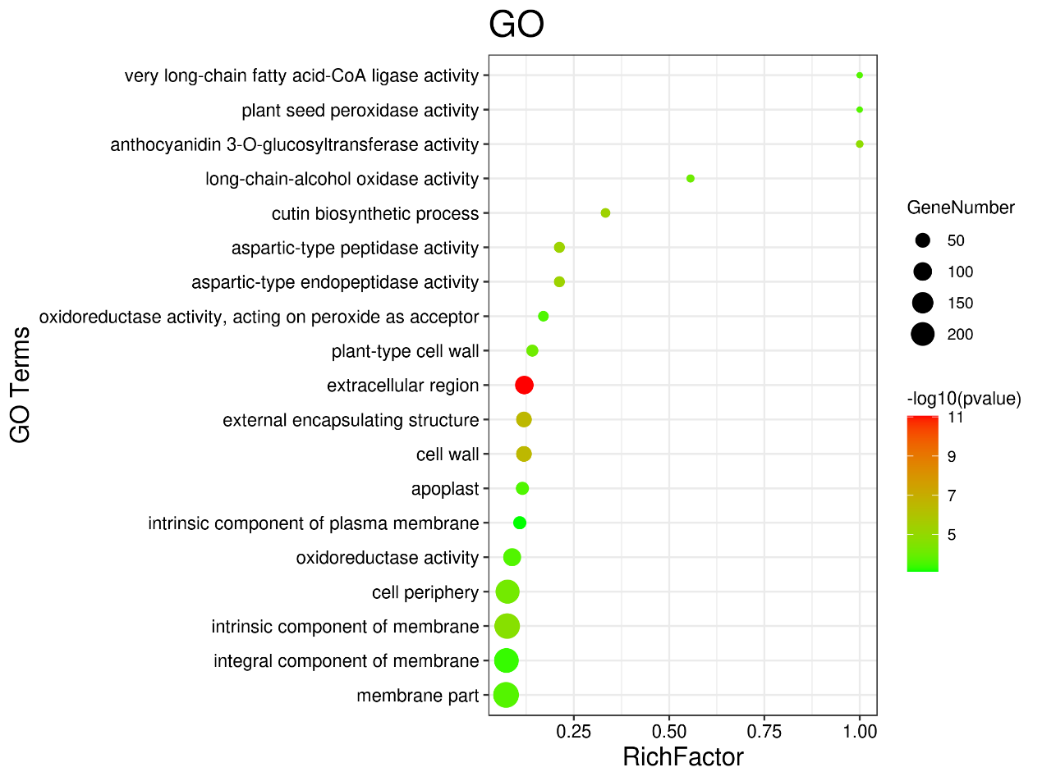


**Figure S12.** GO enrichment analysis of 897 core DEGs in *J. mandshurica*. The x-axis indicates the rich factor, and the y-axis indicates the GO terms. The circle from small to big indicates the gene number from low to high. The color scale from green to red indicates the -log_10_(*p*-value) from low to high.


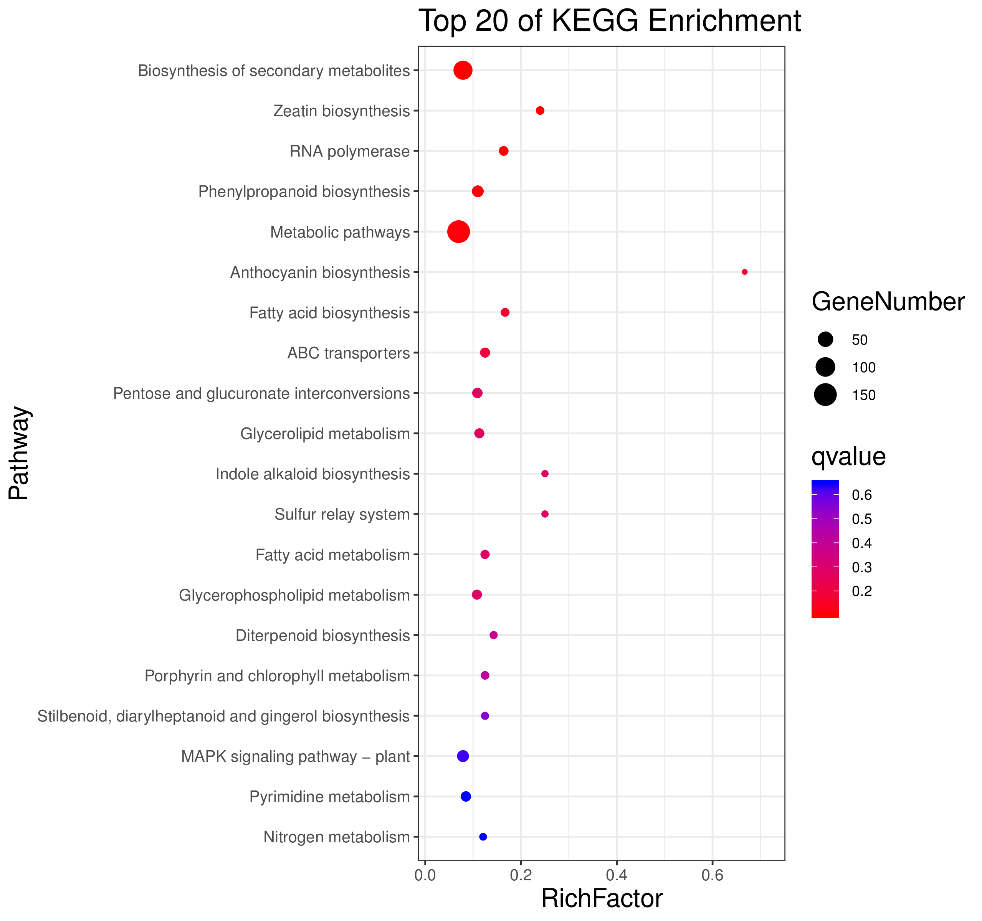


**Figure S13.** KEGG enrichment analysis of 897 core DEGs in *J. mandshurica*. The x-axis indicates the rich factor, and the y-axis indicates the pathways. The circle from small to big indicates the gene number from low to high. The color scale from red to blue indicates the q-value from low to high.


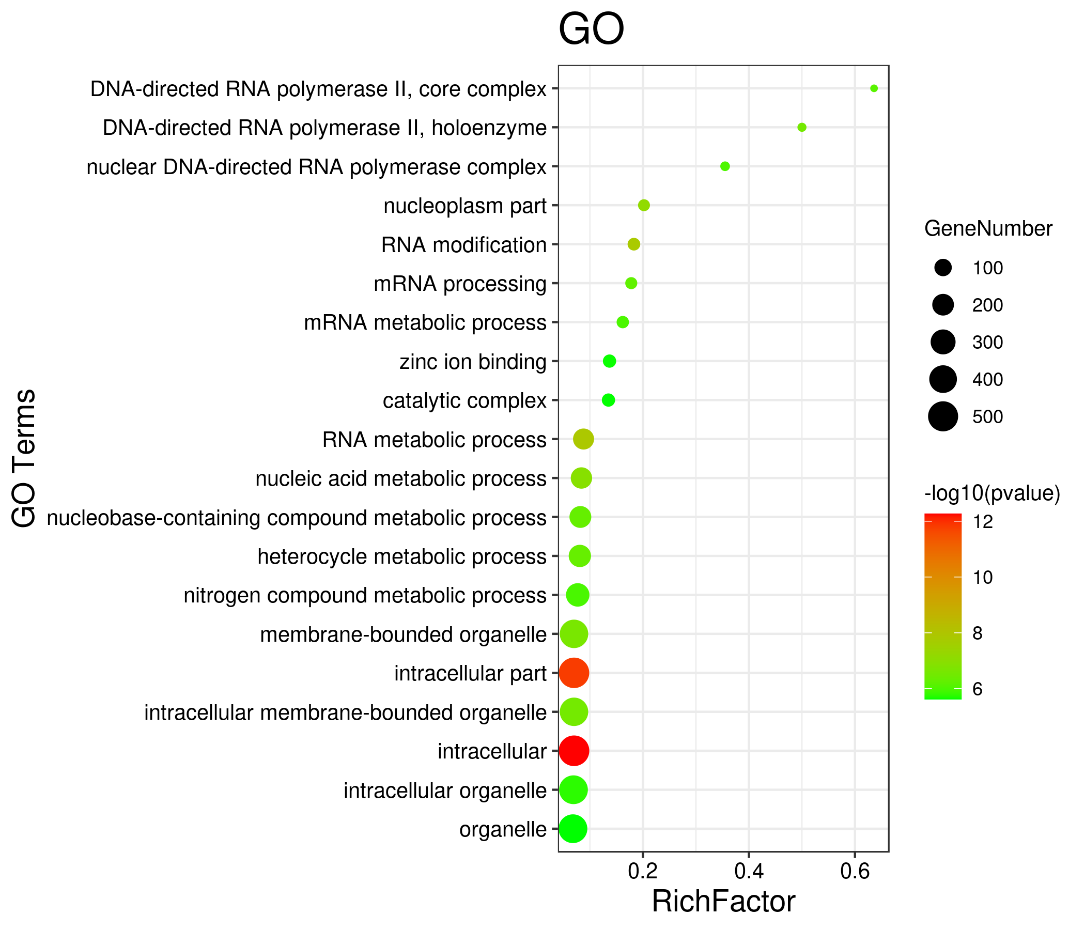


**Figure S14.** GO enrichment analysis of 990 DEGs identified in cluster 6. The x-axis indicates the rich factor, and the y-axis indicates the GO terms. The circle from small to big indicates the gene number from low to high. The color scale from green to red indicates the -log10(p-value) from low to high.


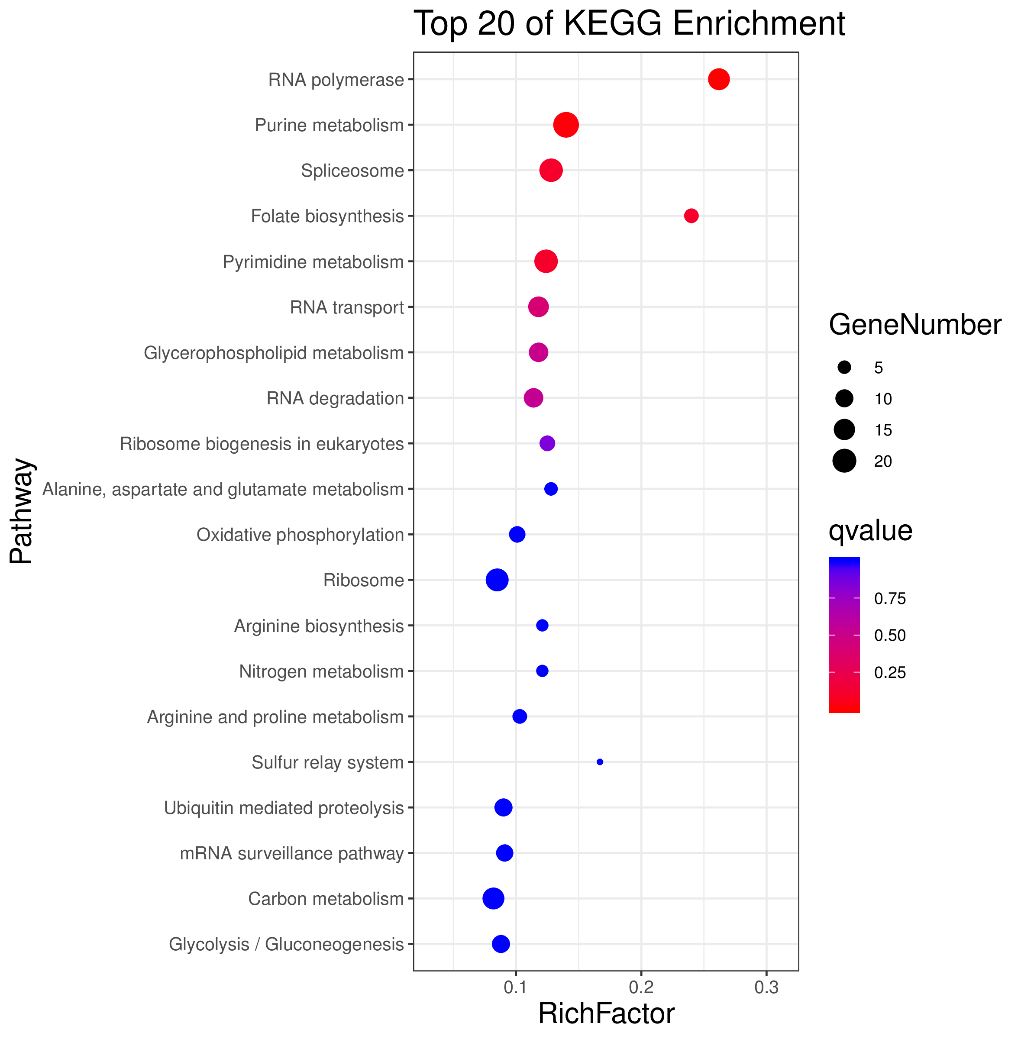


**Figure S15.** KEGG enrichment analysis of 990 DEGs identified in cluster 6. The x-axis indicates the rich factor, and the y-axis indicates the pathways. The circle from small to big indicates the gene number from low to high. The color scale from red to blue indicates the q-value from low to high.


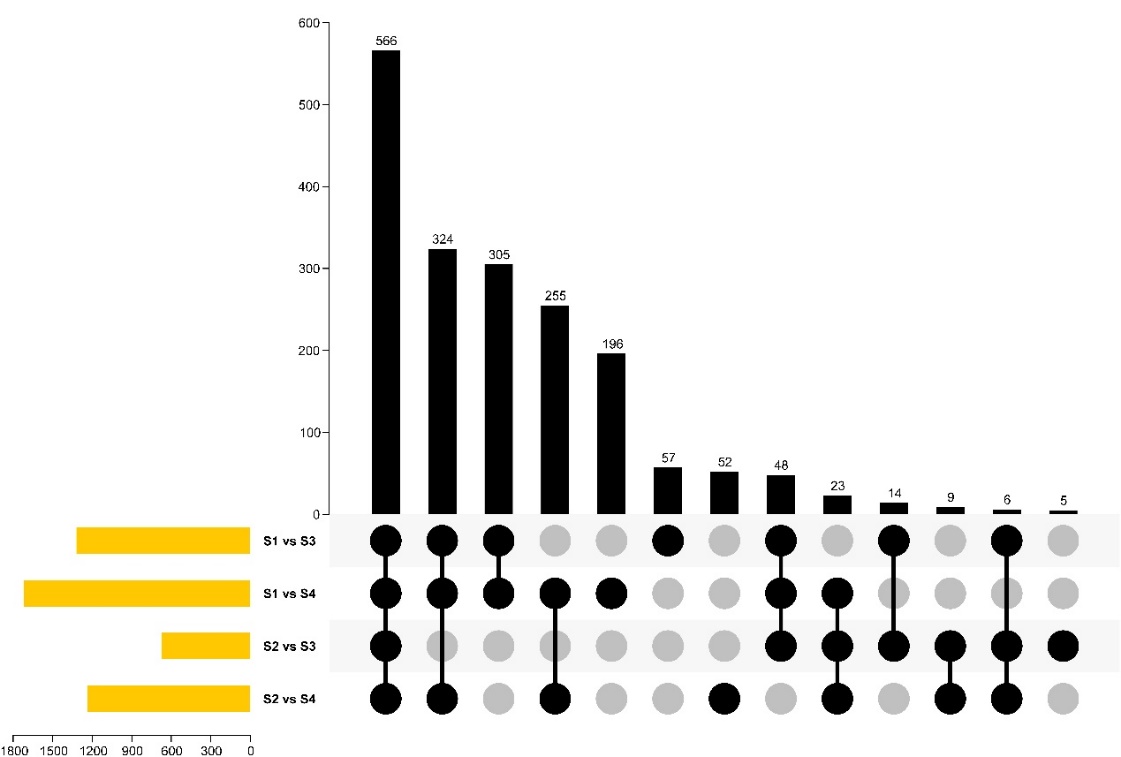


**Figure S16.** Upset plot of differentially expression genes associated with Juglone (*r*>0.8) in S1, S2, S3 and S4 stage in *J. mandshurica*.


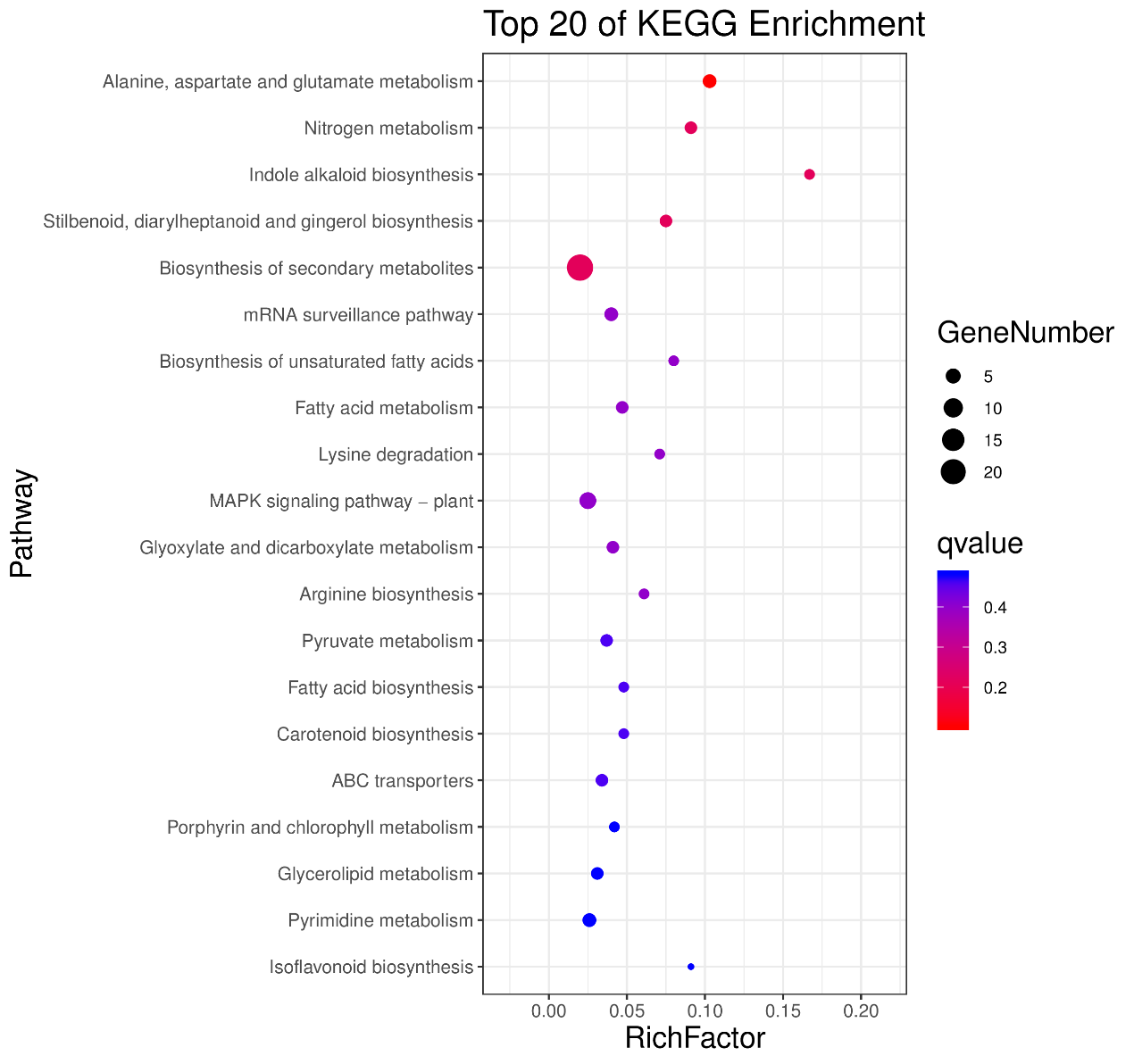


**Figure S17.** KEGG enrichment analysis of 566 core DEGs associated with Juglone (r > 0.8 or < -0.8) in S1 vs S3, S1 vs S4, S2 vs S3 and S2 vs S4 in *J. mandshurica*. The x-axis indicates the rich factor, and the y-axis indicates the pathways. The circle from small to big indicates the gene number from low to high. The color scale from red to blue indicates the q-value from low to high.


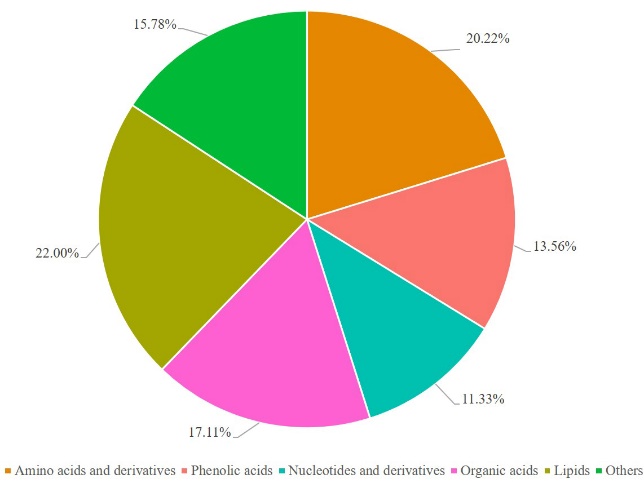


**Figure S18.** Distribution of identified metabolites in walnut kernels in *J. mandshurica*


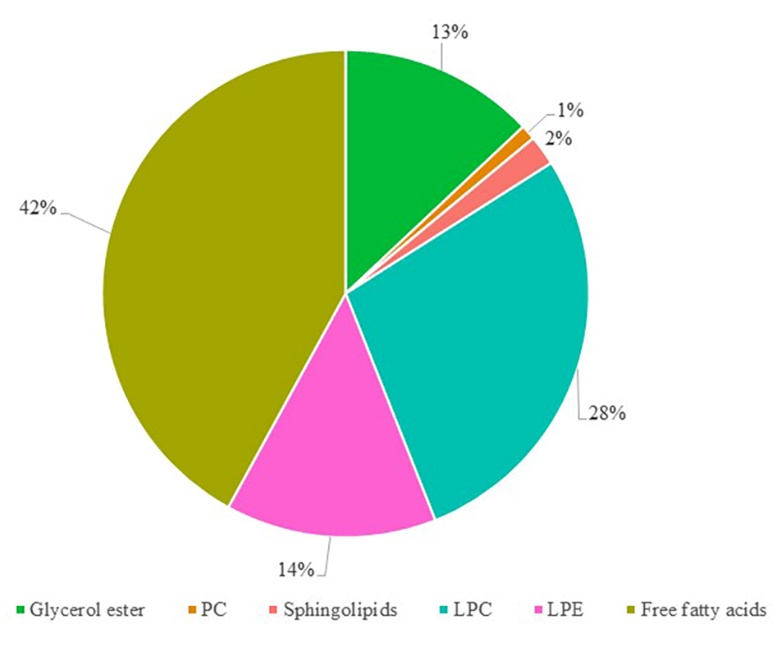


**Figure S19.** Distribution of identified lipid components in walnut kernel in *J. mandshurica*. PC, phosphatidylcholine; LPC, lysophatidylcholine; LPE, lysophatidylethanolamines.


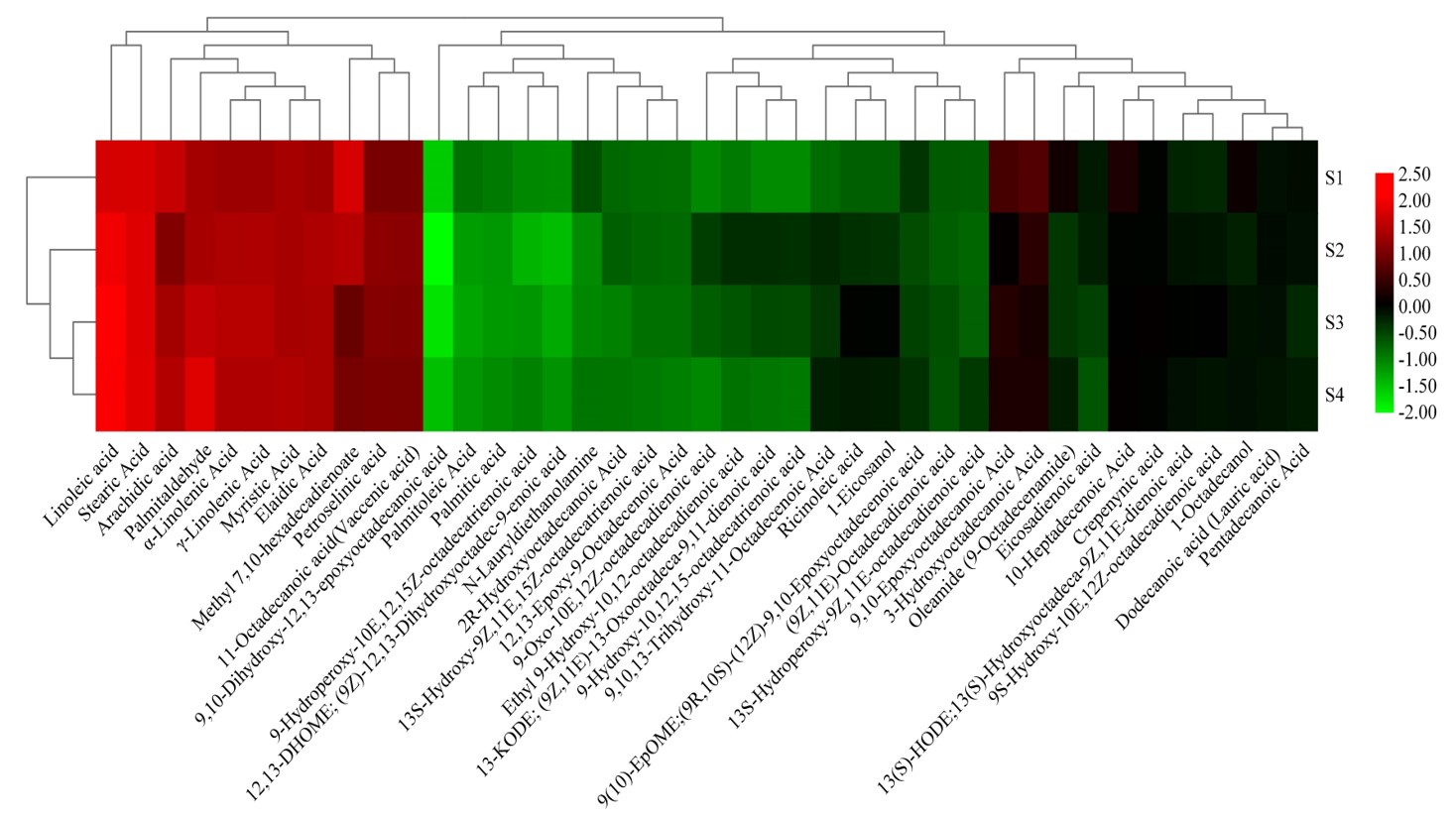


**Figure S20.** Heatmap of the free fatty acids during walnut kernel development in *J. mandshurica*. The color scale from green to red indicates the metabolite content from low to high.


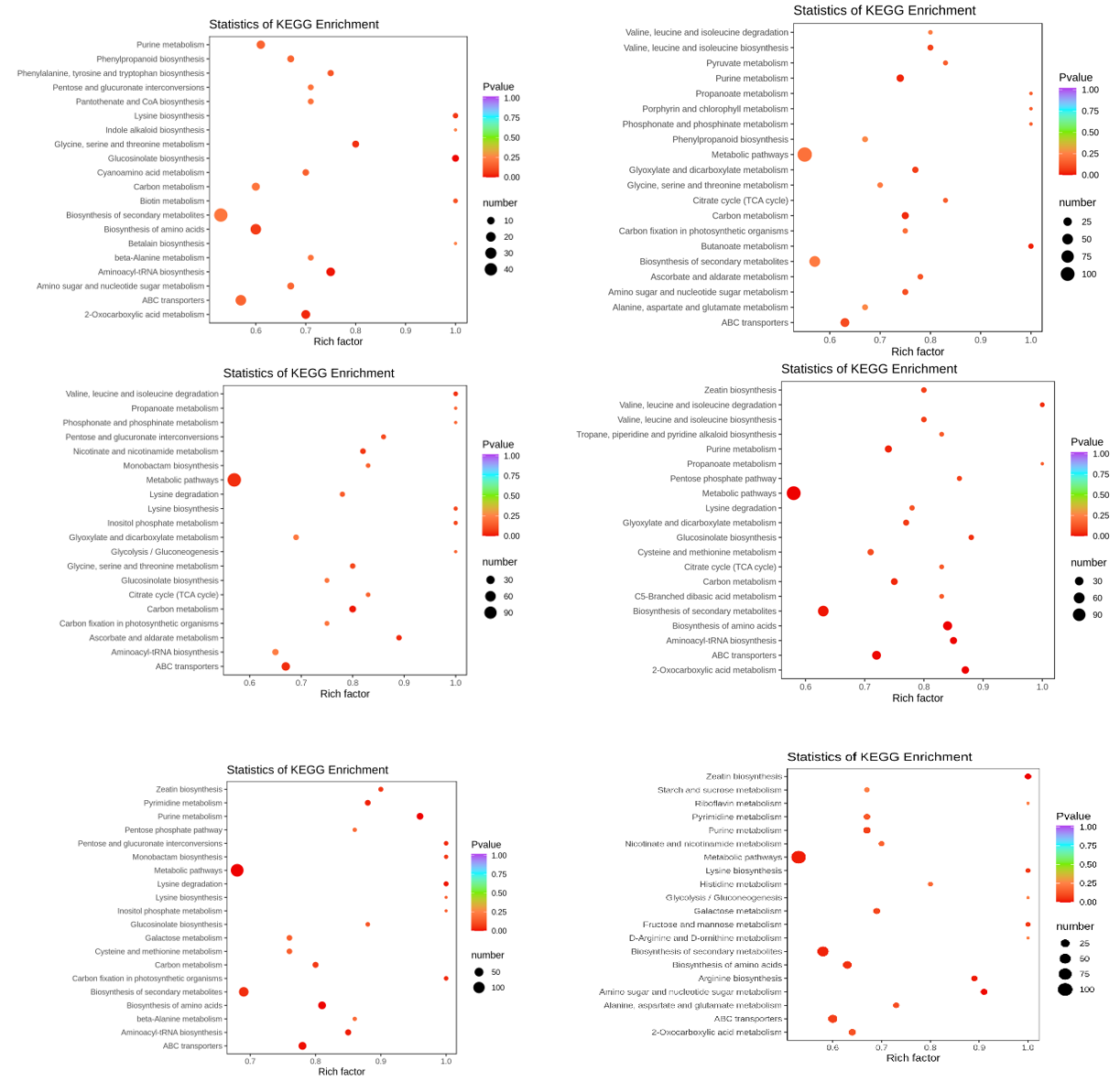


**Figure S21.** KEGG enrichment analysis of different metabolites in six comparison groups. (a) S1 vs S2 (b) S1 vs S3 (c) S1 vs S4 (d) S2 vs S3 (e) S2 vs S4 (f) S3 vs S4.


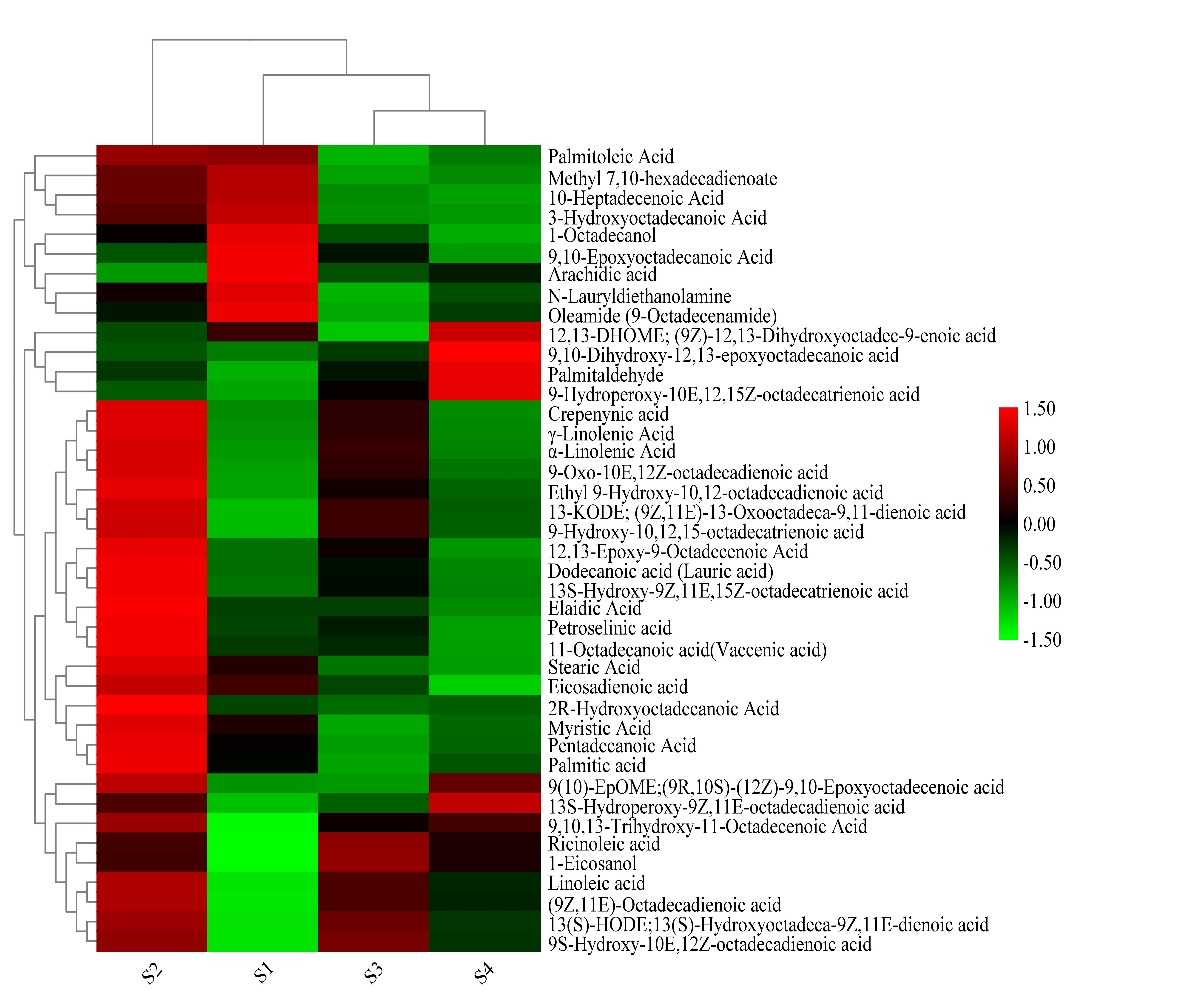


**Figure S22.** Heatmap of the free fatty acids during walnut kernel development in *J. mandshurica*. The color scale from green to red indicates the metabolite content from low to high.


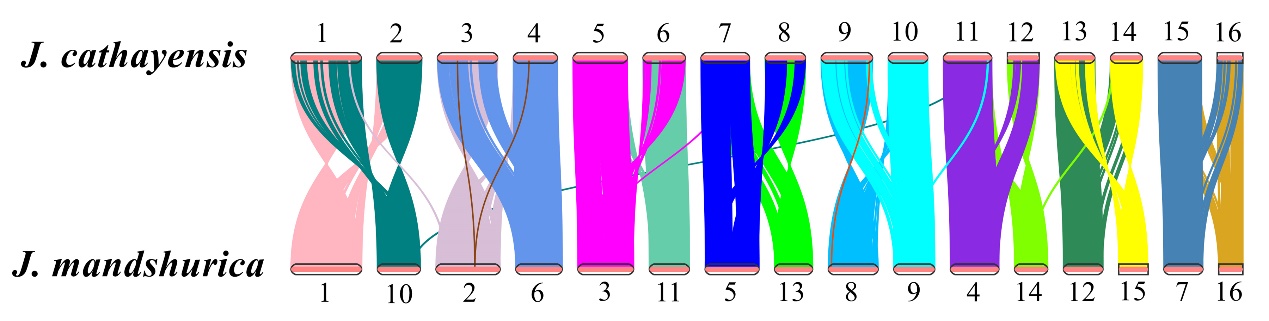


**Figure S23.** The schematic representation of syntenic genes among *J. mandshurica* and *J. cathayensis.* The red lines highlight the syntenic gene pairs.


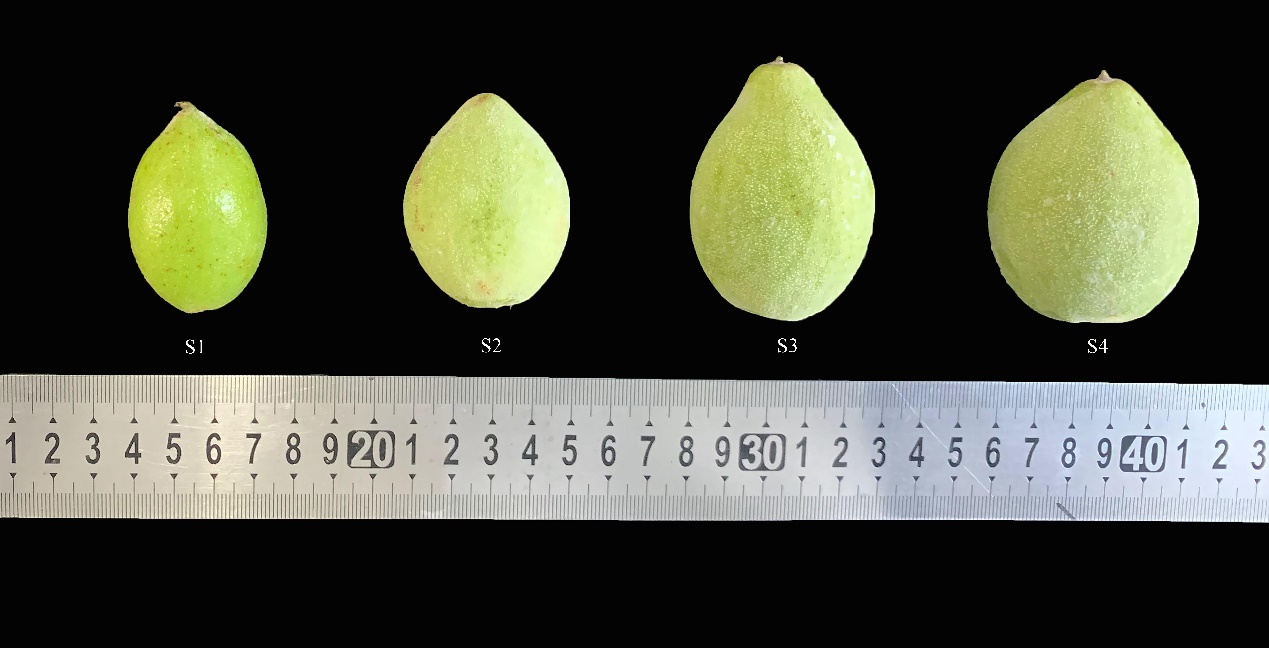


**Figure S24.** Changes of *J. mandshurica* fruits in different development periods. S1-S4 indicate the fruit collected at 30 days (S1 stage), 50 days (S2 stage), 70 days (S3 stage) and 90 days (S4 stage) after natural pollination.


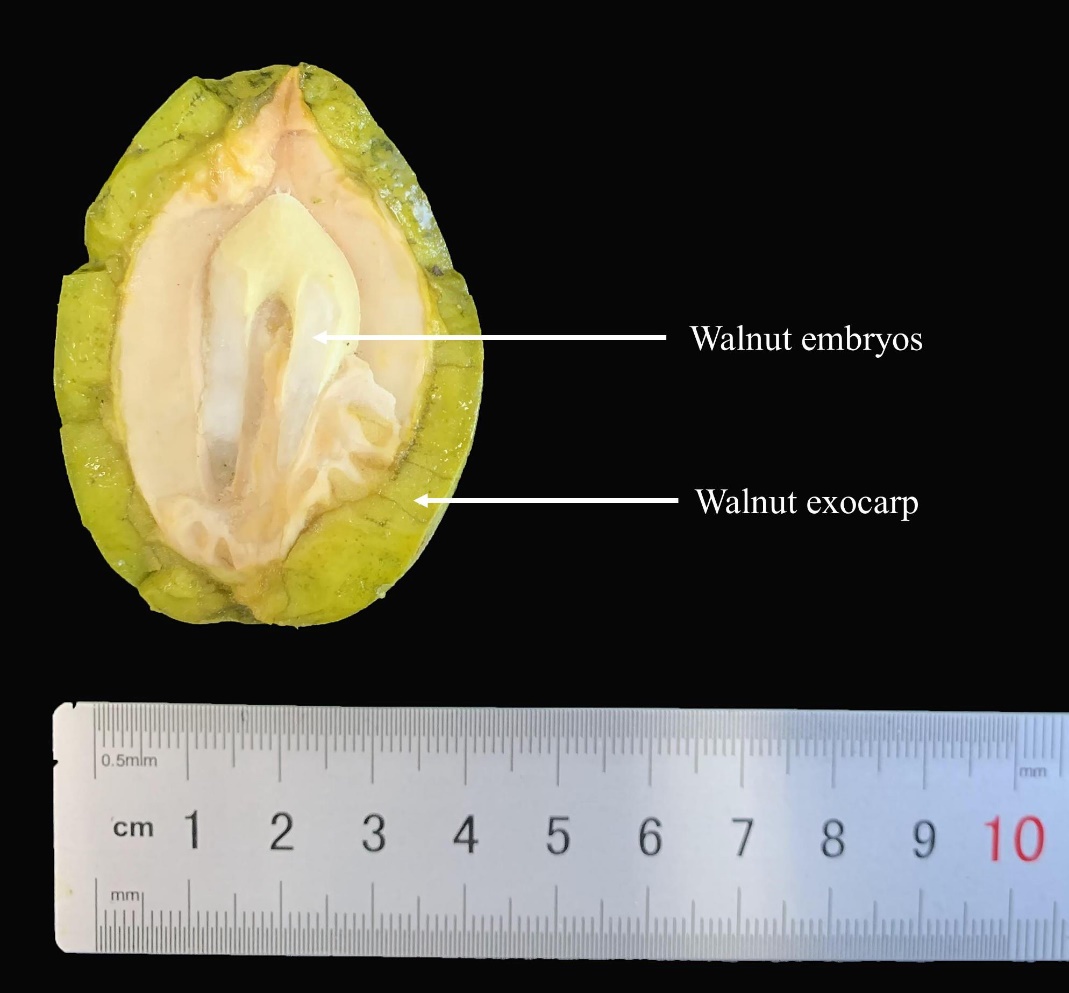


**Figure S25.** The tissue structure of *J. mandshurica* fruit including the walnut exocarp and walnut embryos.
