# Supplementary material for: The Manchurian Walnut Genome: Insights into Juglone and Lipid Biosynthesis
Source: Gigascience. 2022 Jun 28;11:giac057. doi: 10.1093/gigascience/giac057 (PMC9239856; doi:10.1093/gigascience/giac057)
Supplement: giac057_Response_to_Reviewer_Comments_Original_Submission [file giac057_response_to_reviewer_comments_original_submission.pdf]

Author's Response To Reviewer Comments

Close

Manuscript ID: GIGA-D-21-00355
Dear Editor-in-chief and Reviewers:

Thank you for your letter again and for the reviewers' comments concerning our manuscript entitled "The Manchurian Walnut Genome: Insights into Juglone and Lipid Biosynthesis" (GIGA-D-21-00355). Those comments are all valuable and very helpful for revising and improving the quality our paper. We have studied all comments carefully and have made corrections which we hope meet the standard of your highly esteemed journal. The main corrections in the paper and the responses to the reviewers' comments are given below:

Response to Reviwer 1:
(1) A high-quality genome is an important genetic resource for the improvement of horticultural traits in perennial crops. A combination of long reads (Nanopore sequencing platform), Illumina and Hi-C auxiliary assembly can be used to produce a high-quality, chromosome-level genome. Recently, the availability of high-throughput sequencing has accelerated the publication of the genomes of walnut (Juglans) species and hybrids (J. regia × J. microcarpa) (Martínez-García et al., 2016; Stevens et al., 2018; Bai et al., 2018; Zhang et al., 2020; Yan et al., 2021). For Juglans mandshurica, it has been published three assembly genomes, including scaffold lever (Bai et al., 2018; Stevens et al., 2018) and high-quality chromosome-level (Yan et al., 2021). Therefore, it is necessary to perform a comparison using multiple genomes, especially previous versions of J. mandshurica. To compare the GC content and size of genome (in this study, genome size of ~548.7 Mb and Yan et al., 2021genome size of ~548.5 Mb, only 0.2 Mb difference), which are did not showing anything about the high-quality of the genome assembly from Line 601 to Line 613. Furthermore, the author described "the best available reference genome of J. mandshurica" is from Line 611 to Line 613 is inaccurate statement, please revise it. It is necessary to compare the scaffolds N50 size, mapped and anchored percentage, and BUSCOs. The comparison may give some interesting discoveries, such as TEs, InDels and SVs between two genomes.
Response: Thanks for your comments. The sentence has been corrected, see Line 477-479. For Juglans mandshurica, it has been published only two assembly genomes, including scaffold lever (Bai et al., 2018) and high-quality chromosome-level (Yan et al., 2021). In Stevens et al., study, the genome of Juglans mandshurica was not assembled. Therefore, here, the genome information of Juglans mandshurica in present study was compared with two previously reported assemblies for this species. See Table 1. It is obvious that the contig N50 (21.4 Mb) and complete BUSCO (98.3%) of Juglans mandshurica (V 3.0) were significantly higher than that in previously reported assemblies, and the scaffold N50 was higher than that in Juglans mandshurica (V 1.0), but similar to Juglans mandshurica (V 2.0). Although the anchored rate in present study (96.26%) was lower than that in Juglans mandshurica (V 2.0) (99%), the number of anchored genes (38940) were significantly higher than Juglans mandshurica (V 2.0) (28742). These results indicated that our assembly maintain a relatively high level compared with previously reported genome of this species. Additionally, because of the vastly climate difference of sampling location (Harbin vs Qinling Mountain in V 3.0 vs V 2.0), which would cause specific environmental suitability and genetic differentiation for this species. There are differences for the number of variable sites, such as TEs. Particularly, although the Juglans mandshurica (V 2.0) has been published, the assembly and annotation data were still unpublic, which limited subsequent molecular study for this species to some extent. Thus, it is difficult for us to further carry out the comparative genome analysis for these two assemblies for this species.

(2) In this study, the author used total of 15 species to analyze the comparison genomes, including seven Juglans species. I question the value of the phylogenetic analysis of fifteen species from across all angiosperm lineages. This does not seem to provide any new significant insights. It is necessary to perform a comparison expansion and contraction gene families within Juglans species. J. mandshurica a synonym of J. cathayensis Dode in Flora of China. However, a significantly different for contraction gene families between J. mandshurica (405) and J. cathayensis (2193), which is not consistently with the taxa and gene family evolution. The authors need to explain the reasons for the two J. mandshurica genome

and J. cathayensis genome. What is the deep relationship for J. mandshurica and J. cathayensis based on the high-quality genomes?

Response: For the relationship of J. mandshurica and J. cathayensis, it is still controversy at present. In Flora of China, J. mandshurica was consider as a synonym of J. cathayensis Dode according to the phenotypic characteristic, but there is no definitive molecular evidence at present at the genome level. Here, the synteny analysis and genome-wide alignment between J. mandshurica and J. cathayensis in present study was performed by jcvi and Last software, respectively. See Supplementary Fig. S22 and Table 2. The results showed that the number of protein coding genes in J. mandshurica (40453) was similar to that in J. cathayensis (39905). However, the synteny analysis found that approximately 60% of the J. mandshurica genome was colinear with J. cathayensis. Additionally, the alignment analysis suggested that the obtained identity genes (identity >=90%) approximately accounted for ~66% of genome. According to these results, we speculated that J. mandshurica and J. cathayensis are eventually established as two separate species status at the genomic level.

(3) The collinearity of J. mandshurica and J. regia genomes were compared using 16 chromosomes in Figure 2. I question the order of chromosome numbers J. mandshurica and J. regia genomes are uniformity or not? It is important of order of chromosome numbers for two Juglans species in the length, gene distributions, and collinearity.

Response: Thanks for comments. For J. mandshurica (V 2.0), the chromosome numbering was based on homology to the numbering of J. regia chromosomes. However, in present study, chromosome numbering for J. mandshurica was based on the size of chromosomes from to maximum (Chr1) to minimum (Chr16), See Line 114-116. Thus, there are difference for the order of chromosome numbers for these two species. We have corrected the Figure 4c according to the collinearity relationship of between J. mandshurica and J. regia.

(4) The whole-genome duplication events (WGD) showed that the peak of Ks from 0-0.5 and 0-0.25 using Ks and 4DTV, respectively. It is necessary to compare the WGD in previous walnut genomes studies and discuss it.

Response: We have added the WGD compared with other walnut genomes in discuss Part. See line 505-510.

(5) Along with the development of sequencing technology, to get a relative high-quality genome is not a challenge now. I would expect to see some deeper analyses based on the genome assembly. For example, to explain why J. mandshurica is abundant juglone, potential as a medicinal crop. Furthermore, the other main weakness of this manuscript is short of comprehensive discussions and interpretations for the relationship between genomic characters and biology of the J. mandshurica. In 2016, Martinez-Garcia et al report the complex pathways required for the synthesis of polyphenols using the J. regia genome. However, the authors of this article did not conduct any comparative research on the relevant complex pathways results, such as juglone and lipid biosynthesis.
Response: The relative discuss contents involved in juglone and lipid biosynthesis have been added to the discuss Part. See Line 568-590.

(6) For the transcriptome and metabolic samples, the authors collected J. mandshurica fruits in different development periods. S1-S4 indicate the fruit collected at 30 days (S1 stage), 50 days (S2 stage), 70 days (S3 stage) and 90 days (S4 stage) after natural pollination. It is necessary to describe the walnut exocarp and walnut embryos in each figure.
Response: Thanks for your comments. The walnut exocarp contains a large amount of tannin juice, which is easily oxidized in air. Tannin and gallic acid will become black after oxidation. In the early stage, the walnut embryos were immature, and have a lot of water. Thus, it is difficult to describe the detail phenotype feature of walnut exocarp and walnut embryos. The samples sampled at 30 days and 50 days do not have hard kernel, whereas the samples at 70 days and 90 days have hard kernel after natural pollination. The relative contents have been added to the materials, see Line 756-759. Additionally, the genes and metabolites changed a lot for four different stages.

(7) Did the author used fossil data to double check the divergence time estimation analysis? The outgroup species should be different genus species within Ranunculaceae family will be good for divergence time estimate analysis. Please explain why the phylogenomic analysis showed that the J. mandshurica was closest to J. cathayensis (divergence at ~13.8 mya).
Response: In present study, the bifurcation time was estimated with five corrected divergence time

point from the TimeTree website (http://www.timetree.org/): O. sativa vs. J. mandshurica (115-308 Mya), V. vinifera vs. J. mandshurica (107-135 Mya), P. trichocarpa vs. J. mandshurica (101-131Mya), Q. lobata vs. C. mollissima (6-49 Mya), Q. lobata vs. J. mandshurica (51-87 Mya), and two corrected divergence time point from M. rubra genome article: M. rubra vs Juglans genus (28-34 Mya) and Carya genome article: genus Juglans vs. genus Carya (~23 Mya). Here, although the phylogenomic analysis showed that the J. mandshurica was closest to J. cathayensis, they still possess a relatively larger divergence time (~13.8 mya), similar results were found in previous study [1]. Using eight chloroplast DNA regions, one single-copy nuclear gene, and 17 nuclear microsatellite loci, the study found that the genetic data consistently identified two clades with estimated divergence time of ~10.93 mya, one northern, comprising J. mandshurica and J. ailantifolia, and one southern, comprising J. cathayensis. Particularly, the two clades diverged through climate-induced vicariance of an ancestral northern range during the mid-Miocene and remained mostly separate thereafter, with geographical isolation of the Japanese Islands and refugial isolation or secondary contacts in the late Pleistocene producing further subdivision within the northern clade. These results further contribute to the fact that J. mandshurica and J. cathayensis are eventually established as two separate species status. See line 488-503.

[1] Bai WN, Wang WT, Zhang DY. Phylogeographic breaks within Asian butternuts indicate the existence of a phytogeographic divide in East Asia. New Phytol. 2016 Mar;209(4):1757-72.

(8) Use of English language. I have noticed the manuscript is written in poor English with many grammatical errors. Please revise the describes logically, organized, and be concise and to the point of RESULTS parts, especially for the "Metabolite profiling and transcriptomics of walnut exocarp development" and "Gene discovery for lipid biosynthesis and oil body formation"
Response: The grammar of manuscript has been checked and polished by editing company.

(9) Please check the numbers consistent with scientific count in the text.
Response: We have checked and corrected the numbers in the manuscript.

(10) Figure legends: please describe the details (legend) of small letter.
Response: We have checked and modified the Figure legends.

(11) It is necessary to improve the quality of Supplementary Figures including legends and data supports.
Response: The Supplementary Figures have been corrected.

Response to Reviwer 2:
(1) There were several genomes for species in Juglans. In 2021, another group leaded by Zhao from Northwest University, China, also published the genomic data of Juglans mandshurica on molecular ecology resource (DOI: 10.1111/1755-0998.13394). In that paper, the author said Juglans mandshurica is a synonym of J. cathayensis Dode. So, what is the different between this study to Zhao`s paper? Though I don`t agree Juglans mandshurica is a synonym of J. cathayensis, but the material used in Zhao`s paper was Juglans mandshurica (not J. cathayensis). If the two papers published the same genome, then there was no novel knowledge for this manuscript in the genome level. Additionally, there were also some other genomes from Juglans released in recent years (Such as from J. regia genome from Pei`s group). In my opinion, the author should not ignore these genomes, at least, a comparison among these genomes should be conducted, especially for your and Zhao`s work.

Response: Thanks for your comments. For Juglans mandshurica, it has been published only two assembly genomes, including scaffold lever (Bai et al., 2018) and high-quality chromosome-level (Yan et al., 2021). Therefore, here, the genome information of Juglans mandshurica in present study was compared with two previously reported assemblies for this species. See Table 1. It is obvious that the contig N50 (21.4 Mb) and complete BUSCO (98.3%) of Juglans mandshurica (V 3.0) were significantly higher than that in previously reported assemblies, and the scaffold N50 was higher than that in Juglans mandshurica (V 1.0), but similar to Juglans mandshurica (V 2.0). Although the anchored rate in present study (96.26%) was lower than that in Juglans mandshurica (V 2.0) (99%), the number of anchored genes (38940) were significantly higher than Juglans mandshurica (V 2.0) (28742). These results indicated that our assembly maintain a relatively high level compared with previously reported genome of this species. Additionally, because of the vastly climate difference of sampling location (Harbin vs Qinling Mounain in V 3.0 vs V 2.0), which would cause specific environmental suitability and genetic differentiation for this species. There are differences for the number of variable sites, such as TEs. Particularly, although the Juglans mandshurica (V 2.0) has been published, the assembly and annotation

data were still unpublic. Thus, it is difficult for us to further carry out the comparative genome analysis for these two assemblies for this species.

(2) In this paper, the author focused on uncovering genes in the biosynthesis of juglone. They also provided data on metabolite and transcriptome. E.g., line 350, there were 146 candidate genes encoding the enzymes for juglone biosynthesis. However, theses genes were not proved by using the metabolic and transcriptomic data. The author just put the two parts separately

Response: The correlation analysis was performed, and the genes involved in juglone have been added to the Supplementary Table S14. Particularly, 16 of 146 key genes were significantly correlated with the juglone ($r > 0.8$ or < -0.8), including 2 genes encoding decarboxylase and 14 genes belonging to CYP450 family. The contents have been added to the manuscript. See line 270-271.

(3) I noticed that there was a hic matrix provided in Fig1. The 3d-DNA was used to anchor contigs into super scaffolds. This software could not provide this HiC matrix plot. So, what software did the author use for this HiC matrix plot and scaffold anchoring
Response: Thanks for your comments. It is our mistake. The detail methods have been corrected and added to the Part of "Genome assembly". See line 110-111.

(4) I am very surprised there was only 484 Mb of the genome size when using K-mer estimation. But the final size of its genome was 548 Mb. In Zhao`s paper, the final genome size is also 548 Mb! However, there was 578.1 Mb of the genome sized when using K-mer estimation. I am confused with these data, which one is solid? The author argued that heterozygosity made the difference between genome estimation and the final assembled size. If so, I think the author should try haplotype-resolved genome assembly of Juglans mandshurica. The data is enough to perform such analysis. Additionally, there was 27901 genes predicted in Zhao`s paper, but 40,453 genes were predicted in this paper (I think 40,453 would be more solid with our experience). In my opinion, the author should say something between these data, which one is more solid? The author should make a BUSCO analysis for the predicted genes.
Response: Here, the survey analysis we performed based on the obtained HiFi reads (~26x sequence depth), 14.62 Gb in length, and the final genome size, repeat sequences and heterozygosity rate were 547.99 Mb, 48.78% and 0.77%, respectively. The predicted genome size was similar to the assembly, but there is still certain degree of heterozygosity, which indicated that sequencing quality and depth would make the difference between genome estimation and the final assembled size. The relative contents have been added to the Supplementary Table S1. In present study, it is obvious that the contig N50 (21.4 Mb) and complete BUSCO (98.3%) of Juglans mandshurica (V 3.0, 40453 genes) were significantly higher than that in previously reported assemblies (6.5 Mb, 92%, 27901 genes) (Table 1), and the complete BUSCO score was 1,588, significantly higher than a recent genome assembly of 1,375, indicating a relatively high-quality assembly for this species in present study.

(5) Line297, 4dTV was not the first time to come in this MS
Response: It has been corrected. See line 218-219.

(6) Line350, how did the author find 146 candidate genes encoding the enzymes for juglone biosynthesis? Only using Arabidopsis seed sequences to perform blastp? If so, the expansion analysis of CYP was not reasonable. E-value of 1e-5 is quite large and this number of course will cause expansions in your gene family analysis.
Response: The detail has been added to the "Functional gene analysis" Part. See line 844-849. Firstly, the identified CYP450 genes have conserved CYP450 domain. Then, from the intersection set between the obtained CYP450 genes and expanded gene families, 14 expanded CYP450 genes were further identified. Particularly, the expression pattern of these the majority of CYP450 genes were consist with the juglone accumulation.

(7) Finally, the author should make a concentration of the whole manuscript, but not just put all data together.
Response: The manuscript has been made a concentration.

Response to Reviwer3:
(1) In the present article, Li et al. describe the application of genomics, transcriptomics, and metabolomics approaches to decipher the biosynthesis of juglone and lipid in the Manchurian walnut.

Even if the work seems to be funded on a great amount of data, the manuscript misses clarity in the methods and results. First of all, they did not follow the journal guidelines for a research article. There is a Data description part that would be more a Background. Then, they combined methods and results together in a way that sometimes it is very difficult to understand the logic behind the experiments. Just this will be enough to justify Major Revision. Without any structure, the paper cannot be read and understood adequately. For instance, lines 98-105: this part is about the methods for RNA sequencing and metabolite quantification. why is it together with the description of the genome assembly?
Response: Thanks for your comments. The manuscript structure has been made a concentration.

(2) My other main concern is genome assembly. Details about the different assemblies (first PacBIo; then PacBio + Hi-C) are missing (e.g., N50, n. of contigs, n. scaffolds). If they are cited in the text, it is very confusing to understand the different steps of assembling and how the combination of the two technologies actually helped. For instance, the paragraph between the lines 118-140 is a mix of methods and results: first, they report the stats about the final assembly, then they talk about the k-mers, then the heterozygosity and why they used Hi-C, and then again the final assembly. Please organize this part better (tables can help). A comparison with other Juglans chromosome-scale assemblies can also be useful for the reader to see how the different species are similar in terms of size, repeat content, genes, etc.
Response: Thanks for your comments. The relative contents have been corrected. See line 98-114. In the present study, the genome comparison between J. mandshurica and previous two version were displayed in Table 1. Furthermore, the genome comparison of J. mandshurica and J. cathayensis was also performed. See Table 2.

(3) line 110: where are the stats of this preliminary assembly?
Response: It has been added to the Table1.

(4) lines 142-145: where are the results of this comparison?
Response: The results have been added to the Analysis Part, See line 160-165. The relative data were shown in Supplementary Table S3 and S4.

(5) lines 160-168: if combining methods and results in one section is allowed by the journal (?), the results about the repeats should be presented after these lines.
Response: The relative content has been reorganized in the manuscript.

(6) lines 195-197: what about the other Juglans species?
Response: The gene distribution in 16 pseudochromosomes of the Juglans species was still uneven [1-2]. The relative contents have been added to the manuscript. See line 152-154.
[1] Zhu T, Wang L, You F M, et al. Sequencing a Juglans regia × J. macrocarpa hybrid yields high-quality genome assemblies of parental species [J]. Horticulture Research, 2019, 6:55.
[2] Annarita M, Monica B, Zaini P A, et al. High-quality chromosome-scale assembly of the walnut (Juglans regia L.) reference genome[J]. Gigaence 9(5): giaa050.

(7) line 223: there are two improved gene annotations for J. regia (see https://academic.oup.com/gigascience/article/9/5/giaa050/5841058?login=true) and J. microcarpa (https://www.nature.com/articles/s41438-019-0139-1). The authors should consider the most updated versions for a better comparison.
Response: Thanks for your comments. Based on the high-quality HiFi sequence data, it is available to carry out the haplotype-resolved genome assembly of Juglans mandshurica. The data is enough to perform such analysis. In the future, we would use the updated versions for a better comparison.

(8) lines 340-347: a figure summarizing these reactions can be helpful for a reader that is not very familiar with metabolomics and biochemistry.
Response: These reactions were displayed in Figure 5a. See line 257.

(9) lines 380-390: are these libraries the same used for the gene annotation? If yes, why don't have a unique paragraph describing the methods of RNA-sequencing? Again, very confusing.
Response: These contents have been corrected. For RNA-seq, the sequencing and library construction were shown in "RNA-seq sequencing and data analysis" methods Part. See line 759-778. For Iso-seq, the sequencing and library construction were shown in "RNA extract and Iso-Seq Sequencing" methods Part. See line 640-656.

(10) line 465: The TFs found are common in many biochemical reactions. I think a reference can help

support your conclusion, or I would not be so sure of your conclusion.
Response: Thanks for your comments. The relative references have been added to the Discussion Part. See line 550-552.

(11) line 534: what about 'better' instead of greater.
Response: It has been corrected. See line 400.

(12) line 588: kernels
Response: It has been corrected. See line 454.

(13) line 608: delete where the and replace with whose.
Response: The sentence has been corrected. See line 476-477.

(14) line 610: instead of was, maybe presents?
Response: The sentence has been corrected. See line 476-477.

Close
