# Supplementary material for: The Manchurian Walnut Genome: Insights into Juglone and Lipid Biosynthesis
Source: Gigascience. 2022 Jun 28;11:giac057. doi: 10.1093/gigascience/giac057 (PMC9239856; doi:10.1093/gigascience/giac057)
Supplement: giac057_Response_to_Reviewer_Comments_Revision_1 [file giac057_response_to_reviewer_comments_revision_1.pdf]

Author's Response To Reviewer Comments

Close

Response to Reviwer1:
(1) 1. The author should compare the genomes of Juglans species (Marrano et al., 2021; Zhu et al., 2019; Zhang et al., 2020). As some genomes from other walnuts have been available (Marrano et al., 2021; Zhu et al., 2019; Zhang et al., 2020), it is necessary to perform a comparison using multiple genomes to detect some structural variation (SV). The comparison may give some interesting discoveries. In my opinion, the author should at least compare the genome of this species with other published species, such as Juglans regia (https://academic.oup.com/gigascience/article/9/5/giaa050/5841058?login=true), J. microcarpa (https://www.nature.com/articles/s41438-019-0139-1) to explain the differences in traits between the two species, but there were no data in the revised version.
Response: Thanks for your comments. The relative content has been added. See line 284-305.

(2) This manuscript was related to Lipid Biosynthesis. I question the value of the J. mandshurica oil compared to the relative species J. regia, J. mandshurica is more value for their resistance, medicinal crop, rootstock traits. J. mandshurica oil value is less than common walnut (J. regia).
Response: Thanks for your comments. The walnut embryos of J. mandshurica has a high fatty acid content, which is composed of linoleic acid, oleic acid, linolenic acid, palmitic acid and stearic acid. Recently, the oil content of J. mandshurica was defined as 68% in the "Catalogue of main tree species for forestry biomass energy" from National Forestry and Grassland Administration (http://www.gov.cn/xinwen/2017-11/17/content_5240492.htm). In previous study, it revealed that a mature J. regia embryo accumulated 69% oil in Pei's paper [1]. Therefore, the oil content of J. mandshurica and J. regia was similar, and they are important woody oilseed tree species. In addition, it is reported that in J. mandshurica, other tissues, including roots, stems, leaves, branches, and bark may also have significant medicinal value [2].
[1] R Huang, Zhou Y, J Zhang, et al. Transcriptome Analysis of Walnut (Juglans regia L.) Embryos Reveals Key Developmental Stages and Genes Involved in Lipid Biosynthesis and Polyunsaturated Fatty Acid Metabolism[J]. Journal of Agricultural and Food Chemistry, 2020, 69(1).
[2] Zhang, S., Zhan, W., Zhang, H., Song, S., Qu, X. et al. (2022). Genetic Improvement in Juglans mandshurica and Its Uses in China: Current Status and Future Prospects. Phyton-International Journal of Experimental Botany, 91(3), 489–505.

(3) What is the role of the authors in listing the chemical equations instead of English name in Figure 5?
Response: Thanks for your comments. The chemical formula can clearly show the structures of various compounds, especially the juglone.

(4) Line 447: Please add the Figure cite of these sentences.
Response: Thanks for your comments. The Figure cite has been added. See line 471.

(5) Figure7: Which tissues were used in comparative transcript analysis of genes involved in lipid biosynthesis of developing fruit.
Response: Thanks for your comments. The walnut embryo was used to perform the comparative transcript analysis of genes involved in lipid biosynthesis of developing fruit.

Response to Reviwer3:
(1) I quickly looked at the authors' responses. It seems they worked to a better version of the manuscript, even if I haven't had the time for reading this new version carefully. It might need some English revision. I have one only question: why the contig and scaffold N50 size does not change from the PacBio assembly to the Hi-C assembly? See lines 104-114.
Response: Thanks for your comments. The language has been revised. For the contig N50 and scaffold N50, we need to explain that the HiFi reads has high accuracy (> 99%); the contig-level PacBio assembly was obtained based on high-quality reads, and contig N50 was calculated based on reads; the chromosome-scale scaffolds were obtained based on contigs using Hi-C technology, and scaffold N50

was calculated based on contigs, rather than the reads. Therefore, the change of contig and scaffold N50 is very small (nearly unchanged) by Hi-C assembly. Similar results were also found in previous study.

Close
