# Supplementary material for: The Manchurian Walnut Genome: Insights into Juglone and Lipid Biosynthesis
Source: Gigascience. 2022 Jun 28;11:giac057. doi: 10.1093/gigascience/giac057 (PMC9239856; doi:10.1093/gigascience/giac057)
Supplement: giac057_Reviewer_1_Report_Original_Submission — Peng Zhao -- 1/19/2022 Reviewed [file giac057_reviewer_1_report_original_submission.pdf]

Reviewer Report

Title: The Manchurian Walnut Genome: Insights into Juglone and Lipid Biosynthesis

Version: Original Submission **Date:** 1/19/2022

Reviewer name: Peng Zhao

Reviewer Comments to Author:

This manuscript entitled "The Manchurian Walnut Genome: Insights into Juglone and Lipid Biosynthesis" assembled a high-quality chromosome-scale reference genome of walnut family (Juglandaceae) species Juglans mandshurica. The author combined the genome assembly, transcriptome, and metabolic data to construct gene expression profiling, network and identified genes regulated the juglone and lipid biosynthesis. In a total, this manuscript is useful genome resources for further genome and evolution studies of walnut species and other Juglandaceae species. The authors need to address the following issues.

1. A high-quality genome is an important genetic resource for the improvement of horticultural traits in perennial crops. A combination of long reads (Nanopore sequencing platform), Illumina and Hi-C auxiliary assembly can be used to produce a high-quality, chromosome-level genome. Recently, the availability of high-throughput sequencing has accelerated the publication of the genomes of walnut (Juglans) species and hybrids (J. regia Ã— J. microcarpa) (MartÃ-nez-GarcÃ-a et al., 2016; Stevens et al., 2018; Bai et al., 2018; Zhang et al., 2020; Yan et al., 2021). For Juglans mandshurica, it has been published three assembly genomes, including scaffold lever (Bai et al., 2018; Stevens et al., 2018) and high-quality chromosome-level (Yan et al., 2021). Therefore, it is necessary to perform a comparison using multiple genomes, especially previous versions of J. mandshurica. To compare the GC content and size of genome (in this study, genome size of ~548.7 Mb and Yan et al., 2021 genome size of ~548.5 Mb, only 0.2 Mb difference), which are did not showing anything about the high-quality of the genome assembly from Line 601 to Line 613. Furthermore, the author described "the best available reference genome of J. mandshurica" is from Line 611 to Line 613 is inaccurate statement, please revise it. It is necessary to compare the scaffolds N50 size, mapped and anchored percentage, and BUSCOs. The comparison may give some interesting discoveries, such as TEs, InDels and SVs between two genomes.
Bai, W. N., Yan, P. C., Zhang, B. W., Woeste, K. E., Lin, K., & Zhang, D. Y. (2018). Demographically idiosyncratic responses to climate change and rapid Pleistocene diversification of the walnut genus Juglans (Juglandaceae) revealed by whole genome sequences. New Phytologist, 217(4), 1726-1736.
MartÃ-nez-GarcÃ-a, P. J., Crepeau, M. W., Puiu, D., Gonzalez-Ibeas, D., Whalen, J., Stevens, K. A., … & Neale, D. B. (2016). The walnut (Juglans regia) genome sequence reveals diversity in genes coding for the biosynthesis of non-structural polyphenols. The Plant Journal, 87(5), 507-532.
Stevens, K. A., Woeste, K., Chakraborty, S., Crepeau, M. W., Leslie, C. A., MartÃ-nez-GarcÃ-a, P. J., ... & Kluepfel, D. (2018). Genomic variation among and within six Juglans species. G3-Genes Genomes Genetics, 8(7), 2153-2165.
Zhang, J., Zhang, W., Ji, F., Qiu, J., Song, X., Bu, D., ... & Pei, D. (2020). A high-quality walnut genome assembly reveals extensive gene expression divergences after whole-genome duplication. Plant

Biotechnology Journal, 18(9), 1848-1850.
Yan F, Xi RM, She RX, Chen PP, Yan YJ, Yang G, Dang M, Yue M, Pei D, Woeste K, Zhao Peng. (2021) Improved de novo chromosome-level genome assembly of the vulnerable walnut tree Juglans mandshurica reveals gene family evolution and possible genome basis of resistance to lesion nematode. Molecular Ecology Resources, DOI:10.1111/1755-0998.13394
2. In this study, the author used total of 15 species to analyze the comparison genomes, including seven Juglans species. I question the value of the phylogenetic analysis of fifteen species from across all angiosperm lineages. This does not seem to provide any new significant insights. It is necessary to perform a comparison expansion and contraction gene families within Juglans species. J. mandshurica a synonym of J. cathayensis Dode in Flora of China. However, a significantly different for contraction gene families between J. mandshurica (405) and J. cathayensis (2193), which is not consistently with the taxa and gene family evolution. The authors need to explain the reasons for the two J. mandshurica genome and J. cathayensis genome. What is the deep relationship for J. mandshurica and J. cathayensis based on the high-quality genomes?
3. The collinearity of J. mandshurica and J. regia genomes were compared using 16 chromosomes in Figure 2. I question the order of chromosome numbers J. mandshurica and J. regia genomes are uniformity or not? It is important of order of chromosome numbers for two Juglans species in the length, gene distributions, and collinearity.
4. The whole-genome duplication events (WGD) showed that the peak of Ks from 0-0.5 and 0-0.25 using Ks and 4DTV, respectively. It is necessary to compare the WGD in previous walnut genomes studies and discuss it.
5. Along with the development of sequencing technology, to get a relative high-quality genome is not a challenge now. I would expect to see some deeper analyses based on the genome assembly. For example, to explain why J. mandshurica is abundant juglone, potential as a medicinal crop. Furthermore, the other main weakness of this manuscript is short of comprehensive discussions and interpretations for the relationship between genomic characters and biology of the J. mandshurica. In 2016, Martinez-Garcia et al report the complex pathways required for the synthesis of polyphenols using the J. regia genome. However, the authors of this article did not conduct any comparative research on the relevant complex pathways results, such as juglone and lipid biosynthesis.
6. For the transcriptome and metabolic samples, the authors collected J. mandshurica fruits in different development periods. S1-S4 indicate the fruit collected at 30 days (S1 stage), 50 days (S2 stage), 70 days (S3 stage) and 90 days (S4 stage) after natural pollination. It is necessary to describe the walnut exocarp and walnut embryos in each figure.
7. Did the author used fossil data to double check the divergence time estimation analysis? The outgroup species should be different genus species within Ranunculaceae family will be good for divergence time estimate analysis. Please explain why the phylogenomic analysis showed that the J. mandshurica was closest to J. cathayensis (divergence at ~13.8 mya).
8. Use of English language. I have noticed the manuscript is written in poor English with many grammatical errors. Please revise the describes logically, organized, and be concise and to the point of RESULTS parts, especially for the "Metabolite profiling and transcriptomics of walnut exocarp development" and "Gene discovery for lipid biosynthesis and oil body formation".
Other small changes:

Please check the numbers consistent with scientific count in the text.
Figure legends: please describe the details (legend) of small letter.
It is necessary to improve the quality of Supplementary Figures including legends and data supports.

Methods

Are the methods appropriate to the aims of the study, are they well described, and are necessary controls included? Choose an item.

Conclusions

Are the conclusions adequately supported by the data shown? Choose an item.

Reporting Standards

Does the manuscript adhere to the journal's guidelines on minimum standards of reporting? Choose an item.

Choose an item.

Statistics

Are you able to assess all statistics in the manuscript, including the appropriateness of statistical tests used? Choose an item.

Quality of Written English

Please indicate the quality of language in the manuscript: Choose an item.

Declaration of Competing Interests

Please complete a declaration of competing interests, considering the following questions:

- Have you in the past five years received reimbursements, fees, funding, or salary from an organisation that may in any way gain or lose financially from the publication of this manuscript, either now or in the future?
- Do you hold any stocks or shares in an organisation that may in any way gain or lose financially from the publication of this manuscript, either now or in the future?
- Do you hold or are you currently applying for any patents relating to the content of the manuscript?
- Have you received reimbursements, fees, funding, or salary from an organization that holds or has applied for patents relating to the content of the manuscript?
- Do you have any other financial competing interests?
- Do you have any non-financial competing interests in relation to this paper?

If you can answer no to all of the above, write 'I declare that I have no competing interests' below. If your reply is yes to any, please give details below.

I declare that I have no competing interests

I agree to the open peer review policy of the journal. I understand that my name will be included on my report to the authors and, if the manuscript is accepted for publication, my named report including any attachments I upload will be posted on the website along with the authors' responses. I agree for my report to be made available under an Open Access Creative Commons CC-BY license (http://creativecommons.org/licenses/by/4.0/). I understand that any comments which I do not wish to be included in my named report can be included as confidential comments to the editors, which will not be published.

Choose an item.

To further support our reviewers, we have joined with Publons, where you can gain additional credit to further highlight your hard work (see: https://publons.com/journal/530/gigascience). On publication of this paper, your review will be automatically added to Publons, you can then choose whether or not to claim your Publons credit. I understand this statement.

Yes Choose an item.
