# Supplementary material for: The Manchurian Walnut Genome: Insights into Juglone and Lipid Biosynthesis
Source: Gigascience. 2022 Jun 28;11:giac057. doi: 10.1093/gigascience/giac057 (PMC9239856; doi:10.1093/gigascience/giac057)
Supplement: giac057_Reviewer_2_Report_Original_Submission — Nian Wang -- 1/23/2022 Reviewed [file giac057_reviewer_2_report_original_submission.pdf]

Reviewer Report

Title: The Manchurian Walnut Genome: Insights into Juglone and Lipid Biosynthesis

Version: Original Submission **Date:** 1/23/2022

Reviewer name: Nian Wang

Reviewer Comments to Author:

This manuscript generated a lot of data for a wild walnut Juglans mandshurica. They assembled the genome of this wild species, provided some metabolic and transcriptomic analyses. However, the manuscript was not well-organized and did not attract me. I think this manuscript should be re-organized. Here are my suggestions.

There were several genomes for species in Juglans. In 2021, another group leaded by Zhao from Northwest University, China, also published the genomic data of Juglans mandshurica on molecular ecology resource (DOI: 10.1111/1755-0998.13394). In that paper, the author said Juglans mandshurica is a synonym of J. cathayensis Dode. So, what is the different between this study to Zhao`s paper? Though I don`t agree Juglans mandshurica is a synonym of J. cathayensis, but the material used in Zhao`s paper was Juglans mandshurica (not J. cathayensis). If the two papers published the same genome, then there was no novel knowledge for this manuscript in the genome level. Additionally, there were also some other genomes from Juglans released in recent years (Such as from J. regia genome from Pei`s group). In my opinion, the author should not ignore these genomes, at least, a comparison among these genomes should be conducted, especially for your and Zhao`s work.

In this paper, the author focused on uncovering genes in the biosynthesis of juglone. They also provided data on metabolite and transcriptome. E.g., line 350, there were 146 candidate genes encoding the enzymes for juglone biosynthesis. However, theses genes were not proved by using the metabolic and transcriptomic data. The author just put the two parts separately

I noticed that there was a hic matrix provided in Fig1. The 3d-DNA was used to anchor contigs into super scaffolds. This software could not provide this HiC matrix plot. So, what software did the author use for this HiC matrix plot and scaffold anchoring.

I am very surprised there was only 484 Mb of the genome size when using K-mer estimation. But the final size of its genome was 548 Mb. In Zhao`s paper, the final genome size is also 548 Mb! However, there was 578.1 Mb of the genome sized when using K-mer estimation. I am confused with these data, which one is solid? The author argued that heterozygosity made the difference between genome estimation and the final assembled size. If so, I think the author should try haplotype-resolved genome assembly of Juglans mandshurica. The data is enough to perform such analysis. Additionally, there was 27901 genes predicted in Zhao`s paper, but 40,453 genes were predicted in this paper (I think 40,453 would be more solid with our experience). In my opinion, the author should say something between these data, which one is more solid? The author should make a BUSCO analysis for the predicted genes.

Line297, 4dTV was not the first time to come in this MS

Line350, how did the author find 146 candidate genes encoding the enzymes for juglone biosynthesis? Only using Arabidopsis seed sequences to perform blastp? If so, the expansion analysis of CYP was not

reasonable. E-value of 1e-5 is quite large and this number of course will cause expansions in your gene family analysis.
Finally, the author should make a concentration of the whole manuscript, but not just put all data together.

Methods

Are the methods appropriate to the aims of the study, are they well described, and are necessary controls included? Choose an item.

Conclusions

Are the conclusions adequately supported by the data shown? Choose an item.

Reporting Standards

Does the manuscript adhere to the journal's guidelines on minimum standards of reporting? Choose an item.

Choose an item.

Statistics

Are you able to assess all statistics in the manuscript, including the appropriateness of statistical tests used? Choose an item.

Quality of Written English

Please indicate the quality of language in the manuscript: Choose an item.

Declaration of Competing Interests

Please complete a declaration of competing interests, considering the following questions:

- Have you in the past five years received reimbursements, fees, funding, or salary from an organisation that may in any way gain or lose financially from the publication of this manuscript, either now or in the future?
- Do you hold any stocks or shares in an organisation that may in any way gain or lose financially from the publication of this manuscript, either now or in the future?
- Do you hold or are you currently applying for any patents relating to the content of the manuscript?
- Have you received reimbursements, fees, funding, or salary from an organization that holds or has applied for patents relating to the content of the manuscript?
- Do you have any other financial competing interests?

- Do you have any non-financial competing interests in relation to this paper?

If you can answer no to all of the above, write 'I declare that I have no competing interests' below. If your reply is yes to any, please give details below.

I declare that I have no competing interests

I agree to the open peer review policy of the journal. I understand that my name will be included on my report to the authors and, if the manuscript is accepted for publication, my named report including any attachments I upload will be posted on the website along with the authors' responses. I agree for my report to be made available under an Open Access Creative Commons CC-BY license (http://creativecommons.org/licenses/by/4.0/). I understand that any comments which I do not wish to be included in my named report can be included as confidential comments to the editors, which will not be published.

Choose an item.

To further support our reviewers, we have joined with Publons, where you can gain additional credit to further highlight your hard work (see: https://publons.com/journal/530/gigascience). On publication of this paper, your review will be automatically added to Publons, you can then choose whether or not to claim your Publons credit. I understand this statement.

Yes Choose an item.
