# Supplementary material for: The Manchurian Walnut Genome: Insights into Juglone and Lipid Biosynthesis
Source: Gigascience. 2022 Jun 28;11:giac057. doi: 10.1093/gigascience/giac057 (PMC9239856; doi:10.1093/gigascience/giac057)
Supplement: giac057_Reviewer_3_Report_Original_Submission — Annarita Marrano, Ph. D. -- 2/2/2022 Reviewed [file giac057_reviewer_3_report_original_submission.pdf]

Reviewer Report

Title: The Manchurian Walnut Genome: Insights into Juglone and Lipid Biosynthesis

Version: Original Submission **Date:** 2/2/2022

Reviewer name: Annarita Marrano, Ph. D.

Reviewer Comments to Author:

In the present article, Li et al. describe the application of genomics, transcriptomics, and metabolomics approaches to decipher the biosynthesis of juglone and lipid in the Manchurian walnut. Even if the work seems to be funded on a great amount of data, the manuscript misses clarity in the methods and results. First of all, they did not follow the journal guidelines for a research article. There is a Data description part that would be more a Background. Then, they combined methods and results together in a way that sometimes it is very difficult to understand the logic behind the experiments. Just this will be enough to justify Major Revision. Without any structure, the paper cannot be read and understood adequately. For instance, lines 98-105: this part is about the methods for RNA sequencing and metabolite quantification. why is it together with the description of the genome assembly?
My other main concern is genome assembly. Details about the different assemblies (first PacBIo; then PacBio + Hi-C) are missing (e.g., N50, n. of contigs, n. scaffolds). If they are cited in the text, it is very confusing to understand the different steps of assembling and how the combination of the two technologies actually helped. For instance, the paragraph between the lines 118-140 is a mix of methods and results: first, they report the stats about the final assembly, then they talk about the k-mers, then the heterozygosity and why they used Hi-C, and then again the final assembly. Please organize this part better (tables can help). A comparison with other Juglans chromosome-scale assemblies can also be useful for the reader to see how the different species are similar in terms of size, repeat content, genes, etc.
Therefore, I recommend Major Revisions.
More detailed comments:
- line 110: where are the stats of this preliminary assembly?
- lines 142-145: where are the results of this comparison?
- lines 160-168: if combining methods and results in one section is allowed by the journal (?), the results about the repeats should be presented after these lines.
- lines 195-197: what about the other juglans species?
- line 223: there are two improved gene annotations for J. regia (see https://academic.oup.com/gigascience/article/9/5/giaa050/5841058?login=true) and J. microcarpa (https://www.nature.com/articles/s41438-019-0139-1). The authors should consider the most updated versions for a better comparison.
- lines 340-347: a figure summarizing these reactions can be helpful for a reader that is not very familiar with metabolomics and biochemistry.
- lines 380-390: are these libraries the same used for the gene annotation? If yes, why don't have a unique paragraph describing the methods of RNA-sequencing? Again, very confusing.

- line 465: The TFs found are common in many biochemical reactions. I think a reference can help support your conclusion, or I would not be so sure of your conclusion.
- line 534: what about 'better' instead of greater.
- line 588: kernels
- line 608: delete where the and replace with whose.
- line 610: instead of was, maybe presents?

Methods

Are the methods appropriate to the aims of the study, are they well described, and are necessary controls included? Choose an item.

Conclusions

Are the conclusions adequately supported by the data shown? Choose an item.

Reporting Standards

Does the manuscript adhere to the journal's guidelines on minimum standards of reporting? Choose an item.

Choose an item.

Statistics

Are you able to assess all statistics in the manuscript, including the appropriateness of statistical tests used? Choose an item.

Quality of Written English

Please indicate the quality of language in the manuscript: Choose an item.

Declaration of Competing Interests

Please complete a declaration of competing interests, considering the following questions:

- Have you in the past five years received reimbursements, fees, funding, or salary from an organisation that may in any way gain or lose financially from the publication of this manuscript, either now or in the future?
- Do you hold any stocks or shares in an organisation that may in any way gain or lose financially from the publication of this manuscript, either now or in the future?
- Do you hold or are you currently applying for any patents relating to the content of the manuscript?
- Have you received reimbursements, fees, funding, or salary from an organization that holds or has applied for patents relating to the content of the manuscript?

- Do you have any other financial competing interests?
- Do you have any non-financial competing interests in relation to this paper?

If you can answer no to all of the above, write 'I declare that I have no competing interests' below. If your reply is yes to any, please give details below.

I declare that I have no competing interests

I agree to the open peer review policy of the journal. I understand that my name will be included on my report to the authors and, if the manuscript is accepted for publication, my named report including any attachments I upload will be posted on the website along with the authors' responses. I agree for my report to be made available under an Open Access Creative Commons CC-BY license (http://creativecommons.org/licenses/by/4.0/). I understand that any comments which I do not wish to be included in my named report can be included as confidential comments to the editors, which will not be published.

Choose an item.

To further support our reviewers, we have joined with Publons, where you can gain additional credit to further highlight your hard work (see: https://publons.com/journal/530/gigascience). On publication of this paper, your review will be automatically added to Publons, you can then choose whether or not to claim your Publons credit. I understand this statement.

Yes Choose an item.
